# Synthesis, Antibacterial Evaluation, and Computational Studies of a Diverse Set of Linezolid Conjugates

**DOI:** 10.3390/ph15020191

**Published:** 2022-02-03

**Authors:** Riham M. Bokhtia, Adel S. Girgis, Tarek S. Ibrahim, Fatma Rasslan, Eman S. Nossier, Reham F. Barghash, Rajeev Sakhuja, Eatedal H. Abdel-Aal, Siva S. Panda, Amany M. M. Al-Mahmoudy

**Affiliations:** 1 Department of Pharmaceutical Organic Chemistry, Faculty of Pharmacy, Zagazig University, Zagazig 44519, Egypt; rehamabdelrehem@yahoo.com (R.M.B.); eatedalabdelaal@yahoo.com (E.H.A.-A.); amanysinger77@gmail.com (A.M.M.A.-M.); 2 Department of Chemistry and Physics, Augusta University, Augusta, GA 30912, USA; 3 Department of Pesticide Chemistry, National Research Centre, Dokki, Giza 12622, Egypt; girgisas10@yahoo.com (A.S.G.); reham_fawzy@yahoo.com (R.F.B.); 4 Department of Pharmaceutical Chemistry, Faculty of Pharmacy, King Abdulaziz University, Jeddah 21589, Saudi Arabia; tmabrahem@kau.edu.sa; 5 Department of Microbiology and Immunology, Faculty of Pharmacy (Girls), Al Azhar University, Cairo 11651, Egypt; fatma_rasslan@yahoo.com; 6 Department of Pharmaceutical Medicinal Chemistry and Drug Design, Faculty of Pharmacy (Girls), Al-Azhar University, Cairo 11651, Egypt; dr.emannossier@gmail.com; 7 Department of Chemistry, Birla Institute of Technology and Science, Pilani 333031, India; sakhuja.rajeev@gmail.com

**Keywords:** linezolid, conjugates, antibacterial, QSAR, pharmacophore

## Abstract

The development of new antibiotics to treat multidrug-resistant (MDR) bacteria or possess broad-spectrum activity is one of the challenging tasks. Unfortunately, there are not many new antibiotics in clinical trials. So, the molecular hybridization approach could be an effective strategy to develop potential drug candidates using the known scaffolds. We synthesized a total of 31 diverse linezolid conjugates **3**, **5**, **7**, **9**, **11**, **13**, and **15** using our established benzotriazole chemistry with good yield and purity. Some of the synthesized conjugates exhibited promising antibacterial properties against different strains of bacteria. Among all the synthesized compounds, **5d** is the most promising antibacterial agent with MIC 4.5 µM against *S. aureus* and 2.25 µM against *B. subtilis*. Using our experimental data pool, we developed a robust QSAR (*R*^2^ = 0.926, 0.935; *R*^2^cvOO = 0.898, 0.915; *R*^2^cvMO = 0.903, 0.916 for the *S. aureus* and *B. subtilis* models, respectively) and 3D-pharmacophore models. We have also determined the drug-like properties of the synthesized conjugates using computational tools. Our findings provide valuable insight into the possible linezolid-based antibiotic drug candidates.

## 1. Introduction

Antibiotics are among the most clinically used drugs for the treatment of various serious diseases in humans [1,2]. Due to overuse and abuse of antibiotics under the influence of various man-made and external factors, the drug resistance for both Gram-positive and Gram-negative bacteria is increasing at an alarming rate [3,4,5]. Today, MDR has attracted increasing attention because of the recent statistics reported by the World Health Organization (WHO). According to the WHO, around 50,000 patients die from infectious diseases every day worldwide [6]. Oxazolidinones are a new class of antimicrobial agents that have a unique structure and show good activity against different pathogenic bacteria, including methicillin- and vancomycin-resistant staphylococci, vancomycin-resistant enterococci, penicillin-resistant pneumococci, and anaerobes [7]. Oxazolidinone antibacterial agents are new synthetic antibacterial agents that have been explored after sulfonamides and quinolones [8]. 5-(Aminomethyl)-3-(3-fluoro-4-morpholinophenyl)oxazolidin-2-one (Linezolid) can be considered as the first member of the class of oxazolidinone antibiotics. Linezolid’s mode of action is to prevent the synthesis of bacterial protein via binding to rRNA on both the 30S and 50S ribosomal subunits [9]. It inhibits the formation of the initiation complex, which can reduce the length of the developed peptide chains and decrease the rate of the translation reaction [9]. Because of the unique site of inhibition, cross-resistance to other protein synthesis inhibitors has not yet been demonstrated [10]. Linezolid may also prevent the expression of virulence elements, leading to decreased toxins produced by Gram-positive pathogens [11].

Although linezolid was approved by the US Food and Drug Administration in 2000 as an antibiotic, oxazolidinones are considered a potential building block in the development of drug candidates for the last 40 years [12,13]. Despite extensive efforts that have been undertaken to synthesize more potential and safer analogs, linezolid remained the only FDA-approved antibiotic in this class for more than 20 years. Currently, there are several oxazolidinones in the process of clinical trials [14].

The ideal goal is to develop a new potential antibiotic that should show broad-spectrum effect, enhanced efficacy, a better safety profile, and the possibility for use by multiple routes of administration. To achieve the desired target, molecular hybridization is one of the attractive strategies.

Molecular hybridization is about modifying the parent structural motifs to derive an array of significantly potent candidates with minimal toxic or side effects and to avoid any possible drug resistance development. This approach seemed attractive because it does not require the discovery of new antibacterial scaffolds or validation of novel biological targets, which has proven to be an extremely difficult and time-consuming task.

**Figure 1:**
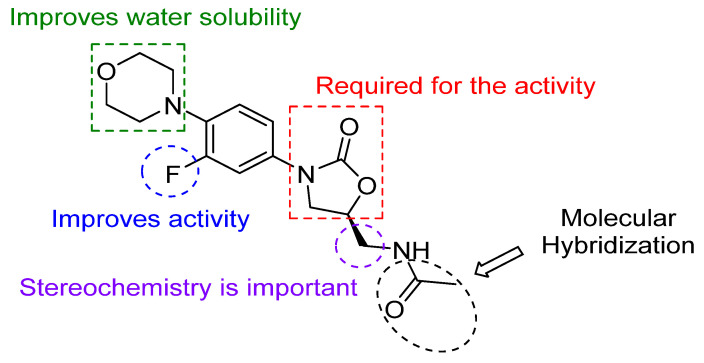
Structure of Linezolid and its important scaffolds.

In continuation to our current efforts in drug development [15,16,17,18,19,20], we utilized a molecular modification approach for enhancing the drug-like properties of linezolid; herein, we synthesized a series of novel linezolid conjugates via NH_2_ acylation of de-acetyl linezolid **1** using a series of different benzotriazole-activated molecules. Recently, Rahman and coworkers reported the synthesis of linezolid analogs with different C5-acylamino substituents using EDC and DMAP as coupling reagents [21]. The reported method gave moderate yield after HPLC purification. Our method of *N*-acylation using benzotriazole chemistry [22] has several advantages, such as quantitative yield and high purity without the use of column chromatography. In this present work, we did not make any change to the essential components of the linezolid structure (Figure 1). We introduced amino acids as part of the conjugates, as amino acids are well known for improving the antibacterial property and cell permeability [23,24,25,26].

Moreover, acylation using different benzotriazole-activated aromatic acids and alkylation using a series of substituted benzyl halides, and 3-nitroxyl propyl bromide have been carried out to study the structure-activity relationship. On the other hand, the reaction of deacetyl linezolid **1** with 3-bromopropyl nitrate is supposed to produce a NO-releasing compound, which acts as a delivery system of NO that plays an integral role in defending against a wide range of pathogens [27] and results in synergizing the deacetyl linezolid antimicrobial effect.

## 2. Results and Discussion

### 2.1. Chemistry

**Scheme 1:**
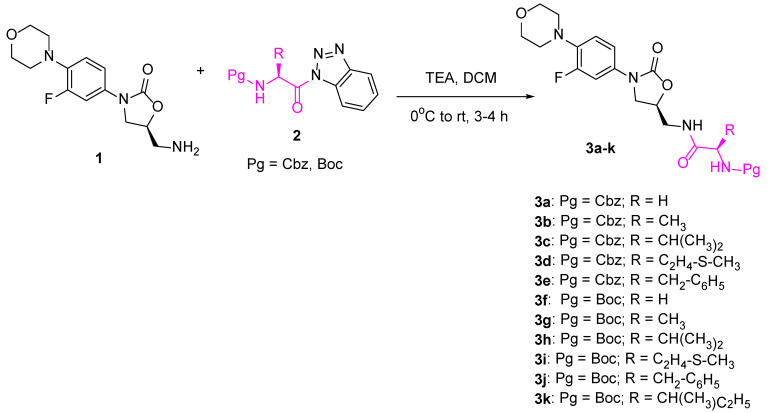
Synthesis of linezolid-amino acid conjugates **3a–k**.

The current study is focused on synthesizing some linezolid conjugates **3a–k** by reacting linezolid **1** with benzotriazole-activated protected amino acids **2a–k** in dichloromethane (DCM) containing triethylamine at 0 °C for 3–4 h (Scheme 1).

**Scheme 2:**
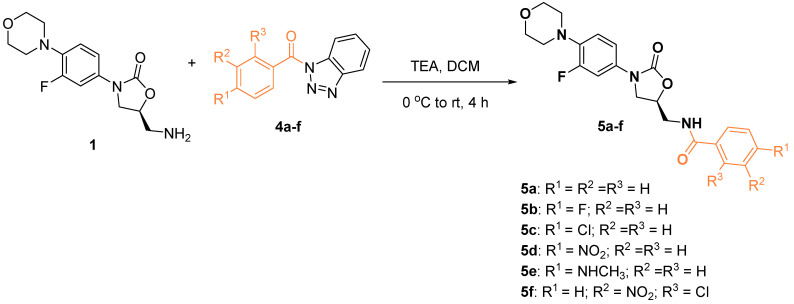
Synthesis of linezolid-aromatic acid conjugates **5a–f**.

**Scheme 3:**
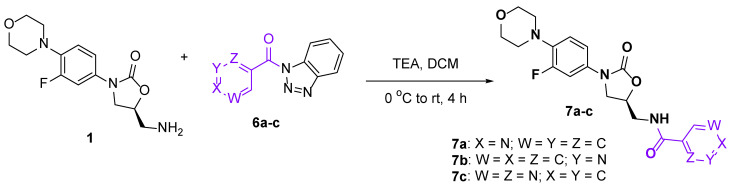
Synthesis of linezolid-heteroaromatic acid conjugates **7a–c**.

To diversify the pool of conjugates and study the structure-activity relationship, we have prepared the linezolid conjugates with substituted aromatic and heteroaromatic acids using our optimized benzotriazole chemistry (Scheme 2 and Scheme 3).

Compound **5a** was prepared from benzoyl benzotriazole in an excellent yield (98%) compared with the reported yield of the same compound (54%) using benzoyl chloride [28]. Moreover, compound **5c** was prepared in yield 30% using 4-chlorobenzoyl chloride, which reflects the advantage of utilizing benzotriazole chemistry in obtaining pure products in high yields.

**Scheme 4:**
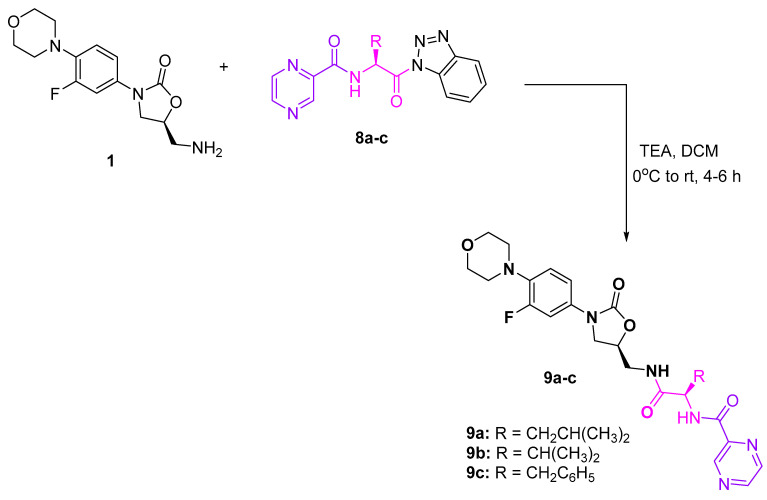
Synthesis of linezolid hybrid conjugates **9a–c**.

To synthesize a set of hybrid conjugates of linezolid, amino acid, and heteroaromatic acid, we treated benzotriazole-activated pyrazinoic acid-amino acid conjugate **8a–c** (which we reported in our previous report [19]) in DCM in the presence of TEA at 0 °C for 4–6 h (Scheme 4).

**Scheme 5:**
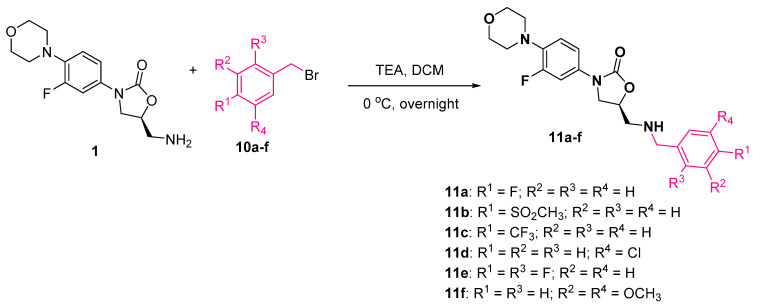
Synthesis of linezolid conjugates **11a–f**.

Further, we treated de-acetyl linezolid **1** with substituted aryl bromides **10a–f** in DCM in presence of TEA at 0 °C overnight. The desired conjugates **11a–f** were isolated in good yields after purification using column chromatography (Scheme 5).

**Scheme 6:**
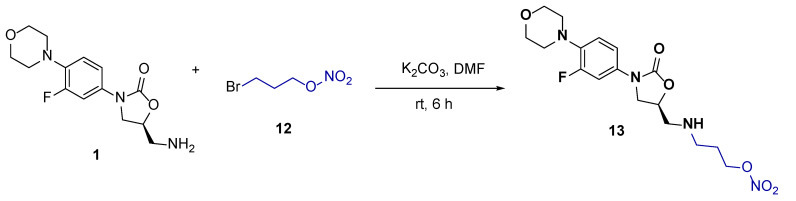
Synthesis of linezolid conjugates with 3-bromopropyl nitrate.

To introduce the nitrogen-releasing component in the linezolid conjugate, we reacted linezolid **1** with 3-bromopropyl nitrate **12** in DMF in the presence of potassium carbonate (K_2_CO_3_) at room temperature for 6 h (Scheme 6).

**Scheme 7:**
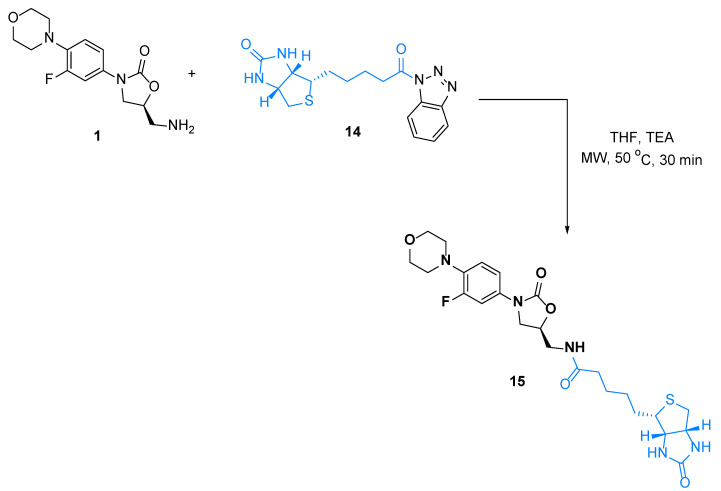
Synthesis of linezolid conjugates with biotin.

Linezolid is known as a highly tolerated antibiotic and is used for various complicated infections. This drug has lots of potentials; to better understand the role of this drug in various treatments, we coupled linezolid with biotin. Biotin is usually used as a marker to unfold the mode of action of drugs. We treated de-acetyl linezolid **1** with benzotriazole activated biotin **14** [23] in THF under microwave irradiation in the presence of TEA at 50 °C for 30 min to obtain the desired conjugate **15** in good yield (Scheme 7). We tried this reaction both in conventional heating and microwave heating, we found that the use of microwave gave us better yield with high purity.

### 2.2. Biological Studies

#### 2.2.1. Antimicrobial Studies

The synthesized linezolid conjugates (**3a–k**, **5a–f, 7a–c**, **9a–c**, **11a–f**, **13,** and **15**) were subjected for antimicrobial properties determination against Gram-positive (*Staphylococcus aureus* ATCC 6538, *Bacillus subtilis* ATCC 6633) and Gram-negative (*Pseudomonas aeruginosa* ATCC 15692, *Escherichia coli* ATCC 47076) bacteria by the standard technique [29,30]. From the antimicrobial properties revealed (Table 1), it is noticeable that the synthesized agents still possess better activity towards the tested Gram-positive bacteria than the Gram-negative ones in similar behavior to their parent antibiotic (standard reference, linezolid). It has also been noticed that compound **5d** (R^1^ = NO_2_, R^2^ = R^3^ = H) is the most effective agent prepared with comparable potency against *S. aureus* relative to the parent antibiotic (MIC = 4.5, 5.929 μM for **5d** and linezolid, respectively). Considerable antimicrobial properties were also noticed by compounds **5a**, **5b,** and **5e** against *S. aureus* (MIC = 9.336–10.015 μM).

Compound **5d** also exhibited promising antimicrobial properties against *B. subtilis* (MIC = 2.25 μM). Similar observations were also noticed by compounds **5a**, **5b** and **5e** (MIC = 2.334–2.504 μM). Compounds **5c**, **7a–c** also showed considerable antimicrobial properties against *B. subtilis* (MIC = 4.61–4.995 μM).

Linezolid is an effective antibiotic against Gram-positive bacteria; however, some of the synthesized agents exhibited antimicrobial properties against *P. aeruginosa* (Gram-negative bacteria), with slightly enhanced efficacy compared to that of the parent. Compound **7a** was the most effective agent prepared with moderate antimicrobial properties revealed against *P. aeruginosa* (MIC = 19.98, 94.857 μM for **7a** and linezolid, respectively). Most of the synthesized agents reveal mild properties against *P. aeruginosa* (MIC = 58.332–80.323 μM).

Based on the antimicrobial properties observed, some SAR (structure-activity relationships) were noticed. Generally, the synthesized agents **5** and **7** were the most effective conjugates synthesized against the Gram-positive bacteria tested. In other words, attachment of either aromatic or heteroaromatic acids with linezolid can afford a promising antimicrobial agent. Attachment of an electron-withdrawing group to the aroyl fragment enhanced the antimicrobial properties observed, as shown by compounds **5d** and **5b** “possessing *p*-nitro and *p*-fluorobenzoyl substituent, respectively” (MIC = 4.5, 9.583; 2.25, 2.396 μM for compounds **5d** and **5b** against *S. aureus* and *B. subtilis*, respectively). The π-deficient heterocycle (pyridinyl and pyrazinyl) “which possessed electron-withdrawing effects” also enhanced the revealed antimicrobial properties against the Gram-positive bacteria, as revealed by compounds **7a–c** (MIC = 19.93–19.98; 4.983–4.995 μM for compounds **7a–c** against *S. aureus* and *B. subtilis*, respectively).

#### 2.2.2. Cytotoxicity Studies

Safety profile against the RPE1 (human immortalized retinal pigment epithelial cell line) was investigated, along with the antiproliferation activity studies of the synthesized compounds by following the standard MTT bioassay. None of the synthesized agents showed any signs of toxicity towards the tested cell line up to 100 µM (Supplementary Figure S1).

### 2.3. Computational Studies

#### 2.3.1. QSAR Studies

Physico-chemical properties (descriptors) can determine the correlation between the biological/antimicrobial properties and chemical entities quantitatively in mathematical equations expressed in the QSAR model, which can determine the most important features needed for the antimicrobial properties [31,32].

#### 2.3.2. *S. Aureus*

The four-descriptor model describes the antimicrobial properties of the tested compounds against *S. aureus* with a broad range of biological observations expressed in log (MIC, μM) values “observed log (MIC, μM) = 0.653–2.072, predicted log (MIC, μM) = 0.793–2.057 i.e., observed (MIC, μM) = 4.5–117.948, predicted (MIC, μM) = 6.212–114.088”. The correlation coefficient values support the accuracy/goodness of the model (*R*^2^ = 0.926, *R*^2^cvOO = 0.898, *R*^2^cvMO = 0.903) (Supplementary Tables S1–S3, Figure S2). The partial surface area for atom H is a charge-related descriptor with a high criterion value among the other model’s descriptors (*t* = 7.89) with a high mathematically coefficient value (3.29174). This is an indication of the low antimicrobial efficacy of an agent with a high mathematical descriptor value and vice versa, as shown by compounds **5d** and **11f** (descriptor value = 0.465, 0.640; predicted MIC = 6.212, 114.088 μM for **5d** and **11f**, respectively). The partial positively (PPSA1)/negatively (PNSA1) charged surface area can be determined by the Equations (1) and (2) [33].

(1) $$\mathrm{PPSA}1=\sum_{A}S_{A}\;\;\;\;\;\;\;\;\;\;\;A\in \left\{\delta_{A}>0\right\}$$

(2) $$\mathrm{PNSA}1=\sum_{A}S_{A}\;\;\;\;\;\;\;\;\;\;\;A\in \left\{\delta_{A}>0\right\}$$

S_A_ is either the positively or negatively charged solvent accessible surface area.

The difference (DPSA) between the total partial charged positive and negative surface areas is also a charge-related descriptor (*t* = 6.954). Although its low coefficient value (0.00087934) and its high mathematical value affords capability for controlling the estimated antimicrobial observations as exhibited in compounds **3e** and **5d** (descriptor value = 1308.602, 847.5723 corresponding to estimated MIC = 49.005, 6.212 μM for compounds **3e** and **5d**, respectively). The descriptor value can be calculated by Equation (3) [33].

(3) DPSA2=PPSA2−PNSA2

PPSA2 and PNSA2 stand for the total weighted partial charge of the positively and negatively charged surface areas, respectively.

The highest occupied molecular orbital (HOMO) energy is also a semi-empirical descriptor with a negative coefficient value (−0.426925). Due to the negative mathematical value of the descriptor energy, the synthesized agent with high mathematical descriptor value turns a potent antimicrobial property and was revealed by compounds **5d** and **7a** (descriptor value = −8.532, −9.02 corresponding to an estimated MIC = 6.212, 19.693 μM for compounds **3e** and **5d**, respectively). The descriptor value can be calculated by Equation (4) [33].

(4) $$\varepsilon_{\mathrm{HOMO}}=\langle \phi_{\mathrm{HOMO}}|\hat{F}|\phi_{\mathrm{HOMO}}\rangle$$

since $\hat{F}$ stands for the Fock operator.

The maximum e-e repulsion for bond C-N is also a semi-empirical descriptor with a negative coefficient value (−0.475568). This is why the synthesized agent with a high mathematical descriptor value optimizes a potent antimicrobial active agent, as shown in compounds **3d** and **5e** (descriptor value = 165.9557, 166.9239 corresponding to an estimated MIC = 84.064, 9.498 μM for compounds **3d** and **5e**, respectively). The descriptor value can be calculated by Equation (5) [33].

(5) $$E_{\mathrm{ee}}\left(A\right)=\sum_{B\neq A}\sum_{\mu ,\nu \epsilon A}\sum_{\lambda ,\sigma \epsilon B}P_{\mu \nu }P_{\lambda \sigma }\left\langle \mu \nu |\lambda \sigma \right\rangle$$

since A and B are two different atomic species. The P_μν_, P_λσ_ are the density matrix elements over the atomic basis {μνλσ}.

The ⟨μν│λσ⟩ are the electron repulsion integrals on the atomic basis {μνλσ}.

#### 2.3.3. *B. Subtilis*

The three-descriptor model describes the broad-range antimicrobial properties of the synthesis agents against *B. subtilis* (observed log (MIC, μM) = 0.352–1.905, predicted log (MIC, μM) = 0.47–1.99, i.e., observed (MIC, μM) = 2.25–80.323, predicted (MIC, μM) = 2.953–97.751). The coefficient values support the QSAR goodness (*R*^2^ = 0.935, *R*^2^cvOO = 0.915, *R*^2^cvMO = 0.916) (Supplementary Tables S4–S6, Figure S3). The semi-empirical descriptor and minimum total interaction for the H-C (*t* = −3.867) bond appeared with a negative sign in the QSAR model (coefficient = −2.09499). This explains the high estimated antimicrobial properties of the compounds with a high mathematical descriptor value, as revealed in compounds **3d** and **5b** (descriptor value = 11.8739, 12.0794, corresponding to an estimated MIC = 97.751, 2.953 μM for compounds **3d** and **5b**, respectively). Equation (6) can calculate the total interaction energy of two atoms [33].

(6) $$E_{\mathrm{tot}}\left(\mathrm{AB}\right)={\;E}_{c}\left(\mathrm{AB}\right)+{\;E}_{\mathrm{exc}}\left(\mathrm{AB}\right)$$

where A and B are the two different atoms. The E_C_ (AB) is electrostatic interaction energy between the two atomic species A and B. The E_exc_ (AB) is electronic exchange energy between the two atomic species A and B.

The minimum total interaction for bond C-C is also a semi-empirical descriptor with a negative coefficient value (−2.13915). Again, the higher the mathematical descriptor value, the higher potency of the constructed agent, as shown in compounds **3d** and **5b** (descriptor value = 13.2499, 13.6499 corresponding to an estimated MIC = 97.751, 2.953 μM for compounds **3d** and **5b**, respectively). Equation (6) can also calculate the descriptor value [33].

The semi-empirical descriptor maximum e-e repulsion for N also appeared with a negative sign in the QSAR model. Similar to the aforementioned, the high antimicrobial properties of **3d** over **5b** can be justified (descriptor value = 143.4363, 144.2946 corresponding to estimated MIC = 97.751, 2.953 μM for compounds **3d** and **5b**, respectively). Equation (7) can calculate the electron–electron repulsion energy of an atom [33].

(7) $$E_{\mathrm{ee}}\left(A\right)=\sum_{B\neq A}\sum_{\mu ,\nu \epsilon A}\sum_{\lambda ,\sigma \epsilon B}P_{\mu \nu }P_{\lambda \sigma }\left\langle \mu \nu |\lambda \sigma \right\rangle$$

since A and B are two different atomic species. The P_μν_, P_λσ_ are the density matrix elements over the atomic basis {μνλσ}.

The ⟨μν│λσ⟩ are the electron repulsion integrals on the atomic basis {μνλσ}.

The comparable predicted antimicrobial properties relative to the observed, especially for the potent analogs, is a good indication for the accuracy of the QSAR models. The statistical parameters, including the Fisher criteria (*F*) and standard deviation (*s*), are also good indications for the QSAR goodness (*F* = 81.973, 129.818; *s*^2^ = 0.010, 0.024 for the *S. aureus* and *B. subtilis* models, respectively). The comparable values of leave-one-out and leave-many-out modifications to the QSAR model coefficient value are also good evidence for the accuracy of the models (*R*^2^ = 0.926, 0.935; *R*^2^cvOO = 0.898, 0.915; *R*^2^cvMO = 0.903, 0.916 for the *S. aureus* and *B. subtilis* models, respectively).

### 2.4. Pharmacophoric Modeling

Pharmacophoric modeling is an accessible technique in medicinal chemistry that is usually expressed in various steric or electrostatic features (positive/negative ionizable, hydrogen bonding donor/acceptor, and hydrophobic) [34,35].

#### 2.4.1. *S. Aureus*

Three chemical feature models were observed for the 3D-pharmacophoric model of the synthesized agents with variable antimicrobial properties against *S. aureus*, including a hydrogen-bonding donor and two hydrophobics (Supplementary Table S7, Figures S4 and S5). It is noticeable that the estimated antimicrobial properties were comparable with the observed properties, preserving the potency, especially for the highly effective agents discovered. It has also been noticed that all the synthesized agents (compound **5e** is an exception) show the alignment of the morpholinyl and phenyl group attached to the oxazolidinyl *N* with the hydrophobics. However, compound **5e** shows an alignment of the methylphenyl group with the hydrophobic, while the hydrogen bonding acceptor function is aligned with the exocyclic amidic carbonyl. It is also noticeable that all the potent, and most of the mild, antimicrobial agents synthesized reveal the alignment of the oxazolidinyl carbonyl with the hydrogen-bonding acceptor function. However, the low antimicrobial agents show the alignment of the hydrogen-bonding function with the exocyclic amidic carbonyl. In other words, the fitness of the proper group in the hydrogen bonding acceptor function can predicate the potency of the constructed agent. This supports the aforementioned SAR describing the role of phenyl substituted with the electron-withdrawing group and the π-deficient heterocycles in enhancing the antimicrobial properties observed. This is rationalized due to the electronic effect transfer giving rise to better accessibility to the oxazolidinyl carbonyl for interacting/alignment with the pharmacophoric hydrogen-bonding acceptor function.

#### 2.4.2. *B. Subtilis*

Four chemical features were observed by the pharmacophoric model of the antimicrobial agents against *B. subtilis*, comprising three hydrogen-bonding acceptors and a hydrophobic. All the tested agents show the alignment of the morpholinyl oxygen and oxazolidinyl carbonyl with hydrogen-bonding acceptors. Additionally, the phenyl group attached to the oxazolidinyl *N* is aligned with the hydrophobic. These observations are similar to what was revealed in the pharmacophoric model by most of the tested agents against *S. aureus*. It is noticeable that the hydrogen bonding acceptor (HBA-1) is aligned with the group linked to the oxazolindinyl C-5. Variation of this group, due to the diversity in chemical function utilized, led to variable fitness and, consequently, variability in estimated bio-properties. This, again, can support the aforementioned SAR (Supplementary Table S8, Figures S6 and S7).

#### 2.4.3. ADMET

Computational ADMET (absorption, distribution, metabolism, excretion, and toxicity) “Discovery Studio 2.5 software” [36] exhibits that the aqueous solubility of the synthesized agents ranged from good to low level (aqueous solubility level: 2–3). This is due to the heterocyclic scaffold of the synthesized agents. Most of the synthesized agents revealed good intestinal absorption (intestinal absorption level = 0). Additionally, all the synthesized conjugates **5a–f** and **7a–c**, show promising antimicrobial properties that exhibit high plasma protein binding (PPB level = 2). Compounds **11a–f** also show a high PPB level. All the synthesized conjugates were non-hepatotoxic agents. The ADMET descriptor values seemed promising, especially for the potent agents discovered, and can be considered for future studies targeting the development of promising hits/leads (Table 2).

## 3. Materials and Methods

### 3.1. Chemistry

Melting points were determined on a capillary point apparatus equipped with a digital thermometer and were uncorrected. NMR spectra were recorded in CDCl_3_ or DMSO-*d_6_* on a Bruker spectrometer operating at 500 MHz for ^1^H (with TMS as an internal standard) and 125 MHz for ^13^C using the NMR facility at the Department of Chemistry and Physics, Faculty of Science and Mathematics, Augusta University, Augusta, GA, USA. IR spectra (KBr, cm^−1^) were recorded on a Thermo Fisher Scientific, Waltham, MA, USA (Nicolet iS5) spectrophotometer at the Department of Chemistry and Physics, Faculty of Science and Mathematics, Augusta University, Augusta, GA, USA. HRMS were measured using Agilent Technologies 6545 Q-TOF LC/MS. TLC was performed on precoated silica gel (Merck 60 F254); spots were visualized by iodine vapors or irradiation with UV light (254 nm).

#### 3.1.1. General Method for Preparation of Compounds **3a–k**, **5a–f**, **7a–c**, and **9a–c**

A round bottom flask (50 mL) containing a small stir bar was charged with a series of benzotriazole-activated acids (**2a–k, 4a–f, 6a–c,** and **8a–c**) (1.1 eq.) and 5-(aminomethyl)-3-(3-fluoro-4-morpholinophenyl)oxazolidin-2-one (**1**) (500 mg, 1.0 eq.) dissolved in DCM (15 mL), along with TEA (1.5 eq.). The reaction mixture was stirred starting at 0 °C and continuing until at room temperature; the progress of the reaction was monitored by TLC. After completion of the reaction, the solvent was evaporated under reduced pressure and the residue was treated three times with 10 mL 20% Na_2_CO_3_ cold solution. The precipitate formed was filtered out, washed with water, and dried under vacuum to get the desired products.

#### 3.1.2. Benzyl (S)-(2-(((3-(3-fluoro-4-morpholinophenyl)-2-oxooxazolidin-5-yl)methyl) amino)-2-oxoethyl)carbamate (**3a**)

White solid, mp: 153–155 °C, yield: 63% (0.57 g). IR: *ν*_max_/cm^−1^; 3336 (NH), 3296 (NH), 3065 (CH, aromatic), 2931, 2846 (CH, aliphatic), 1728, 1697, 1672 (C = O), 1517 (C = C), 1291 (C-N), 1238 (C-O), 1110 (C-F); ^1^H NMR (CDCl_3_) δ: 7.46 (s, 1H, NH), 7.43 (s, 1H, NH), 7.33–7.30 (m, 5H, Ar–H), 7.05 (s, 2H, Ar–H), 6.78 (s, 1H, Ar–H), 5.37 (s, 1H, CH), 5.05 (s, 2H, CH_2_), 4.72 (s, 1H, CH_2_), 3.96 (s, 1H, CH_2_), 3.87 [s, 5H, (1H, CH_2_) + (4H, 2CH_2_)], 3.72 (s, 1H, CH_2_), 3.65 (s, 2H, CH_2_), 3.06 (s, 4H, 2CH_2_); ^13^C NMR (CDCl_3_) δ: 170.4, 156.9, 154.7, 154.5, 136.1, 128.8, 128.5, 128.4, 114.2, 107.8, 71.9, 67.6, 66.7, 51.5, 47.7, 44.8, 41.9, 31.2; HRMS: *m*/*z* for C_24_H_27_FN_4_O_6_ [M+Na]^+^ Calcd.: 509.1807, Found: 509.1807.

#### 3.1.3. Benzyl ((S)-1-((((S)-3-(3-fluoro-4-morpholinophenyl)-2-oxooxazolidin-5-yl)methyl) amino)-1-oxopropan-2-yl)carbamate (**3b**)

White solid, mp: 186–188 °C, yield: 51% (0.48 g). IR: *ν*_max_/cm^−1^; 3319 (NH), 3040, 3001 (CH, aromatic), 2928, 2845 (CH, aliphatic), 1726, 1690 (C = O), 1518 (C = C), 1254 (C-N), 1238 (C-O), 1110 (C-F); ^1^H NMR (CDCl_3_) δ: 7.50 (s, 1H, NH), 7.47 (s, 1H, NH), 7.32–7.29 (m, 5H, Ar–H), 7.06 (s, 2H, Ar–H), 6.78 (s, 1H, Ar–H), 5.12 (s, 1H, CH), 5.05 (d, *J* = 11.1 Hz, 1H, CH_2_), 4.94 (d, *J* = 11.0 Hz, 1H, CH_2_), 4.72 (s, 1H, CH), 4.17 (s, 1H, CH_2_), 3.90 (s, 4H, 2CH_2_), 3.77 (s, 1H, CH_2_), 3.72–3.70 (m, 1H, CH_2_), 3.61–3.58 (m, 1H, CH_2_), 3.08 (s, 4H, 2CH_2_), 1.32 (s, 3H, CH_3_); ^13^C NMR (CDCl_3_) δ: 173.8, 156.7, 154.4, 136.1, 128.8, 128.5, 128.4, 114.2, 108.0, 107.8, 72.0, 67.5, 66.5, 51.7, 51.0, 47.7, 41.8, 18.2; HRMS: *m*/*z* for C_25_H_29_FN_4_O_6_ [M+H]^+^ Calcd.: 501.2144, Found: 501.2132.

#### 3.1.4. Benzyl ((S)-1-((((S)-3-(3-fluoro-4-morpholinophenyl)-2-oxooxazolidin-5-yl)methyl) amino)-3-methyl-1-oxobutan-2-yl)carbamate (**3c**)

White solid, mp: 173–175 °C, yield: 60% (0.59 g). IR: *ν*_max_/cm^−1^; 3298 (NH), 3058 (CH, aromatic), 2958, 2854 (CH, aliphatic), 1744, 1692 (C = O), 1518 (C = C), 1257 (C-N), 1240 (C-O), 1115 (C-F); ^1^H NMR (CDCl_3_) δ: 7.51 (s, 1H, NH), 7.48 (s, 1H, NH), 7.32–7.30 (m, 5H, Ar–H), 7.17 (s, 1H, Ar–H), 7.05 (s, 1H, Ar–H), 6.60 (s, 1H, Ar–H), 5.20 (s, 1H, CH), 5.04 (d, *J* = 11.6 Hz, 1H, CH_2_), 4.95 (d, *J* = 11.8 Hz, 1H, CH_2_), 4.71 (s, 1H, CH), 3.95 (s, 1H, CH_2_), 3.91 (s, 4H, 2CH_2_), 3.74 (s, 1H, CH_2_), 3.65 (s, 2H, CH_2_), 3.10 (s, 4H, 2CH_2_), 2.15 (s, 1H, CH), 0.93 (s, 3H, CH_3_) 0.84 (s, 3H, CH_3_); ^13^C NMR (CDCl_3_) δ: 186.8, 157.2, 156.7, 145.1, 128.9, 128.8, 128.5, 128.3, 120.7, 114.2, 72.0, 67.4, 66.4, 61.0, 51.8, 51.7, 47.7, 42.0, 30.7, 19.5; HRMS: *m*/*z* for C_27_H_33_FN_4_O_6_ [M+Na]^+^ Calcd.: 551.2276, Found: 551.2284.

#### 3.1.5. Benzyl ((S)-1-((((S)-3-(3-fluoro-4-morpholinophenyl)-2-oxooxazolidin-5-yl)methyl) amino)-4-(methylthio)-1-oxobutan-2-yl)carbamate (**3d**)

White solid, mp: 165–167 °C, yield: 84% (0.88 g). IR: *ν*_max_/cm^−1^; 3271 (NH), 3020 (CH, aromatic), 2915, 2850 (CH, aliphatic), 1744, 1706 (C = O), 1516 (C = C), 1258 (C-N), 1226 (C-O), 1118 (C-F); ^1^H NMR (CDCl_3_) δ: 7.52 (s, 1H, NH), 7.49 (s, 1H, NH), 7.31–7.29 (m, 5H, Ar–H), 7.18 (s, 1H, Ar–H), 7.05 (s, 1H, Ar–H), 6.97 (s, 1H, Ar–H), 5.45 (s, 1H, CH), 5.04 (d, *J* = 11.4 Hz, 1H, CH_2_), 4.94 (d, *J* = 11.6 Hz, 1H, CH_2_), 4.72 (s, 1H, CH), 4.30 (s, 1H, CH_2_), 3.91 [s, 5H, (1H, CH_2_) + (4H, 2CH_2_)], 3.76–3.68 (m, 1H, CH_2_), 3.61–3.59 (m, 1H, CH_2_), 3.10 (s, 4H, 2CH_2_), 2.49 (s, 2H, CH_2_), 2.03 (s, 3H, CH_3_), 1.89 (s, 2H, CH_2_); ^13^C NMR (CDCl_3_) δ: 172.7, 156.8, 154.4, 136.1, 128.8, 128.5, 128.3, 114.1, 108.0, 107.8, 79.9, 71.9, 67.5, 66.4, 54.3, 51.8, 47.7, 41.9, 30.4, 15.5; HRMS: *m*/*z* for C_27_H_33_FN_4_O_6_S [M+Na]^+^ Calcd.: 583.1997, Found: 583.1994.

#### 3.1.6. Benzyl ((S)-1-((((S)-3-(3-fluoro-4-morpholinophenyl)-2-oxooxazolidin-5-yl)methyl) amino)-1-oxo-3-phenylpropan-2-yl)carbamate (**3e**)

White solid, mp: 120–122 °C, yield: 85% (0.91 g). IR: *ν*_max_/cm^−1^; 3308 (NH), 3025 (CH, aromatic), 2951, 2923, 2854 (CH, aliphatic), 1739, 1687 (C = O), 1515 (C = C), 1285 (C-N), 1237 (C-O), 1121 (C-F); ^1^H NMR (DMSO-*d_6_*) δ: 8.44 (s, 1H, NH), 7.91 (s, 1H, NH), 7.54–7.46 (m, 2H, Ar–H), 7.31–7.04 (m, 10H, Ar–H), 7.03 (s, 1H, Ar–H), 4.91–4.84 (m, 2H, CH_2_), 4.72 (s, 1H, CH), 4.23 (s, 1H, CH), 4.03 (s, 1H, CH_2_), 3.72 (s, 5H, (1H, CH_2_) + (4H, 2CH_2_)), 3.60 (s, 1H, CH_2_), 3.47 (s, 1H, CH_2_), 2.92 (s, 4H, 2CH_2_), 2.89 (s, 1H, CH_2_), 2.77–2.72 (m, 1H, CH_2_); ^13^C NMR (DMSO-*d_6_*) δ: 172.5, 155.8, 155.5, 154.0, 138.0, 136.9, 135.4, 133.4, 129.1, 128.2, 128.0, 127.4, 126.2, 119.1, 114.2, 106.9, 78.9, 71.5, 67.0, 66.1, 65.1, 56.3, 50.7, 47.0, 41.0, 37.4; HRMS: *m*/*z* for C_31_H_33_FN_4_O_6_ [M+H]^+^ Calcd.: 577.2457, Found: 577.2445.

#### 3.1.7. Tert-butyl (S)-(2-(((3-(3-fluoro-4-morpholinophenyl)-2-oxooxazolidin-5-yl)methyl) amino)-2-oxoethyl)carbamate (**3f**)

Off white solid, mp: 54–56 °C, yield: 92% (0.70 g). IR: *ν*_max_/cm^−1^; 3305 (NH), 3016 (CH, aromatic), 2968, 2875 (CH, aliphatic), 1748, 1681 (C = O), 1515 (C = C), 1218 (C-N), 1116 (C-O), 1049 (C-F); ^1^H NMR (DMSO-*d_6_*) δ: 8.18 (s, 1H, NH), 7.52–7.43 (m, 1H, Ar–H), 7.17 (d, *J* = 7.5 Hz, 1H, Ar–H), 7.04 (s, 1H, NH), 6.97 (s, 1H, Ar–H), 4.71 (s, 1H, CH), 4.05–4.03 (m, 1H, CH_2_), 3.73 (s, 5H, (1H, CH_2_) + (4H, 2CH_2_)), 3.54 (s, 2H, CH_2_), 3.44 (s, 2H, CH_2_), 2.95 (s, 4H, 2CH_2_), 1.36 (s, 9H, 3CH_3_); ^13^C NMR (DMSO-*d_6_*) δ: 170.2, 155.8, 154.0, 153.6, 135.6, 133.5, 119.2, 114.2, 106.6, 78.0, 71.4, 66.1, 50.7, 47.2, 43.2, 41.1, 28.2; HRMS: *m*/*z* for C_21_H_29_FN_4_O_6_ [M+H]^+^ Calcd.: 453.2144, Found: 453.2146.

#### 3.1.8. Tert-butyl ((S)-1-((((S)-3-(3-fluoro-4-morpholinophenyl)-2-oxooxazolidin-5-yl) methyl) amino)-1-oxopropan-2-yl)carbamate (**3g**)

Yellowish white solid, mp: 71–72 °C, yield: 78% (0.62 g). IR: *ν*_max_/cm^−1^; 3306 (NH), 3050 (CH, aromatic), 2972, 2857 (CH, aliphatic), 1737, 1673 (C = O), 1514 (C = C), 1227 (C-N), 1168 (C-O), 1114 (C-F); ^1^H NMR (DMSO-*d_6_*) δ: 8.18 (s, 1H, NH), 7.53–7.44 (m, 1H, Ar–H), 7.21–7.14 (m, 1H, Ar–H), 7.05 (s, 1H, NH), 6.92 (s, 1H, Ar–H), 4.72 (s, 1H, CH), 4.04–4.03 (m, 1H, CH), 3.92–3.82 (m, 1H, CH_2_), 3.73 [s, 5H, (1H, CH_2_) + (4H, 2CH_2_)], 2.95 [s, 5H, (1H, CH_2_) + (4H, 2CH_2_)], 2.86–2.77 (m, 1H, CH_2_), 1.34 (s, 9H, 3CH_3_), 1.18–1.07 (m, 3H, CH_3_); ^13^C NMR (DMSO-*d_6_*) δ: 173.9, 155.0, 154.4, 154.1, 135.5, 133.4, 119.2, 113.9, 106.6, 77.9, 71.6, 66.1, 64.9, 50.7, 49.8, 47.0, 44.1, 28.2, 15.2; HRMS: *m*/*z* for C_22_H_31_FN_4_O_6_ [M+H]^+^ Calcd.: 467.2300, Found: 467.2329.

#### 3.1.9. Tert-butyl ((S)-1-((((S)-3-(3-fluoro-4-morpholinophenyl)-2-oxooxazolidin-5-yl) methyl) amino)-3-methyl-1-oxobutan-2-yl)carbamate (**3h**)

Off white solid, mp: 61–63 °C, yield: 87% (0.73 g). IR: *ν*_max_/cm^−1^; 3306 (NH), 3045 (CH, aromatic), 2965 (CH, aliphatic), 1747 (C = O), 1514 (C = C), 1224 (C-N), 1167 (C-O), 1115 (C-F); ^1^H NMR (DMSO-*d_6_*) δ: 8.25 (s, 1H, NH), 7.52–7.44 (m, 1H, Ar–H), 7.15 (s, 1H, NH), 7.04 (s, 1H, Ar–H), 6.70 (d, *J* = 5.1 Hz, 1H, Ar–H), 4.73 (s, 1H, CH), 4.05–4.03 (m, 1H, CH), 3.73 (s, 6H, 3CH_2_), 3.51–3.45 (m, 2H, CH_2_), 2.95 (s, 4H, 2CH_2_), 1.87 (s, 1H, CH), 1.34 (s, 9H, 3CH_3_), 0.81 (s, 6H, 2CH_3_); ^13^C NMR (DMSO-*d_6_*) δ: 172.3, 155.4, 154.0, 153.6, 135.5, 133.5, 125.2, 119.2, 114.2, 106.6, 77.9, 71.5, 66.1, 59.9, 50.7, 46.9, 40.9, 30.2, 28.1, 19.2, 18.3; HRMS: *m*/*z* for C_24_H_35_FN_4_O_6_ [M+H]^+^ Calcd.: 495.2613, Found: 495.2607.

#### 3.1.10. Tert-butyl ((S)-1-((((S)-3-(3-fluoro-4-morpholinophenyl)-2-oxooxazolidin-5-yl) methyl) amino)-4-(methyl thio)-1-oxobutan-2-yl)carbamate (**3i**)

White solid, mp: 56–58 °C, yield: 64% (0.57 g). IR: *ν*_max_/cm^−1^; 3304 (NH), 3086 (CH, aromatic), 2970, 2856 (CH, aliphatic), 1745, 1708 (C = O), 1514 (C = C), 1225 (C-N), 1166 (C-O), 1114 (C-F); ^1^H NMR (DMSO-*d_6_*) δ: 8.23 (s, 1H, NH), 7.52–7.43 (m, 1H, Ar–H), 7.21–7.13 (m, 1H, Ar–H), 7.06–6.98 (m, 2H, NH + Ar–H), 4.73 (s, 1H, CH), 4.08–4.02 (m, 1H, CH), 3.96 (s, 1H, CH_2_), 3.73 [s, 5H, (1H, CH_2_) + (4H, 2CH_2_)], 2.95 [s, 5H, (1H, CH_2_) + (4H, 2CH_2_)], 2.85–2.77 (m, 1H, CH_2_), 2.42–2.36 (m, 2H, CH_2_), 2.04–1.95 (m, 3H, CH_3_), 1.74–1.64 (m, 2H, CH_2_), 1.35 (s, 9H, 3CH_3_); ^13^C NMR (DMSO-*d_6_*) δ: 176.0, 155.4, 154.1, 153.6, 135.4, 133.4, 119.2, 113.9, 106.7, 78.0, 74.0, 71.5, 66.2, 53.8, 50.7, 47.1, 44.2, 29.7, 28.8, 28.1, 14.5; HRMS: *m*/*z* for C_24_H_35_FN_4_O_6_S [M+H]^+^ Calcd.: 527.2334, Found: 527.2373.

#### 3.1.11. Tert-butyl ((S)-1-((((S)-3-(3-fluoro-4-morpholinophenyl)-2-oxooxazolidin-5-yl) methyl) amino)-1-oxo-3-phenylpropan-2-yl)carbamate (**3j**)

Off white solid, mp: 69–71 °C, yield: 74% (0.75 g). IR: *ν*_max_/cm^−1^; 3294 (NH), 3040 (CH, aromatic), 2975 (CH, aliphatic), 1748 (C = O), 1514 (C = C), 1225 (C-N), 1166 (C-O), 1115 (C-F); ^1^H NMR (CDCl_3_) δ: 7.94 (s, 1H, NH), 7.49–7.28 (m, 6H, Ar–H), 7.16 (s, 1H, Ar–H), 6.97 (s, 1H, Ar–H), 5.43 (s, 1H, CH), 4.77–4.71 (m, 1H, CH), 4.54 (s, 1H, CH_2_), 3.99 (s, 1H, CH_2_), 3.93 (s, 4H, 2CH_2_), 3.68 (s, 2H, CH_2_), 3.15 (s, 1H, CH_2_), 3.10 (s, 4H, 2CH_2_), 3.02 (s, 1H, CH_2_), 1.43 (s, 9H, 3CH_3_); ^13^C NMR (CDCl_3_) δ: 173.0, 156.4, 155.5, 154.5, 136.6, 136.4, 133.1, 129.3, 128.6, 127.0, 118.9, 114.2, 114.0, 107.6, 107.4, 80.1, 72.0, 71.8, 67.0, 60.5, 55.9, 51.0, 47.7, 41.7, 38.7, 28.3; HRMS: *m*/*z* for C_28_H_35_FN_4_O_6_ [M+Na]^+^ Calcd.: 565.2433, Found: 565.2439.

#### 3.1.12. Tert-butyl ((2S,3S)-1-((((S)-3-(3-fluoro-4-morpholinophenyl)-2-oxooxazolidin-5-yl) methyl)amino)-3-methyl-1-oxopentan-2-yl)carbamate (**3k**)

White solid, mp: 62–64 °C, yield: 77% (0.67 g). IR: *ν*_max_/cm^−1^; 3306 (NH), 3075 (CH, aromatic), 2966 (CH, aliphatic), 1747 (C = O), 1514 (C = C), 1225 (C-N), 1168 (C-O), 1115 (C-F); ^1^H NMR (DMSO-*d_6_*) δ: 8.23 (s, 1H, NH), 7.53–7.45 (m, 1H, Ar–H), 7.22–7.15 (m, 1H, Ar–H), 7.05 (s, 1H, NH), 6.84–6.75 (m, 1H, Ar–H), 4.73 (s, 1H, CH), 4.07–4.03 (m, 1H, CH), 3.78 (s, 1H, CH_2_), 3.73 [s, 5H, (1H, CH_2_) + (4H, 2CH_2_)], 3.45–3.37 (m, 2H, CH_2_), 2.95 (s, 4H, 2CH_2_),1.62 (s, 1H, CH), 1.33 (s, 9H, 3CH_3_), 1.09–1.04 (m, 2H, CH_2_), 0.75 (s, 6H, 2CH_3_); ^13^C NMR (DMSO-*d_6_*) δ: 172.3, 155.5, 155.3, 154.0, 135.5, 133.5, 119.2, 114.2, 106.6, 77.9, 71.5, 66.1, 58.9, 50.7, 46.9, 44.1, 36.2, 28.1, 24.5, 15.3, 10.9; HRMS: *m*/*z* for C_25_H_37_FN_4_O_6_ [M+H]^+^ Calcd.: 509.2770, Found: 509.2800.

#### 3.1.13. General Method for Synthesis of Compounds **4a–f**

1*H*-Benzotriazole (4.0 eq.) was dissolved in anhydrous THF. Thionyl chloride (1.5 eq.) was added and the mixture was stirred for 30 min. The corresponding acid (1.0 eq.) was then added and the reaction mixture was stirred for another 2–3 h at room temperature. The reaction was monitored by TLC and, upon its completion, the solvent was evaporated, a few crystals of ice were added and the formed residue was then washed 3 times with 15 mL 20% Na_2_CO_3_ solution. After filtration, the collected precipitate was crystallized from diethyl ether to yield **4e** and **4f** in pure form [22].

#### 3.1.14. (1H-Benzo[d][1,2,3] triazol-1-yl)(4-(methylamino)phenyl)methanone (**4e**)

Shiny yellow microcrystals, mp: 179–181 °C, yield: 85% (0.71 g). IR: *ν*_max_/cm^−1^; 3364 (NH streching), 3001 (CH, aromatic), 2875, 2783 (CH, aliphatic), 1680 (C = O), 1595 (NH bending), 1530 (C = C), 1286 (C-N); ^1^H NMR (CDCl_3_) δ: 8.32 (d, *J* = 8.3 Hz, 1H, Ar–H), 8.22–8.19 (m, 2H, Ar–H), 8.12 (d, *J* = 8.3 Hz, 1H, Ar–H), 7.63 (t, *J* = 8.1 Hz, 1H, Ar–H), 7.48 (t, *J* = 8.1 Hz, 1H, Ar–H), 6.70–6.67 (m, 2H, Ar–H), 2.94 (s, 3H, CH_3_); ^13^C NMR (CDCl_3_) δ: 165.6, 153.7, 145.8, 134.9, 133.0, 130.0, 126.0, 120.1, 115.0, 111.8, 30.5; HRMS: *m*/*z* for C_14_H_12_N_4_O [M^+^] Calcd.: 252.1011, Found: 252.1019.

#### 3.1.15. (1H-Benzo[d][1,2,3] triazol-1-yl)(2-chloro-3-nitrophenyl)methanone (**4f**)

White solid, mp: 135–137 °C, yield: 73% (0.55 g). IR: *ν*_max_/cm^−1^; 3029 (CH, aromatic), 1723 (C = O), 1533 (C = C), 1290 (C-N), 748 (C-Cl); ^1^H NMR (CDCl_3_) δ: 8.39 (d, *J* = 8.3 Hz, 1H, Ar–H), 8.16 (d, *J* = 8.3 Hz, 1H, Ar–H), 8.06 (dd, *J* = 8.2, 1.6 Hz, 1H, Ar–H), 7.81 (dd, *J* = 7.7, 1.6 Hz, 1H, Ar–H), 7.78–7.74 (m, 1H, Ar–H), 7.64–7.58 (m, 2H, Ar–H); ^13^C NMR (CDCl_3_) δ: 164.1, 148.9, 146.6, 136.3, 133.0, 131.4, 131.2, 128.0, 127.8, 127.4, 125.6, 120.9, 114.6; HRMS: *m*/*z* for C_13_H_7_ClN_4_O_3_ [M+H]^+^ Calcd.: 303.0279, Found: 303.0281.

#### 3.1.16. (S)-N-((3-(3-Fluoro-4-morpholinophenyl)-2-oxooxazolidin-5-yl)methyl)benzamide (**5a**)

White microcrystals, mp: 174–176 °C, yield: 98% (0.66 g). IR: *ν*_max_/cm^−1^; 3280 (NH), 3062 (CH, aromatic), 2854 (CH, aliphatic), 1720 (C = O), 1517 (C = C), 1223 (C-N), 1193 (C-O), 1117 (C-F); ^1^H NMR (CDCl_3_) δ: 7.75–7.73 (m, 2H, Ar–H), 7.52–7.40 (m, 4H, Ar–H), 7.04 (s, 1H, Ar–H), 7.71 (t, *J* = 6.1 Hz, 1H, Ar–H), 4.89–4.84 (m, 1H, CH), 4.06 (t, *J* = 9.0 Hz, 1H, CH_2_), 3.94–3.90 (m, 1H, CH_2_), 3.88 (t, *J* = 4.3 Hz, 4H, 2CH_2_) =, 3.84–3.77 (m, 2H, CH_2_), 3.07 (t, *J* = 4.4 Hz, 4H, 2CH_2_); ^13^C NMR (CDCl_3_) δ: 168.5, 156.7, 154.8, 154.5, 133.7, 132.2, 128.9, 127.3, 114.2, 108.0, 107.7, 72.3, 66.7, 51.6, 48.0, 42.7; HRMS: *m*/*z* for C_21_H_22_FN_3_O_4_ [M+H]^+^ Calcd.: 400.1667, Found: 400.1679.

#### 3.1.17. (S)-4-Fluoro-N-((3-(3-fluoro-4-morpholinophenyl)-2-oxooxazolidin-5-yl)methyl) benzamide (**5b**)

White microcrystals, mp: 173–175 °C, yield: 96% (0.68 g). IR: *ν*_max_/cm^−1^; 3363 (NH), 3058 (CH, aromatic), 2932, 2867 (CH, aliphatic), 1745 (C = O), 1503 (C = C), 1220 (C-N), 1196 (C-O), 1120 (C-F); ^1^H NMR (DMSO-*d_6_*) δ: 8.84 (t, *J* = 5.7 Hz, 1H, Ar–H), 7.93–7.90 (m, 2H, NH + Ar–H), 7.47 (dd, *J* = 15.0, 2.6 Hz, 1H, Ar–H), 7.32–7.28 (m, 2H, Ar–H), 7.18 (dd, *J* = 8.8, 2.2 Hz, 1H, Ar–H), 7.05 (t, *J* = 9.2 Hz, 1H, Ar–H), 4.87–4.82 (m, 1H, CH), 4.14 (t, *J* = 9.0 Hz, 1H, CH_2_), 3.83 (q, *J* = 6.0 Hz, 1H, CH_2_), 3.73 (t, *J* = 4.5 Hz, 4H, 2CH_2_), 3.67–3.57 (m, 2H, CH_2_), 2.95 (t, *J* = 4.6 Hz, 4H, 2CH_2_); ^13^C NMR (DMSO-*d_6_*) δ: 165.9, 164.9, 162.9, 155.5, 154.1, 153.6, 135.5, 133.4, 130.5, 130.0, 119.2, 115.3, 114.1, 106.7, 71.3, 66.1, 50.7, 47.5, 42.3; HRMS: *m*/*z* for C_21_H_21_F_2_N_3_O_4_ [M+Na]^+^ Calcd.: 440.1392, Found: 440.1405.

#### 3.1.18. (S)-4-Chloro-N-((3-(3-fluoro-4-morpholinophenyl)-2-oxooxazolidin-5-yl)methyl) benzamide (**5c**)

Off white microcrystals, mp: 213–215 °C, yield: 30% (0.22 g). IR: *ν*_max_/cm^−1^; 3349 (NH), 3085 (CH, aromatic), 2955 (CH, aliphatic), 1732 (C = O), 1515 (C = C), 1226 (C-N), 1197 (C-O), 1112 (C-F), 757 (C-Cl); ^1^H NMR (DMSO-*d_6_*) δ: 8.91 (t, *J* = 5.7 Hz, 1H, Ar–H), 7.88–7.85 (m, 2H, NH + Ar–H), 7.57–7.54 (m, 2H, Ar–H), 7.48 (dd, *J* = 15.0, 2.5 Hz, 1H, Ar–H), 7.19 (dd, *J* = 8.8, 2.1 Hz, 1H, Ar–H), 7.06 (t, *J* = 9.1 Hz, 1H, Ar–H), 4.88–4.83 (m, 1H, CH), 4.15 (t, *J* = 9.0 Hz, 1H, CH_2_), 3.84 (q, *J* = 6.0 Hz, 1H, CH_2_), 3.74 (t, *J* = 4.5 Hz, 4H, 2CH_2_), 3.68–3.58 (m, 2H, CH_2_), 2.96 (t, *J* = 4.6 Hz, 4H, 2CH_2_); ^13^C NMR (DMSO-*d_6_*) δ: 166.0, 155.5, 154.1, 153.6, 136.3, 135.5, 133.5, 132.7, 129.2, 128.4, 119.2, 114.2, 106.8, 71.3, 66.2, 50.7, 47.6, 42.4; HRMS: *m*/*z* for C_21_H_21_ClFN_3_O_4_ [M+H]^+^ Calcd.: 434.1277, Found: 434.1285.

#### 3.1.19. (S)-N-((3-(3-Fluoro-4-morpholinophenyl)-2-oxooxazolidin-5-yl)methyl)-4-nitro benzamide (**5d**)

Yellow microcrystals, mp: 218–220 °C, yield: 97% (0.73 g). IR: *ν*_max_/cm^−1^; 3370 (NH), 3076 (CH, aromatic), 2850 (CH, aliphatic), 1734 (C = O), 1518 (C = C), 1237 (C-N), 1210 (C-O), 1113 (C-F); ^1^H NMR (DMSO-*d_6_*) δ: 9.16 (t, *J* = 5.7 Hz, 1H, Ar–H), 8.33–8.30 (m, 2H, NH + Ar–H), 8.08–8.05 (m, 2H, Ar–H), 7.47 (dd, *J* = 15.0, 2.5 Hz, 1H, Ar–H), 7.19 (dd, *J* = 8.8, 2.1 Hz, 1H, Ar–H), 7.05 (t, *J* = 9.2 Hz, 1H, Ar–H), 4.90–4.85 (m, 1H, CH), 4.16 (t, *J* = 9.0 Hz, 1H, CH_2_), 3.84 (q, *J* = 6.0 Hz, 1H, CH_2_), 3.73 (t, *J* = 4.5 Hz, 4H, 2CH_2_), 3.69–3.63 (m, 2H, CH_2_), 2.95 (t, *J* = 4.6 Hz, 4H, 2CH_2_); ^13^C NMR (DMSO-*d_6_*) δ: 165.4, 155.5, 154.0, 153.6, 149.1, 139.6, 135.6, 133.3, 128.8, 123.5, 119.2, 114.1, 106.8, 71.2, 66.1, 50.7, 47.5, 42.5; HRMS: *m*/*z* for C_21_H_21_FN_4_O_6_ [M+H]^+^ Calcd.: 445.1518, Found: 445.1523.

#### 3.1.20. (S)-N-((3-(3-Fluoro-4-morpholinophenyl)-2-oxooxazolidin-5-yl)methyl)-4-(methyl amino) benzamide (**5e**)

The crude product was purified through column chromatography using 1% MeOH in DCM to obtain shiny white crystals, **5e** in pure form, mp: 195–197 °C, yield: 40% (0.29 g). IR: *ν*_max_/cm^−1^; 3373 (NH), 3062 (CH, aromatic), 2918, 2812 (CH, aliphatic), 1738 (C = O), 1506 (C = C), 1226 (C-N), 1188 (C-O), 1119 (C-F); ^1^H NMR (DMSO-*d_6_*) δ: 8.36 (t, *J* = 5.8 Hz, 1H, Ar–H), 7.66 (s, 1H, NH), 7.64 (s, 1H, NH), 7.48 (dd, *J* = 15.0, 2.6 Hz, 1H, Ar–H), 7.18 (dd, *J* = 8.8, 2.1 Hz, 1H, Ar–H), 7.04 (t, *J* = 9.2 Hz, 1H, Ar–H), 6.53 (s, 1H, Ar–H), 6.51 (s, 1H, Ar–H), 6.22–6.19 (m, 1H, Ar–H), 4.84–4.79 (m, 1H, CH), 4.12 (t, *J* = 9.0 Hz, 1H, CH_2_), 3.83 (q, *J* = 6.0 Hz, 1H, CH_2_), 3.73 (t, *J* = 4.5 Hz, 4H, 2CH_2_), 3.62–3.51 (m, 2H, CH_2_), 2.95 (t, *J* = 4.6 Hz, 4H, 2CH_2_), 2.70 (d, *J* = 4.8 Hz, 3H, CH_3_); ^13^C NMR (DMSO-*d_6_*) δ: 166.9, 155.5, 154.1, 153.6, 152.4, 135.5, 133.5, 128.8, 120.3, 119.2, 114.1, 110.3, 106.7, 71.5, 66.1, 50.7, 47.5, 42.1, 29.3; HRMS: *m*/*z* for C_22_H_25_FN_4_O_4_ [M^+^] Calcd.: 428.1860, Found: 428.1864.

#### 3.1.21. (S)-2-Chloro-N-((3-(3-fluoro-4-morpholinophenyl)-2-oxooxazolidin-5-yl)methy-l)-3-nitrobenzamide (**5f**)

Fine yellow microcrystals, mp: 200–202 °C, yield: 91% (0.74 g). IR: *ν*_max_/cm^−1^; 3290 (NH), 3054 (CH, aromatic), 2938, 2856 (CH, aliphatic), 1720 (C = O), 1514 (C = C), 1239 (C-N), 1146 (C-O), 1115 (C-F), 716 (C-Cl); ^1^H NMR (DMSO-*d_6_*) δ: 9.09 (t, *J* = 5.8 Hz, 1H, Ar–H), 8.08 (dd, *J* = 7.9, 1.8 Hz, 1H, Ar–H), 7.68–7.62 (m, 2H, NH + Ar–H), 7.50 (dd, *J* = 15.0, 2.5 Hz, 1H, Ar–H), 7.19 (dd, *J* = 8.8, 2.2 Hz, 1H, Ar–H), 7.07 (t, *J* = 9.2 Hz, 1H, Ar–H), 4.90–4.85 (m, 1H, CH), 4.17 (t, *J* = 9.1 Hz, 1H, CH_2_), 3.84 (q, *J* = 6.4 Hz, 1H, CH_2_), 3.73 (t, *J* = 4.5 Hz, 4H, 2CH_2_), 3.69–3.62 (m, 2H, CH_2_), 2.96 (t, *J* = 4.6 Hz, 4H, 2CH_2_); ^13^C NMR (DMSO-*d_6_*) δ: 165.6, 155.5, 154.0, 148.5, 139.1, 135.5, 133.4, 131.9, 128.6, 125.5, 121.4, 119.3, 114.0, 106.6, 71.2, 66.1, 50.7, 47.2, 41.7; HRMS: *m*/*z* for C_21_H_20_ClFN_4_O_6_ [M^+^] Calcd.: 478.1055, Found: 478.1070.

#### 3.1.22. (S)-N-((3-(3-Fluoro-4-morpholinophenyl)-2-oxooxazolidin-5-yl)methyl)isonicotin-amide (**7a**)

White solid, mp: 198–200 °C, yield: 90% (0.61 g). IR: *ν*_max_/cm^−1^; 3303 (NH), 3031 (CH, aromatic), 2953 (CH, aliphatic), 1746 (C = O), 1647 (C = N), 1513 (C = C), 1223 (C-N), 1118 (C-F); ^1^H NMR (DMSO-*d_6_*) δ: 9.12 (t, *J* = 5.5 Hz, 1H, Ar–H), 8.73 (s, 1H, NH), 8.72 (s, 1H, Ar–H), 7.74 (s, 1H, Ar–H), 7.73 (s, 1H, Ar–H), 7.47 (dd, *J* = 14.9, 1.8 Hz, 1H, Ar–H), 7.18 (d, *J* = 8.5 Hz, 1H, Ar–H), 7.05 (t, *J* = 9.2 Hz, 1H, Ar–H), 4.89–4.84 (m, 1H, CH), 4.15 (t, *J* = 9.0 Hz, 1H, CH_2_), 3.84 (q, *J* = 6.2 Hz, 1H, CH_2_), 3.73 (t, *J* = 3.8 Hz, 4H, 2CH_2_), 3.68–3.62 (m, 2H, CH_2_), 2.95 (t, *J* = 3.8 Hz, 4H, 2CH_2_); ^13^C NMR (DMSO-*d_6_*) δ: 165.5, 155.5, 154.0, 153.6, 150.2, 140.9, 135.5, 133.4, 121.2, 119.2, 114.1, 106.8, 71.2, 66.1, 50.7, 47.5, 42.4; HRMS: *m*/*z* for C_20_H_21_FN_4_O_4_ [M+H]^+^ Calcd.: 401.1620, Found: 401.1617.

#### 3.1.23. (S)-N-((3-(3-Fluoro-4-morpholinophenyl)-2-oxooxazolidin-5-yl)methyl)nicotinamide (**7b**)

White solid, mp: 182–184 °C, yield: 86% (0.59 g). IR: *ν*_max_/cm^−1^; 3286 (NH), 3031 (CH, aromatic), 2938, 2872 (CH, aliphatic), 1737 (C = O), 1651 (C = N), 1511 (C = C), 1219 (C-N), 1195 (C-O), 1109 (C-F); ^1^H NMR (DMSO-*d_6_*) δ: 9.02 (t, *J* = 5.5 Hz, 1H, Ar–H), 8.98 (s, 1H, NH), 8.71 (d, *J* = 3.9 Hz, 1H, Ar–H), 8.17 (d, *J* = 8.0 Hz, 1H, Ar–H), 7.52–7.49 (m, 1H, Ar–H), 7.46 (d, *J* = 2.1 Hz, 1H, Ar–H), 7.19 (dd, *J* = 8.9, 1.2 Hz, 1H, Ar–H), 7.05 (t, *J* = 9.2 Hz, 1H, Ar–H), 4.89–4.84 (m, 1H, CH), 4.15 (t, *J* = 9.0 Hz, 1H, CH_2_), 3.85 (q, *J* = 6.1 Hz, 1H, CH_2_), 3.73 (t, *J* = 4.1 Hz, 4H, 2CH_2_), 3.68–3.62 (m, 2H, CH_2_), 2.95 (t, *J* = 4.2 Hz, 4H, 2CH_2_); ^13^C NMR (DMSO-*d_6_*) δ: 165.6, 155.5, 154.0, 152.0, 148.4, 135.6, 135.0, 133.4, 129.5, 123.4, 119.2, 114.1, 106.7, 71.2, 66.1, 50.7, 47.5, 42.2; HRMS: *m*/*z* for C_20_H_21_FN_4_O_4_ [M+H]^+^ Calcd.: 401.1620, Found: 401.1617.

#### 3.1.24. (S)-N-((3-(3-Fluoro-4-morpholinophenyl)-2-oxooxazolidin-5-yl)methyl)pyrazine-2-carboxamide (**7c**)

White solid, mp: 190–192 °C, yield: 92% (0.63 g). IR: *ν*_max_/cm^−1^; 3389 (NH), 3004 (CH, aromatic), 2906 (CH, aliphatic), 1741, 1681 (C = O), 1603 (C = N), 1514 (C = C), 1224 (C-N), 1192 (C-O), 1115 (C-F); ^1^H NMR (DMSO-*d_6_*) δ: 9.22–9.20 (m, 2H, Ar–H), 8.89 (d, *J* = 2.0 Hz, 1H, Ar–H), 8.75 (s, 1H, NH), 7.46 (dd, *J* = 14.9, 1.7 Hz, 1H, Ar–H), 7.19 (d, *J* = 8.7 Hz, 1H, Ar–H), 7.05 (t, *J* = 9.3 Hz, 1H, Ar–H), 4.91–4.86 (m, 1H, CH), 4.13 (t, *J* = 8.9 Hz, 1H, CH_2_), 3.89 (q, *J* = 5.9 Hz, 1H, CH_2_), 3.73 (t, *J* = 3.9 Hz, 4H, 2CH_2_), 3.69–3.62 (m, 2H, CH_2_), 2.96 (t, *J* = 4.0 Hz, 4H, 2CH_2_); ^13^C NMR (DMSO-*d_6_*) δ: 163.6, 155.5, 154.0, 147.7, 144.4, 143.6, 143.4, 135.6, 133.4, 119.2, 114.2, 106.8, 71.0, 66.1, 50.7, 47.6, 42.0; HRMS: *m*/*z* for C_19_H_20_FN_5_O_4_ [M+H]^+^ Calcd.: 402.1572, Found: 402.1580.

#### 3.1.25. N-((S)-1-((((S)-3-(3-Fluoro-4-morpholinophenyl)-2-oxooxazolidin-5-yl)methyl) amino) -4-methyl-1-oxopentan-2-yl)pyrazine-2-carboxamide (**9a**)

The crude product was purified through column chromatography using 1% MeOH in DCM to obtain shiny yellowish white microcrystals, **9a** in pure form, mp: 90–92 °C, yield: 38% (0.33 g). IR: *ν*_max_/cm^−1^; 3306 (NH), 3062 (CH, aromatic), 2957 (CH, aliphatic), 1747 (C = O), 1662 (C = N), 1514 (C = C), 1224 (C-N), 1171 (C-O), 1115 (C-F); ^1^H NMR (CDCl_3_) δ: 9.13 (s, 1H, NH), 8.69–8.68 (m, 1H, Ar–H), 8.43 (s, 1H, NH), 8.01 (d, *J* = 8.0 Hz, 1H, Ar–H), 7.39 (d, *J* = 14.4 Hz, 1H, Ar–H), 7.33–7.29 (m, 1H, Ar–H), 7.04–7.03 (m, 1H, Ar–H), 6.96–6.95 (m, 1H, Ar–H), 4.76–4.72 (m, 1H, CH), 4.62–4.58 (m, 1H, CH), 3.90 (s, 4H, 2CH_2_), 3.82–3.71 (m, 3H, (2H, CH_2_) + (1H, CH_2_)), 3.57–3.52 (m, 1H, CH_2_), 3.12 (s, 4H, 2CH_2_), 1.78–1.72 (m, 1H, CH), 1.68–1.62 (m, 2H, CH_2_), 0.91 (d, *J* = 6.3 Hz, 3H, CH_3_), 0.93 (d, *J* = 6.3 Hz, 3H, CH_3_); ^13^C NMR (CDCl_3_) δ: 173.1, 163.5, 156.6, 154.4, 147.8, 144.5, 143.7, 142.8, 134.6, 128.5, 120.1, 114.0, 107.8, 72.0, 66.6, 52.3, 51.6, 47.5, 41.6, 40.8, 25.1, 23.1, 22.1; HRMS: *m*/*z* for C_25_H_31_FN_6_O_5_ [M+H]^+^ Calcd.: 515.2413, Found: 515.2418.

#### 3.1.26. N-((S)-1-((((S)-3-(3-Fluoro-4-morpholinophenyl)-2-oxooxazolidin-5-yl)methyl) amino) -3-methyl-1-oxobutan-2-yl)pyrazine-2-carboxamide (**9b**)

The crude product was purified through column chromatography using 1% MeOH in DCM to obtain shiny offwhite microcrystals, **9b** in pure form, mp: 94–96 °C, yield: 78% (0.66 g). IR: *ν*_max_/cm^−1^; 3305 (NH), 3062 (CH, aromatic), 2963 (CH, aliphatic), 1747 (C = O), 1660 (C = N), 1514 (C = C), 1224 (C-N), 1171 (C-O), 1115 (C-F); ^1^H NMR (CDCl_3_) δ: 9.18 (s, 1H, NH), 8.69 (d, *J* = 2.4 Hz, 1H, Ar–H), 8.46 (s, 1H, NH), 8.23 (d, *J* = 8.7 Hz, 1H, Ar–H), 7.51 (t, *J* = 6.0 Hz, 1H, Ar–H), 7.34 (dd, *J* = 14.4, 2.3 Hz, 1H, Ar–H), 6.99 (dd, *J* = 8.8, 2.2 Hz, 1H, Ar–H), 6.89 (t, *J* = 9.0 Hz, 1H, Ar–H), 4.76–4.71 (m, 1H, CH), 4.46 (q, *J* = 6.9 Hz, 1H, CH), 3.91 (t, *J* = 9.0 Hz, 1H, CH_2_), 3.85 (t, *J* = 4.4 Hz, 4H, 2CH_2_), 3.78–3.69 (m, 2H, (1H, CH_2_) + (1H, CH_2_)), 3.62–3.57 (m, 1H, CH_2_), 3.04 (t, *J* = 4.4 Hz, 4H, 2CH_2_), 2.28–2.21 (m, 1H, CH), 0.97 (d, *J* = 6.8 Hz, 3H, CH_3_), 0.93 (d, *J* = 6.8 Hz, 3H, CH_3_); ^13^C NMR (CDCl_3_) δ: 172.3, 163.4, 156.5, 154.6, 147.7, 144.5, 144.0, 142.8, 135.2, 133.9, 119.6, 114.1, 107.8, 72.1, 66.8, 59.0, 53.6, 51.4, 47.7, 41.8, 31.1, 19.6, 18.3; HRMS: *m*/*z* for C_24_H_29_FN_6_O_5_ [M+H]^+^ Calcd.: 501.2256, Found: 501.2269.

#### 3.1.27. N-((S)-1-((((S)-3-(3-Fluoro-4-morpholinophenyl)-2-oxooxazolidin-5-yl)methyl) amino) -1-oxo-3-phenylpropan-2-yl)pyrazine-2-carboxamide (**9c**)

The crude product was purified through column chromatography using 1% MeOH in DCM to obtain a fine pale brown solid, **9c** in pure form, mp: 172–174 °C, yield: 52% (0.49 g). IR: *ν*_max_/cm^−1^; 3306 (NH), 3032 (CH, aromatic), 2858 (CH, aliphatic), 1743, 1681 (C = O), 1665 (C = N), 1514 (C = C), 1227 (C-N), 1146 (C-O), 1115 (C-F); ^1^H NMR (CDCl_3_) δ: 9.14 (s, 1H, Ar–H), 8.70–8.68 (m, 1H, Ar–H), 8.42 (s, 1H, NH), 8.22 (d, *J* = 8.0 Hz, 1H, Ar–H), 7.41–7.35 (m, 1H, Ar–H), 7.22–7.17 (m, 4H, Ar–H), 7.13–6.97 (m, 3H, Ar–H), 4.91–4.81 (m, 1H, CH), 4.69–4.59 (m, 1H, CH), 3.89–3.84 [m, 5H, (4H, 2CH_2_) + (1H, CH_2_)], 3.73–3.50 [m, 3H, (1H, CH_2_) + (2H, CH_2_)], 3.22–3.18 (m, 1H, CH_2_), 3.13–3.08 (m, 5H, (1H, CH_2_) + (4H, 2CH_2_)); ^13^C NMR (CDCl_3_) δ: 171.9, 163.3, 156.6, 154.3, 147.8, 144.5, 143.7, 142.9, 136.3, 129.4, 129.3, 128.9, 127.4, 114.1, 107.6, 71.9, 66.6, 55.0, 51.6, 47.6, 41.7, 38.2; HRMS: *m*/*z* for C_28_H_29_FN_6_O_5_ [M+H]^+^ Calcd.: 549.2256, Found: 549.2254.

#### 3.1.28. General Method for Preparation of Compounds **11a–f**

Different substituted benzyl halides **10a–f** (1.2 eq.) were added to a solution of deacetyl linezolid **1** (500 mg, 1.0 eq.) in anhydrous DCM (10 mL), in the presence of TEA (2.5 eq.) and chilled to 0 °C. The reaction mixture was stirred overnight, allowing the temperature to become room temperature; TLC monitored the progress of the reaction. After completion of the reaction, the solvent was evaporated under reduced pressure and the residue was treated with a few crystals of ice and 10 mL of 10% NaOH solution. The crude product was extracted with ethyl acetate (15 mL) three times and was then purified by column chromatography (1% MeOH in DCM) to get the desired products **11a–f** in pure form.

#### 3.1.29. (S)-3-(3-Fluoro-4-morpholinophenyl)-5-[{(4-fluorobenzyl) amino} methyl] oxazolidin-2-one (**11a**)

Yellow oil, yield: 28% (0.19 g). IR: *ν*_max_/cm^−1^; 3334 (NH), 3074 (CH, aromatic), 2938, 2853 (CH, aliphatic), 1743 (C = O), 1602 (NH bending), 1510 (C = C), 1218 (C-N), 1114 (C-F); ^1^HNMR (CDCl_3_) δ: ^1^H NMR (CDCl_3_) δ: 8.00 (s, 1H, NH), 7.41 (dd, *J* = 14.4, 2.6 Hz, 1H, Ar–H), 7.27–7.24 (m, 2H, Ar–H), 7.09 (dd, *J* = 8.8, 2.2 Hz, 1H, Ar–H), 6.99 (t, *J* = 8.7 Hz, 2H, Ar–H), 6.90 (t, *J* = 9.1 Hz, 1H, Ar–H), 4.76–4.70 (m, 1H, CH), 3.96 (t, *J* = 8.7 Hz, 1H, CH_2_), 3.85 [t, *J* = 4.6 Hz, 5H, (1H, CH) + (4H, 2CH_2_)], 3.82–3.76 (m, 2H, CH_2_), 3.03 (t, *J* = 4.6 Hz, 4H, 2CH_2_), 2.97 (dd, *J* = 13.0, 4.1 Hz, 1H, CH_2_), 2.87–2.83 (m, 1H, CH_2_); ^13^C NMR (CDCl_3_) δ: 161.3, 156.7, 154.7, 136.6, 135.3, 133.6, 130.0, 129.9, 119.1, 115.7, 115.5, 114.0, 107.7, 72.4, 67.2, 53.2, 51.6, 51.2, 48.4; HRMS: *m*/*z* for C_21_H_23_F_2_N_3_O_3_ [M^+^] Calcd.: 403.1707, Found: 403.1728.

#### 3.1.30. (S)-3-(3-Fluoro-4-morpholinophenyl)-5-[{(4-(methylsulfonyl) benzyl} amino] methyl) oxazolidin-2-one (**11b**)

White paste, yield: 32% (0.25 g). IR: *ν*_max_/cm^−1^; 3270 (NH), 3064 (CH, aromatic), 2941, 2850 (CH, aliphatic), 1732 (C = O), 1647 (NH bending), 1515 (C = C), 1228 (C-N), 1195 (C-O), 1114 (C-F); ^1^H NMR (CDCl_3_) δ: 7.96 (s, 1H, NH), 7.94–7.87 (m, 2H, Ar–H), 7.52 (s, 1H, Ar–H), 7.50 (s, 1H, Ar–H), 7.42 (dd, *J* = 14.4, 2.5 Hz, 1H, Ar–H), 7.09 (dd, *J* = 8.8, 2.1 Hz, 1H, Ar–H), 6.91 (t, *J* = 9.1 Hz, 1H, Ar–H), 4.77–4.72 (m, 1H, CH), 3.98 (t, *J* = 8.6 Hz, 1H, CH_2_), 3.93 (d, *J* = 3.4 Hz, 2H, CH_2_), 3.85 (t, *J* = 4.5 Hz, 4H, 2CH_2_), 3.79 (q, *J* = 7.0 Hz, 1H, CH_2_), 3.04–3.03 [m, 5H, (1H, CH_2_) + (4H, 2CH_2_)), 3.03 (s, 3H, CH_3_], 2.85 (q, *J* = 7.1 Hz, 1H, CH_2_); ^13^C NMR (CDCl_3_) δ: 156.7, 154.6, 146.4, 139.7, 136.6, 133.4, 130.1, 129.1, 128.5, 127.9, 119.1, 115.7, 114.0, 107.7, 72.4, 67.2, 53.4, 51.8, 51.3, 48.3, 44.8; HRMS: *m*/*z* for C_22_H_26_FN_3_O_5_S [M^+^] Calcd.: 463.1577, Found: 463.1591.

#### 3.1.31. (S)-3-(3-Fluoro-4-morpholinophenyl)-5-[{(4-(trifluoromethyl) benzyl} amino] methyl) oxazolidin-2-one (**11c**)

Yellow paste, yield: 31% (0.24 g). IR: *ν*_max_/cm^−1^; 3271 (NH), 3062 (CH, aromatic), 2925, 2853 (CH, aliphatic), 1746 (C = O), 1515 (C = C), 1226 (C-N), 1112 (C-F); ^1^H NMR (CDCl_3_) δ: 7.98 (s, 1H, NH), 7.56 (s, 1H, Ar–H), 7.55 (s, 1H, Ar–H), 7.43–7.40 (m, 3H, Ar–H), 7.09 (dd, *J* = 8.8, 1.8 Hz, 1H, Ar–H), 6.90 (t, *J* = 9.1 Hz, 1H, Ar–H), 4.75–4.70 (m, 1H, CH), 3.96 (t, *J* = 8.7 Hz, 1H, CH_2_), 3.89 (d, *J* = 5.1 Hz, 2H, CH_2_), 3.84 (t, *J* = 4.6 Hz, 4H, 2CH_2_), 3.78 (q, *J* = 6.8 Hz, 1H, CH_2_), 3.02 (t, *J* = 4.7 Hz, 4H, 2CH_2_), 2.97 (dd, *J* = 13.0, 4.0 Hz, 1H, CH_2_), 2.84 (q, *J* = 5.9 Hz, 1H, CH_2_); ^13^C NMR (CDCl_3_) δ: 156.7, 154.6, 143.9, 136.5, 133.6, 129.8, 129.6, 129.3, 128.5, 125.6, 123.3, 119.0, 114.0, 107.7, 72.4, 67.1, 53.4, 51.7, 51.2, 48.3; HRMS: *m*/*z* for C_22_H_23_F_4_N_3_O_3_ [M+H]^+^ Calcd.: 454.1748, Found: 454.1759.

#### 3.1.32. (S)-5-[{(3-Chlorobenzyl) amino} methyl]-3-(3-fluoro-4-morpholinophenyl) oxazolidin-2-one (**11d**)

Yellow oil, yield: 26% (0.18 g). IR: *ν*_max_/cm^−1^; 3269 (NH), 3059 (CH, aromatic), 2957, 2853 (CH, aliphatic), 1746 (C = O), 1514 (C = C), 1224 (C-N), 1194 (C-O), 1115 (C-F), 732 (C-Cl); ^1^H NMR (CDCl_3_) δ: 7.99 (s, 1H, NH), 7.42 (dd, *J* = 14.4, 2.5 Hz, 1H, Ar–H), 7.30–7.28 (m, 1H, Ar–H), 7.25–7.20 (m, 2H, Ar–H), 7.17–7.16 (m, 1H, Ar–H), 7.10 (dd, *J* = 8.8, 1.8 Hz, 1H, Ar–H), 6.90 (t, *J* = 9.1 Hz, 1H, Ar–H), 4.74–4.69 (m, 1H, CH), 3.95 (t, *J* = 8.7 Hz, 1H, CH_2_), 3.84 (t, *J* = 4.6 Hz, 4H, 2CH_2_), 3.81–3.77 (m, 3H, (1H, CH_2_) + (2H, CH_2_)), 3.02 (t, *J* = 4.6 Hz, 4H, 2CH_2_), 2.96 (dd, *J* = 13.0, 4.1 Hz, 1H, CH_2_), 2.83 (dd, *J* = 13.2, 6.0 Hz, 1H, CH_2_); ^13^C NMR (CDCl_3_) δ: 156.7, 154.6, 142.0, 136.5, 134.6, 133.6, 130.0, 128.4, 127.6, 126.4, 119.0, 114.0, 107.7, 72.4, 67.2, 53.4, 51.7, 51.2, 48.3; HRMS: *m*/*z* for C_21_H_23_ClFN_3_O_3_ [M^+^] Calcd.: 419.1412, Found: 419.1425.

#### 3.1.33. (S)-5-[{(2,4-Difluorobenzyl) amino} methyl]-3-(3-fluoro-4-morpholinophenyl) oxazolid-ine-2-one (**11e**)

Shiny white microcrystals obtained after purification of the crude product prepared either by the above method or using anhydrous K_2_CO_3_ (3 eq.) in DMF, mp: 74–76 °C, yield: 42% (0.30 g). IR: *ν*_max_/cm^−1^; 3250 (NH), 3068 (CH, aromatic), 2936, 2850 (CH, aliphatic), 1733 (C = O), 1509 (C = C), 1226 (C-N), 1194 (C-O), 1112 (C-F); ^1^H NMR (DMSO-*d_6_*) δ: 7.95 (s, 1H, NH), 7.52–7.47 (m, 2H, Ar–H), 7.20–7.14 (m, 2H, Ar–H), 7.07–7.02 (m, 2H, Ar–H), 4.75–4.70 (m, 1H, CH), 4.05 (t, *J* = 8.9 Hz, 1H, CH_2_), 3.79–3.76 [m, 3H, (1H, CH_2_) + (2H, CH_2_)], 3.73 (t, *J* = 4.7 Hz, 4H, 2CH_2_), 2.96 (t, *J* = 4.7 Hz, 4H, 2CH_2_), 2.80 (d, *J* = 5.2 Hz, 2H, CH_2_); ^13^C NMR (DMSO-*d_6_*) δ: 161.3, 159.4, 155.5, 154.2, 135.3, 133.5, 131.3, 123.7, 119.2, 114.0, 111.2, 106.6, 103.4, 72.4, 66.1, 50.9, 50.7, 47.7, 45.4; HRMS: *m*/*z* for C_21_H_22_F_3_N_3_O_3_ [M^+^] Calcd.: 421.1613, Found: 421.1628.

#### 3.1.34. (S)-5-[{(3,5-Dimethoxybenzyl) amino} methyl]-3-(3-fluoro-4-morpholinophenyl) oxazo-lidine-2-one (**11f**)

Yellow oil, yield: 24% (0.18 g). IR: *ν*_max_/cm^−1^; 3343 (NH), 3031 (CH, aromatic), 2917, 2837 (CH, aliphatic), 1743 (C = O), 1595 (NH bending), 1514 (C = C), 1223 (C-N), 1200 (C-O), 1114 (C-F); ^1^H NMR (CDCl_3_) δ: 8.04 (s, 1H, NH), 7.45 (dd, *J* = 14.4, 2.6 Hz, 1H, Ar–H), 7.13 (dd, *J* = 8.8, 1.8 Hz, 1H, Ar–H), 6.94 (t, *J* = 9.1 Hz, 1H, Ar–H), 6.54 (d, *J* = 2.2 Hz, 2H, Ar–H), 6.39 (t, *J* = 2.2 Hz, 1H, Ar–H), 4.88–4.83 (m, 1H, CH), 4.02 (t, *J* = 8.7 Hz, 1H, CH_2_), 3.90–3.88 [m, 6H, (2H, CH_2_) + (4H, 2CH_2_)], 3.84–3.81 (m, 1H, CH_2_), 3.80 (s, 6H, 2OCH_3_), 3.08–3.04 (m, 5H, (4H, 2CH_2_) + (1H, CH_2_)), 2.98–2.90 (m, 1H, CH_2_); ^13^C NMR (CDCl_3_) δ: 161.3, 156.7, 154.5, 136.7, 136.6, 133.4, 133.3, 119.0, 114.2, 107.8, 106.7, 100.0, 71.7, 67.2, 55.6, 53.4, 51.2, 50.9, 48.5; HRMS: *m*/*z* for C_23_H_28_FN_3_O_5_ [M+Na]^+^ Calcd.: 468.1905, Found: 468.1925.

#### 3.1.35. General Procedure for Preparation of Compound **13**

A round bottom flask (50 mL) containing a small stir bar was charged with deacetyl-linezolid **1** (500 mg, 1.0 eq.) and a solution of 3-nitrooxypropyl bromide **12**, prepared as reported [29] (1.2 eq.) in DMF (20 mL) along with anhydrous K_2_CO_3_ (414 mg, 3.0 eq.). The reaction mixture was stirred at room temperature; TLC monitored the progress of the reaction. After completion of the reaction, the reaction mixture was poured on iced water and extracted with ethyl acetate (15 mL) three times; then, the solvent was evaporated and the residue was purified by column chromatography to get the desired product **13**.

#### 3.1.36. (S)-3-[{(3-(3-Fluoro-4-morpholinophenyl)-2-oxooxazolidin-5-yl) methyl} amino] propyl nitrate (**13**)

White crystals from ethanol, mp: 183–185 °C, yield: 37% (0.25 g). IR:*ν*_max_/cm^−1^; 3052 (CH, aromatic), 2950, 2857 (CH, aliphatic), 1732 (C = O), 1604 (NH bending), 1514 (C = C), 1220 (C-N), 1195 (C-O), 1114 (C-F); ^1^H NMR (DMSO-*d_6_*) δ: 7.49 (dd, *J* = 15.0, 2.4 Hz, 1H, Ar–H), 7.20 (dd, *J* = 8.8, 1.8 Hz, 1H, Ar–H), 7.07 (t, *J* = 9.3 Hz, 1H, Ar–H), 4.92–4.86 (m, 1H, CH), 4.23–4.16 (m, 2H, CH_2_), 4.12 (t, *J* = 9.0 Hz, 1H, CH_2_), 3.77 (q, *J* = 6.5 Hz, 1H, CH_2_), 3.73 (t, *J* = 4.4 Hz, 4H, 2CH_2_), 3.63 (dd, *J* = 14.7, 4.0 Hz, 1H, CH_2_), 3.55 (dd, *J* = 14.6, 7.3 Hz, 1H, CH_2_), 3.45–3.39 (m, 2H, CH_2_), 2.96 (t, *J* = 4.5 Hz, 4H, 2CH_2_), 1.97–1.93 (m, 2H, CH_2_); ^13^C NMR (DMSO-*d_6_*) δ: 155.5, 153.9, 135.6, 133.4, 119.2, 114.2, 106.8, 71.3, 66.6, 66.1, 51.8, 50.7, 47.5, 46.6, 21.8; HRMS: *m*/*z* for C_17_H_23_FN_4_O_6_ [M+H]^+^ Calcd.: 399.1674, Found: 399.1667.

#### 3.1.37. General Procedure for Preparation of Compound **15**

A dried heavy-walled Pyrex tube containing a small stir bar was charged with a solution of benzotriazole activated biotin **14** (1.1 eq.) in THF (3 mL) and de-acetyl linezolid **1** (1.0 eq.) along with triethylamine (1.5 eq.). The reaction mixture was exposed to microwave irradiation (20 W) at 50 °C for 30 min. After completion of the reaction, the solvent was evaporated under reduced pressure and the residue was treated three times with 5 mL of cold 20% Na_2_CO_3_ solution. The precipitate formed was filtered, washed with de-ionized water (10 mL) and dried under vacuum to get the desired product **15** in very good yield.

#### 3.1.38. N-(((S)-3-(3-Fluoro-4-morpholinophenyl)-2-oxooxazolidin-5-yl)methyl)-5-((3aS, 4S, 6aR)-2-oxohexahydro-1H-thieno [3,4-d] imidazol-4-yl)pentanamide (**15**)

White solid, mp: 222–224 °C, yield: 88% (0.77 g). IR: *ν*_max_/cm^−1^; 3442, 3282, 3268 (NH streching), 3062 (CH, aromatic), 2941 (CH, aliphatic), 1731, 1700 (C = O), 1648 (NH bending), 1517 (C = C), 1230 (C-N), 1194 (C-O), 1115 (C-F); ^1^H NMR (DMSO-*d_6_*) δ: 8.19 (s, 1H, NH), 7.48 (dd, *J* = 15.0, 2.4 Hz, 1H, Ar–H), 7.16 (dd, *J* = 8.8 Hz, 2.1 Hz, 1H, Ar–H), 7.06 (t, *J* = 9.2 Hz, 1H, Ar–H), 6.38 (s, 1H, NH), 6.34 (s, 1H, NH), 4.74–4.69 (m, 1H, CH), 4.28 (t, *J* = 5.4 Hz, 1H, CH), 4.09–4.05 [m, 2H, (1H, CH) + (1H, CH_2_)], 3.73 (t, *J* = 4.4 Hz, 4H, 2CH_2_), 3.71–3.69 (m, 1H, CH_2_), 3.47–3.36 (m, 2H, CH_2_), 3.03–2.99 (m, 1H, CH_2_), 2.96 (t, *J* = 4.5 Hz, 4H, 2CH_2_), 2.79 (q, *J* = 5.1 Hz, 1H, CH_2_), 2.56 (d, *J* = 12.4 Hz, 1H, CH), 2.11–2.08 (m, 2H, CH_2_), 1.59–1.37 (m, 4H, 2CH_2_), 1.31–1.17 (m, 2H, CH_2_); ^13^C NMR (DMSO-*d_6_*) δ: 173.0, 162.7, 155.5, 154.0, 135.5, 133.4, 119.2, 114.0, 106.6, 71.5, 66.1, 60.9, 59.2, 55.3, 50.7, 47.2, 41.2, 35.0, 30.7, 28.0, 27.9, 25.3; HRMS: *m*/*z* for C_24_H_32_FN_5_O_5_S [M+H]^+^ Calcd.: 522.2181, Found: 522.2192.

### 3.2. Biological Studies

Antibacterial properties of the synthesized compounds (3a–k, 5a–f, 7a–c, 9a–c, 11a–f, 13 and 15) were screened against Gram-positive (*Staphylococcus aureus* ATCC 6538, *Bacillus subtilis* ATCC 6633) and Gram-negative (*Pseudomonas aeruginosa* ATCC 15,692 and *Escherichia coli* ATCC 47076) bacteria utilizing the standard technique [30]. Linezolid was used as a standard reference/drug in the current study. The tested bacterial strains were obtained from MERCIN Center, Faculty of Agriculture, Ain Shams University, Cairo, Egypt.

To determine the antibacterial activity, stock solutions of the tested compounds and standard reference were prepared with up to 100% DMSO and stored at 20 °C. If necessary, the solutions were heated to 40–60 °C before testing to facilitate complete dissolution. Double distilled water was used in all dilutions prepared. The final concentration of DMSO in the test series was <1% and did not affect the assay results. A microdilution susceptibility test was used for MIC determination (Mueller–Hinton broth in 96-well plates) according to the CLSI (the Clinical and Laboratory Standards Institute, Malvern, PA, USA). The bacterial inoculum was adjusted to approximately 2.5–3 × 10^4^ cells/mL in Mueller–Hinton broth and incubated in a ratio of 1:1 with the test derivatives in polystyrene 96-well flat-bottom microplates. Positive growth control (without test derivatives) and negative control (without bacteria) were included. The microplates were placed in an incubator set to 37 °C for 24 h. Absorbance was measured at 620 nm at the end of the incubation period. The cut-off level for follow-up studies was defined as a minimum of 90% inhibition of growth compared to the untreated sample. Experiments were conducted once in triplicate. For selected compounds, minimum inhibitory concentration (MIC) values were determined by dose–response experiments. Experiments were performed at least twice, in triplicate per concentration tested.

## 4. Conclusions

In summary, we synthesized a total of 31 diverse linezolid conjugates in good yields using benzotriazole chemistry. The synthesized conjugates were screened against four different strains of bacteria following the in vitro standard procedure. Some of the synthesized conjugates show promising antibacterial activity and have drug-like properties. All the biological data were validated by QSAR and 3D-pharmacophore studies. We believe the developed SAR, robust BMLR-QSAR model, and 3D-pharmacophore data could be utilized as useful tools for the development of potential drug candidates.

## Figures and Tables

**Table 1:** Antimicrobial properties of the tested compounds.

Entry	Compd.	MIC, μg/mL ± SE (μmol)			
		*S. Aureus* (ATCC 6538)	*B. Subtilis* (ATCC 6633)	*P. Aeruginosa* (ATCC 15692)	*E. Coli* (ATCC 47076)
1	**3a**	16 ± 2.1 (32.888)	4 ± 0.3 (8.222)	64 ± 1.4 (131.552)	32 ± 1.0 (65.776)
2	**3b**	32 ± 1.5 (63.932)	32 ± 2.1 (63.932)	64 ± 1.9 (127.864)	32 ± 2.5 (63.932)
3	**3c**	32 ± 1.1 (60.540)	32 ± 2.7 (60.540)	64 ± 2.9 (121.079)	32 ± 1.6 (60.540)
4	**3d**	32 ± 1.6 (57.078)	32 ± 2. 4 (57.078)	64 ± 3.1(114.155)	32 ± 2.0 (57.078)
5	**3e**	32 ± 2.2 (55.495)	32 ± 1.9 (55.495)	64 ± 2.1 (110.990)	32 ± 1.0 (55.495)
6	**3f**	32 ± 1.4 (70.721)	8 ± 0.4 (17.680)	32 ± 1.6 (70.721)	32 ± 2.8 (70.721)
7	**3g**	32 ± 1.5 (68.594)	32 ± 2.4 (68.594)	32 ± 1.2 (68.594)	32 ± 2.7 (68.594)
8	**3h**	32 ± 1.3 (64.704)	32 ± 0.7 (64.704)	32 ± 0.5 (64.704)	32 ± 1.1 (64.704)
9	**3i**	32 ± 2.1 (60.765)	32 ± 1.4 (60.765)	32 ± 0.8 (60.765)	32 ± 0.5 (60.765)
10	**3j**	64 ± 2.5 (117.948)	32 ± 1.3 (58.974)	64 ± 1.8 (117.948)	32 ± 0.9 (58.974)
11	**3k**	32 ± 2.1 (62.919)	32 ± 1.3 (62.919)	32 ± 1.3 (62.919)	32 ± 2.1 (62.919)
12	**5a**	4 ± 0.2 (10.015)	1 ± 0.1 (2.504)	32 ± 0.8 (80.116)	32 ± 1.6 (80.116)
13	**5b**	4 ± 0.1 (9.583)	1 ± 0.1 (2.396)	32 ± 1.2 (76.663)	32 ± 2.4 (76.663)
14	**5c**	8 ± 0.3 (18.439)	2 ± 0.1 (4.610)	32 ± 1.9 (73.757	32 ± 0.7 (73.757)
15	**5d**	2 ± 0.1 (4.500)	1 ± 0.1 (2.250)	32 ± 1.6 (72.004)	32 ± 2.2 (72.004)
16	**5e**	4 ± 0.2 (9.336)	1 ± 0.1 (2.334)	32 ± 2.2 (74.686)	32 ± 1.6 (74.686)
17	**5f**	8 ± 0.2 (16.706)	4 ± 0.3 (8.353)	32 ± 1.4 (66.825)	32 ± 0.6 (66.825)
18	**7a**	8 ± 0.3 (19.980)	2 ± 0.2 (4.995)	8 ± 0.1 (19.980)	32 ± 0.9 (79.918)
19	**7b**	8 ± 0.2 (19.980)	2 ± 0.1 (4.995)	32 ± 1.2 (79.918)	32 ± 1.0 (79.918)
20	**7c**	8 ± 0.1 (19.930)	2 ± 0.1 (4.983)	32 ± 1.1 (79.721)	32 ± 0.7 (79.721)
21	**9a**	32 ± 0.4 (62.189)	32 ± 2.0 (62.189)	32 ± 1.3 (62.189)	32 ± 1.6 (62.189)
22	**9b**	32 ± 1.7 (63.932)	32 ± 1.3 (63.932)	32 ± 2.5 (63.932)	32 ± 3.4 (63.932)
23	**9c**	32 ± 0.9 (58.332)	32 ± 0.5 (58.332)	32 ± 0.8 (58.332)	32 ± 1.0 (58.332)
24	**11a**	32 ± 2.7 (79.320)	32 ± 1.2 (79.320)	32 ± 2.2 (79.320)	32 ± 2.5 (79.320)
25	**11b**	32 ± 1.2 (69.037)	32 ± 1.4 (69.037)	64 ± 2.8 (138.074)	32 ± 1.1 (69.037)
26	**11c**	32 ± 0.8 (70.572)	16 ± 0.4 (35.286)	64 ± 1.5 (141.143)	32 ± 0.9 (70.572)
27	**11d**	32 ± 1.0 (76.212)	32 ± 1.2 (76.212)	32 ± 1.7 (76.212)	32 ± 2.1 (76.212)
28	**11e**	32 ± 0.2 (75.934)	32 ± 1.3 (75.934)	32 ± 0.5 (75.934)	32 ± 0.3 (75.934)
29	**11f**	32 ± 1.7 (71.831)	32 ± 0.3 (71.831)	32 ± 1.9 (71.831)	32 ± 0.8 (71.831)
30	**13**	32 ± 1.0 (80.323)	32 ± 0.9 (80.323)	32 ± 1.5 (80.323)	32 ± 0.6 (80.323)
31	**15**	32 ± 0.4 (61.349)	32 ± 0.2 (61.349)	32 ± 0.3 (61.349)	32 ± 0.7 (61.349)
32	Linezolid	2 ± 0.1 (5.929)	0.25 ± 0.01 (0.741)	32 ± 0.7 (94.857)	8 ± 0.2 (23.714)
33	Ciprofloxacin [24]	1250.0 ± 9.6 (3772.4)	NT	4.8 ± 0.5 (14.5)	NT
34	Norfloxacin [24]	1250.0 ± 14.2 (3914.3)	NT	4.8 ± 0.2 (15.0)	NT

NT = not tested.

**Table 2:** Computational ADMET descriptor values for the synthesized agents.

Entry	Compd.	Aqueous Solubility	Intestinal Absorption	PPB	Hepatotoxicity
1	**3a**	3	0	0	0
2	**3b**	3	0	0	0
3	**3c**	2	0	0	0
4	**3d**	3	0	0	0
5	**3e**	2	1	1	0
5	**3f**	3	0	0	0
7	**3g**	3	0	0	0
8	**3h**	3	0	0	0
9	**3i**	3	0	0	0
10	**3j**	2	0	0	0
11	**3k**	2	0	0	0
12	**5a**	3	0	2	0
13	**5b**	2	0	2	0
14	**5c**	2	0	2	0
15	**5d**	3	0	2	0
16	**5e**	3	0	2	0
17	**5f**	2	0	2	0
18	**7a**	3	0	2	0
19	**7b**	3	0	2	0
20	**7c**	3	0	2	0
21	**9a**	3	0	0	0
22	**9b**	3	0	0	0
23	**9c**	3	0	0	0
24	**11a**	2	0	2	0
25	**11b**	3	0	2	0
26	**11c**	2	0	2	0
27	**11d**	2	0	2	0
28	**11e**	2	0	2	0
29	**11f**	2	0	2	0
30	**13**	3	0	0	1
31	**15**	3	0	0	0

Aqueous solubility level: 0, extremely low; 1, very low; 2, low; 3, good; 4; optimal; 5, too soluble; 6, unknown. Intestinal absorption level: 0, good; 1, moderate; 2, poor; 4, very poor. Plasma protein binding (PPB) level: 0, <90%; 1, >90%; 2, >95%. Hepatotoxicity level: 0, non toxic; 1, toxic.

## Data Availability

Data is contained within the article or supplementary material.

## Supplementary Materials

The following supporting information can be downloaded at: https://www.mdpi.com/article/10.3390/ph15020191/s1, 1H NMR, 13C NMR, HRMS and IR spectra of all compounds; Table S1: Descriptors of the QSAR model for the tested agents against *S. aureus*; Table S2: Observed and estimated antimicrobial properties for the tested compounds agents against *S. aureus* according to the BMLR-QSAR model; Table S3: Molecular descriptor values of the QSAR model for the tested compounds against *S. aureus;* Table S4: Descriptors of the QSAR model for the tested agents against *B. subtilis*; Table S5: Observed and estimated antimicrobial properties for the tested compounds agents against *B. subtilis* according to the BMLR-QSAR model; Table S6: Molecular descriptor values of the QSAR model for the tested compounds against *B. subtilis*; Table S7. Observed and estimated activity values for the tested compounds against *S. aureus* according to the 3D-pharmacophore model; Table S8. Observed and estimated activity values for the tested compounds against *B. subtilis* according to the 3D-pharmacophore model; Figure: S1. Dose-response curve for the tested compounds against RPE1 (retinal pigment epithelium) cell line; Figure S2: QSAR plot representing the observed versus predicted log(MIC, *μ*M) for the tested compounds against *S. aureus*; Figure S3: QSAR plot representing the observed versus predicted log(MIC, *μ*M) for the tested compounds against *B. subtilis*; Figure S4: (A) Constraint distances “H-1 – H-2 = 3.696, H-1 – HBA = 3.768, H-2 – HBA = 6.837 Å”, (B) Constraint angle “H-2 – H-1 – HBA = 132.68 °” of the generated 3D-pharmacophore for the tested compounds against *S. aureus*; Figure S5: 3D-pharmacophore model mapped on the tested compounds against *S. aureus*; Figure S6: (A) Constraint distances “HBA-1 – HBA-2 = 10.738, HBA-1 – HBA-3 = 3.833, HBA-2 – HBA-3 = 8.105, HBA-1 – H = 6.626, HBA-2 – H = 5.232, HBA-3 – H = 3.905 Å”, (B) Constraint angle “HBA-1 – HBA-2 – HBA-3 = 17.17, HBA-1 – HBA-2 – H = 28.48, HBA-1 – HBA-3 – H = 117.82, HBA-2 – HBA-2 – H = 32.21 °” of the generated 3D-pharmacophore for the tested compounds against *B. subtilis*; Figure S7: 3D-pharmacophore model mapped on the tested compounds against *B. subtilis*.
