# Supplementary material for: Synthesis, Antibacterial Evaluation, and Computational Studies of a Diverse Set of Linezolid Conjugates
Source: Pharmaceuticals (Basel). 2022 Feb 3;15(2):191. doi: 10.3390/ph15020191 (PMC8880098; doi:10.3390/ph15020191)
Supplement: Supplementary file 1 [file pharmaceuticals-15-00191-s001.zip › pharmaceuticals-1561661-supplementary.pdf]

Supplementary Information

Synthesis, Antibacterial Evaluation, and Computational Studies of a Diverse Set of Linezolid Conjugates

Riham M. Bokhtia [1,2], Adel S. Girgis [3], Tarek S. Ibrahim [4], Fatma Rasslan [5], Eman S. Nossier [6], Reham F. Barghash [3], Rajeev Sakhuja [7], Eatedal H. Abdel-Aal [1], Siva S. Panda [2,*] and Amany M. M. Al-Mahmoudy [1]

[1] Department of Pharmaceutical Organic Chemistry, Faculty of Pharmacy, Zagazig University, Zagazig 44519, Egypt; rehamabdelrehem@yahoo.com (R.M.B.); eatedalabdelaal@yahoo.com (E.H.A.-A.); amanysinger77@gmail.com (A.M.M.A.-M.)

[2] Department of Chemistry and Physics, Augusta University, Augusta, GA 30912, USA

[3] Department of Pesticide Chemistry, National Research Centre, Dokki, Giza 12622, Egypt; girgisas10@yahoo.com (A.S.G.); reham_fawzy@yahoo.com (R.F.B.)

[4] Department of Pharmaceutical Chemistry, Faculty of Pharmacy, King Abdulaziz University, Jeddah 21589, Saudi Arabia; tmabrahem@kau.edu.sa (T.S.I.)

[5] Department of Microbiology and Immunology, Faculty of Pharmacy (Girls), Al Azhar University, Cairo 11651, Egypt; fatma_rasslan@yahoo.com (F.R.)

[6] Department of Pharmaceutical Medicinal Chemistry and Drug Design, Faculty of Pharmacy (Girls), Al-Azhar University, Cairo 11651, Egypt; dr.emannossier@gmail.com (E.S.N)

[7] Department of Chemistry, Birla Institute of Technology and Science, Pilani 333031, India; sakhuja.rajeev@gmail.com (R.S.)

* Correspondence: sspanda12@gmail.com or sipanda@augusta.edu

(3a)

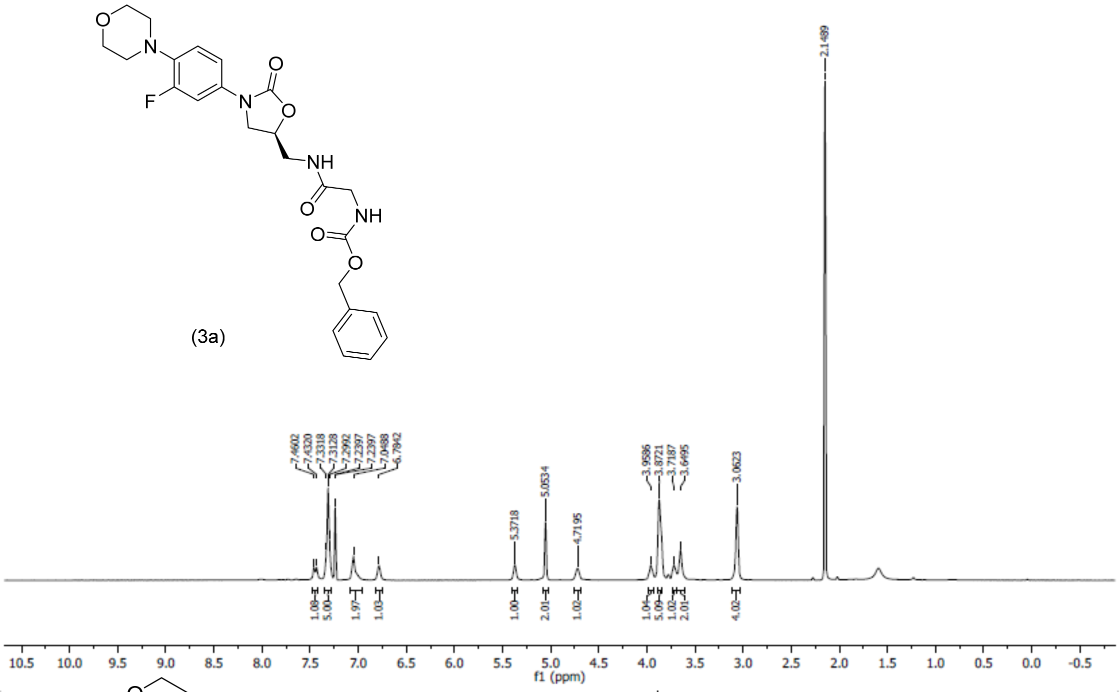

(3a)

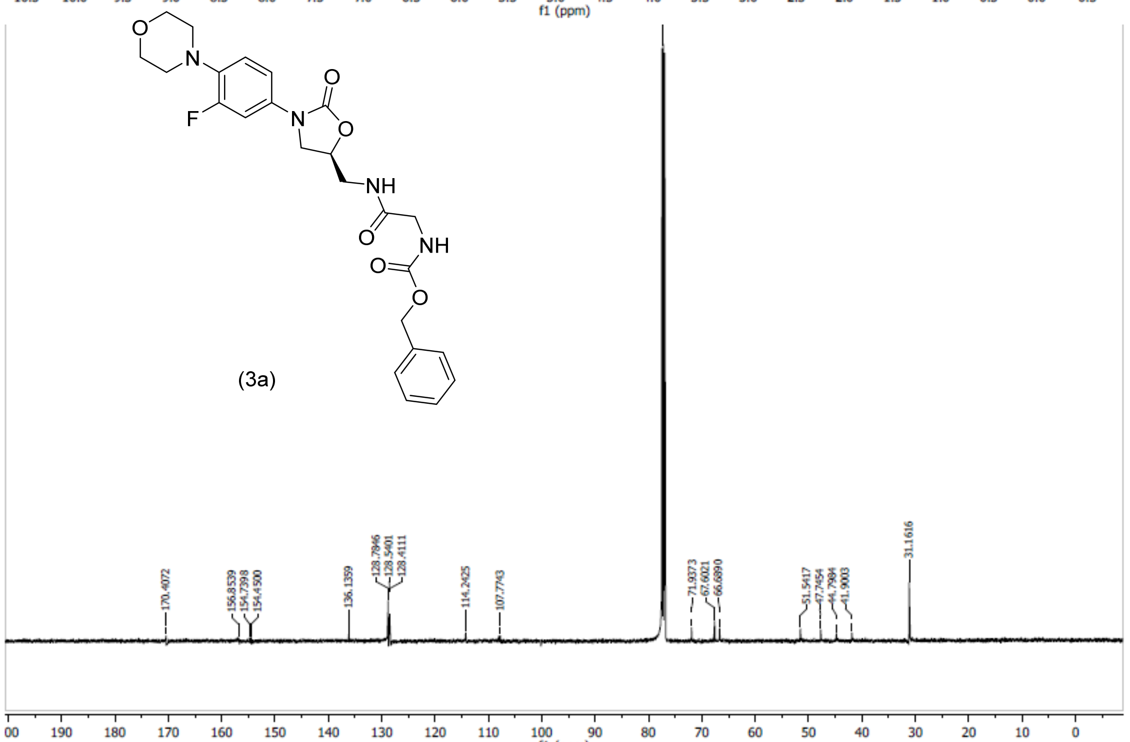

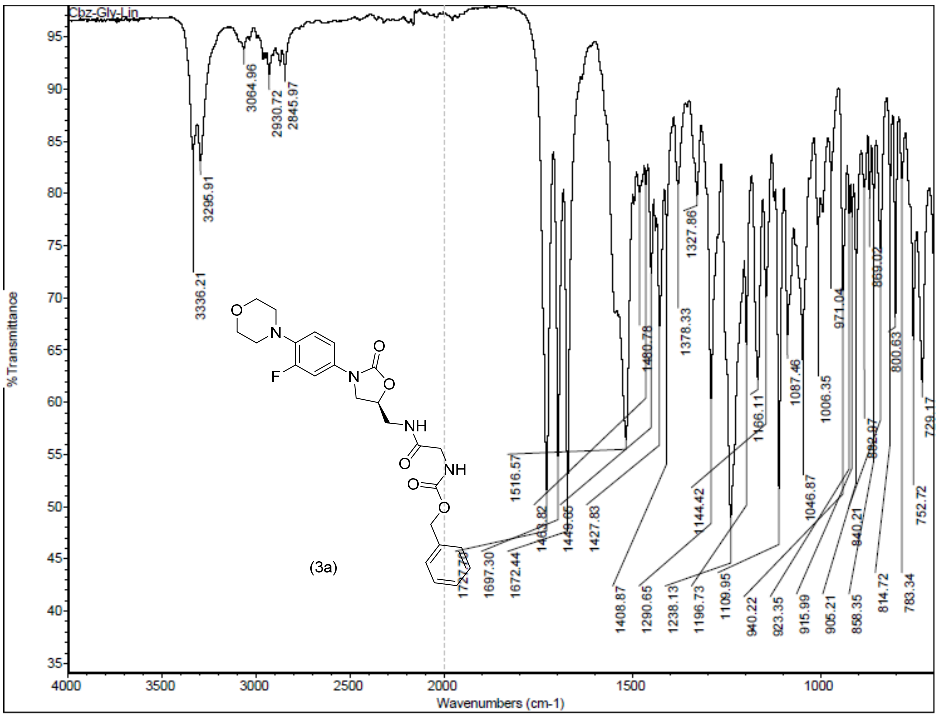
Cbz-Gly-Lin
%Transmittance
Wavenumbers (cm-1)
(3a)
3336.21
3295.91
3064.96
2930.72
2845.97
1516.57
1722.20
1697.30
1672.44
1463.82
1449.66
1427.83
1480.78
1408.87
1378.33
1327.86
1290.65
1238.13
1196.73
1144.42
1166.11
1109.95
1087.46
1046.87
1006.35
971.04
940.22
923.35
915.99
905.21
892.87
869.02
858.35
840.21
814.72
800.63
783.34
752.72
729.17

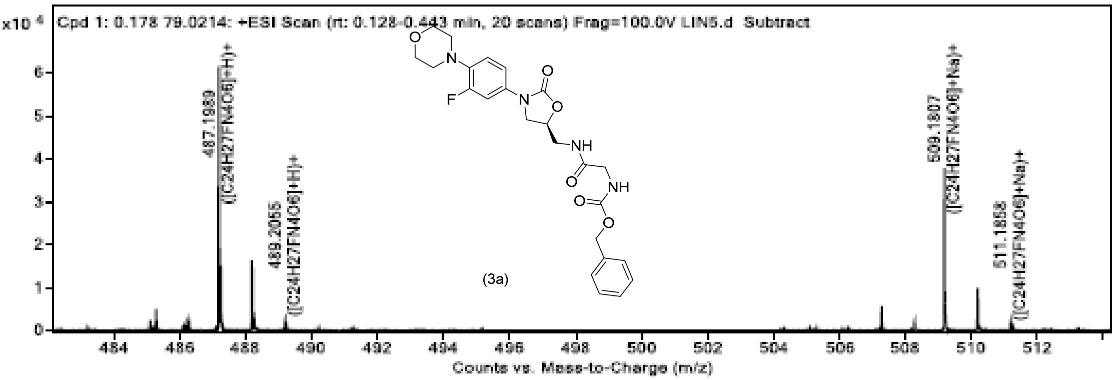
Cpd 1: 0.178 79.0214: +ESI Scan (rt: 0.128-0.443 min, 20 scans) Frag=100.0V LIN5.d Subtract
x10 4
487.1989
([C24H27FN4O6]+H)+
489.2055
([C24H27FN4O6]+H)+
509.1807
([C24H27FN4O6]+Na)+
511.1858
([C24H27FN4O6]+Na)+
(3a)
Counts vs. Mass-to-Charge (m/z)

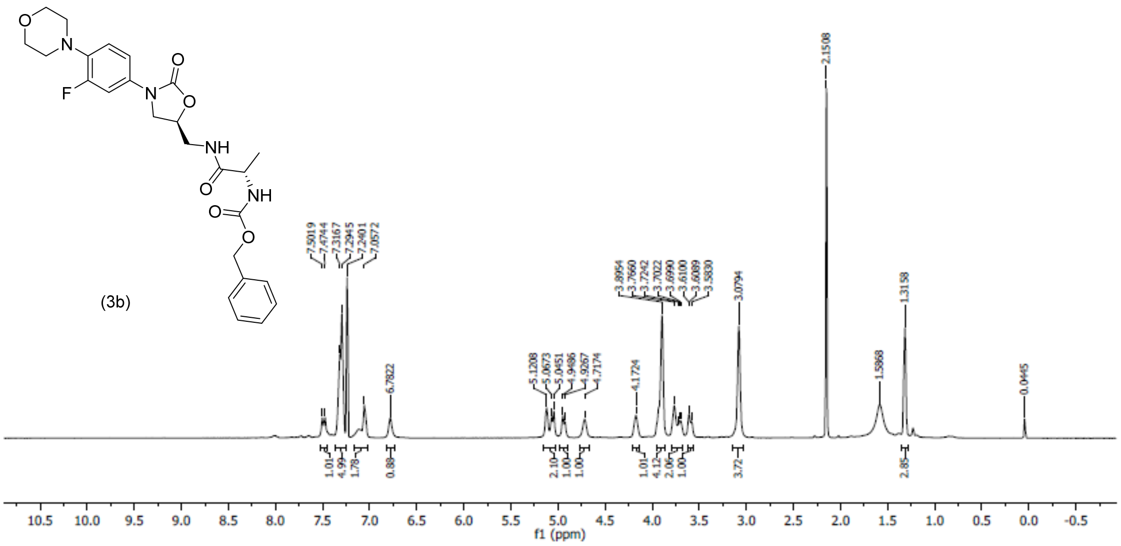

(3b)
7.5019
7.4744
7.3167
7.2945
7.2401
7.0572
6.7822
5.1208
5.0673
5.0451
4.9486
4.9267
4.7174
4.1724
3.8954
3.7660
3.7242
3.7022
3.6990
3.6100
3.6089
3.5830
3.0794
2.1508
1.5868
1.3158
0.0445
1.01
4.99
1.78
0.88
2.10
1.00
1.00
1.01
4.12
2.06
1.00
3.72
2.85
f1 (ppm)

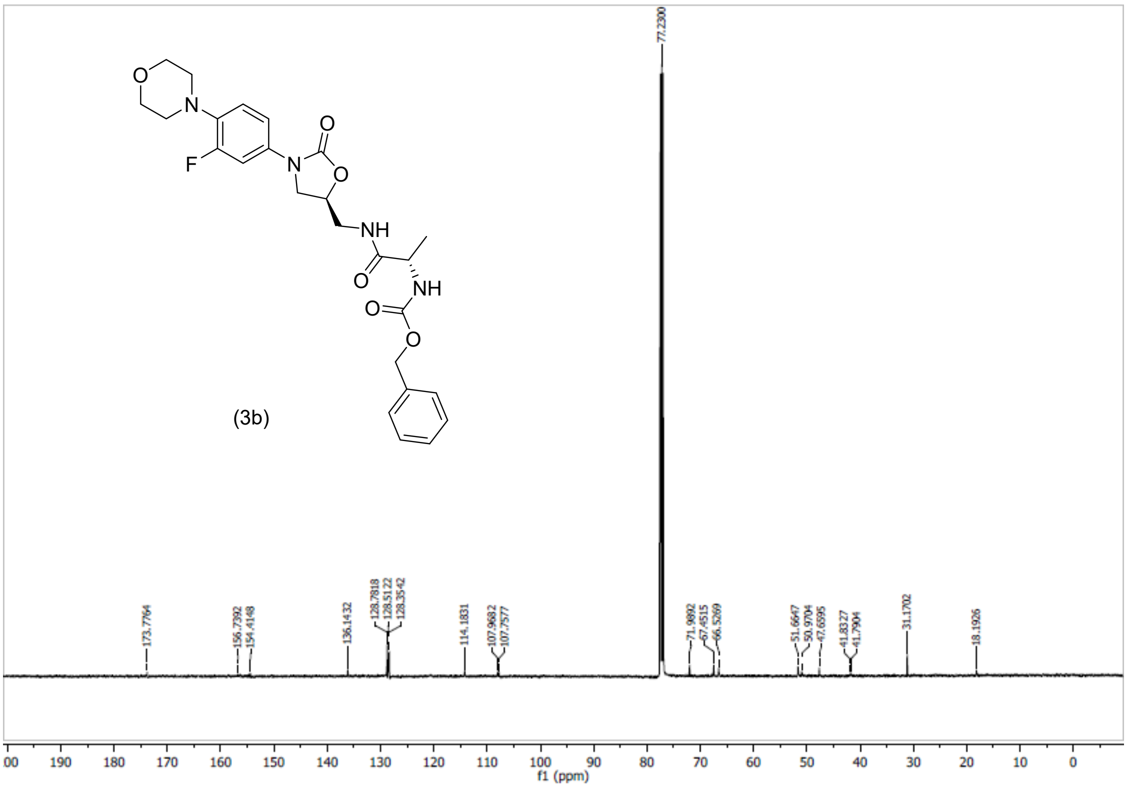

(3b)
77.2300
173.7764
156.7392
154.4148
136.1432
128.7818
128.5122
128.3542
114.1831
107.9682
107.7577
71.9892
67.4515
66.5269
51.6647
50.9704
47.6595
41.8327
41.7904
31.1702
18.1926
f1 (ppm)

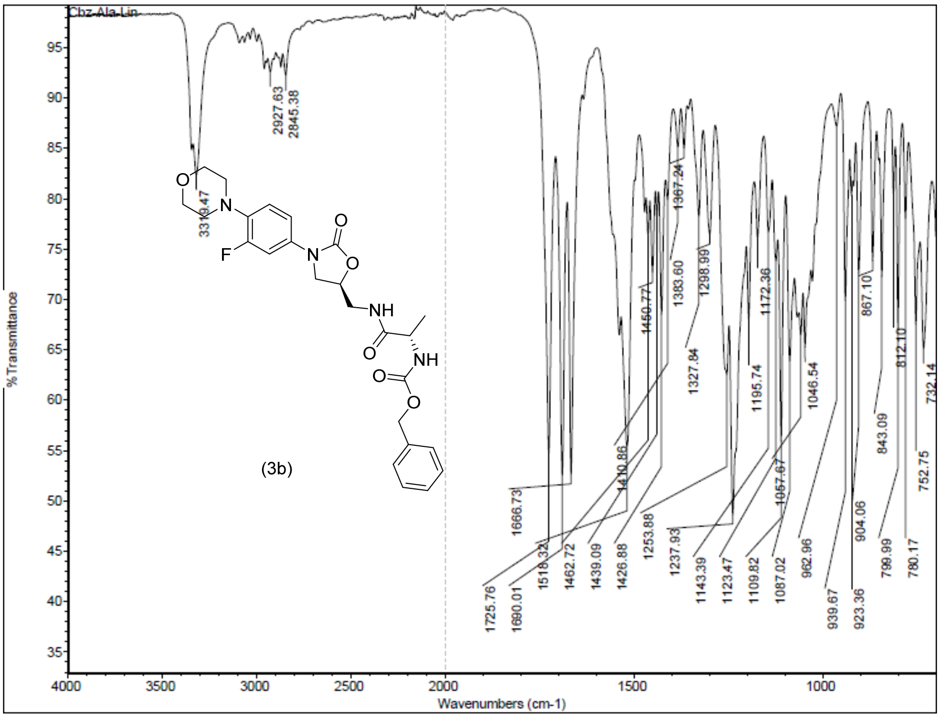
Cbz-Ala-Lin
%Transmittance
Wavenumbers (cm-1)
(3b)
3319.47
2927.63
2845.38
1725.76
1690.01
1666.73
1518.32
1462.72
1450.77
1439.09
1426.88
1410.86
1383.60
1367.24
1327.84
1298.99
1253.88
1237.93
1195.74
1172.36
1143.39
1123.47
1109.82
1087.02
1067.67
1046.54
962.96
939.67
923.36
904.06
867.10
843.09
812.10
799.99
780.17
752.75
732.14

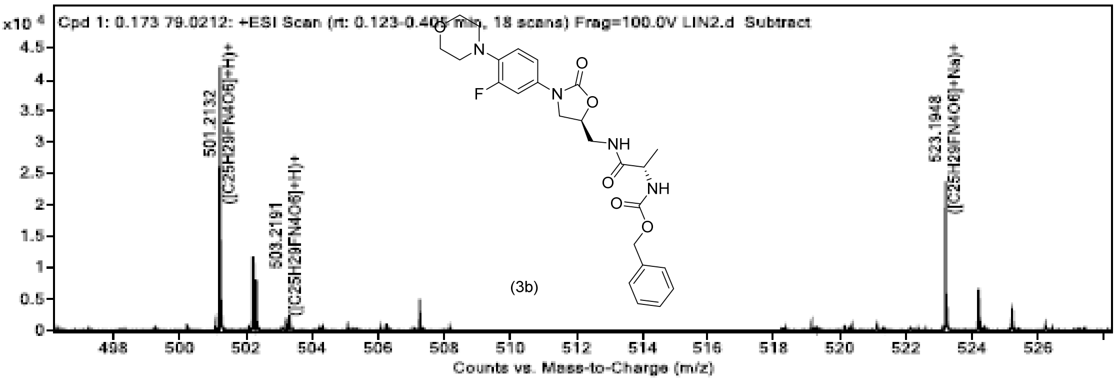
Cpd 1: 0.173 79.0212: +ESI Scan (rt: 0.123-0.405 min, 18 scans) Frag=100.0V LIN2.d Subtract
x10 4
501.2132
([C25H29FN4O6]+H)+
503.2191
([C25H29FN4O6]+H)+
523.1948
([C25H29FN4O6]+Na)+
(3b)
Counts vs. Mass-to-Charge (m/z)

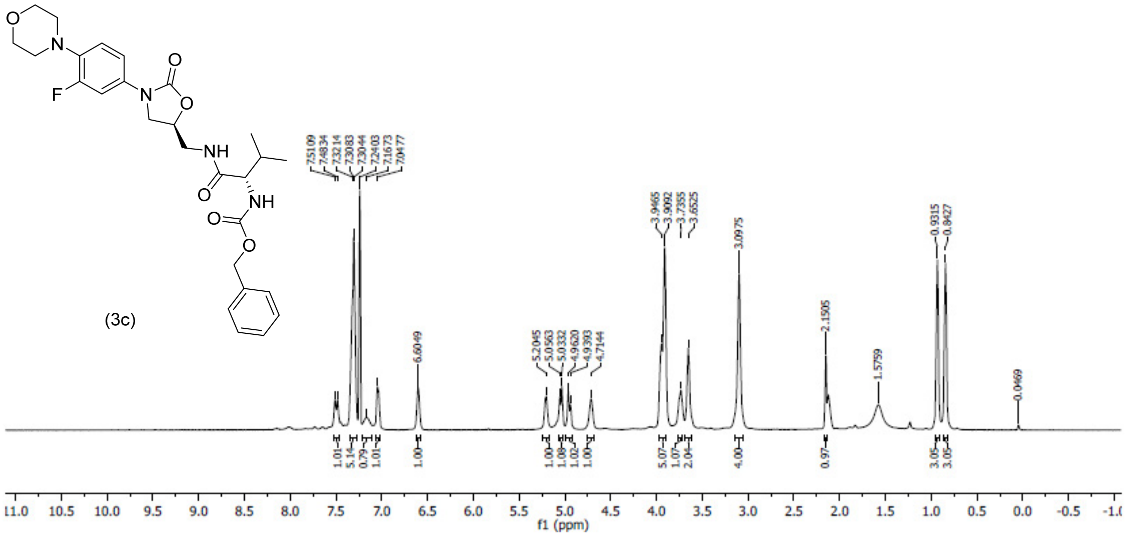

(3c)

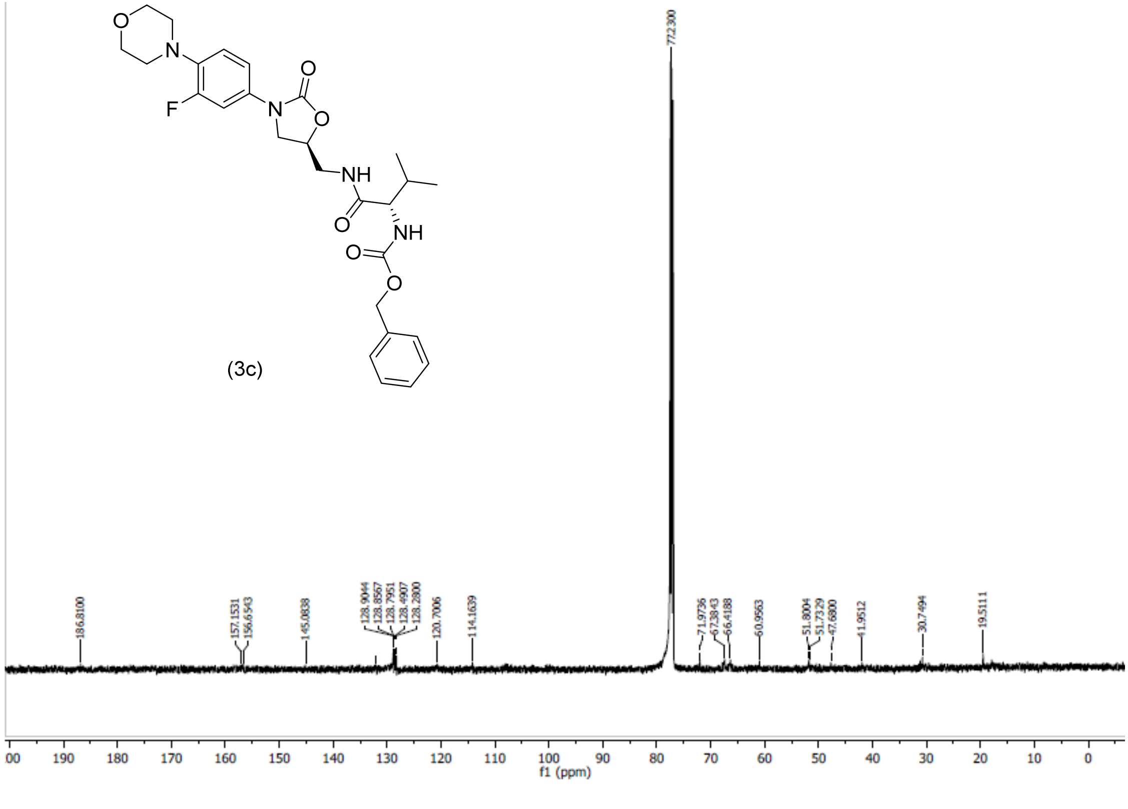

(3c)

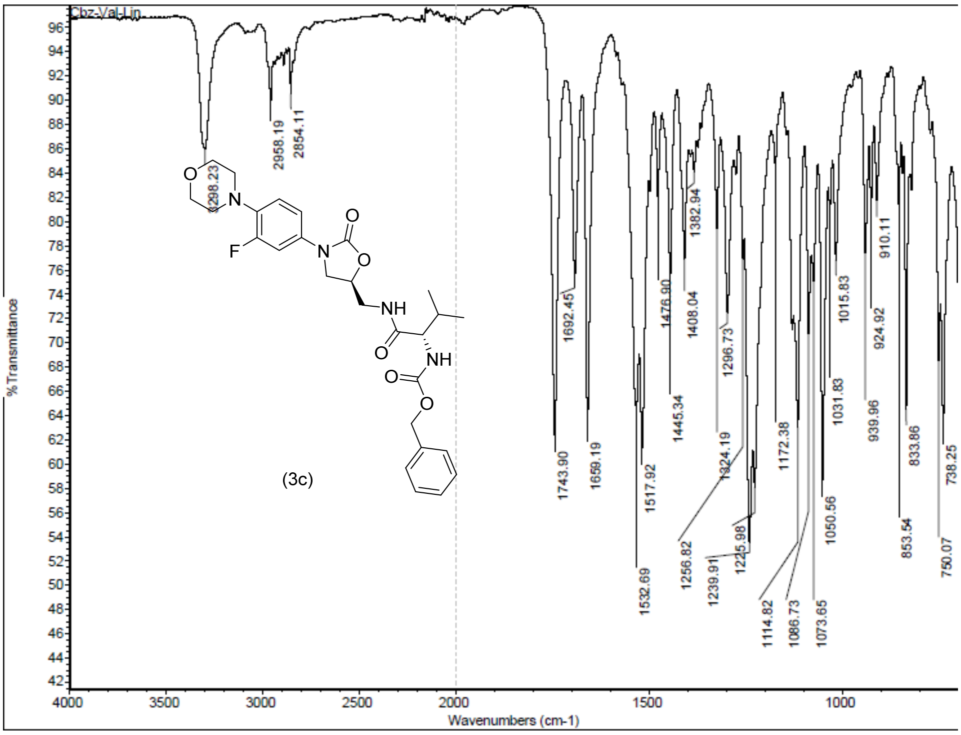
Cbz-Val-Lin
%Transmittance
Wavenumbers (cm-1)
3298.23
2958.19
2854.11
1743.90
1692.45
1659.19
1532.69
1517.92
1476.90
1445.34
1408.04
1382.94
1324.19
1296.73
1256.82
1239.91
1225.98
1172.38
1114.82
1086.73
1073.65
1050.56
1031.83
1015.83
939.96
924.92
910.11
853.54
833.86
750.07
738.25
(3c)

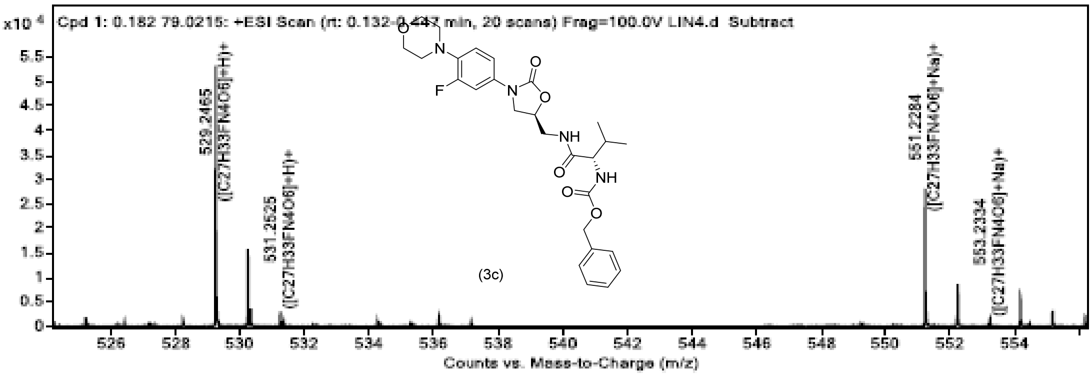
Cpd 1: 0.182 79.0215: +ESI Scan (rt: 0.132-0.447 min, 20 scans) Frag=100.0V LIN4.d Subtract
529.2465 ([C27H33FN4O6]+H)+
531.2525 ([C27H33FN4O6]+H)+
551.2284 ([C27H33FN4O6]+Na)+
553.2334 ([C27H33FN4O6]+Na)+
(3c)
Counts vs. Mass-to-Charge (m/z)

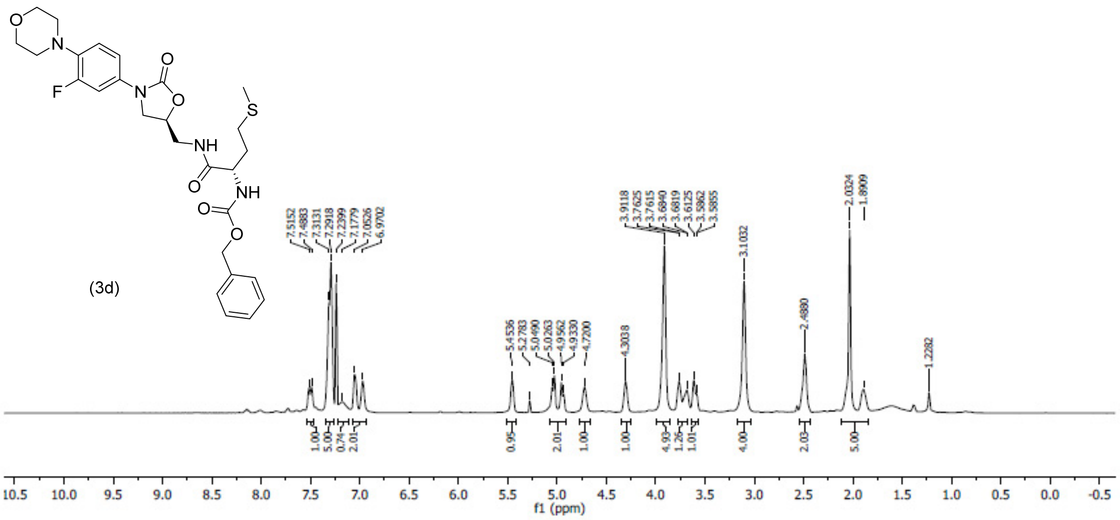
(3d)
7.5152
7.4883
7.3131
7.2918
7.2399
7.1779
7.0626
6.9702
5.4536
5.2783
5.0490
5.0263
4.9562
4.9330
4.7200
4.3038
3.9118
3.7625
3.7615
3.6840
3.6819
3.6125
3.5862
3.5855
3.1032
2.4880
2.0324
1.8909
1.2282
f1 (ppm)

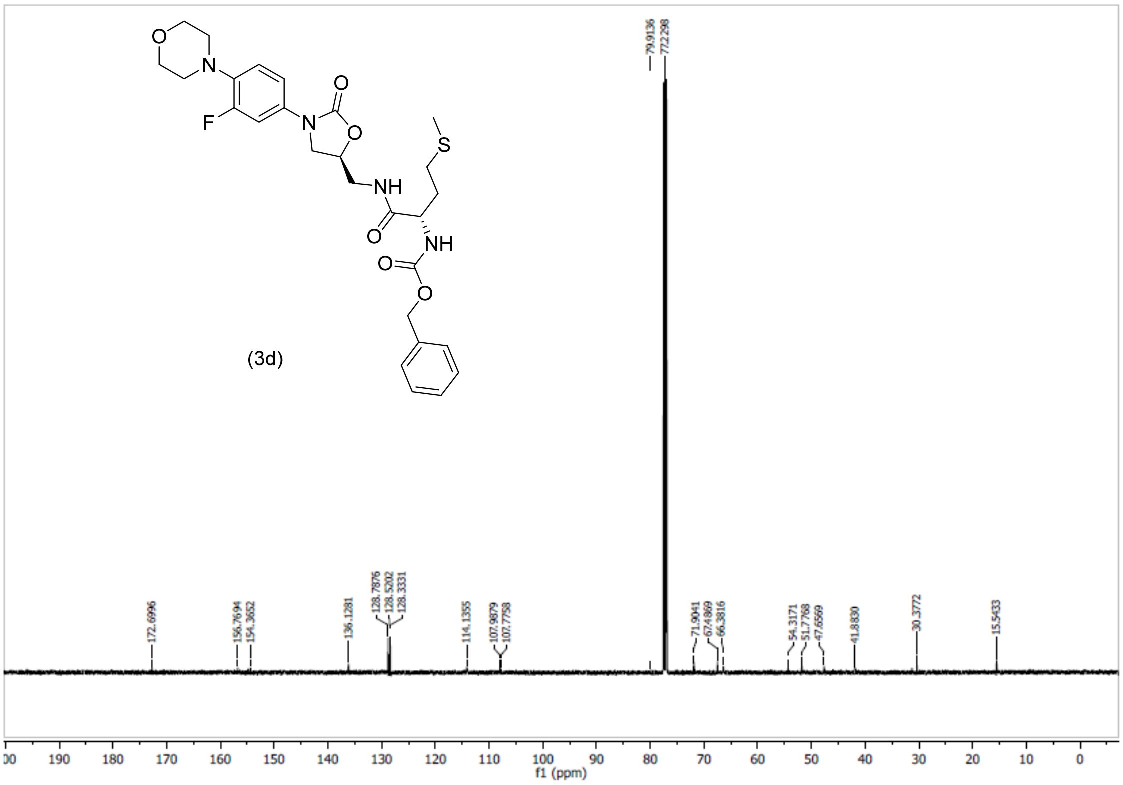
(3d)
79.9136
77.2298
172.6996
156.7694
154.3652
136.1281
128.7876
128.5202
128.3331
114.1355
107.9879
107.7758
71.9041
67.4869
66.3816
54.3171
51.7768
47.6569
41.8830
30.3772
15.5433
f1 (ppm)

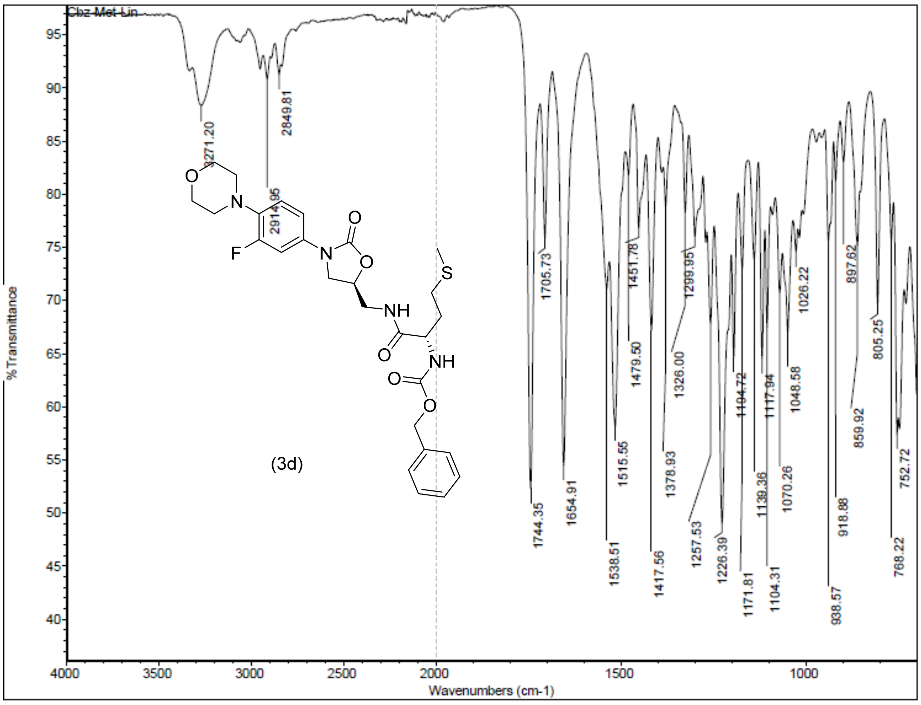

Cbz-Met-Lin
% Transmittance
Wavenumbers (cm-1)
3271.20
2914.95
2849.81
1744.35
1705.73
1654.91
1538.51
1515.55
1479.50
1451.78
1417.56
1378.93
1326.00
1299.95
1257.53
1226.39
1194.72
1171.81
1139.36
1117.94
1104.31
1070.26
1048.58
1026.22
938.57
918.88
897.62
859.92
805.25
768.22
752.72
F
O
N
NH
NH
S
(3d)

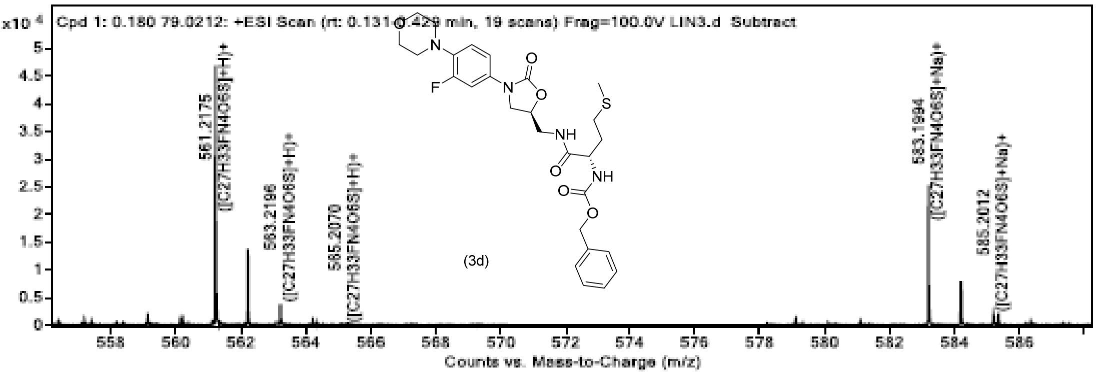

Cpd 1: 0.180 79.0212: +ESI Scan (rt: 0.131-0.429 min, 19 scans) Frag=100.0V LIN3.d Subtract
x10 4
Counts vs. Mass-to-Charge (m/z)
561.2175
([C27H33FN4O6S]+H)+
563.2196
([C27H33FN4O6S]+H)+
565.2070
([C27H33FN4O6S]+H)+
583.1994
([C27H33FN4O6S]+Na)+
585.2012
([C27H33FN4O6S]+Na)+
(3d)

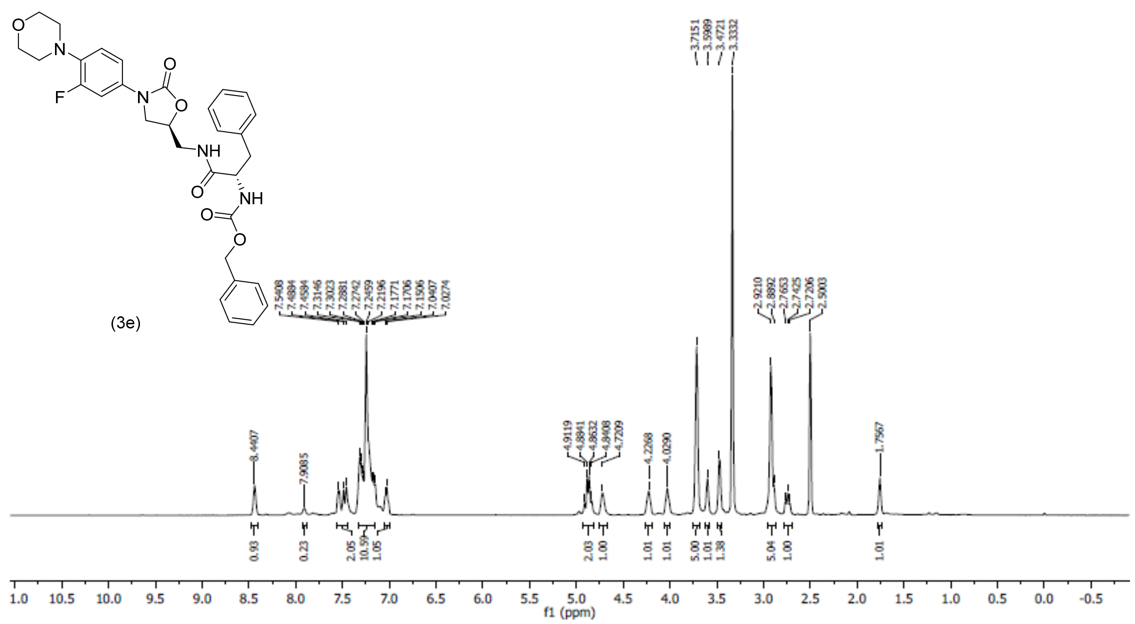

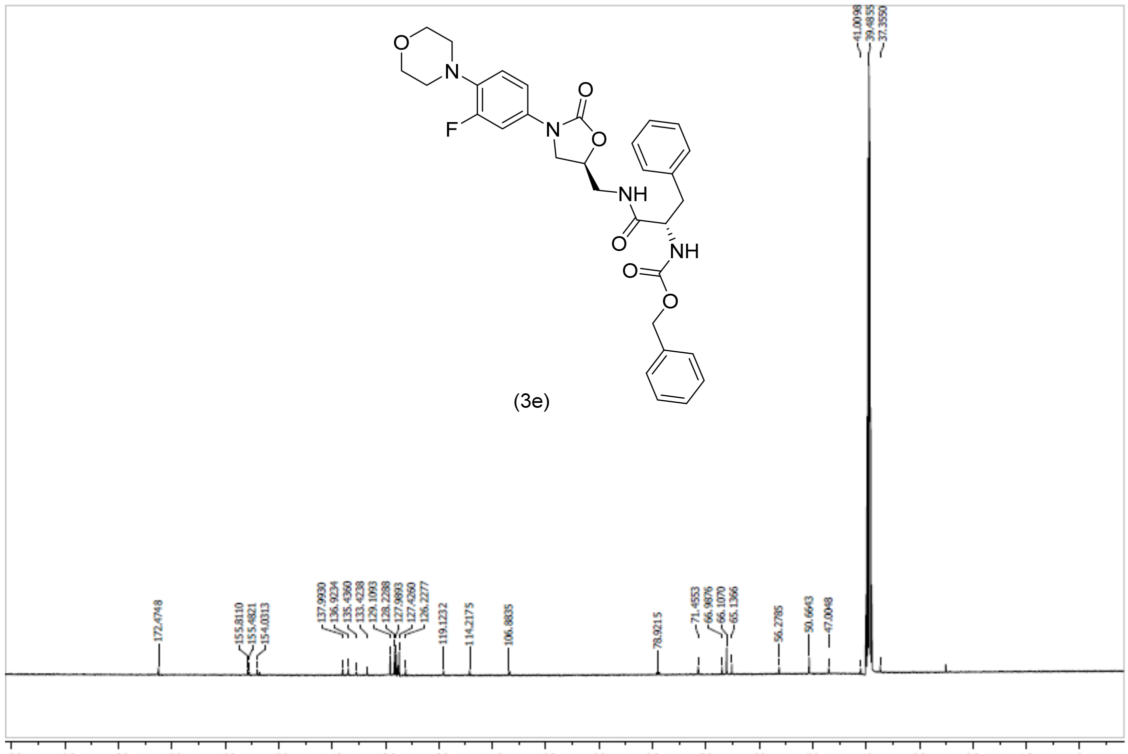

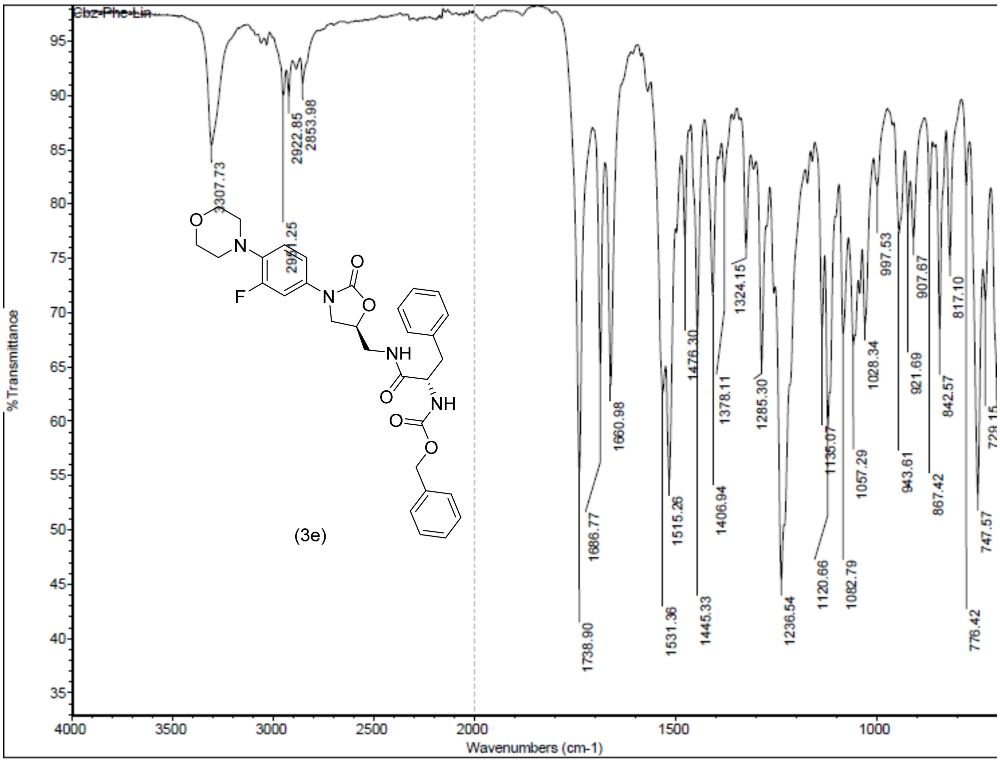

Cbz-Phe-Lin
%Transmittance
Wavenumbers (cm-1)
3307.73
2951.25
2922.85
2853.98
1738.90
1686.77
1660.98
1531.36
1515.26
1476.30
1445.33
1406.94
1378.11
1324.15
1285.30
1236.54
1135.07
1120.66
1082.79
1057.29
1028.34
997.53
943.61
921.69
907.67
867.42
842.57
817.10
776.42
747.57
729.15
(3e)

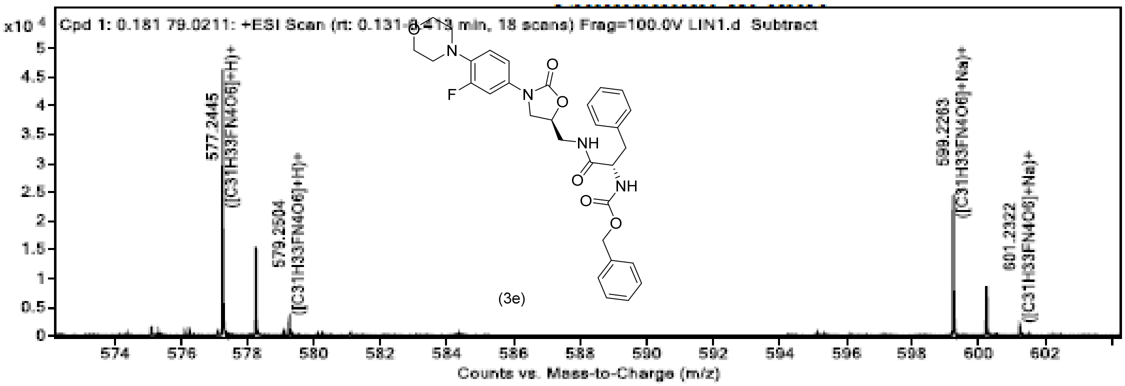

Cpd 1: 0.181 79.0211: +ESI Scan
18 scans) Frag=100.0V LIN1.d Subtract
577.2445
([C31H33FN4O6]+H)+
579.2504
([C31H33FN4O6]+H)+
599.2263
([C31H33FN4O6]+Na)+
601.2322
([C31H33FN4O6]+Na)+
(3e)
Counts vs. Mass-to-Charge (m/z)

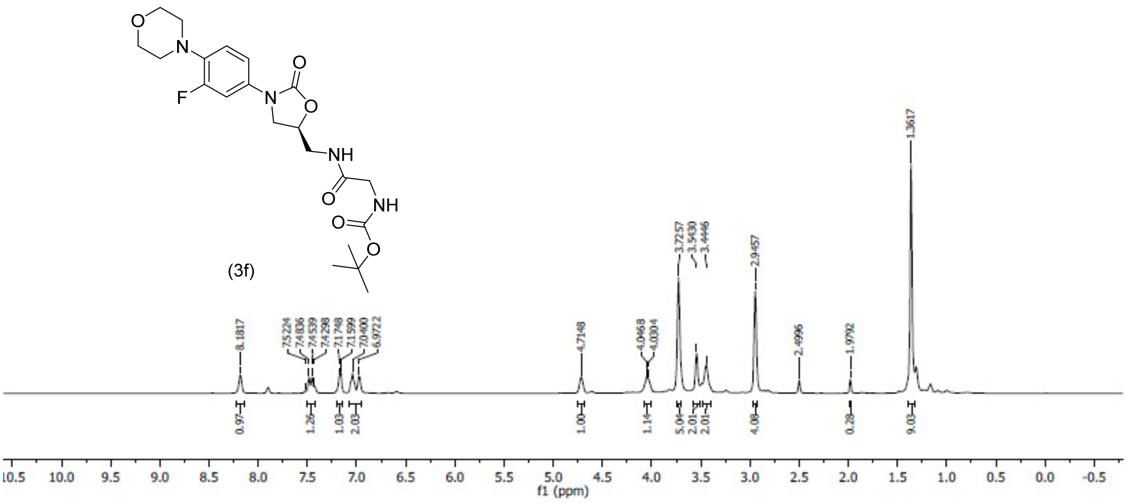
O
N
F
N
O
O
NH
O
NH
O
O
(3f)
8.1817
7.5224
7.4836
7.4539
7.4298
7.1748
7.1599
7.0400
6.9722
4.7148
4.0468
4.0304
3.7257
3.5430
3.4446
2.9457
2.4996
1.9792
1.3617
0.97
1.26
1.03
2.03
1.00
1.14
5.04
2.01
2.01
4.08
0.28
9.03
f1 (ppm)

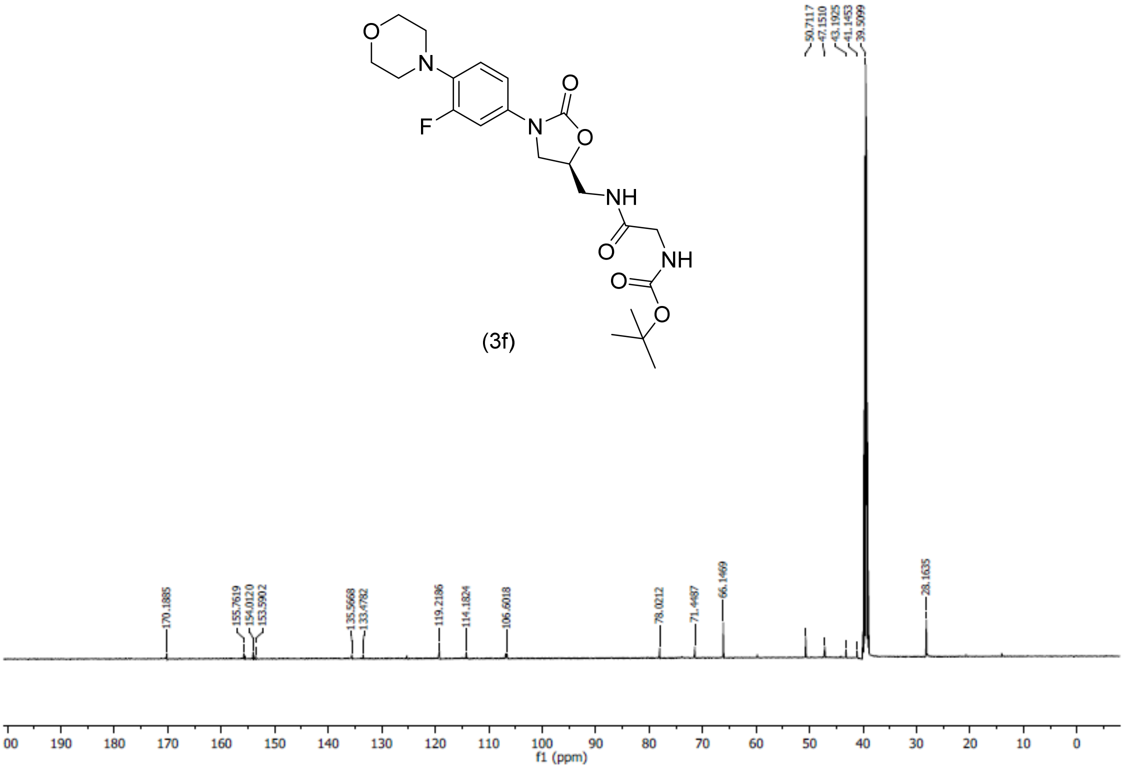
O
N
F
N
O
O
NH
O
NH
O
O
(3f)
170.1885
155.7619
154.0120
153.5902
135.5668
133.4782
119.2186
114.1824
106.6018
78.0212
71.4487
66.1469
50.7117
47.1510
43.1925
41.1453
39.5099
28.1635
f1 (ppm)

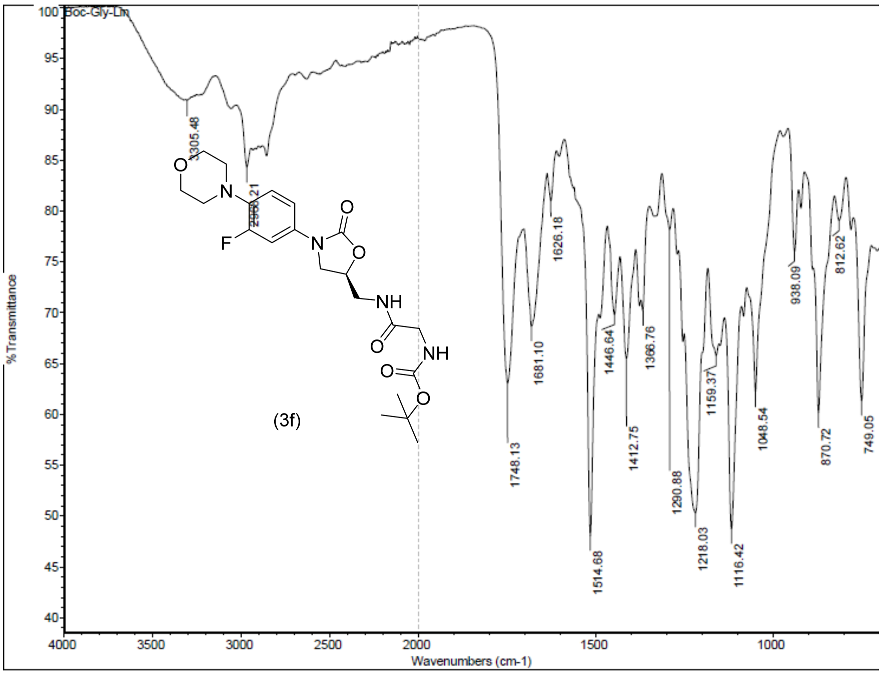
Boc-Gly-Lin
%Transmittance
Wavenumbers (cm-1)
3305.48
2958.21
1748.13
1681.10
1626.18
1514.68
1446.64
1412.75
1366.76
1290.88
1218.03
1159.37
1116.42
1048.54
938.09
870.72
812.62
749.05
O
N
F
NH
NH
(3f)

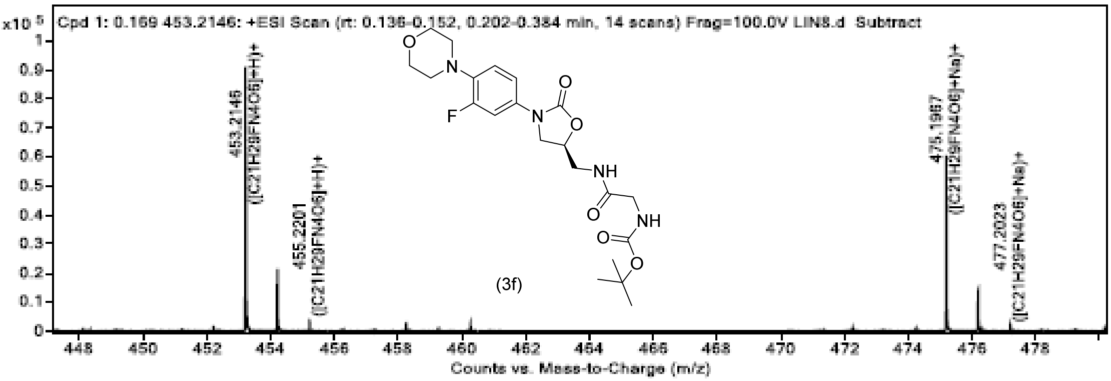
Cpd 1: 0.169 453.2146: +ESI Scan (rt: 0.136-0.152, 0.202-0.384 min, 14 scans) Frag=100.0V LIN8.d Subtract
x10 5
453.2146
([C21H29FN4O6]+H)+
455.2201
([C21H29FN4O6]+H)+
475.1967
([C21H29FN4O6]+Na)+
477.2023
([C21H29FN4O6]+Na)+
Counts vs. Mass-to-Charge (m/z)
(3f)

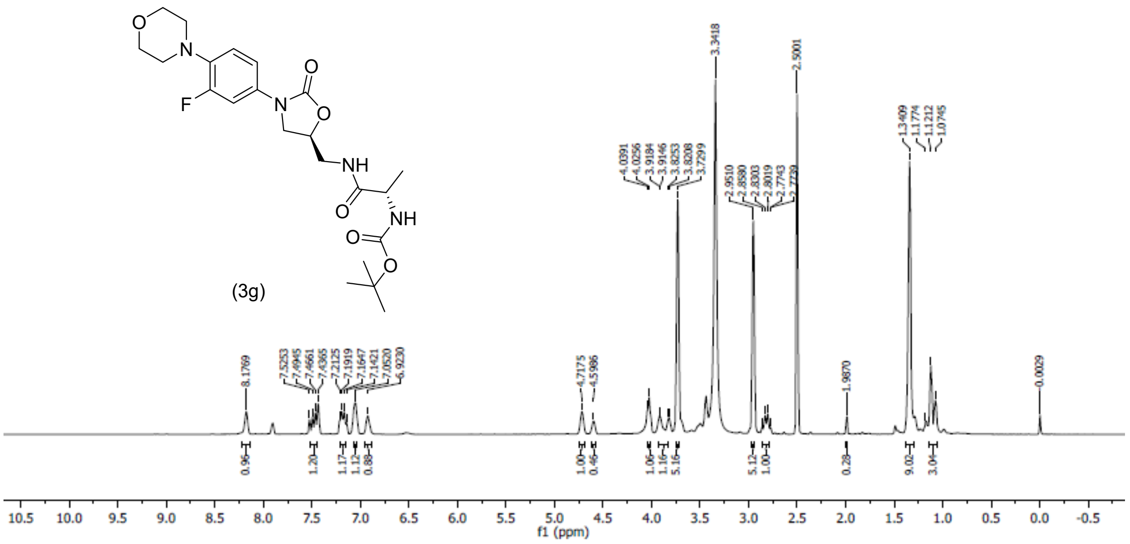

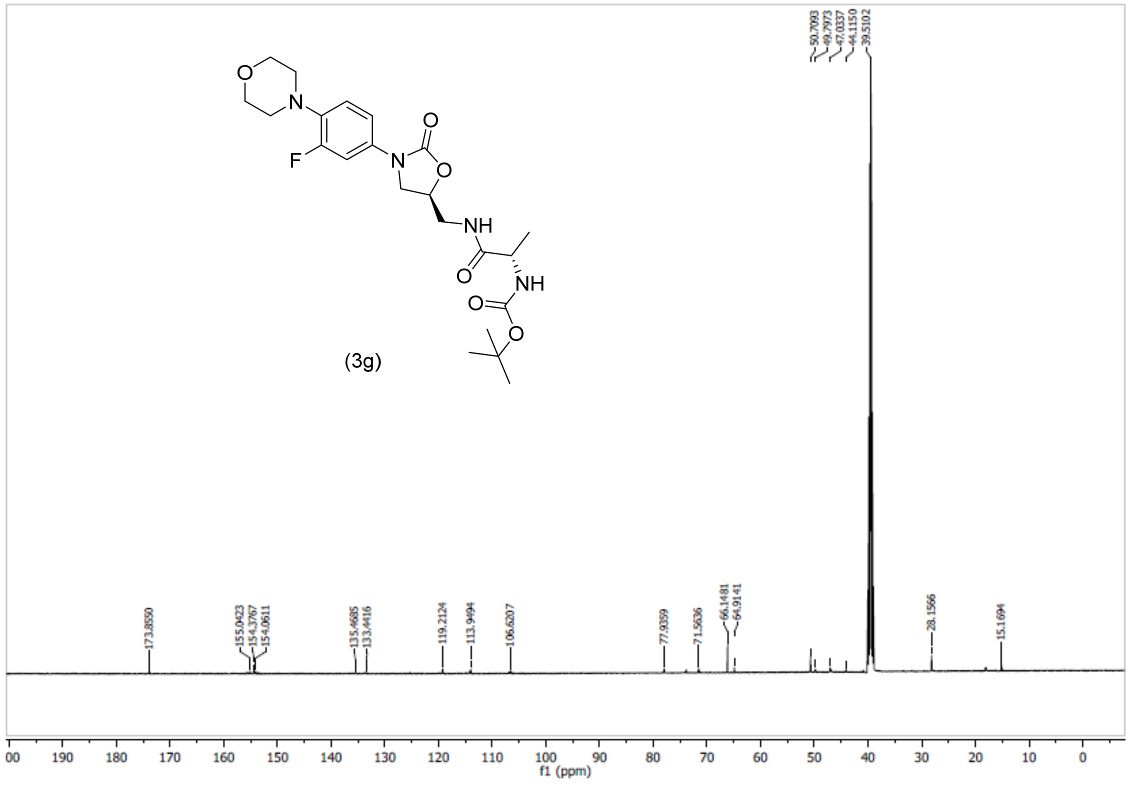

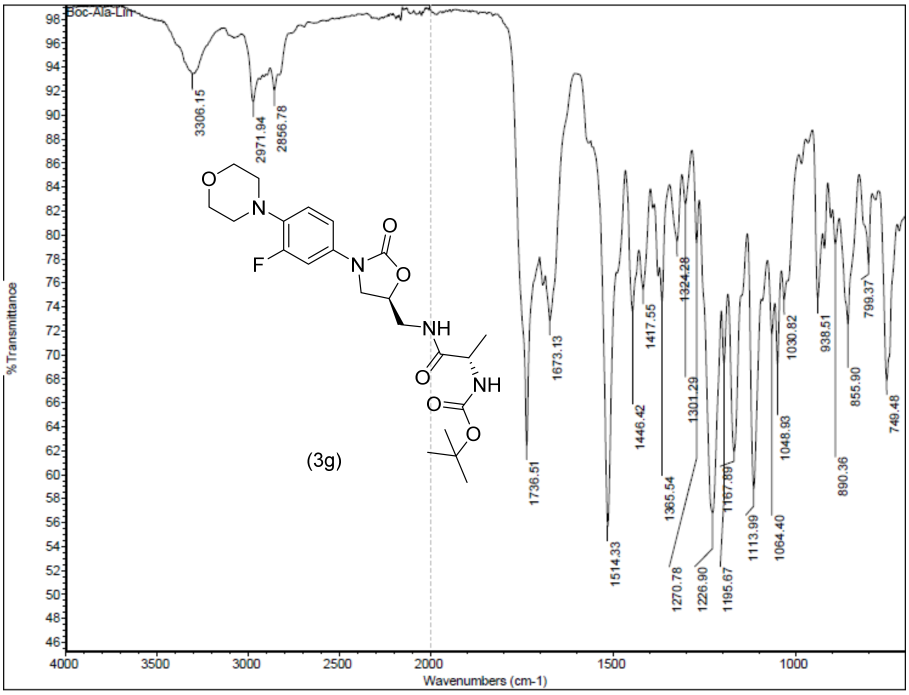
Boc-Ala-Lin
%Transmittance
Wavenumbers (cm-1)
3306.15
2971.94
2856.78
1736.51
1673.13
1514.33
1446.42
1417.55
1365.54
1324.28
1301.29
1270.78
1226.90
1195.67
1167.89
1113.99
1064.40
1048.93
1030.82
938.51
890.36
855.90
799.37
749.48
O
N
F
N
O
O
NH
O
NH
O
O

(3g)

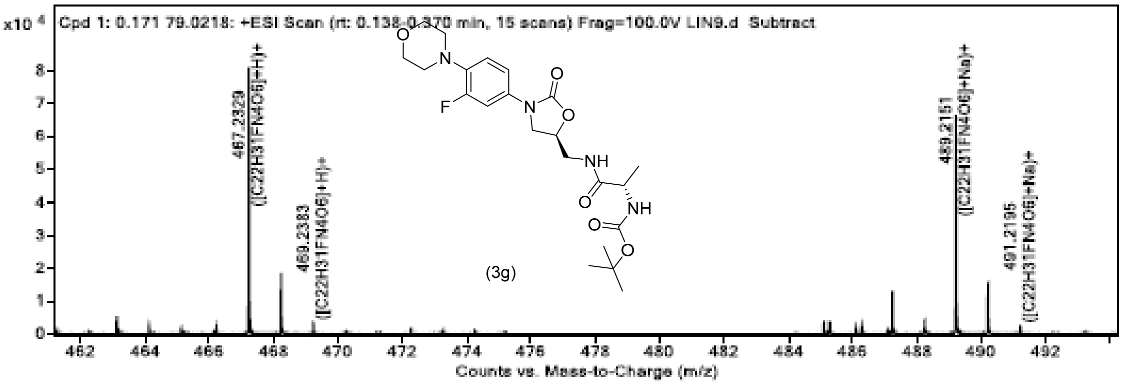
Cpd 1: 0.171 79.0218: +ESI Scan (rt: 0.138-0.370 min, 15 scans) Frag=100.0V LIN9.d Subtract
x10 4
467.2329
([C22H31FN4O6]+H)+
469.2383
([C22H31FN4O6]+H)+
489.2151
([C22H31FN4O6]+Na)+
491.2195
([C22H31FN4O6]+Na)+
Counts vs. Mass-to-Charge (m/z)

(3g)

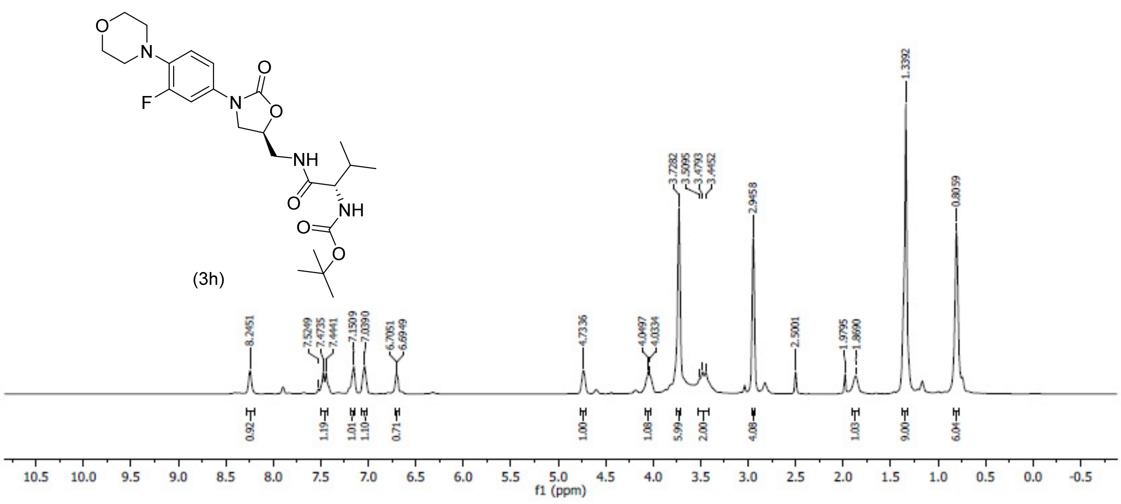

O
N
F
N
O
O
NH
O
NH
O
O
(3h)
8.2451
7.5249
7.4735
7.4441
7.1509
7.0390
6.7051
6.6949
4.7336
4.0497
4.0334
3.7282
3.5095
3.4793
3.4452
2.9458
2.5001
1.9795
1.8690
1.3392
0.8059
0.92
1.19
1.01
1.10
0.71
1.00
1.08
5.99
2.00
4.08
1.03
9.00
6.04
f1 (ppm)

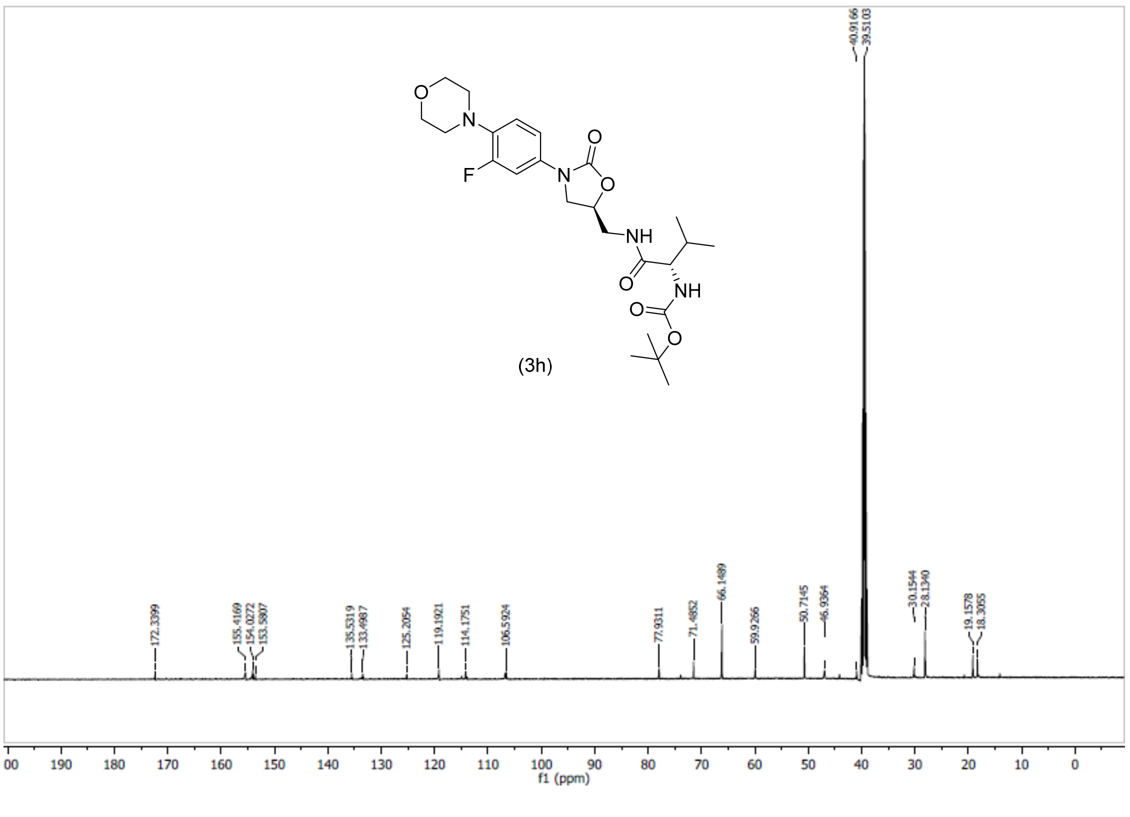

(3h)
172.3399
155.4169
154.0272
153.5807
135.5319
133.4987
125.2054
119.1921
114.1751
106.5924
77.9311
71.4852
66.1489
59.9266
50.7145
46.9364
40.9166
39.5103
30.1544
28.1340
19.1578
18.3055
f1 (ppm)

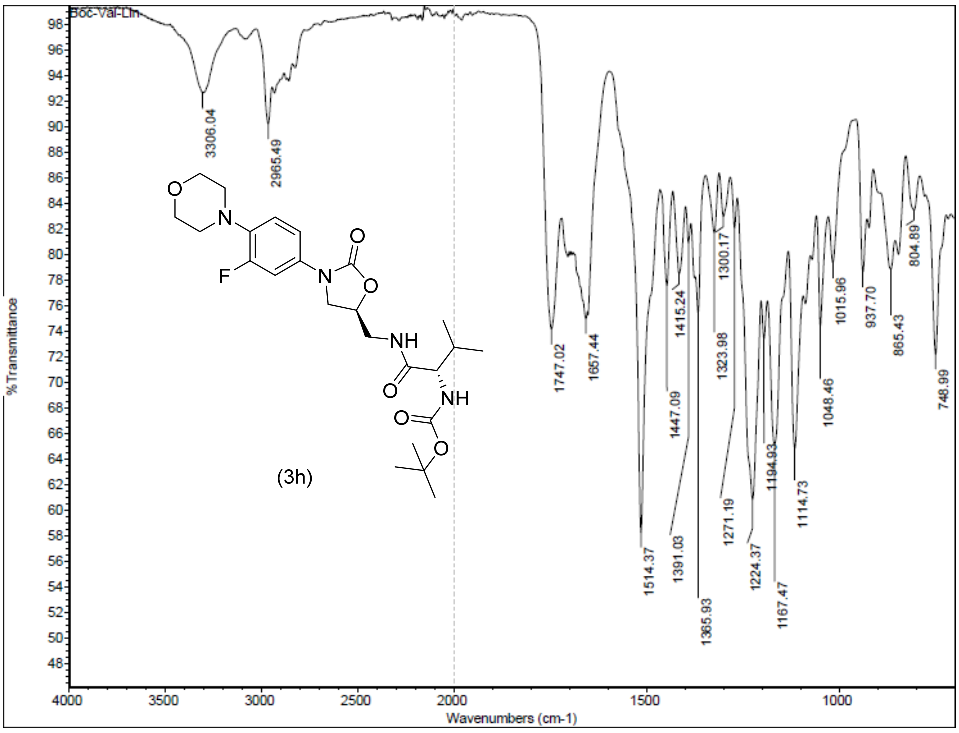
Boc-Val-Lin
% Transmittance
Wavenumbers (cm-1)
3306.04
2965.49
1747.02
1657.44
1514.37
1447.09
1415.24
1391.03
1365.93
1323.98
1300.17
1271.19
1224.37
1194.93
1167.47
1114.73
1048.46
1015.96
937.70
865.43
804.89
748.99
(3h)

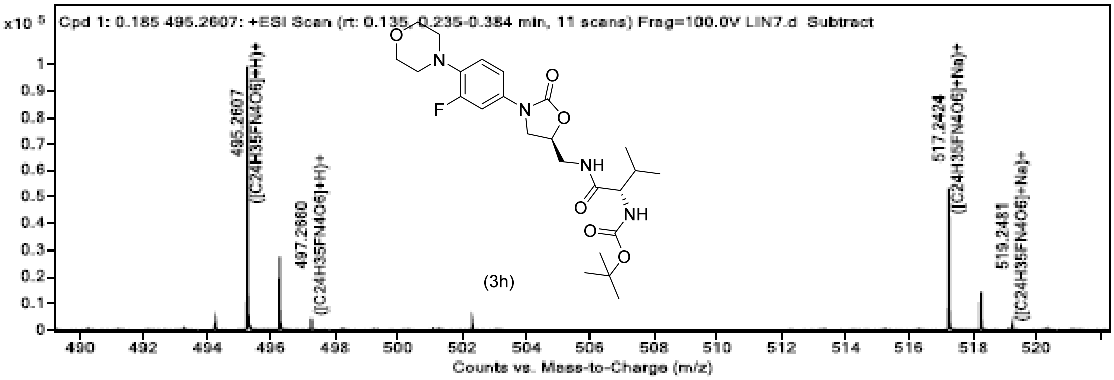
Cpd 1: 0.185 495.2607: +ESI Scan (rt: 0.135, 0.235-0.384 min, 11 scans) Frag=100.0V LIN7.d Subtract
495.2607
([C24H35FN4O6]+H)+
497.2680
([C24H35FN4O6]+H)+
517.2424
([C24H35FN4O6]+Na)+
519.2481
([C24H35FN4O6]+Na)+
(3h)
Counts vs. Mass-to-Charge (m/z)

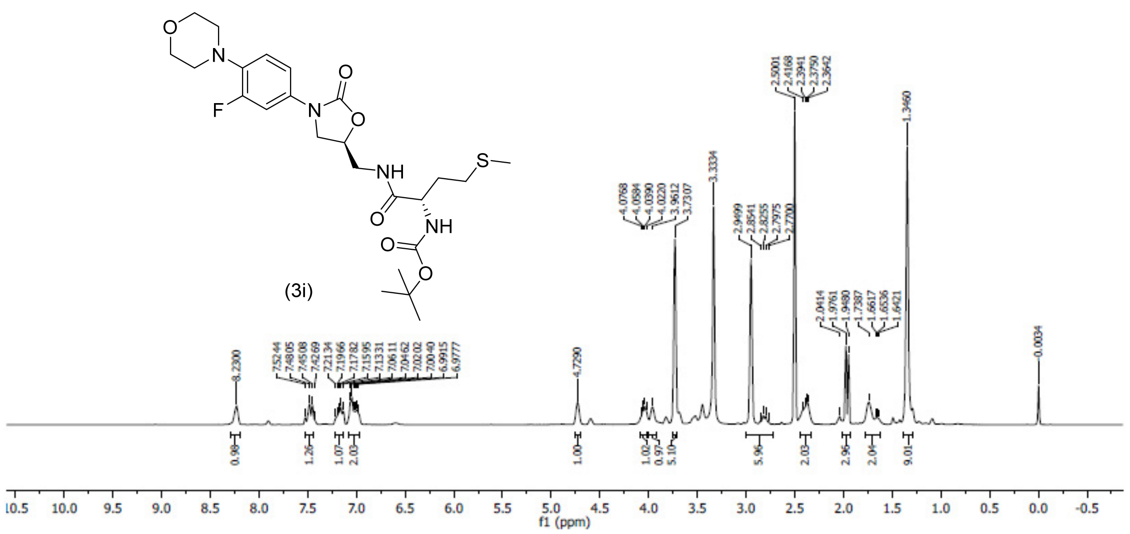
(3i)
f1 (ppm)

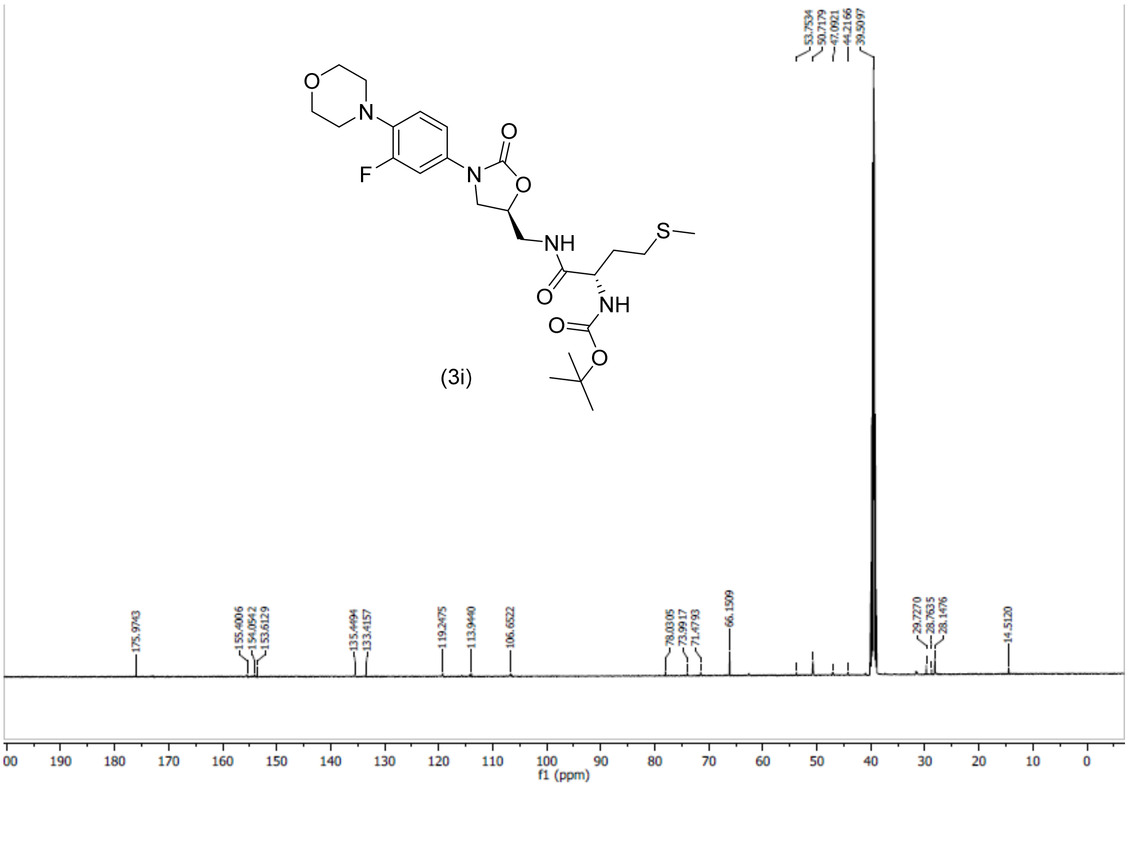
(3i)
f1 (ppm)

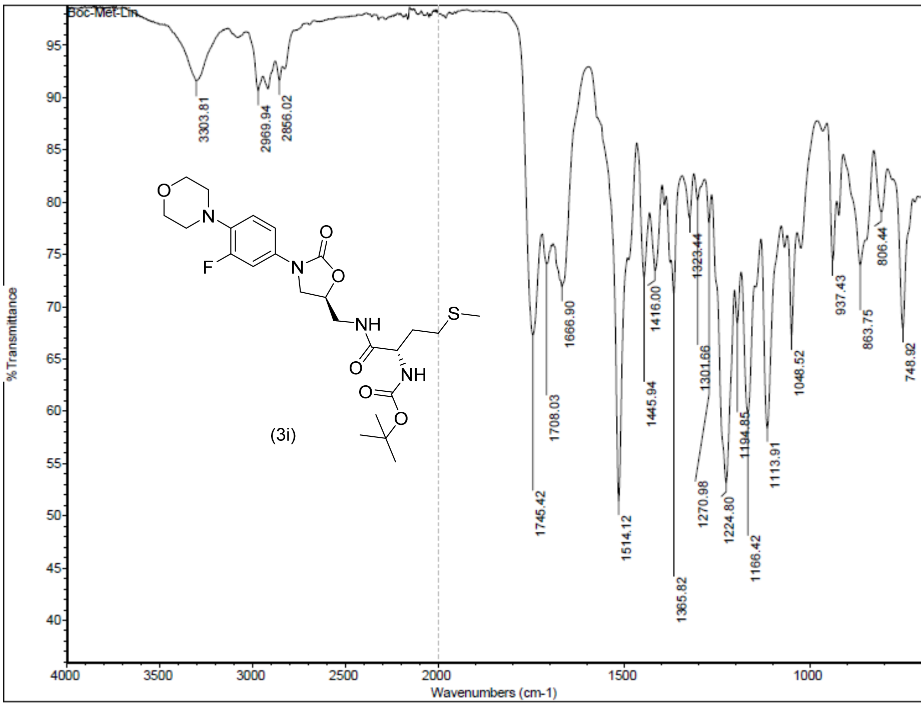
Boc-Met-Lin
%Transmittance
Wavenumbers (cm-1)
3303.81
2969.94
2856.02
1745.42
1708.03
1666.90
1514.12
1445.94
1416.00
1365.82
1323.44
1301.66
1270.98
1224.80
1194.85
1166.42
1113.91
1048.52
937.43
863.75
806.44
748.92
(3i)

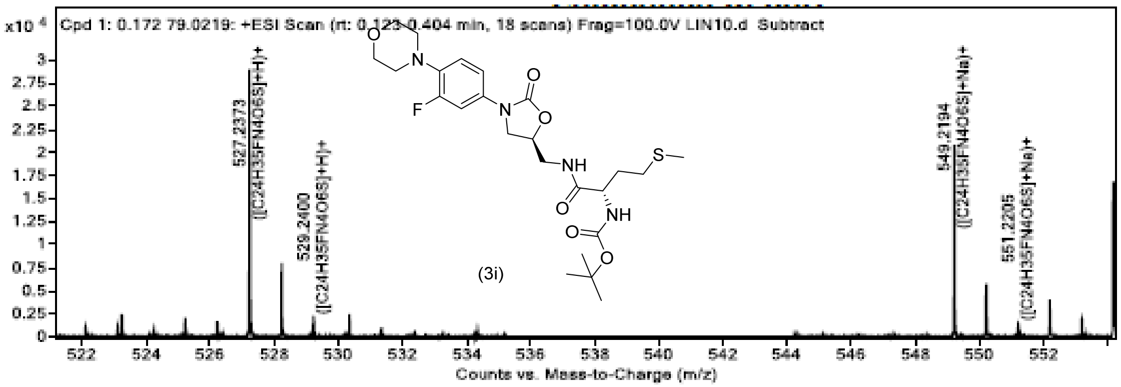
Cpd 1: 0.172 79.0219: +ESI Scan (rt: 0.123-0.404 min, 18 scans) Frag=100.0V LIN10.d Subtract
527.2373 ([C24H35FN4O6S]+H)+
529.2400 ([C24H35FN4O6S]+H)+
549.2194 ([C24H35FN4O6S]+Na)+
551.2205 ([C24H35FN4O6S]+Na)+
Counts vs. Mass-to-Charge (m/z)
(3i)

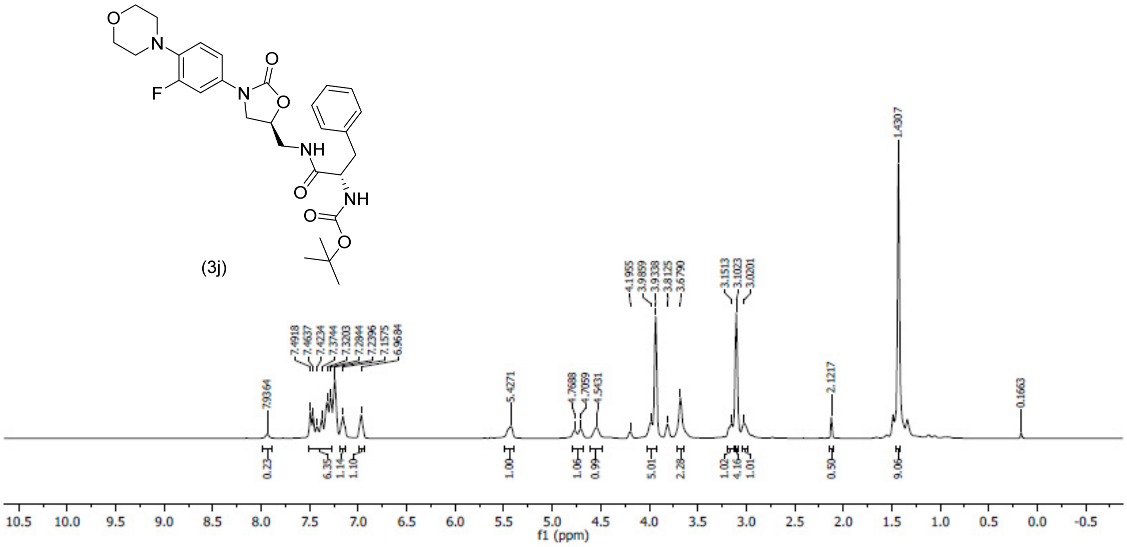
(3j)
f1 (ppm)

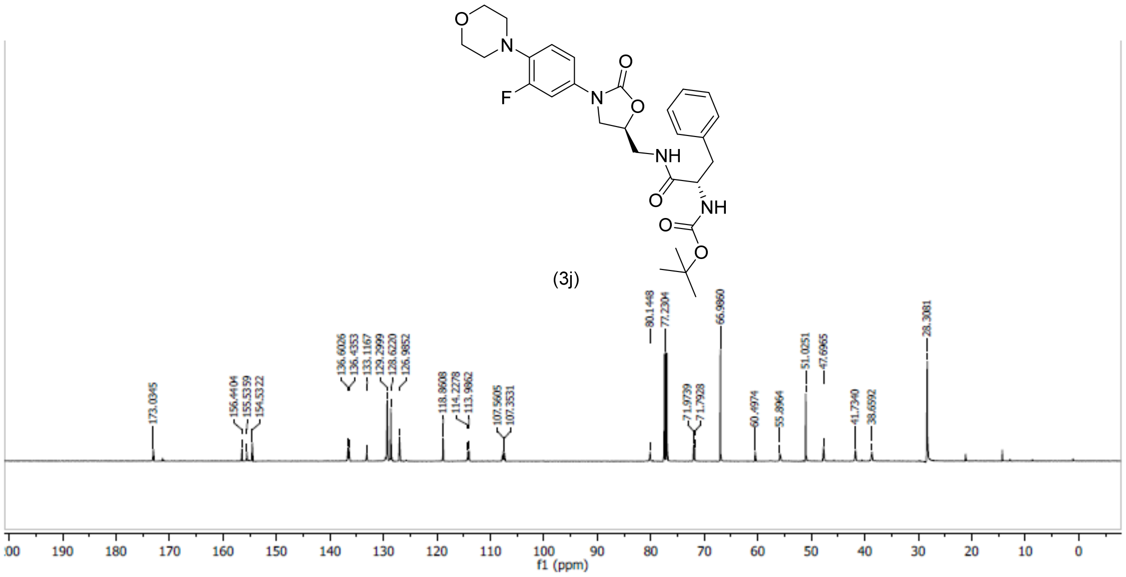
(3j)
f1 (ppm)

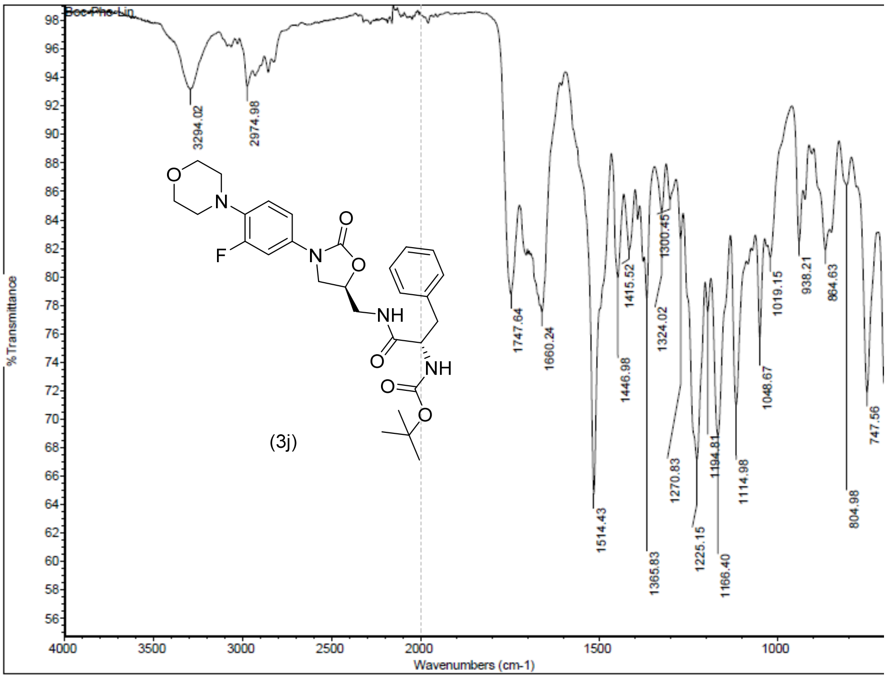
Boc-Phe-Lin
%Transmittance
Wavenumbers (cm-1)
3294.02
2974.98
1747.64
1660.24
1514.43
1446.98
1415.52
1365.83
1324.02
1300.45
1270.83
1225.15
1194.81
1166.40
1114.98
1048.67
1019.15
938.21
864.63
804.98
747.56
O
N
F
N
O
O
NH
O
NH
O
O
(3j)

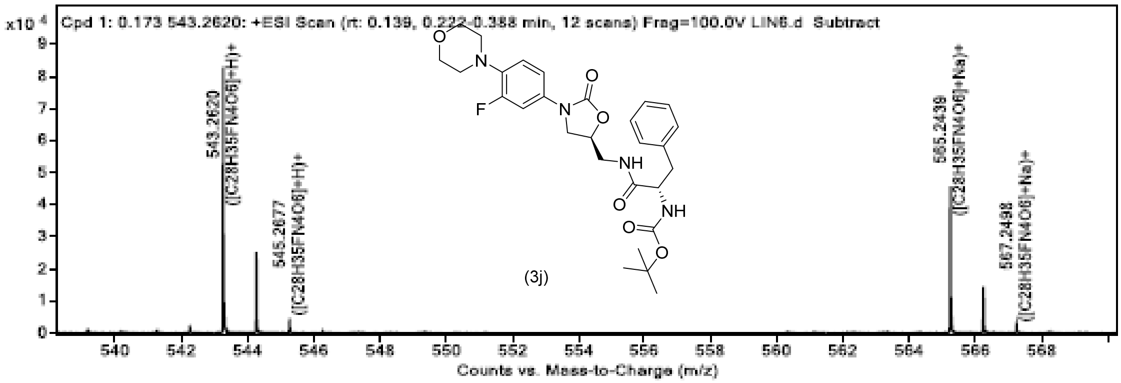
x10 4
Cpd 1: 0.173 543.2620: +ESI Scan (rt: 0.139, 0.222-0.388 min, 12 scans) Frag=100.0V LIN6.d Subtract
543.2620
([C28H35FN4O6]+H)+
545.2677
([C28H35FN4O6]+H)+
565.2439
([C28H35FN4O6]+Na)+
567.2498
([C28H35FN4O6]+Na)+
Counts vs. Mass-to-Charge (m/z)
(3j)

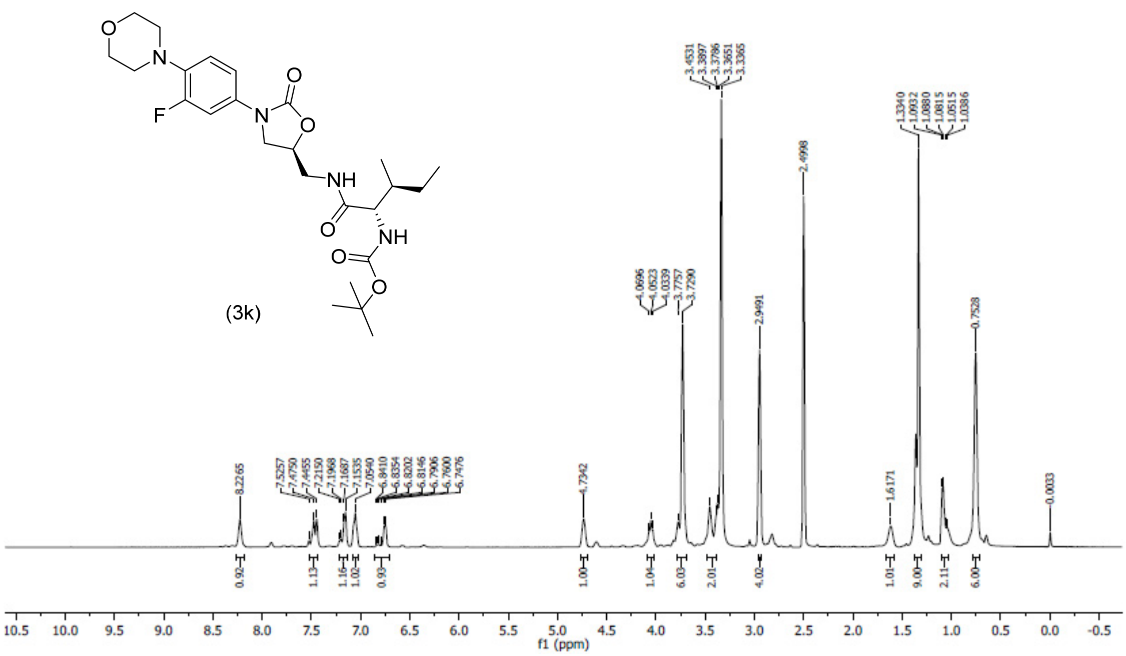

(3k)
f1 (ppm)

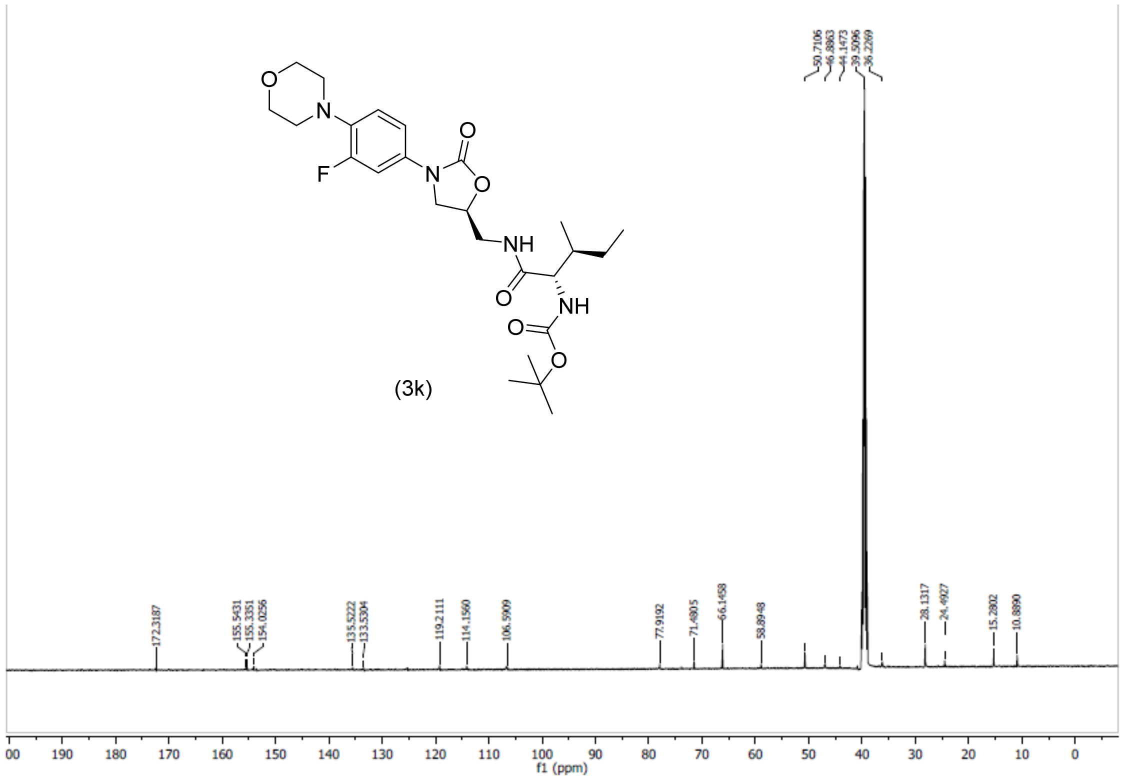

(3k)
f1 (ppm)

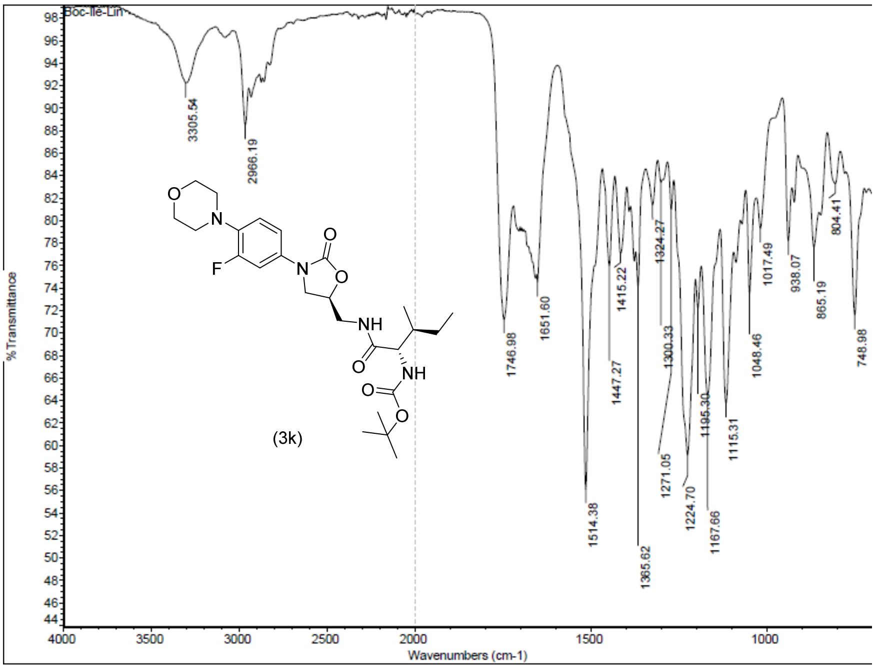

Boc-Ile-Lin
% Transmittance
Wavenumbers (cm-1)
3305.54
2966.19
1746.98
1651.60
1514.38
1447.27
1415.22
1365.62
1324.27
1300.33
1271.05
1224.70
1195.30
1167.66
1115.31
1048.46
1017.49
938.07
865.19
804.41
748.98
(3k)

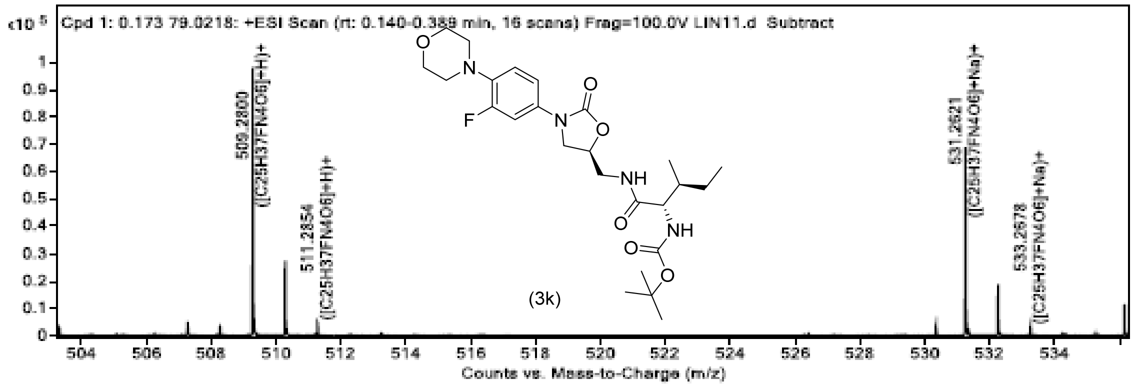

Cpd 1: 0.173 79.0218: +ESI Scan (rt: 0.140-0.389 min, 16 scans) Frag=100.0V LIN11.d Subtract
509.2800 ([C25H37FN4O6]+H)+
511.2854 ([C25H37FN4O6]+H)+
531.2621 ([C25H37FN4O6]+Na)+
533.2678 ([C25H37FN4O6]+Na)+
(3k)
Counts vs. Mass-to-Charge (m/z)

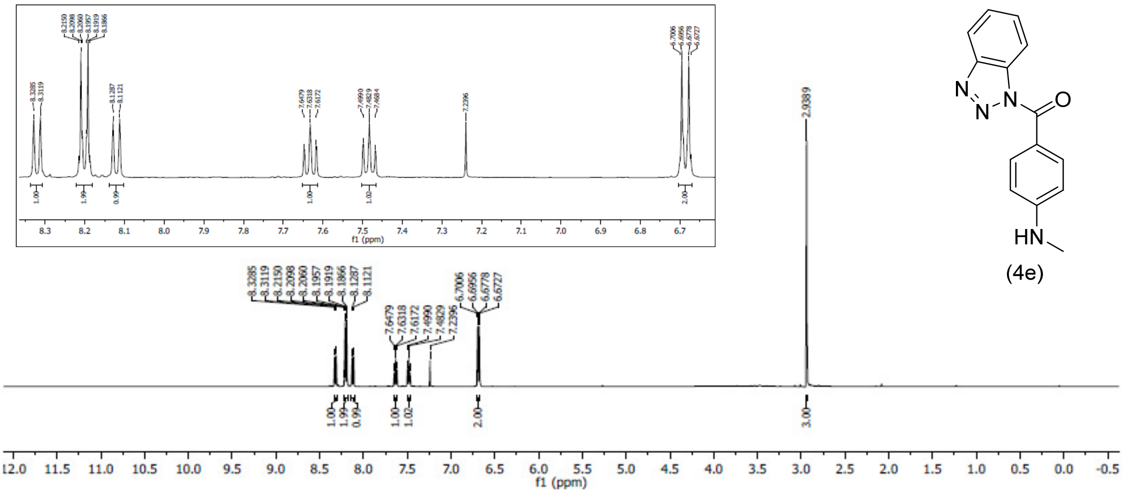
(4e)
f1 (ppm)

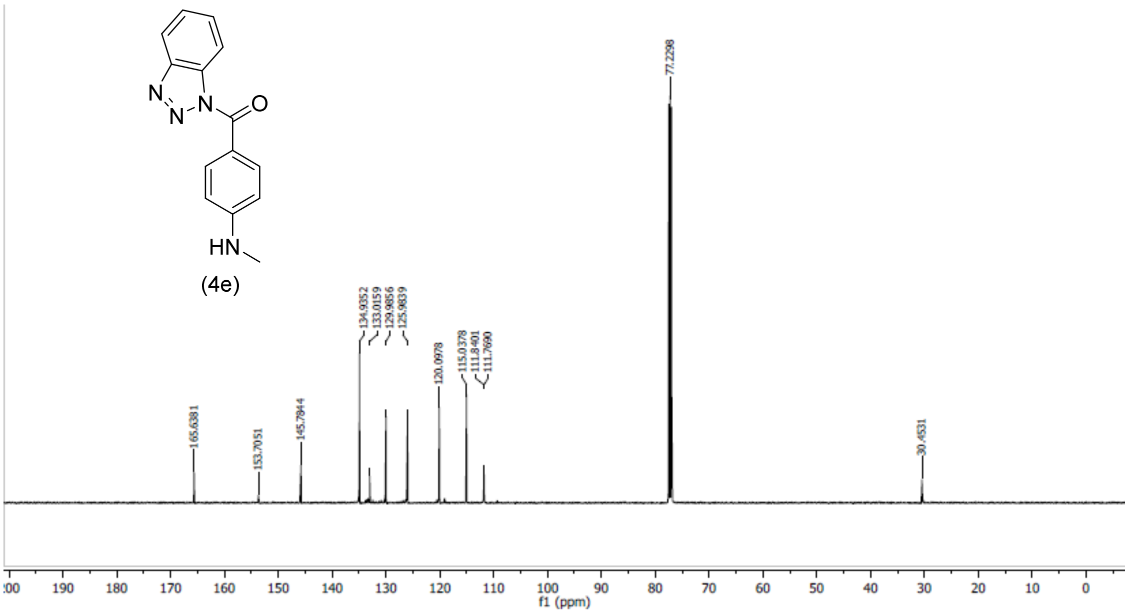
(4e)
f1 (ppm)

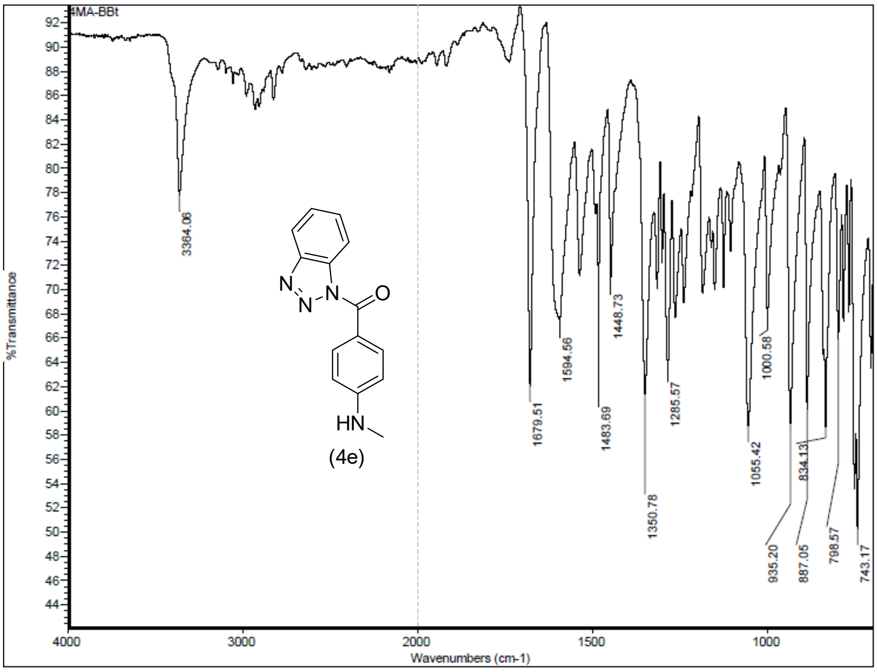
4MA-BBt
%Transmittance
Wavenumbers (cm-1)
3364.06
1679.51
1594.56
1483.69
1448.73
1350.78
1285.57
1055.42
1000.58
935.20
887.05
834.13
798.57
743.17
HN
(4e)

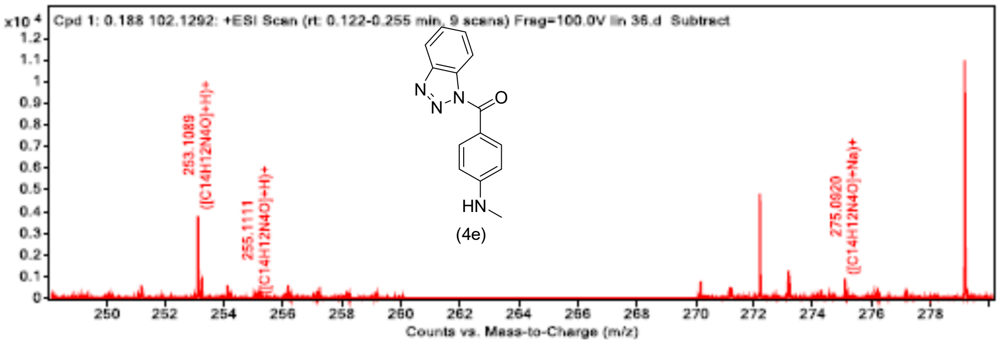
Cpd 1: 0.188 102.1292: +ESI Scan (rt: 0.122-0.255 min, 9 scans) Frag=100.0V lin 36.d Subtract
x10 4
253.1089
([C14H12N4O]+H)+
255.1111
([C14H12N4O]+H)+
275.0920
([C14H12N4O]+Na)+
HN
(4e)
Counts vs. Mass-to-Charge (m/z)

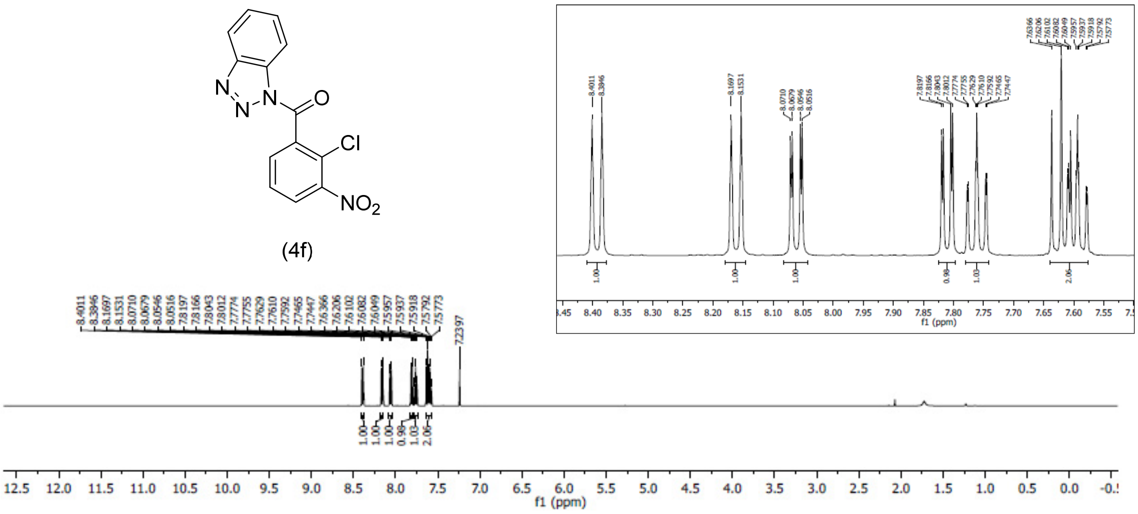
N
N
N
O
Cl
NO2
(4f)
8.4011
8.3946
8.1697
8.1531
8.0710
8.0679
8.0546
8.0516
7.8197
7.8166
7.8043
7.8012
7.7774
7.7755
7.7629
7.7610
7.7592
7.7465
7.7447
7.6366
7.6206
7.6102
7.6082
7.6049
7.5957
7.5937
7.5918
7.5792
7.5773
7.2397
1.00
1.00
1.00
0.98
1.03
2.06
f1 (ppm)

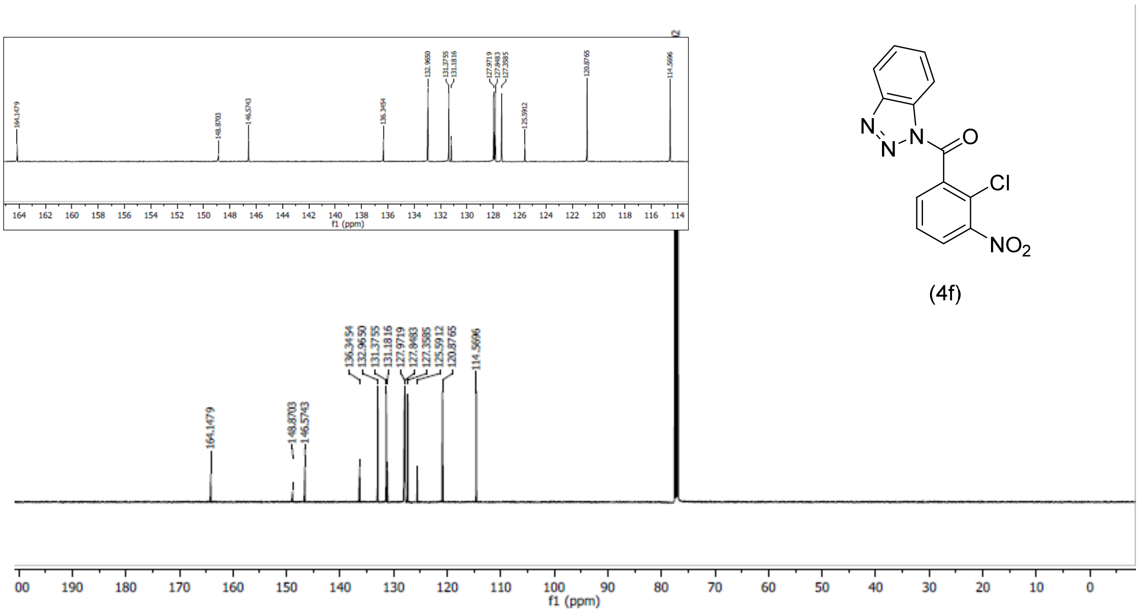
164.1479
148.8703
146.5743
136.3454
132.9650
131.3755
131.1816
127.9719
127.8483
127.3585
125.5912
120.8765
114.5696
f1 (ppm)
N
N
N
O
Cl
NO2
(4f)

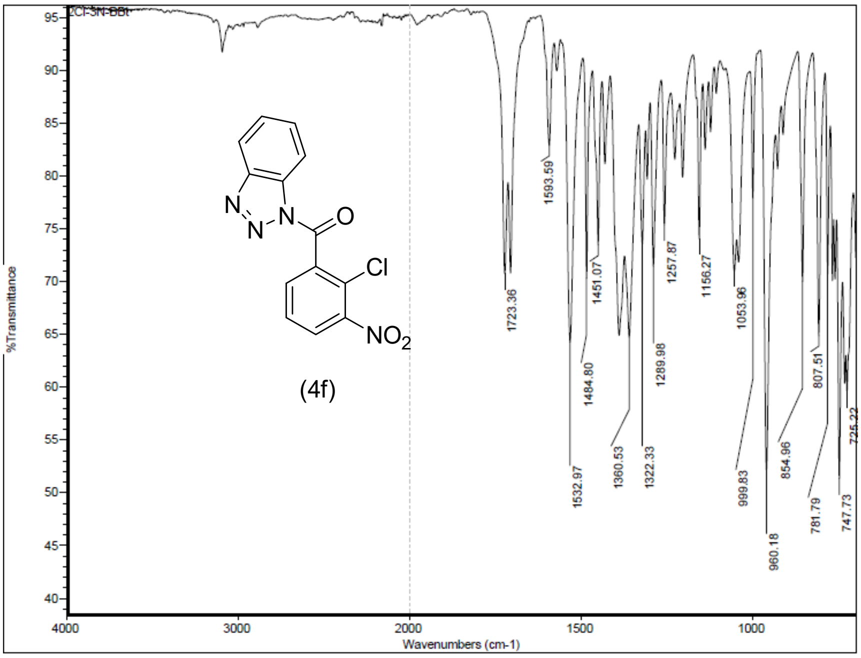
2Cl-3N-BBt
%Transmittance
Wavenumbers (cm-1)
(4f)
1723.36
1593.59
1532.97
1484.80
1451.07
1360.53
1322.33
1289.98
1257.87
1156.27
1053.96
999.83
960.18
854.96
807.51
781.79
747.73
725.22

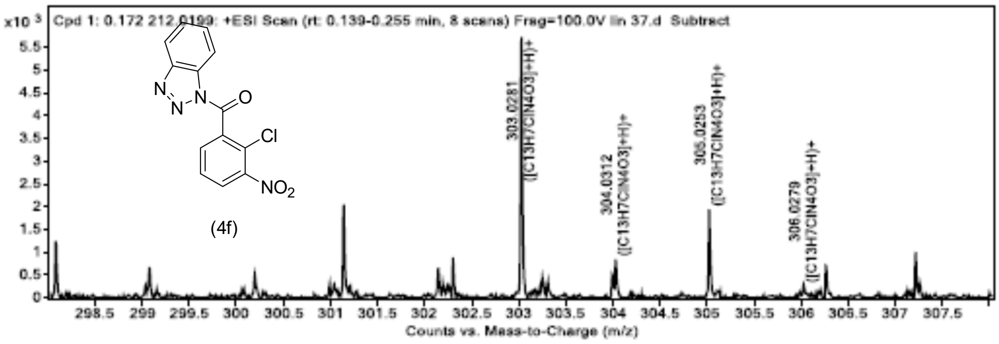
Cpd 1: 0.172 212.0199: +ESI Scan (rt: 0.139-0.255 min, 8 scans) Frag=100.0V lin 37.d Subtract
303.0281 ([C13H7ClN4O3]+H)+
304.0312 ([C13H7ClN4O3]+H)+
305.0253 ([C13H7ClN4O3]+H)+
306.0279 ([C13H7ClN4O3]+H)+
(4f)
Counts vs. Mass-to-Charge (m/z)

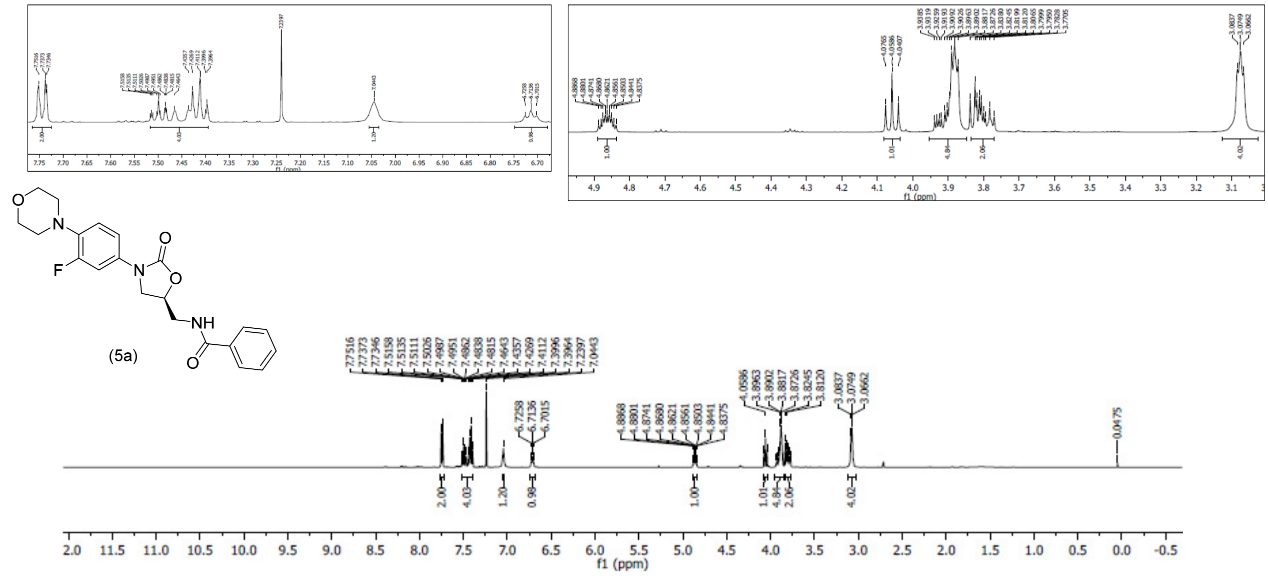

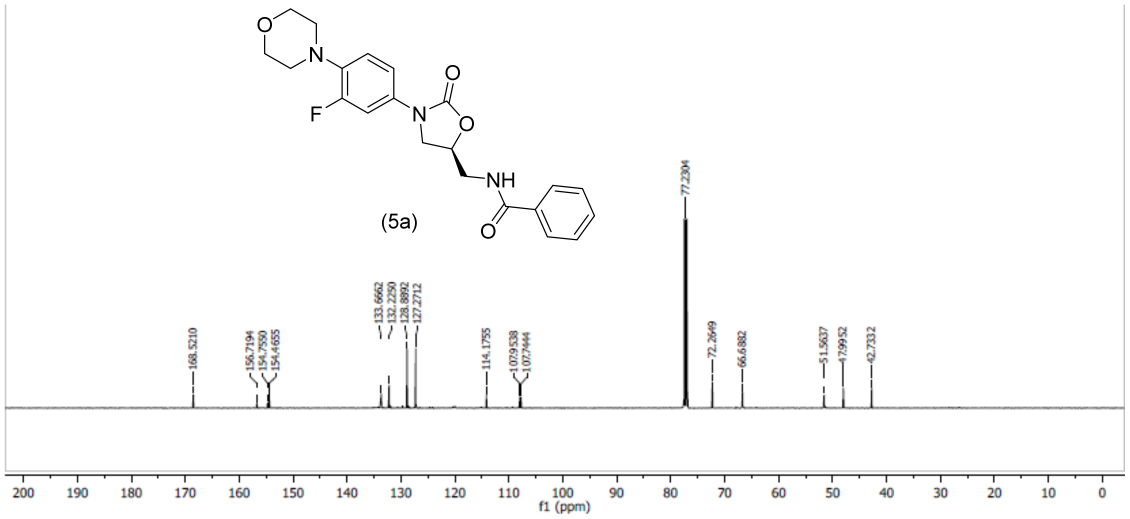

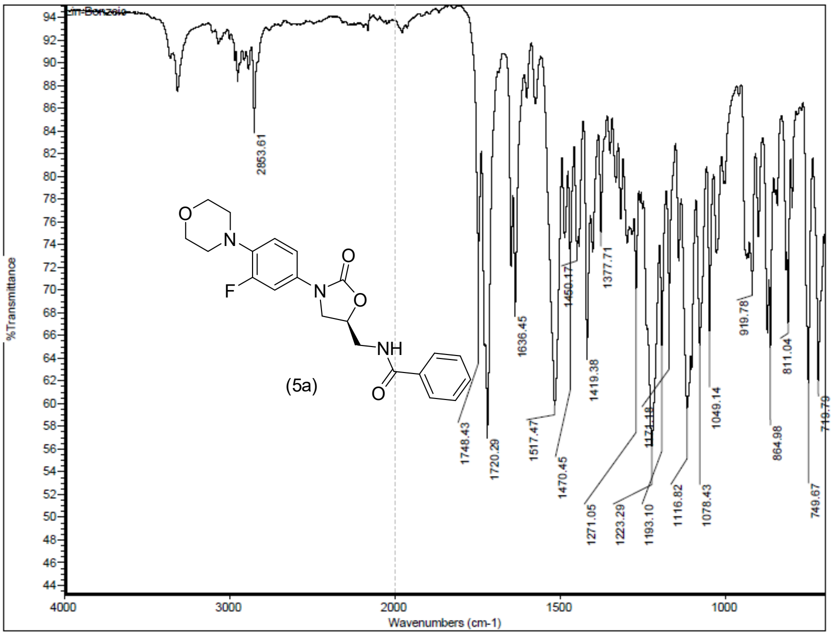

Lin-Benzoic
%Transmittance
Wavenumbers (cm-1)
2853.61
1748.43
1720.29
1636.45
1517.47
1470.45
1450.17
1419.38
1377.71
1271.05
1223.29
1193.10
1171.18
1116.82
1078.43
1049.14
919.78
864.98
811.04
749.67
719.79
O
N
F
O
N
O
NH
O
(5a)

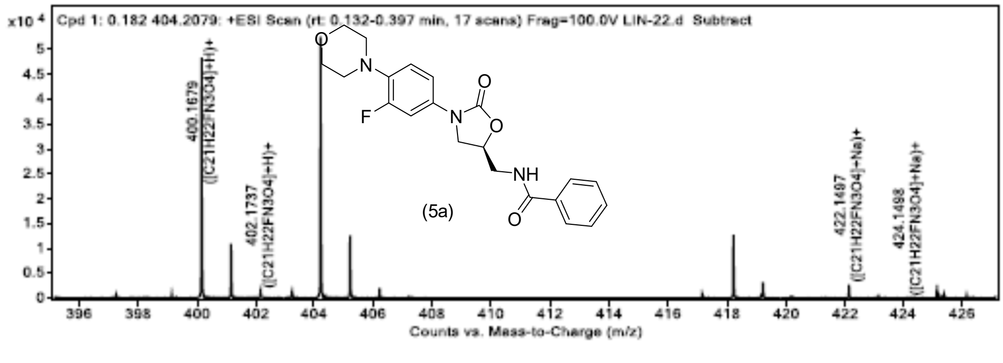

Cpd 1: 0.182 404.2079: +ESI Scan (rt: 0.132-0.397 min, 17 scans) Frag=100.0V LIN-22.d Subtract
x10 4
400.1679
([C21H22FN3O4]+H)+
402.1737
([C21H22FN3O4]+H)+
422.1497
([C21H22FN3O4]+Na)+
424.1498
([C21H22FN3O4]+Na)+
Counts vs. Mass-to-Charge (m/z)
O
N
F
O
N
O
NH
O
(5a)

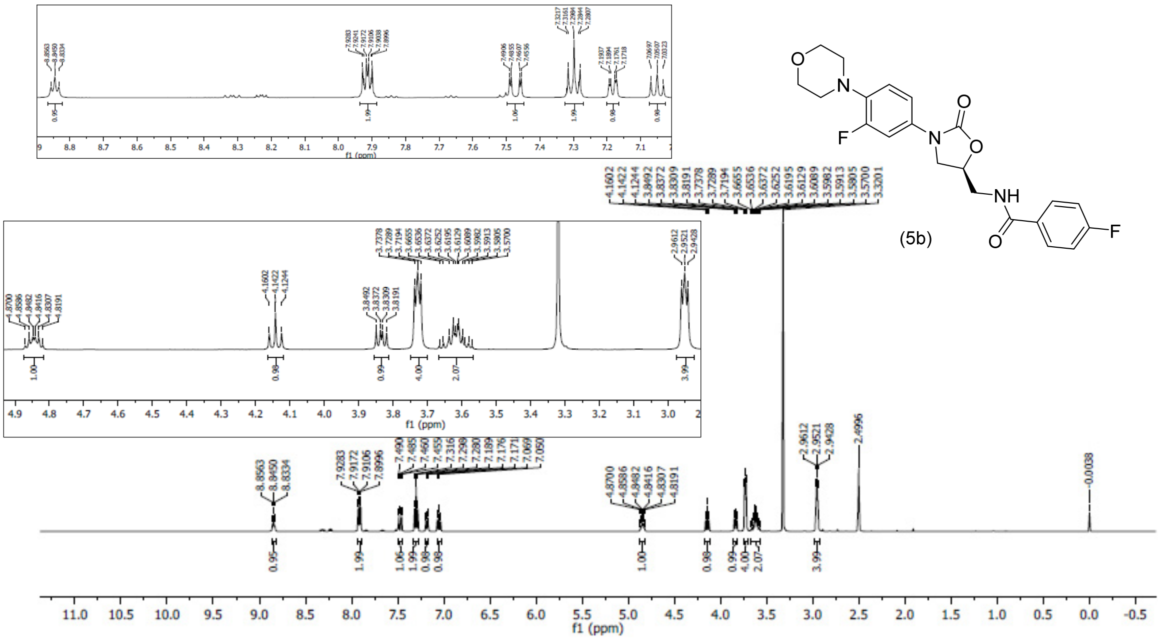

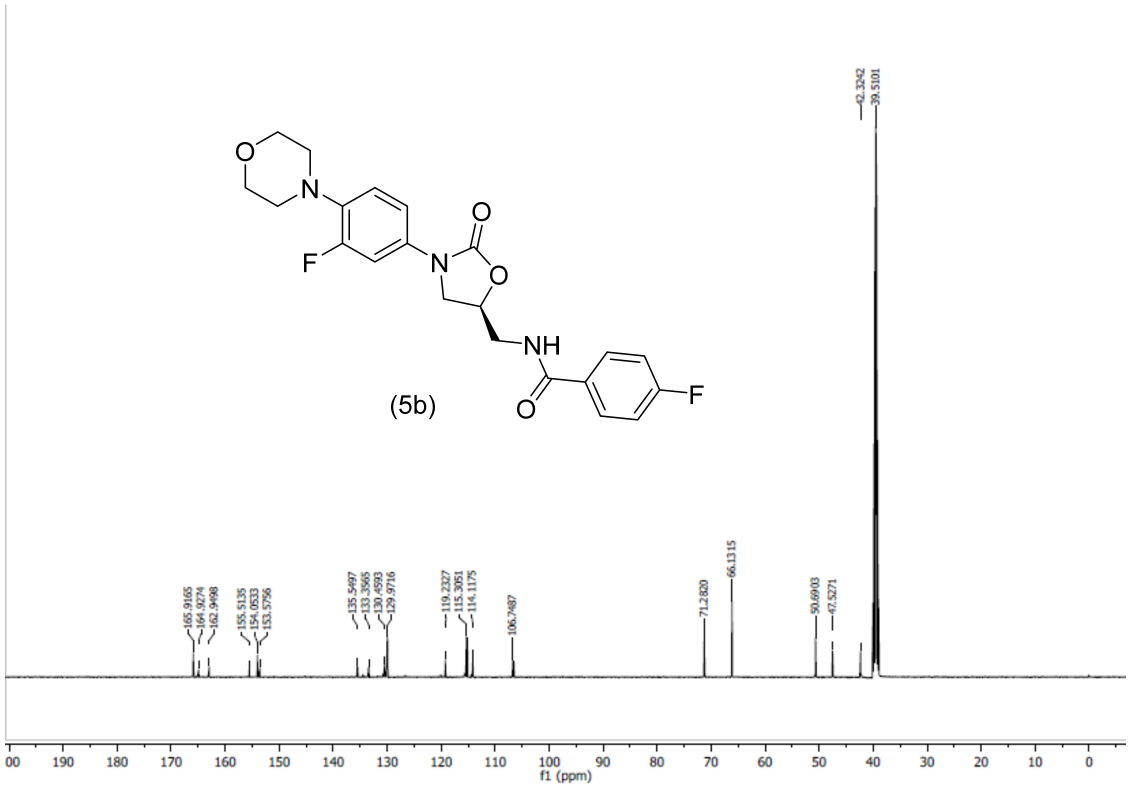

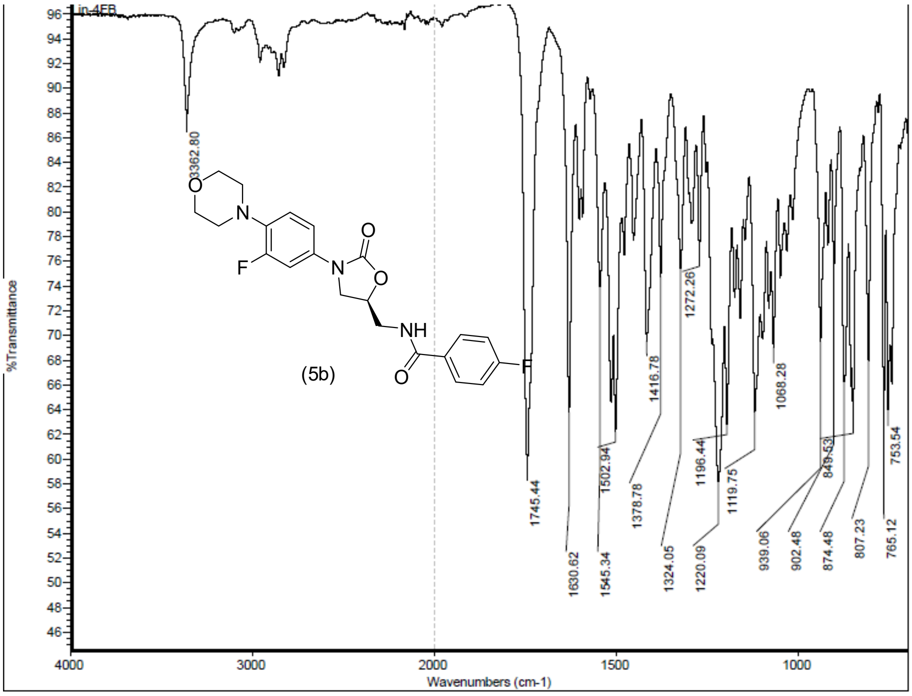

%Transmittance
Wavenumbers (cm-1)
(5b)
3362.80
1745.44
1630.62
1545.34
1502.94
1416.78
1378.78
1324.05
1272.26
1220.09
1196.44
1119.75
1068.28
939.06
902.48
874.48
849.53
807.23
765.12
753.54

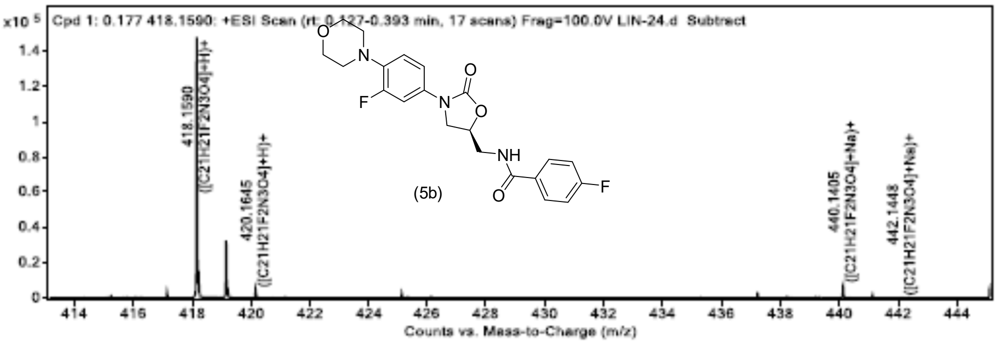

Cpd 1: 0.177 418.1590: +ESI Scan (rt: 0.127-0.393 min, 17 scans) Frag=100.0V LIN-24.d Subtract
x10 5
418.1590
([C21H21F2N3O4]+H)+
420.1645
([C21H21F2N3O4]+H)+
440.1405
([C21H21F2N3O4]+Na)+
442.1448
([C21H21F2N3O4]+Na)+
(5b)
Counts vs. Mass-to-Charge (m/z)

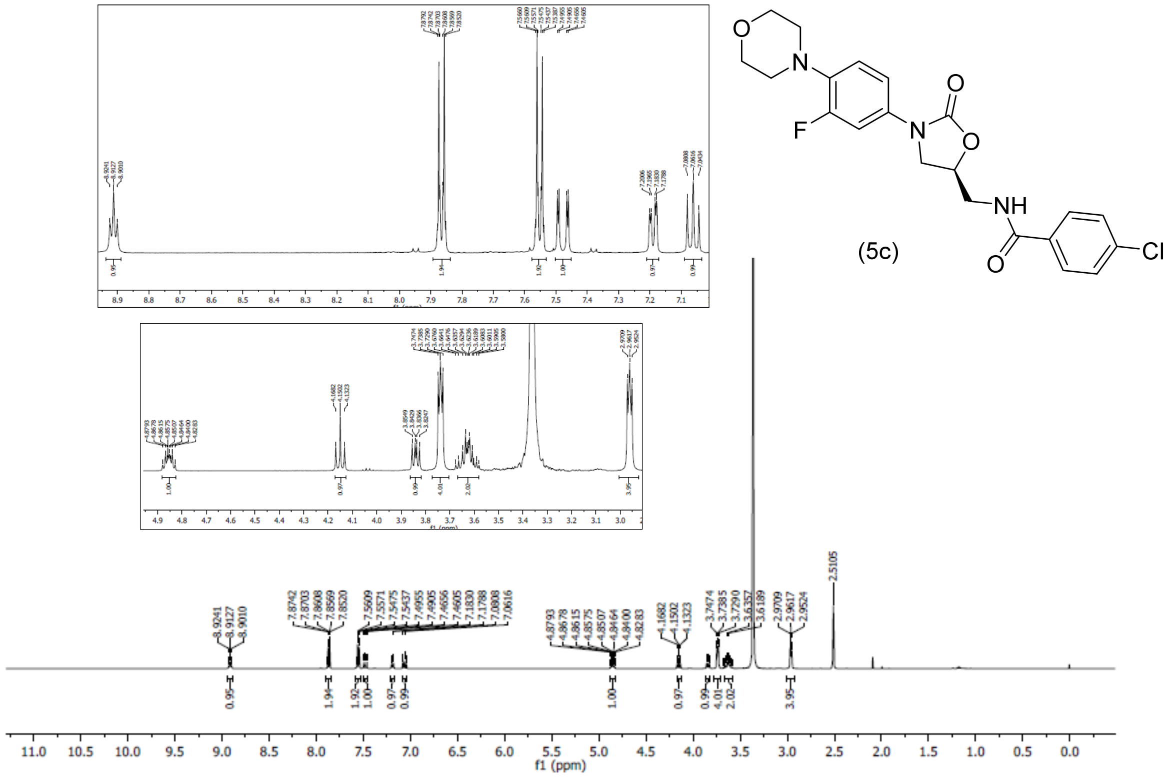
(5c)
f1 (ppm)

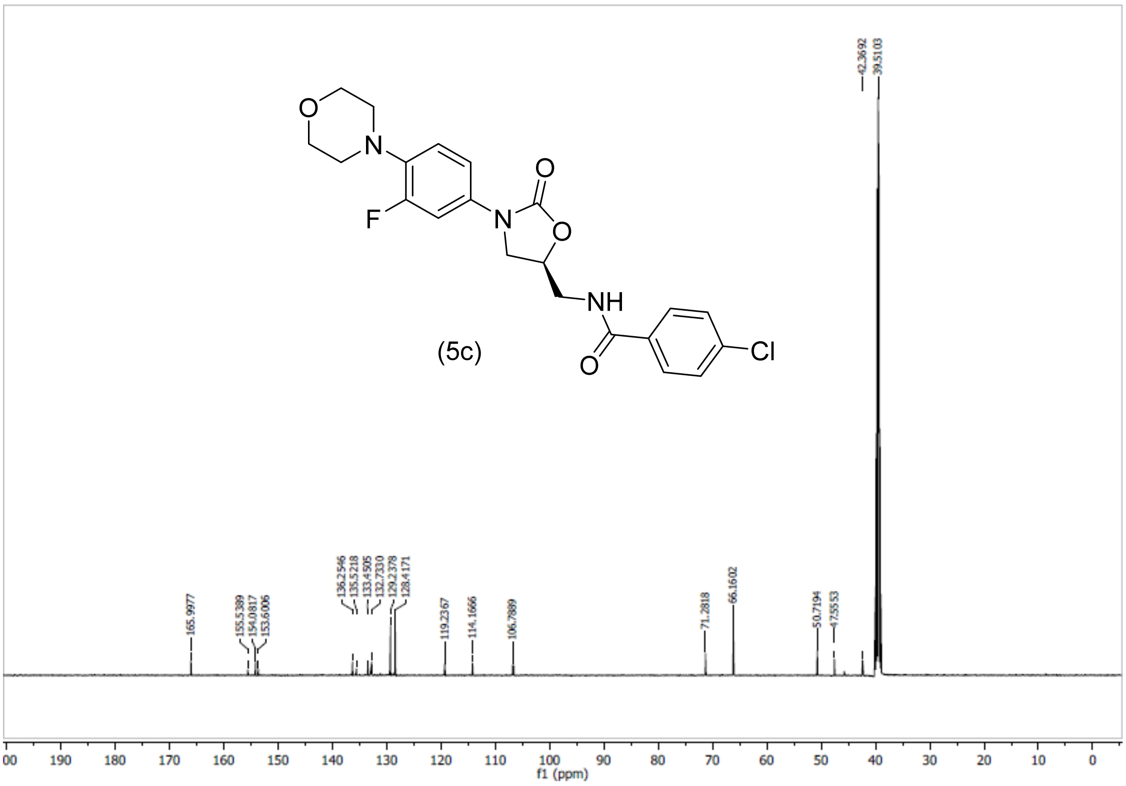
(5c)
f1 (ppm)

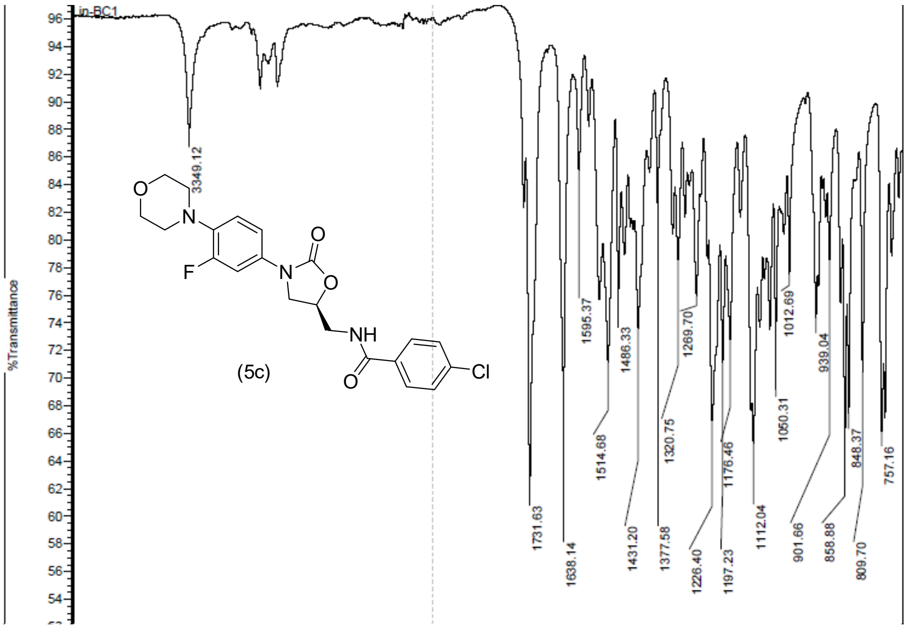

in-BC1
%Transmittance
3349.12
1731.63
1638.14
1595.37
1514.68
1486.33
1431.20
1377.58
1320.75
1269.70
1226.40
1197.23
1176.46
1112.04
1050.31
1012.69
939.04
901.66
858.88
848.37
809.70
757.16
O
N
F
N
O
O
NH
O
Cl
(5c)

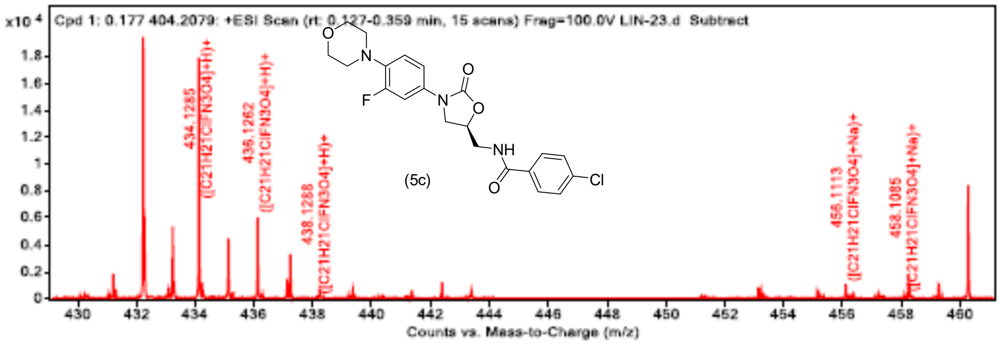

Cpd 1: 0.177 404.2079: +ESI Scan (rt: 0.127-0.359 min, 15 scans) Frag=100.0V LIN-23.d Subtract
x10 4
434.1285
([C21H21ClFN3O4]+H)+
436.1262
([C21H21ClFN3O4]+H)+
438.1288
([C21H21ClFN3O4]+H)+
456.1113
([C21H21ClFN3O4]+Na)+
458.1085
([C21H21ClFN3O4]+Na)+
(5c)
Counts vs. Mass-to-Charge (m/z)

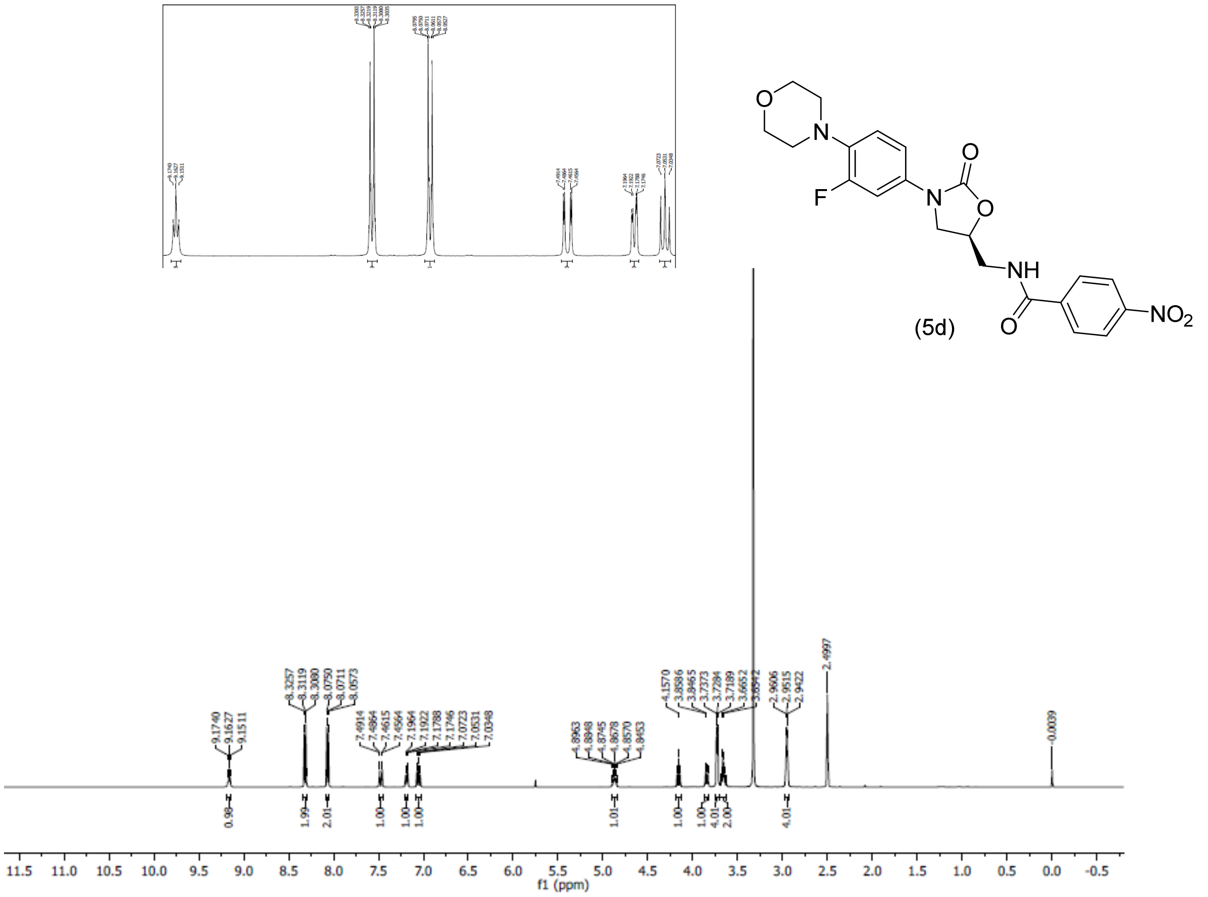
(5d)
NH
NO2
O
N
F
9.1740
9.1627
9.1511
8.3257
8.3119
8.3080
8.0750
8.0711
8.0573
7.4914
7.4864
7.4615
7.4564
7.1964
7.1922
7.1788
7.1746
7.0723
7.0531
7.0348
4.8963
4.8848
4.8745
4.8678
4.8570
4.8453
4.1570
3.8586
3.8465
3.7373
3.7284
3.7189
3.6652
3.6542
2.9606
2.9515
2.9422
2.4997
0.0039
0.98
1.99
2.01
1.00
1.00
1.00
1.01
1.00
1.00
4.01
2.00
4.01
f1 (ppm)

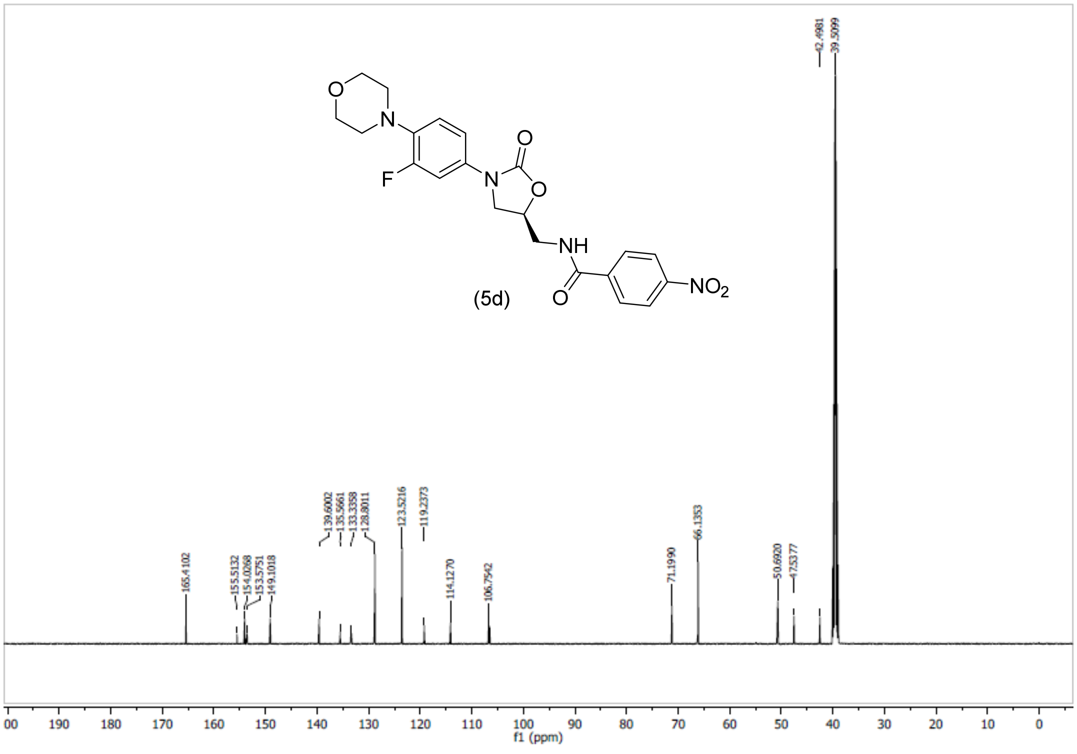
(5d)
NH
NO2
O
N
F
165.4102
155.5132
154.0268
153.5751
149.1018
139.6002
135.5661
133.3358
128.8011
123.5216
119.2373
114.1270
106.7542
71.1990
66.1353
50.6920
47.5377
42.4981
39.5099
f1 (ppm)

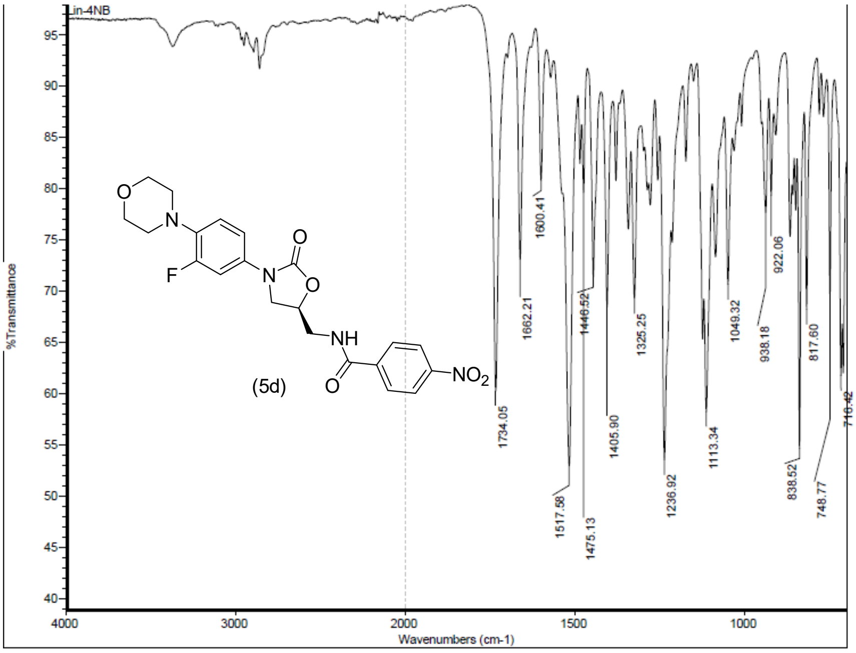
Lin-4NB
%Transmittance
Wavenumbers (cm-1)
(5d)
1734.05
1662.21
1600.41
1517.58
1475.13
1446.52
1405.90
1325.25
1236.92
1113.34
1049.32
938.18
922.06
838.52
817.60
748.77
716.42

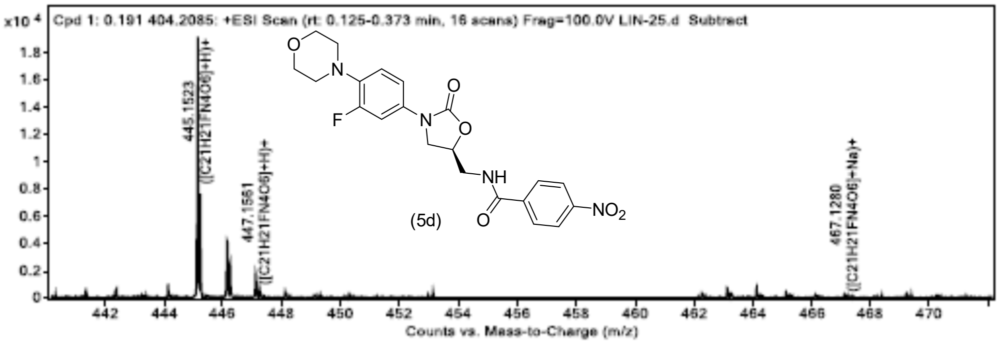
Cpd 1: 0.191 404.2085: +ESI Scan (rt: 0.125-0.373 min, 16 scans) Frag=100.0V LIN-25.d Subtract
445.1523
([C21H21FN4O6]+H)+
447.1561
([C21H21FN4O6]+H)+
467.1280
([C21H21FN4O6]+Na)+
(5d)
Counts vs. Mass-to-Charge (m/z)

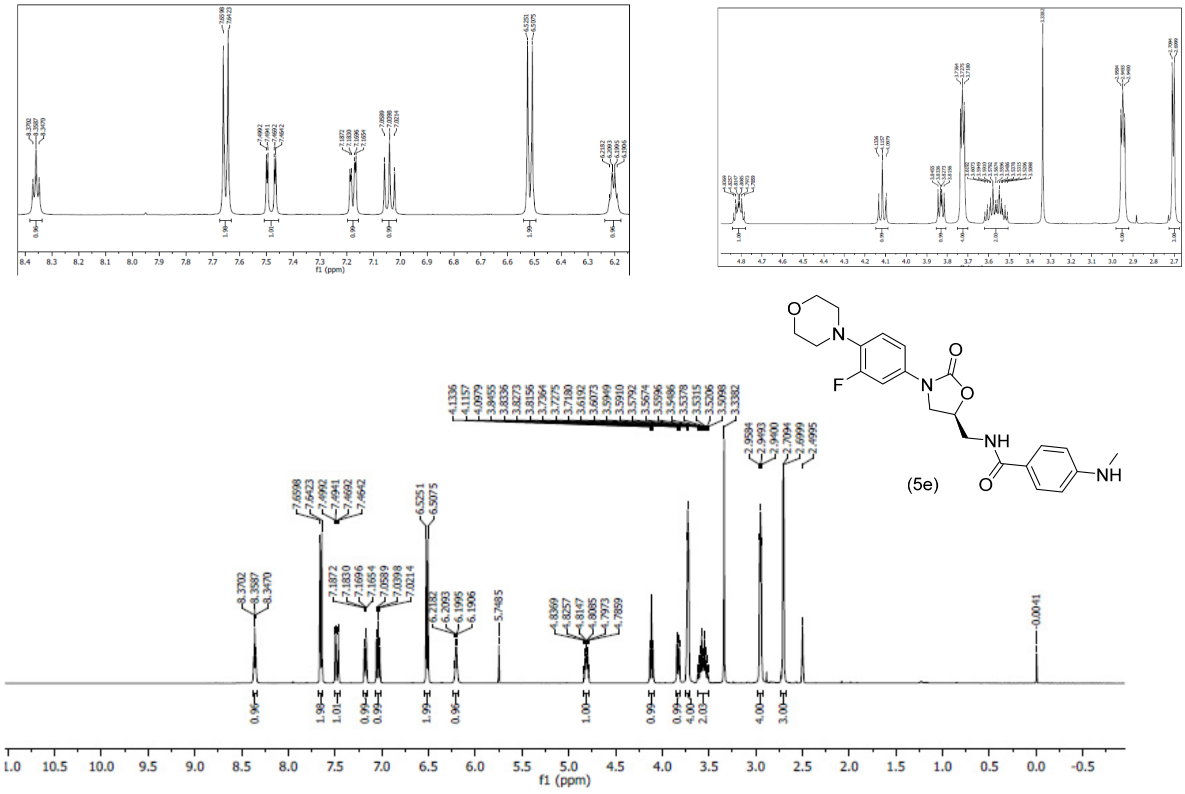
(5e)
O
N
F
NH
f1 (ppm)
8.3702
8.3587
8.3470
7.6598
7.6423
7.4992
7.4941
7.4692
7.4642
7.1872
7.1830
7.1696
7.1654
7.0589
7.0398
7.0214
6.5251
6.5075
6.2182
6.2093
6.1995
6.1906
5.7485
4.8369
4.8257
4.8147
4.8085
4.7973
4.7859
2.9584
2.9493
2.9400
2.7094
2.6999
2.4995
0.96
1.98
1.01
0.99
0.99
1.99
0.96
1.00
0.99
0.99
4.00
2.03
4.00
3.00

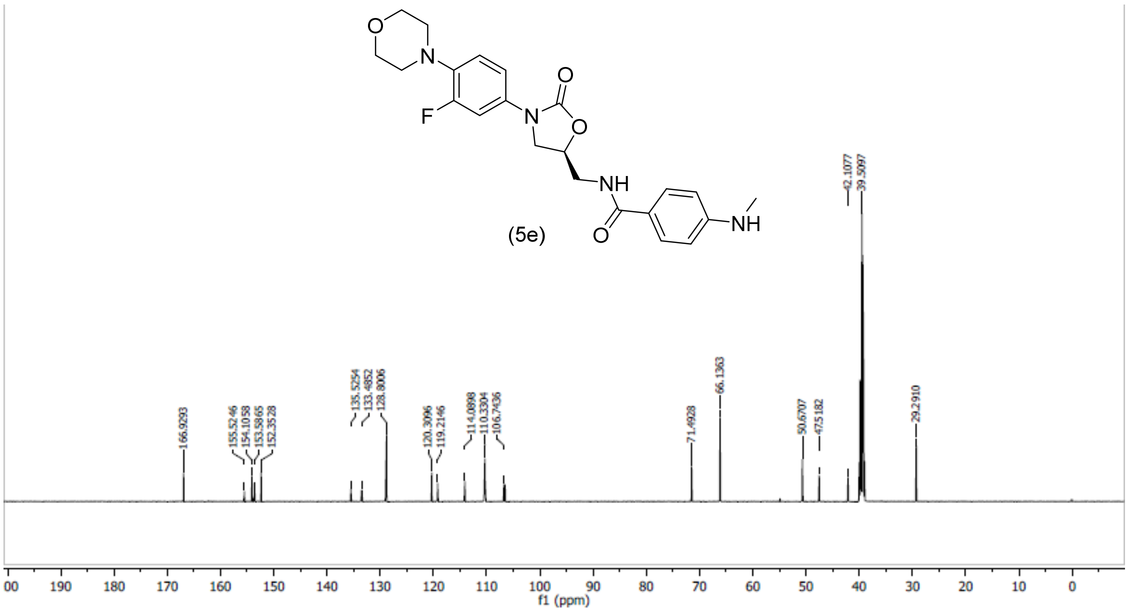
(5e)
O
N
F
NH
f1 (ppm)
166.9293
155.5246
154.1058
153.5865
152.3528
135.5254
133.4852
128.8006
120.3096
119.2146
114.0898
110.3304
106.7436
71.4928
66.1363
50.6707
47.5182
42.1077
39.5097
29.2910

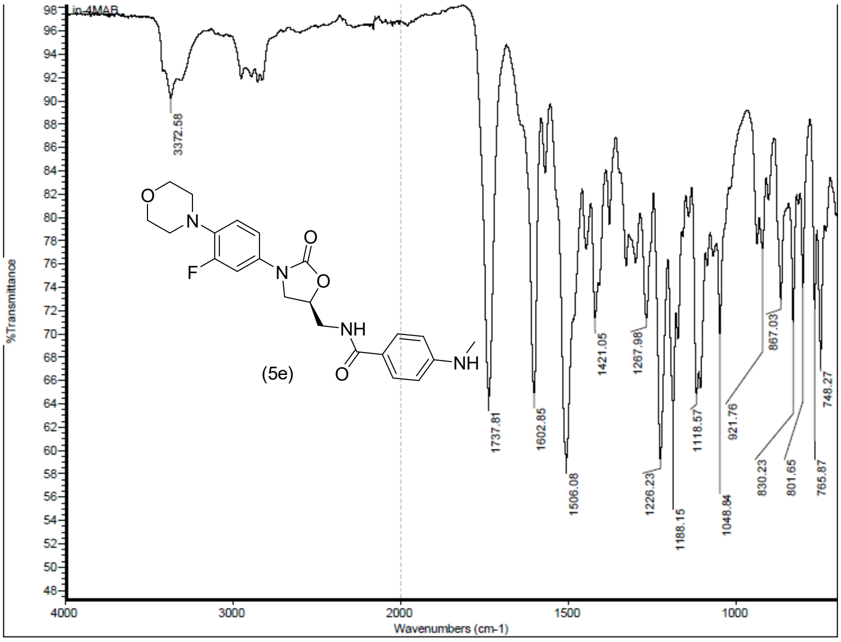

Lin-4MAB
%Transmittance
Wavenumbers (cm-1)
3372.58
1737.81
1602.85
1506.08
1421.05
1267.98
1226.23
1188.15
1118.57
1048.84
921.76
867.03
830.23
801.65
765.87
748.27
(5e)

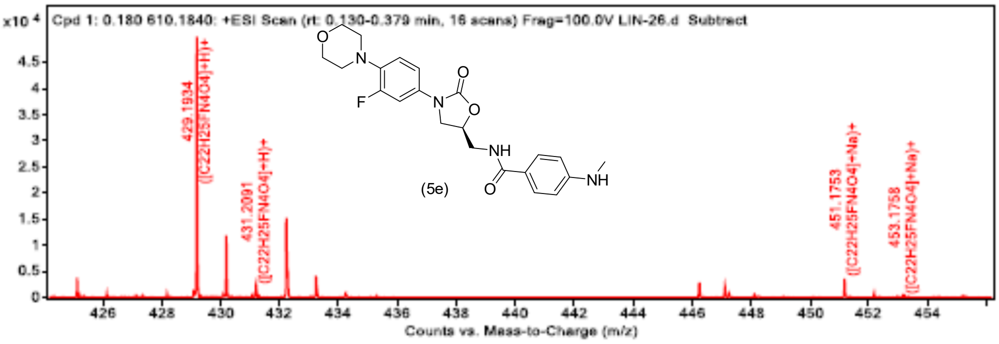

Cpd 1: 0.180 610.1840: +ESI Scan (rt: 0.130-0.379 min, 16 scans) Frag=100.0V LIN-26.d Subtract
x10 4
429.1934
([C22H25FN4O4]+H)+
431.2091
([C22H25FN4O4]+H)+
451.1753
([C22H25FN4O4]+Na)+
453.1758
([C22H25FN4O4]+Na)+
(5e)
Counts vs. Mass-to-Charge (m/z)

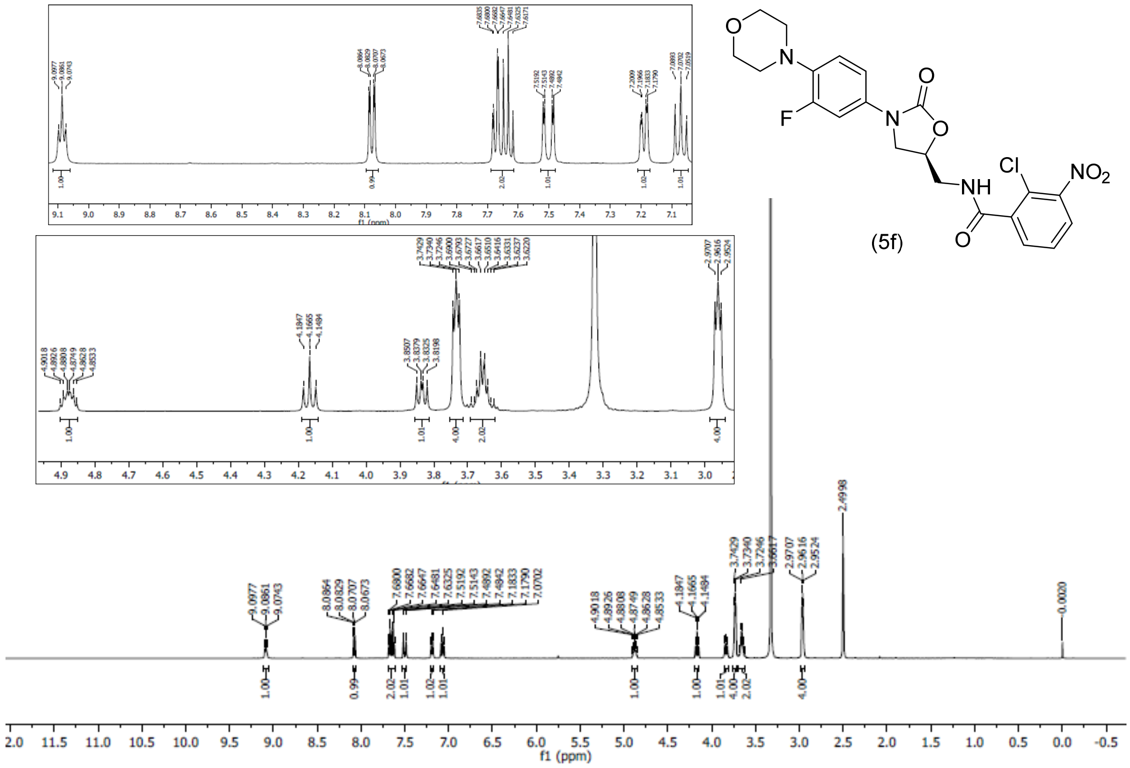

(5f)

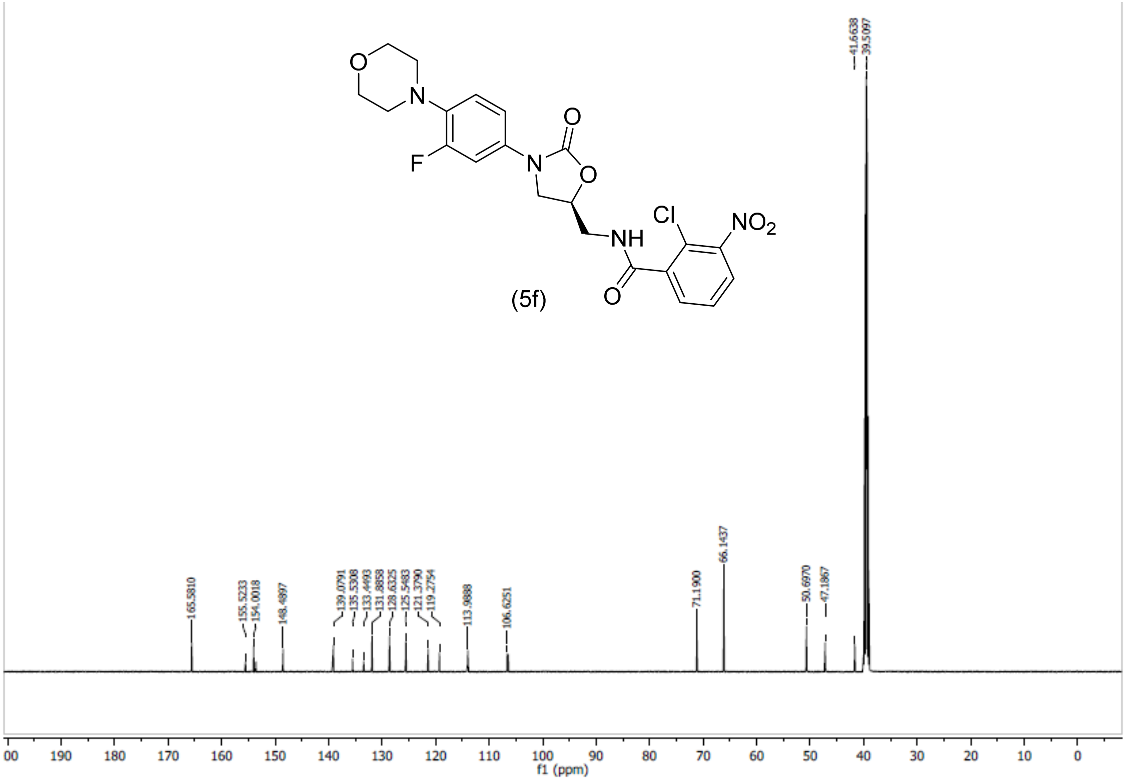

(5f)

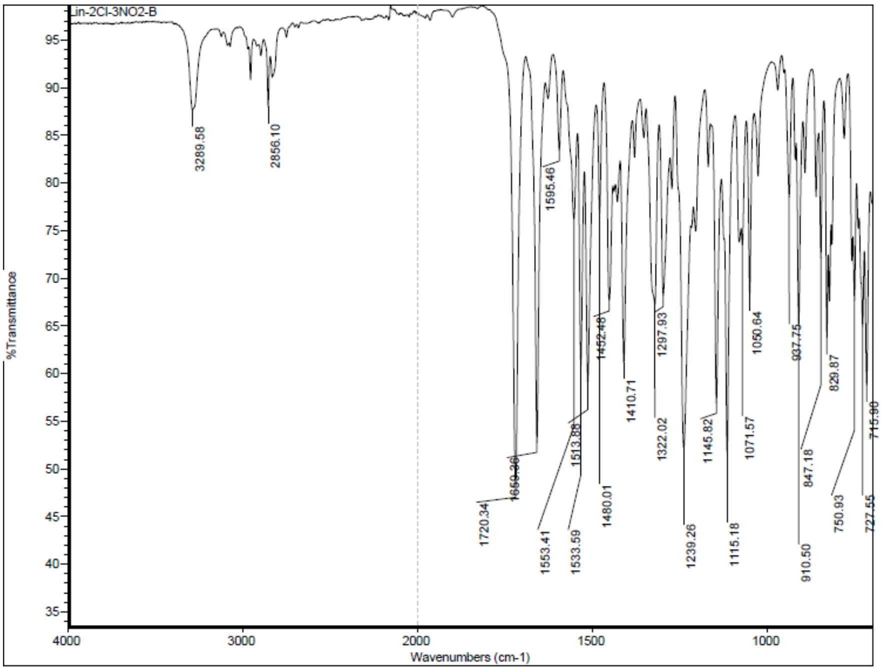
Lin-2Cl-3NO2-B
%Transmittance
Wavenumbers (cm-1)
3289.58
2856.10
1720.34
1659.36
1595.46
1553.41
1533.59
1513.88
1480.01
1452.48
1410.71
1322.02
1297.93
1239.26
1145.82
1115.18
1071.57
1050.64
937.75
910.50
847.18
829.87
750.93
727.55
715.90

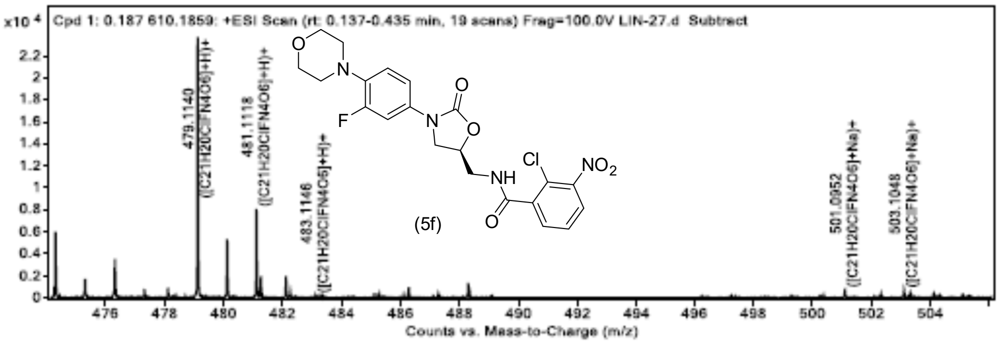
x10 4
Cpd 1: 0.187 610.1859: +ESI Scan (rt: 0.137-0.435 min, 19 scans) Frag=100.0V LIN-27.d Subtract
479.1140 ([C21H20ClFN4O6]+H)+
481.1118 ([C21H20ClFN4O6]+H)+
483.1146 ([C21H20ClFN4O5]+H)+
501.0952 ([C21H20ClFN4O6]+Na)+
503.1048 ([C21H20ClFN4O6]+Na)+
(5f)
Counts vs. Mass-to-Charge (m/z)

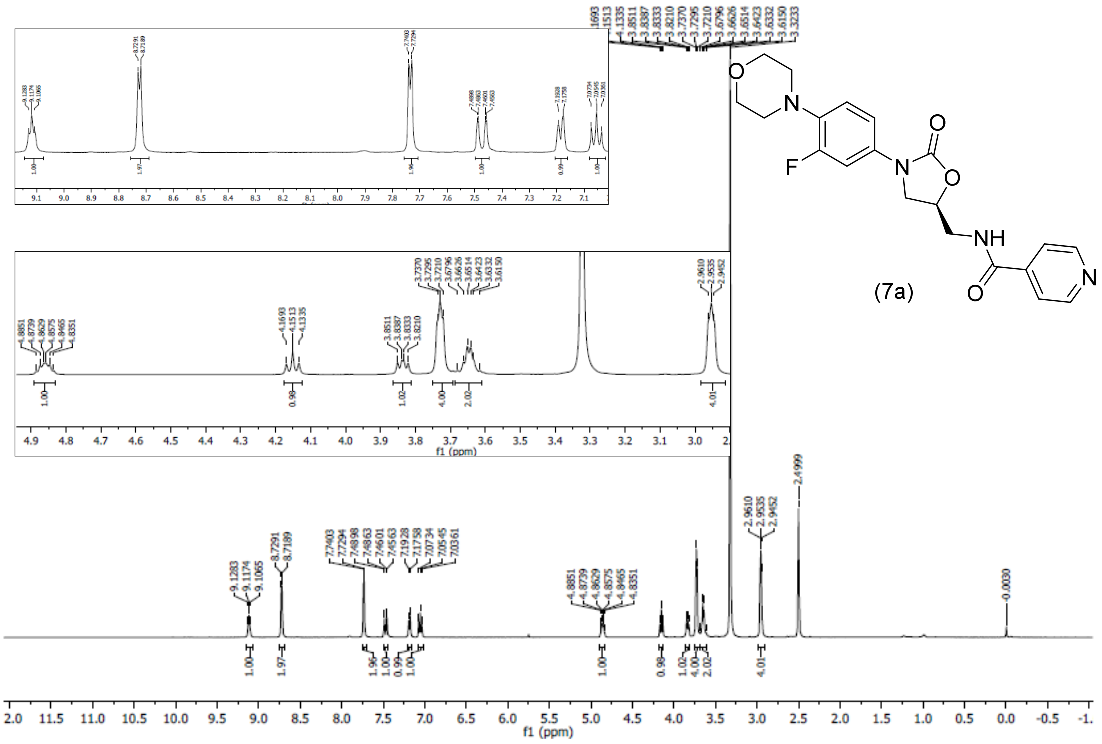

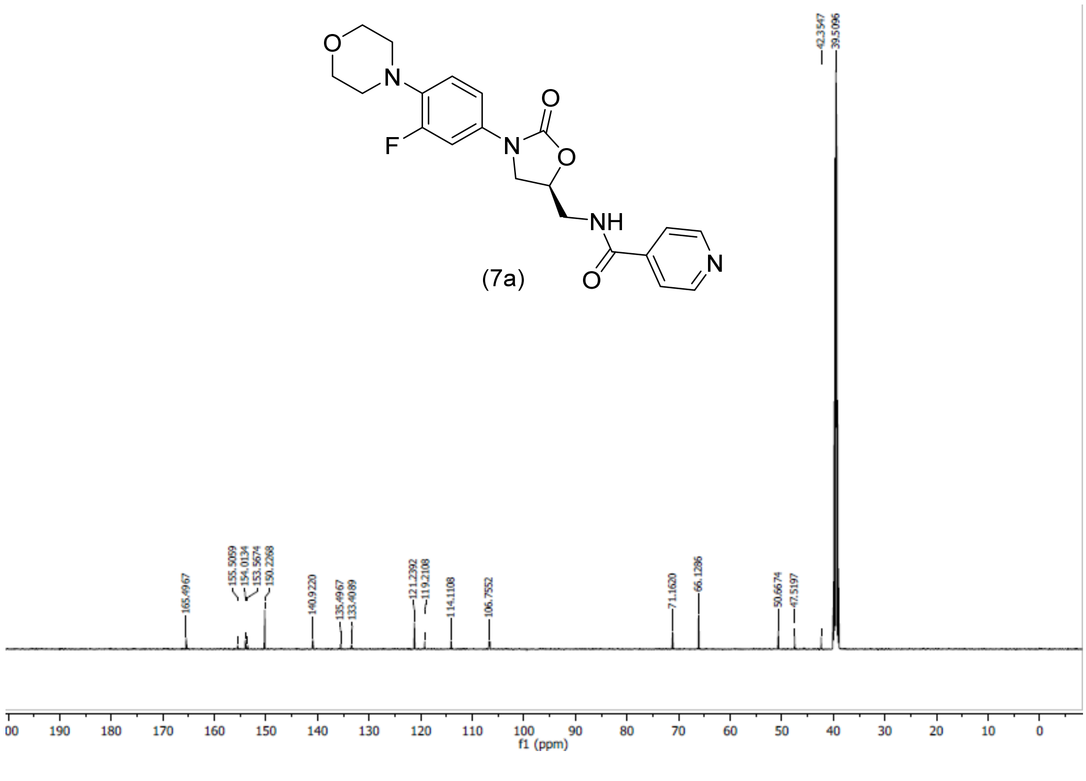

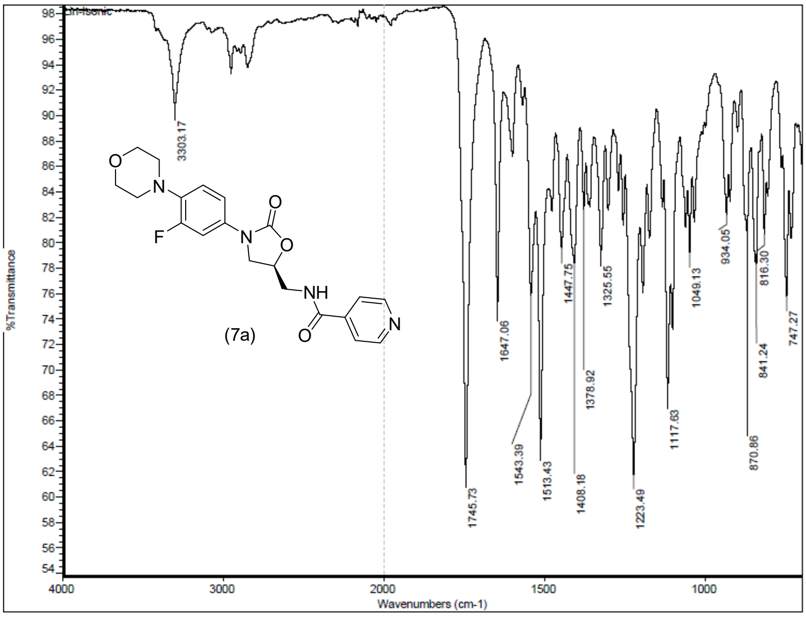
%Transmittance
Wavenumbers (cm-1)
3303.17
1745.73
1647.06
1543.39
1513.43
1447.75
1408.18
1378.92
1325.55
1223.49
1117.63
1049.13
934.05
870.86
841.24
816.30
747.27
(7a)

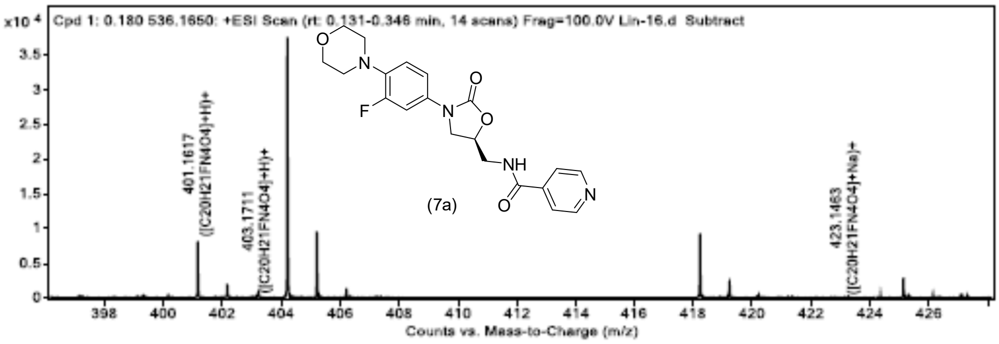
Cpd 1: 0.180 536.1650: +ESI Scan (rt: 0.131-0.346 min, 14 scans) Frag=100.0V Lin-16.d Subtract
401.1617
([C20H21FN4O4]+H)+
403.1711
([C20H21FN4O4]+H)+
423.1463
([C20H21FN4O4]+Na)+
(7a)
Counts vs. Mass-to-Charge (m/z)

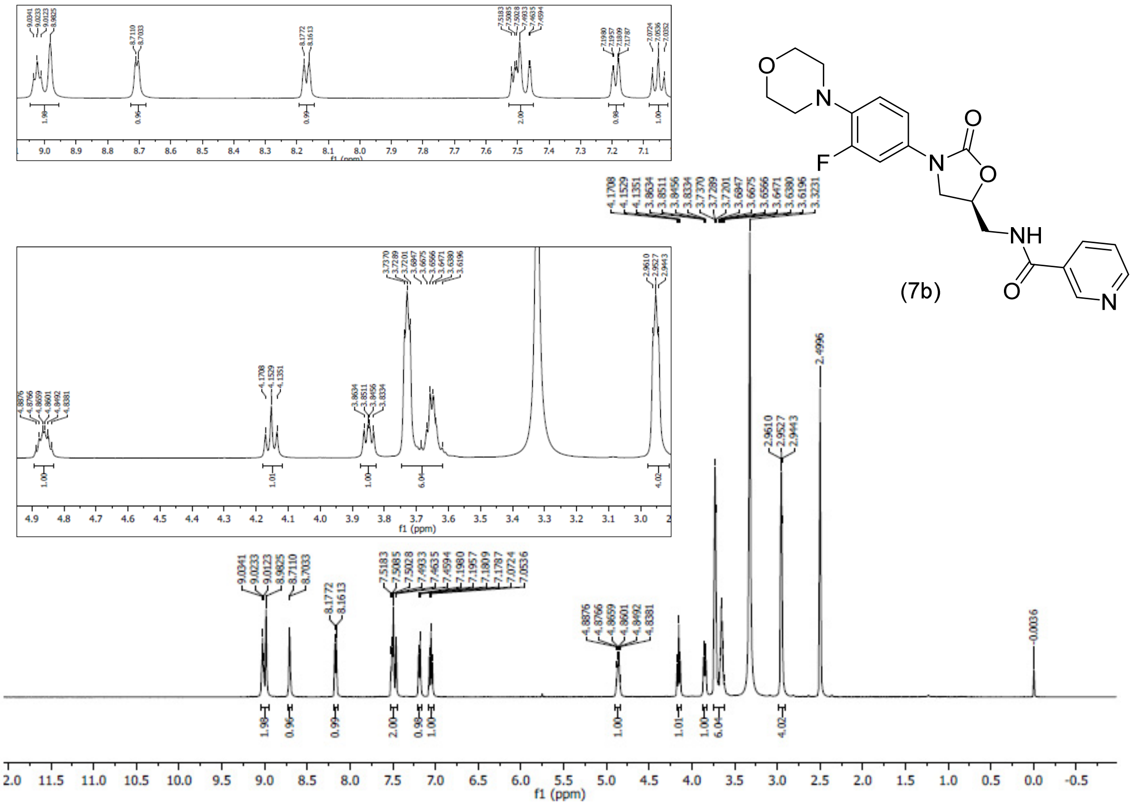
(7b)
f1 (ppm)

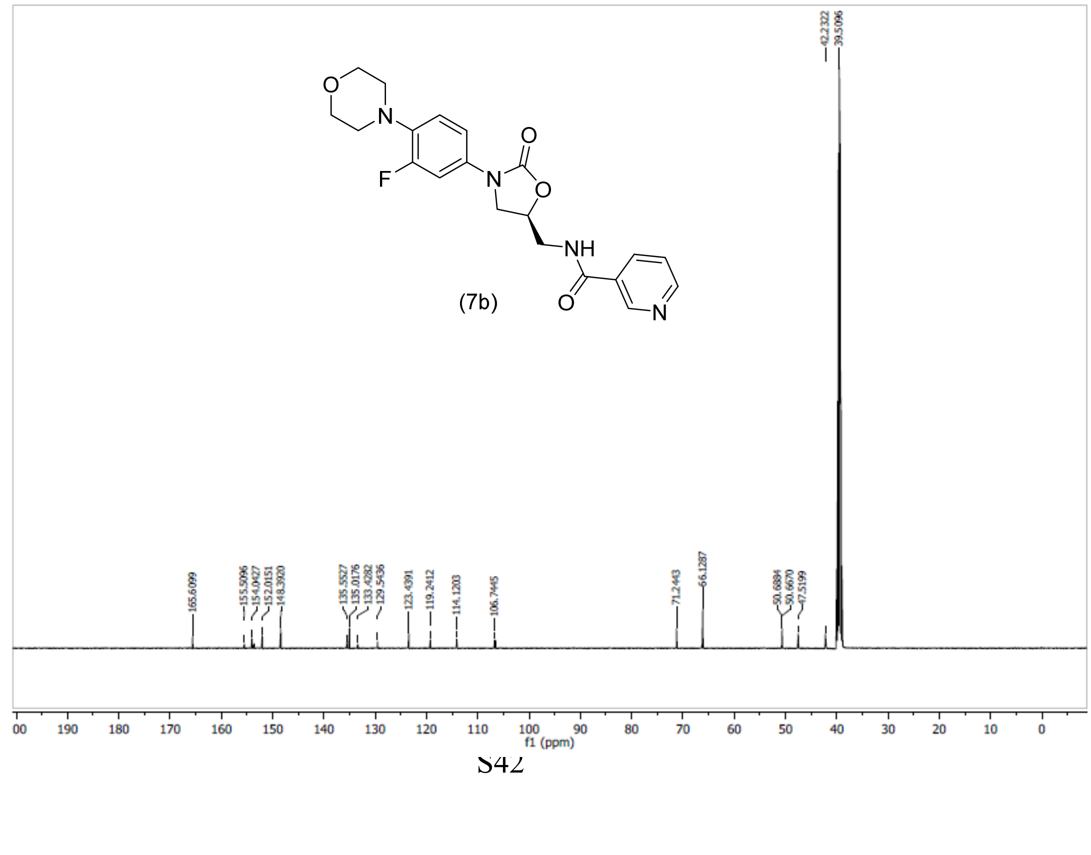
(7b)
f1 (ppm)

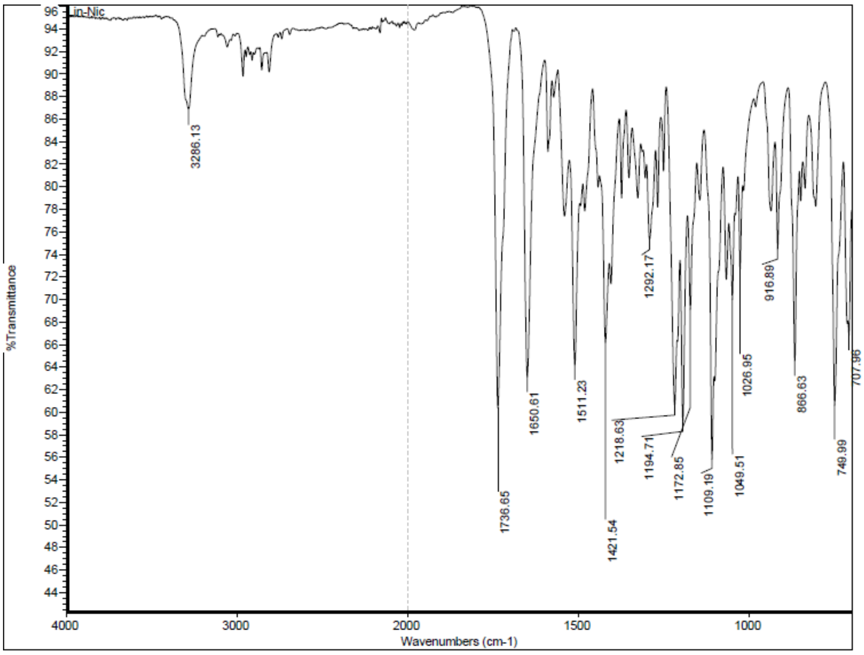
Lin-Nic
%Transmittance
Wavenumbers (cm-1)
3286.13
1736.65
1650.61
1511.23
1421.54
1292.17
1218.63
1194.71
1172.85
1109.19
1049.51
1026.95
916.89
866.63
749.99
707.96

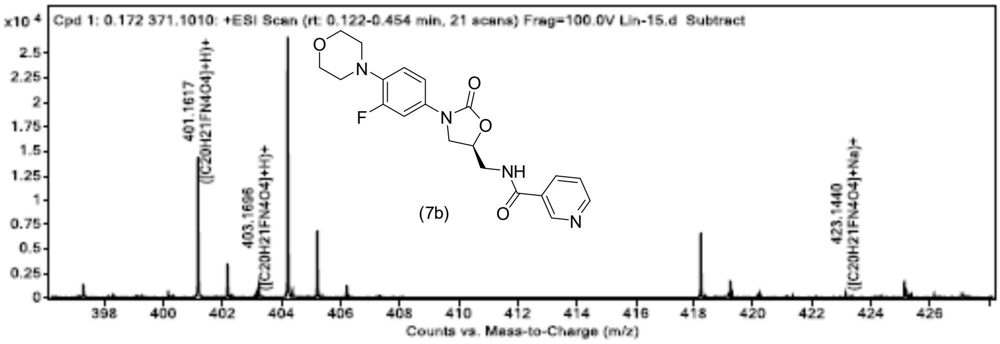
Cpd 1: 0.172 371.1010: +ESI Scan (rt: 0.122-0.454 min, 21 scans) Frag=100.0V Lin-15.d Subtract
401.1617 ([C20H21FN4O4]+H)+
403.1696 ([C20H21FN4O4]+H)+
423.1440 ([C20H21FN4O4]+Na)+
(7b)
Counts vs. Mass-to-Charge (m/z)

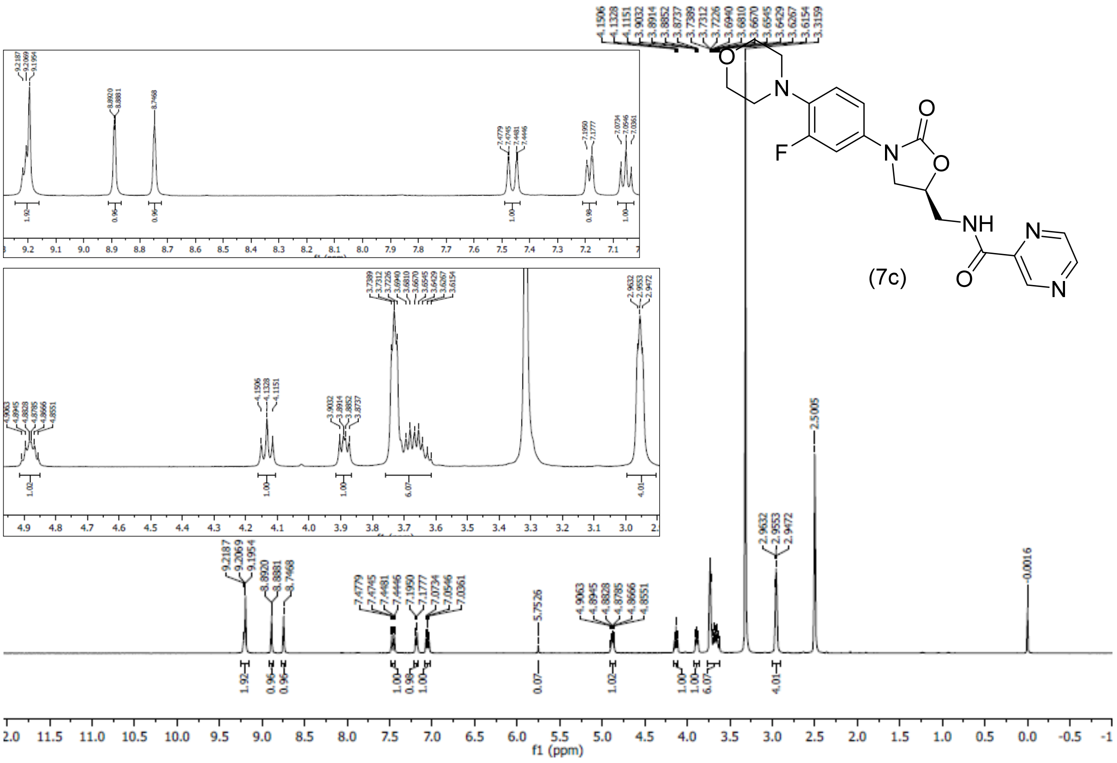
(7c)
f1 (ppm)

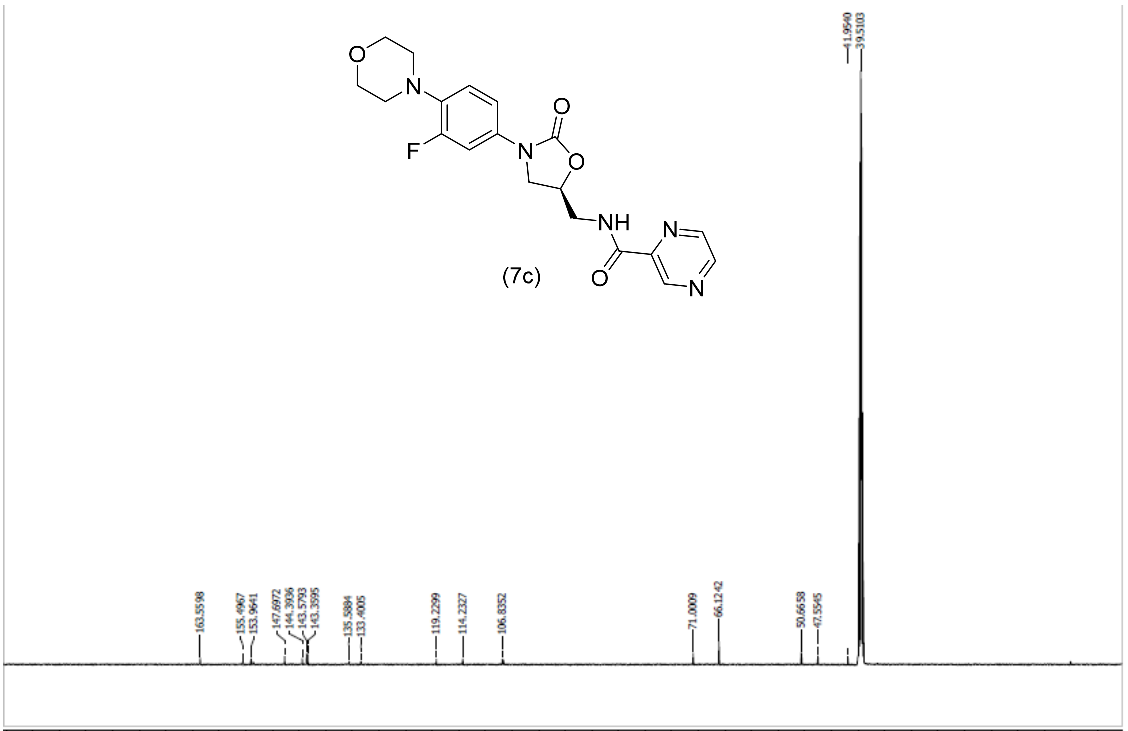
(7c)
f1 (ppm)

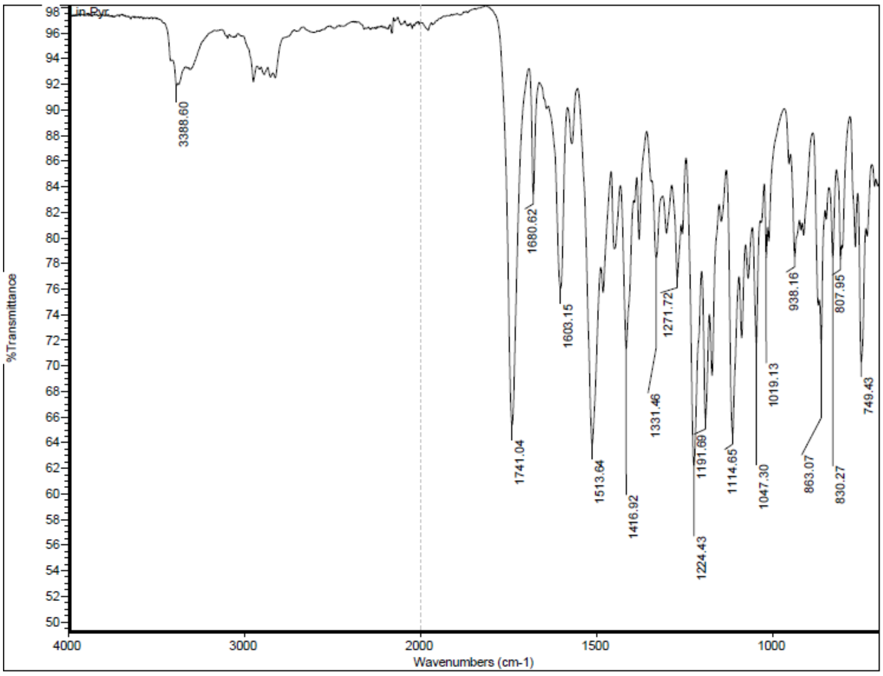
in Pyr
%Transmittance
Wavenumbers (cm-1)
3388.60
1741.04
1680.62
1603.15
1513.64
1416.92
1331.46
1271.72
1224.43
1191.69
1114.65
1047.30
1019.13
938.16
863.07
830.27
807.95
749.43

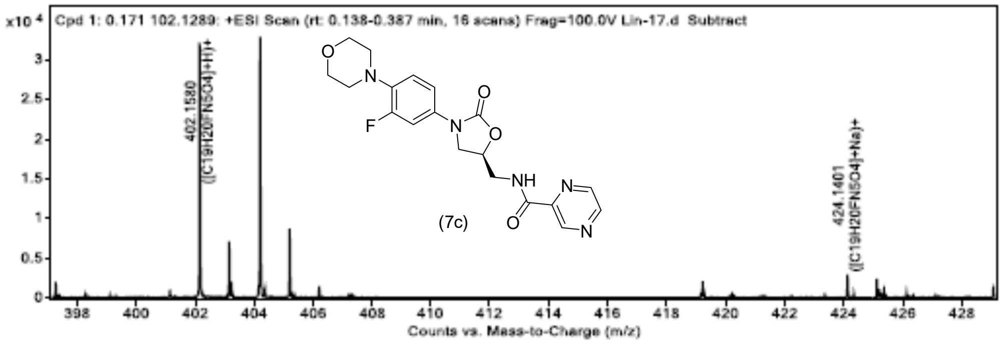
Cpd 1: 0.171 102.1289: +ESI Scan (rt: 0.138-0.387 min, 16 scans) Frag=100.0V Lin-17.d Subtract
x10 4
402.1580
([C19H20FN5O4]+H)+
424.1401
([C19H20FN5O4]+Na)+
(7c)
Counts vs. Mass-to-Charge (m/z)

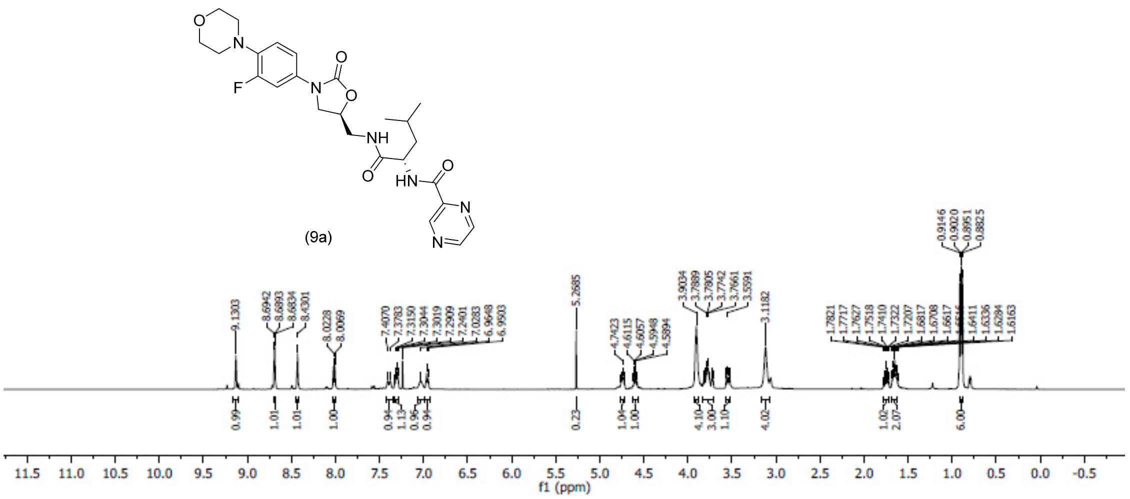

(9a)

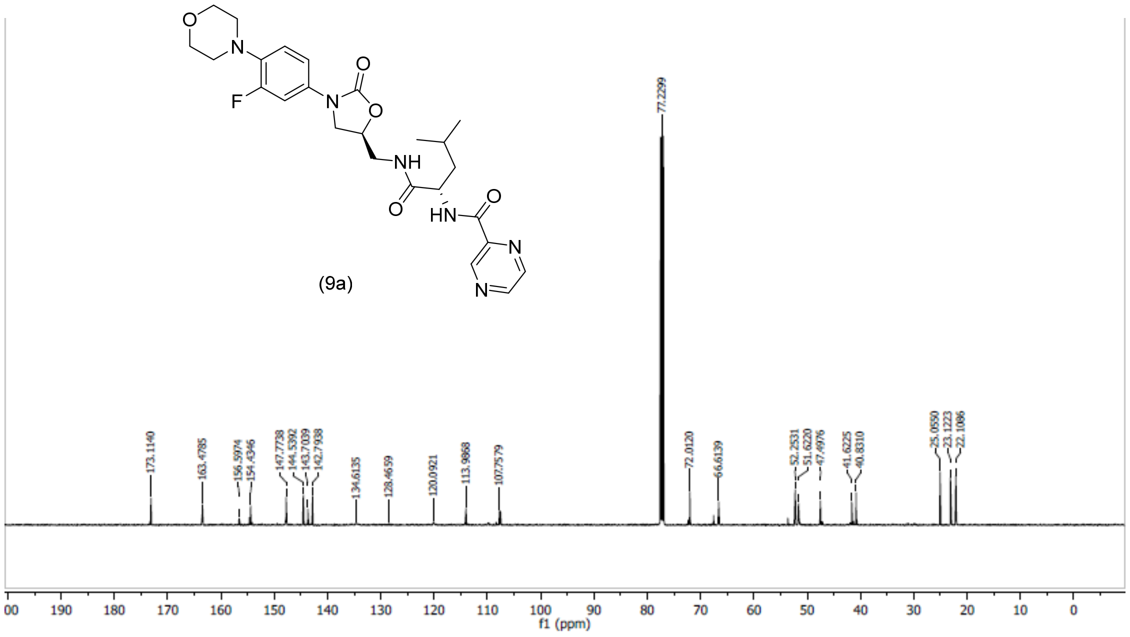

(9a)

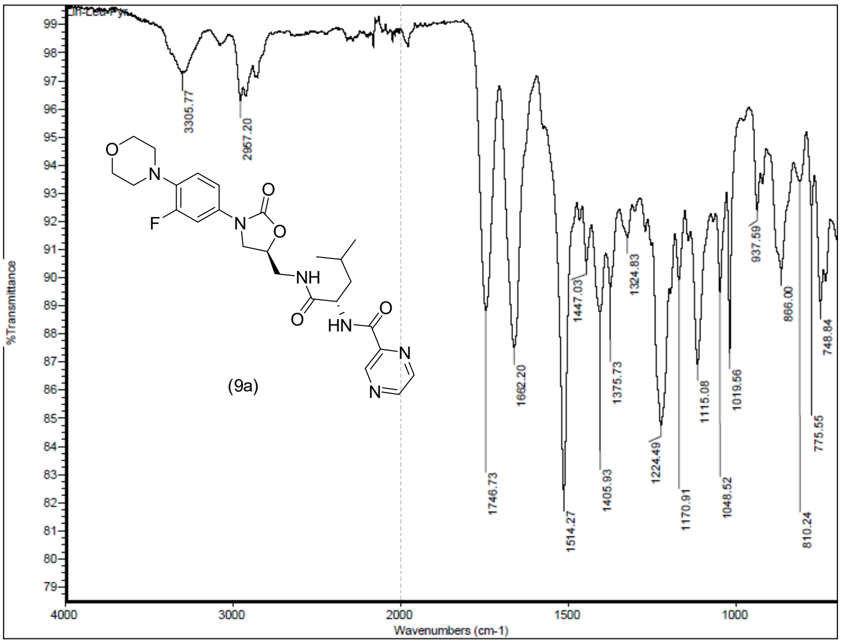
%Transmittance
Wavenumbers (cm-1)
3305.77
2957.20
1746.73
1662.20
1514.27
1447.03
1405.93
1375.73
1324.83
1224.49
1170.91
1115.08
1048.52
1019.56
937.59
866.00
810.24
775.55
748.84
(9a)

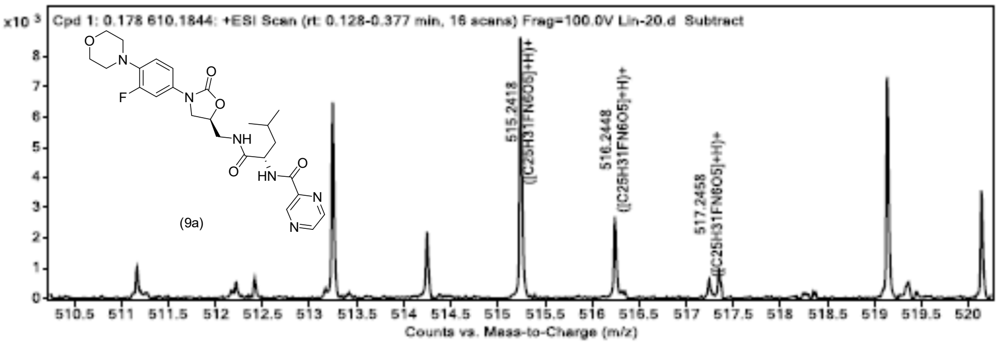
Cpd 1: 0.178 610.1844: +ESI Scan (rt: 0.128-0.377 min, 16 scans) Frag=100.0V Lin-20.d Subtract
515.2418 ([C25H31FN6O5]+H)+
516.2448 ([C25H31FN6O5]+H)+
517.2458 ([C25H31FN6O5]+H)+
Counts vs. Mass-to-Charge (m/z)
(9a)

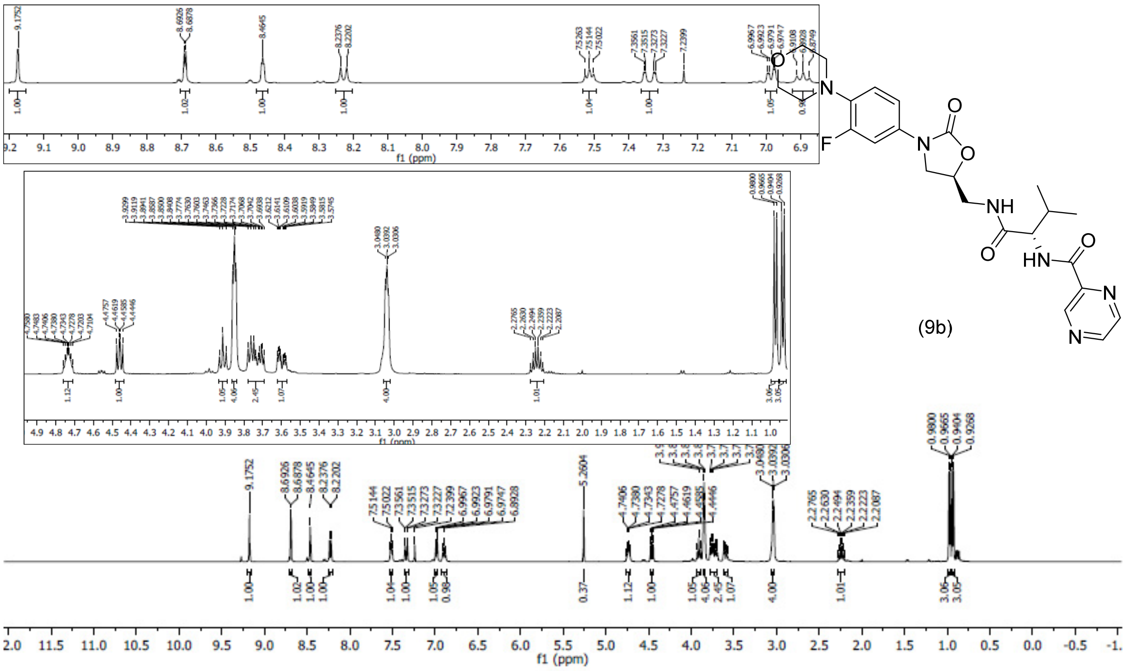

(9b)

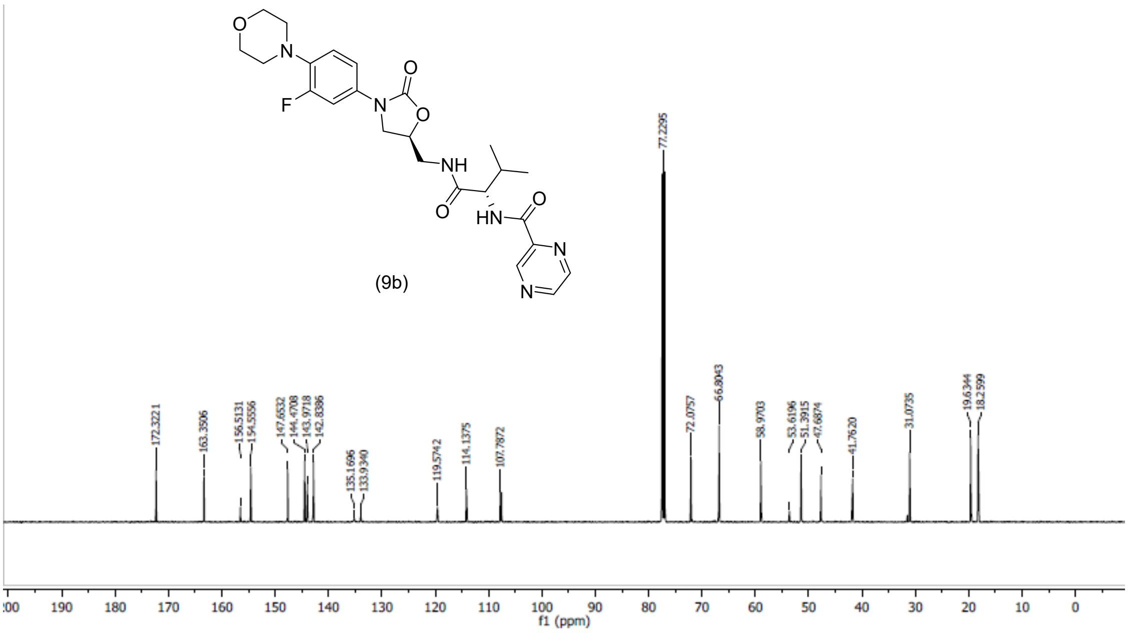

(9b)

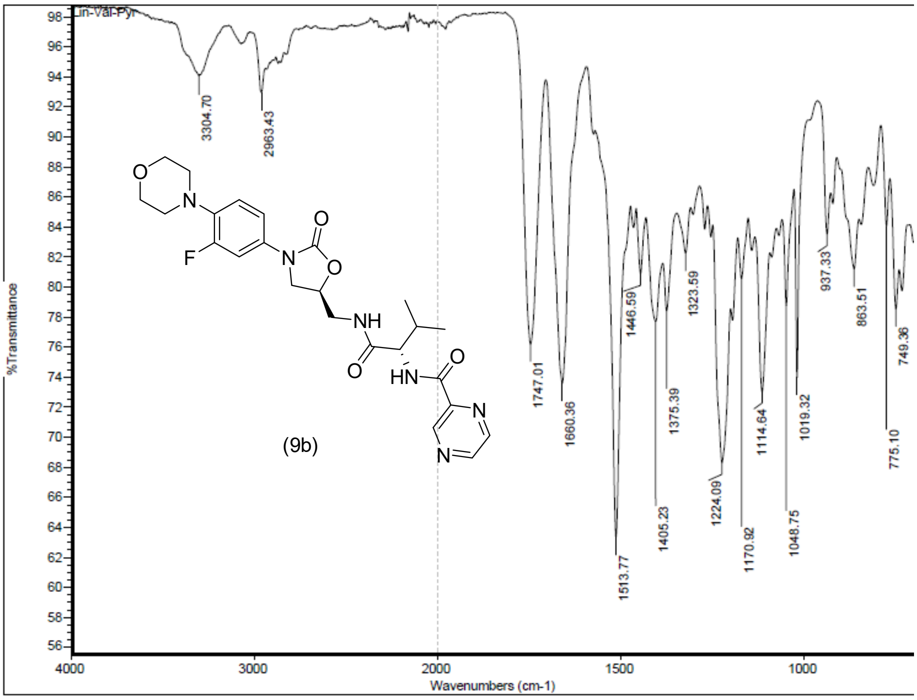
Lin-Val-Pyr
%Transmittance
Wavenumbers (cm-1)
3304.70
2963.43
1747.01
1660.36
1513.77
1446.59
1405.23
1375.39
1323.59
1224.09
1170.92
1114.64
1048.75
1019.32
937.33
863.51
775.10
749.36
(9b)

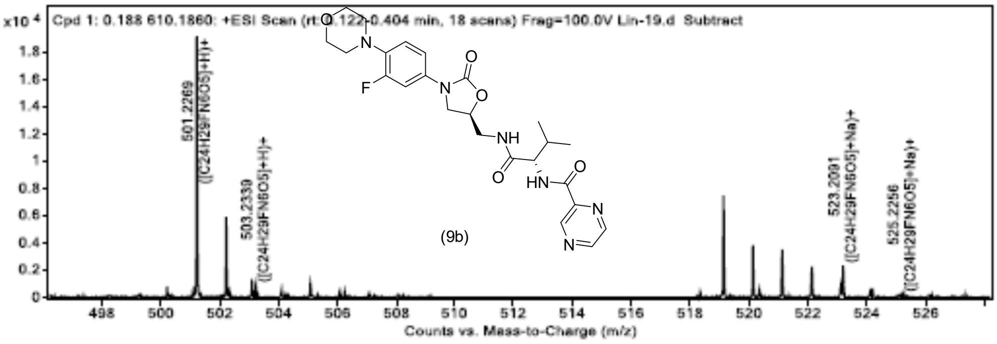
Cpd 1: 0.188 610.1860: +ESI Scan (rt 0.122-0.404 min, 18 scans) Frag=100.0V Lin-19.d Subtract
501.2269 ([C24H29FN6O5]+H)+
503.2339 ([C24H29FN6O5]+H)+
523.2091 ([C24H29FN6O5]+Na)+
525.2256 ([C24H29FN6O5]+Na)+
Counts vs. Mass-to-Charge (m/z)
(9b)

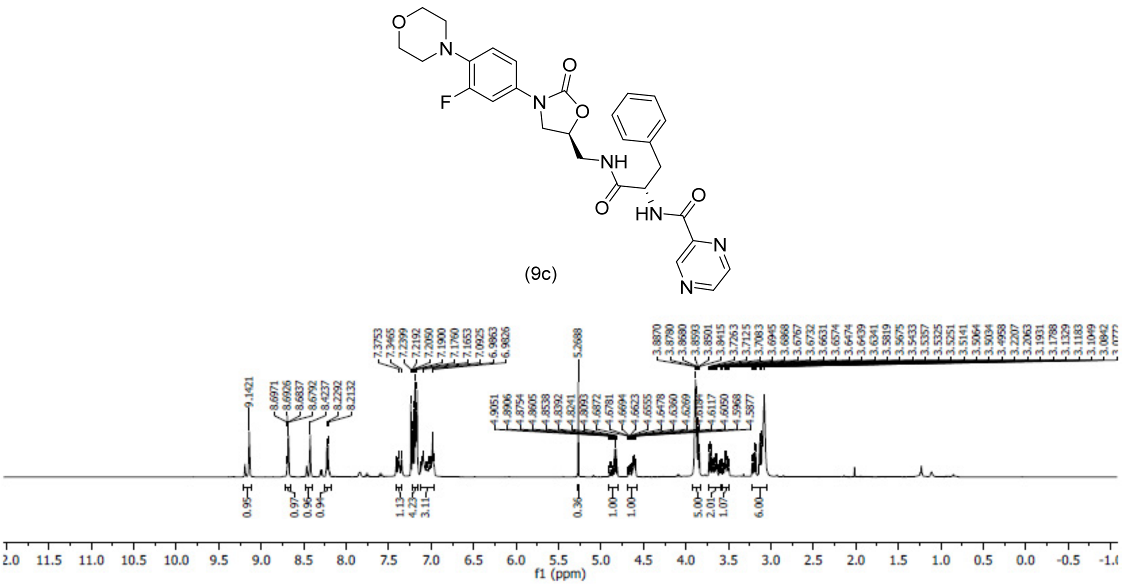
(9c)
f1 (ppm)

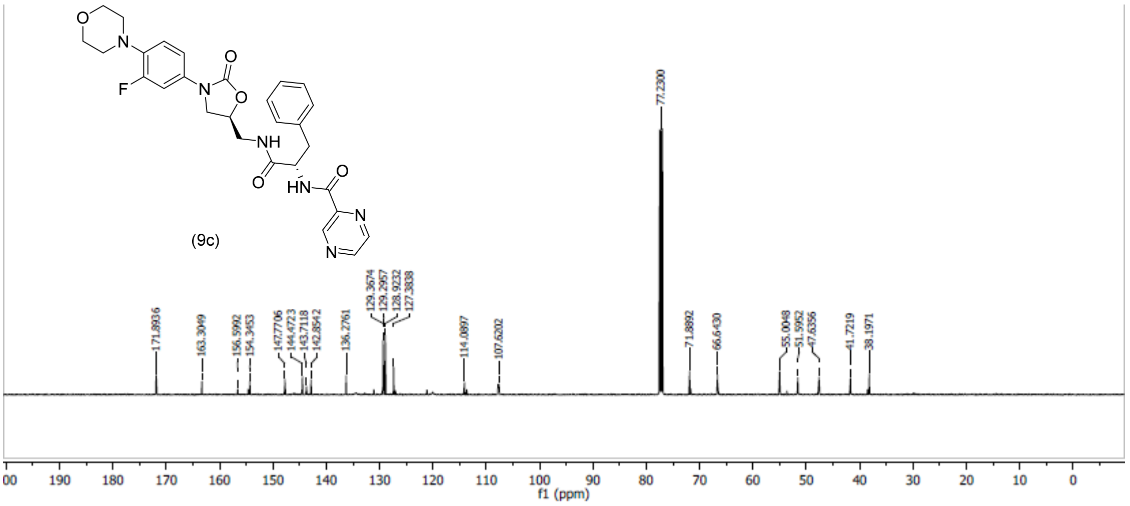
(9c)
f1 (ppm)

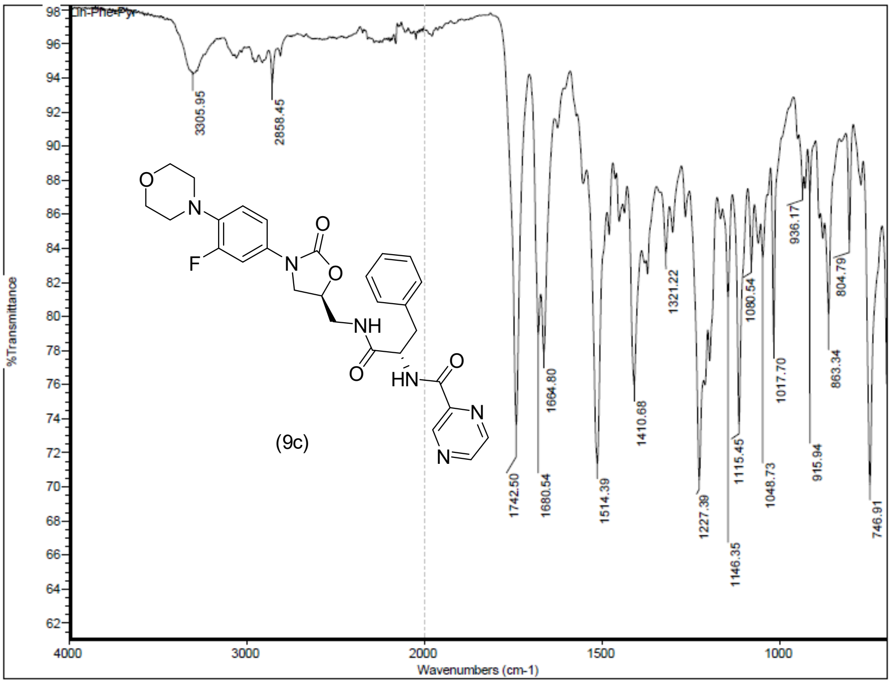

Lin-Phe-Pyr
%Transmittance
Wavenumbers (cm-1)
3305.95
2858.45
1742.50
1680.54
1664.80
1514.39
1410.68
1321.22
1227.39
1146.35
1115.45
1080.54
1048.73
1017.70
936.17
915.94
863.34
804.79
746.91
(9c)

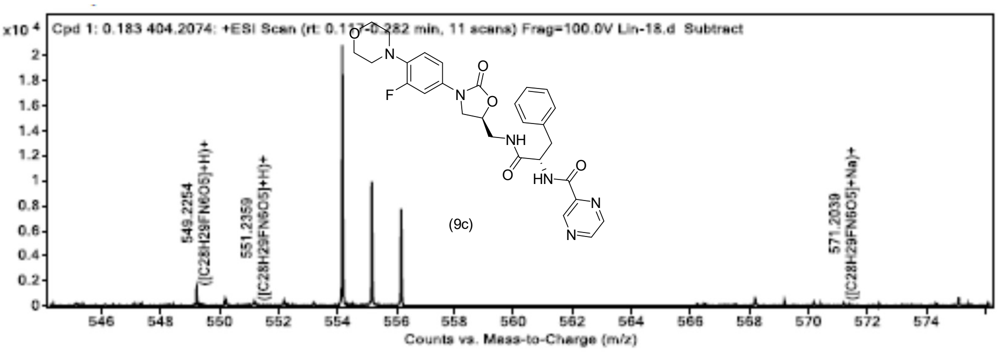

Cpd 1: 0.183 404.2074: +ESI Scan (rt: 0.117-0.282 min, 11 scans) Frag=100.0V Lin-18.d Subtract
549.2254 ([C28H29FN6O5]+H)+
551.2359 ([C28H29FN6O5]+H)+
571.2039 ([C28H29FN6O5]+Na)+
(9c)
Counts vs. Mass-to-Charge (m/z)

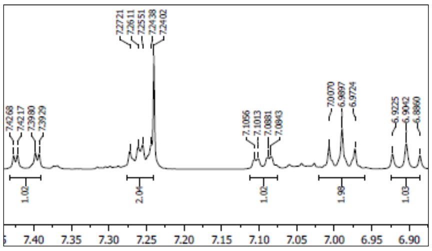
7.4268
7.4217
7.3980
7.3929
7.2721
7.2611
7.2551
7.2438
7.2402
7.1056
7.1013
7.0881
7.0843
7.0070
6.9997
6.9724
6.9225
6.9042
6.8860
1.02
2.04
1.02
1.98
1.03
7.40 7.35 7.30 7.25 7.20 7.15 7.10 7.05 7.00 6.95 6.90

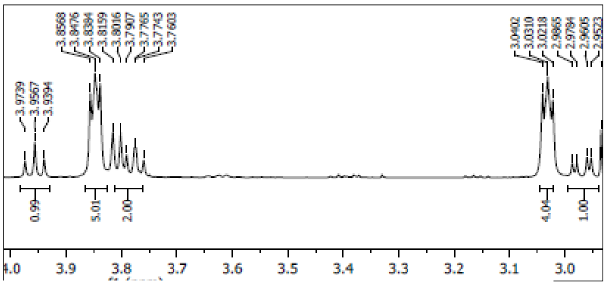
3.9739
3.9567
3.9394
3.8568
3.8476
3.8394
3.8159
3.8016
3.7907
3.7765
3.7743
3.7603
3.0402
3.0310
3.0218
2.9865
2.9784
2.9605
2.9523
0.99
5.01
2.00
4.04
1.00
4.0 3.9 3.8 3.7 3.6 3.5 3.4 3.3 3.2 3.1 3.0

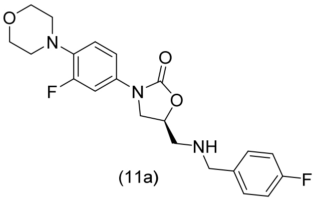
O
N
F
N
O
O
NH
F

(11a)

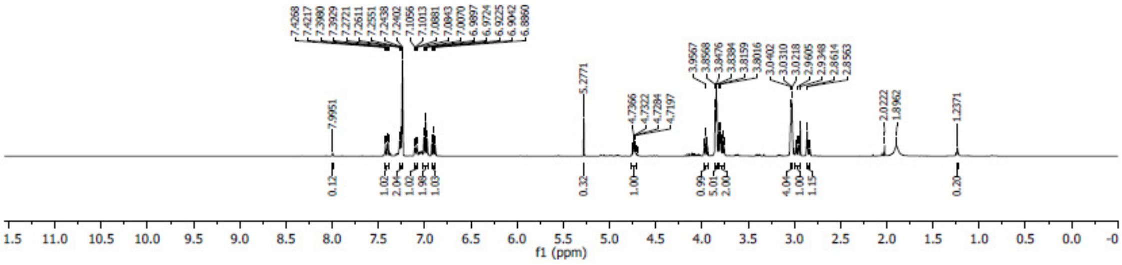
7.4268
7.4217
7.3980
7.3929
7.2721
7.2611
7.2551
7.2438
7.2402
7.1056
7.1013
7.0881
7.0843
7.0070
6.9997
6.9724
6.9225
6.9042
6.8860
7.9951
5.2771
4.7366
4.7322
4.7284
4.7197
3.9567
3.8568
3.8476
3.8394
3.8159
3.8016
3.0402
3.0310
3.0218
2.9605
2.9348
2.8614
2.8563
2.0222
1.8962
1.2371
0.12
1.02
2.04
1.02
1.98
1.03
0.32
1.00
0.99
5.01
2.00
4.04
1.00
1.15
0.20
11.5 11.0 10.5 10.0 9.5 9.0 8.5 8.0 7.5 7.0 6.5 6.0 5.5 5.0 4.5 4.0 3.5 3.0 2.5 2.0 1.5 1.0 0.5 0.0 -0
f1 (ppm)

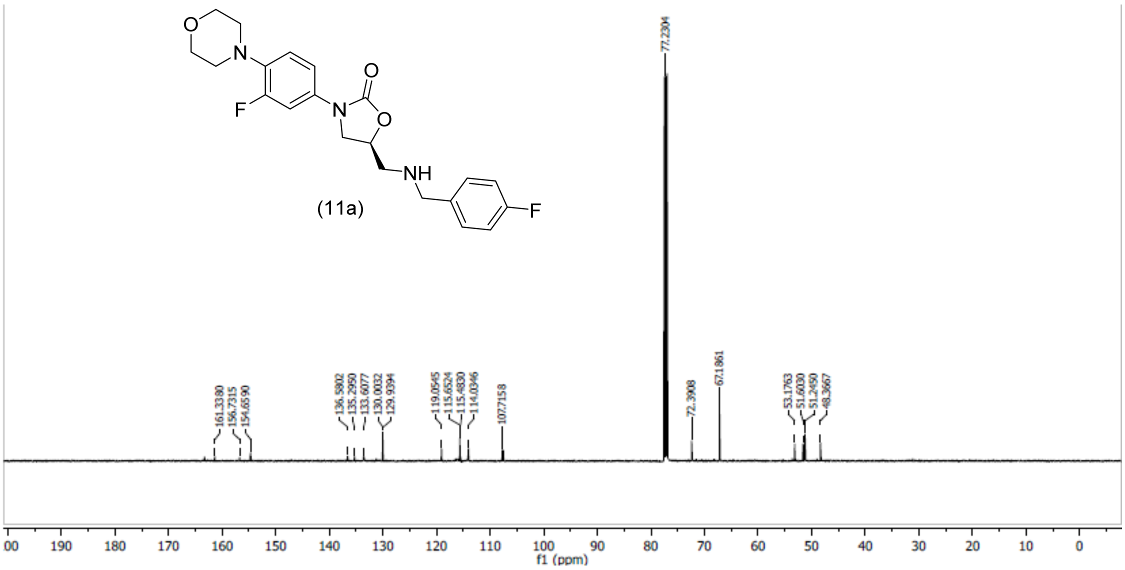
O
N
F
N
O
O
NH
F
(11a)
77.2304
161.3380
156.7315
154.6590
136.5802
135.2950
133.6077
130.0032
129.9394
119.0545
115.6524
115.4830
114.0346
107.7158
72.3908
67.1861
53.1763
51.6030
51.2450
48.3667
200 190 180 170 160 150 140 130 120 110 100 90 80 70 60 50 40 30 20 10 0
f1 (ppm)

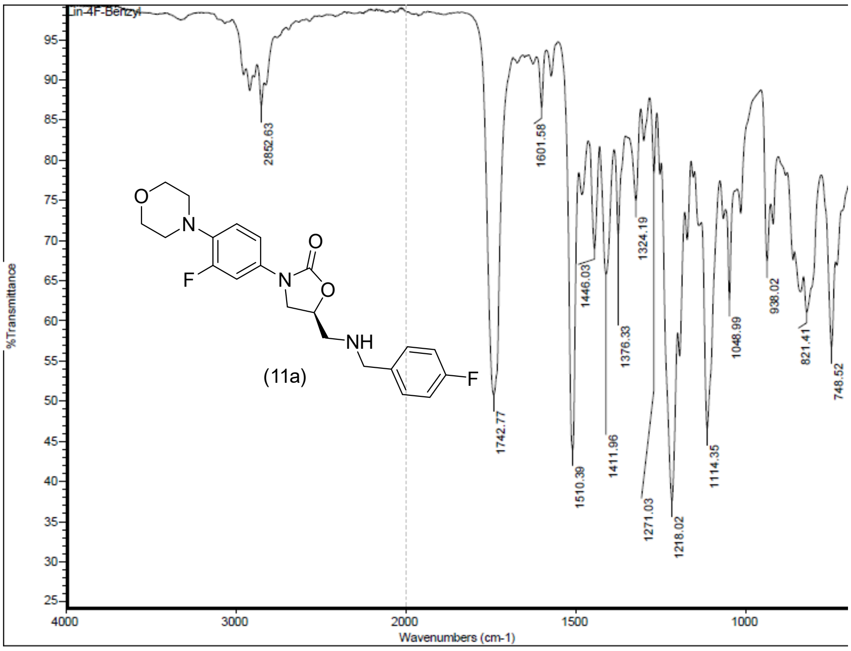
Lin-4F-Benzyl
%Transmittance
Wavenumbers (cm-1)
2852.63
1601.58
1742.77
1510.39
1446.03
1411.96
1376.33
1324.19
1271.03
1218.02
1114.35
1048.99
938.02
821.41
748.52
(11a)

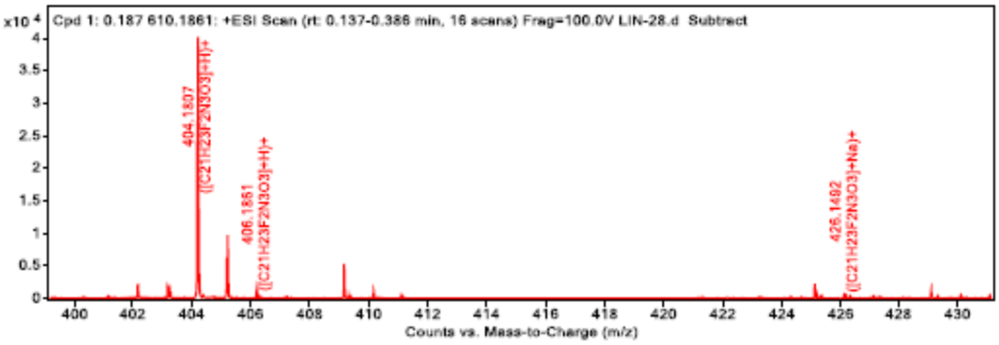
Cpd 1: 0.187 610.1861: +ESI Scan (rt: 0.137-0.386 min, 16 scans) Frag=100.0V LIN-28.d Subtract
404.1807
([C21H23F2N3O3]+H)+
406.1861
([C21H23F2N3O3]+H)+
426.1492
([C21H23F2N3O3]+Na)+
Counts vs. Mass-to-Charge (m/z)

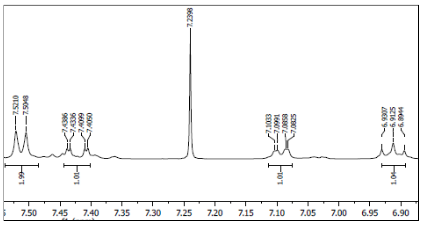

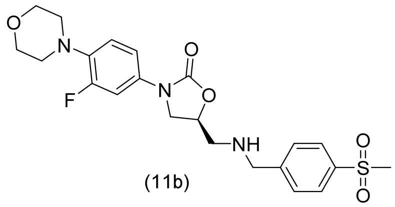
O
N
F
N
O
O
NH
S
O
O

(11b)

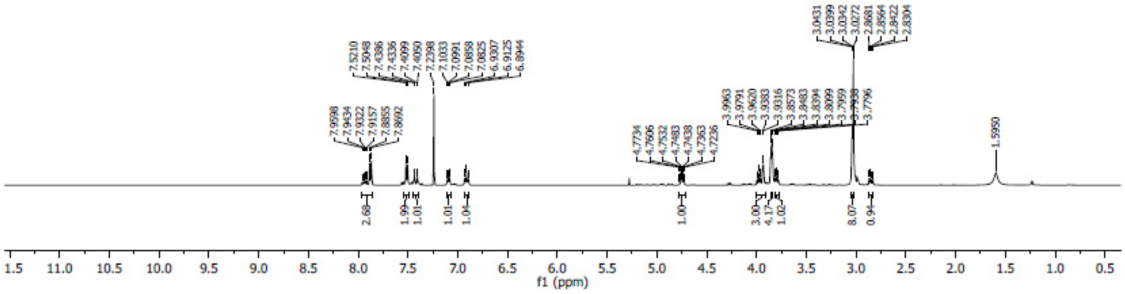
7.5210
7.5048
7.4386
7.4336
7.4099
7.4050
7.2398
7.1033
7.0991
7.0858
7.0825
6.9307
6.9125
6.8944
7.9598
7.9434
7.9322
7.9157
7.8855
7.8692
4.7734
4.7606
4.7532
4.7483
4.7438
4.7363
4.7236
3.9963
3.9791
3.9620
3.9383
3.9316
3.8573
3.8483
3.8394
3.8099
3.7959
3.7938
3.7796
3.0431
3.0399
3.0342
3.0272
2.8681
2.8564
2.8422
2.8304
1.5950
2.68
1.99
1.01
1.01
1.04
1.00
3.00
4.17
1.02
8.07
0.94
f1 (ppm)

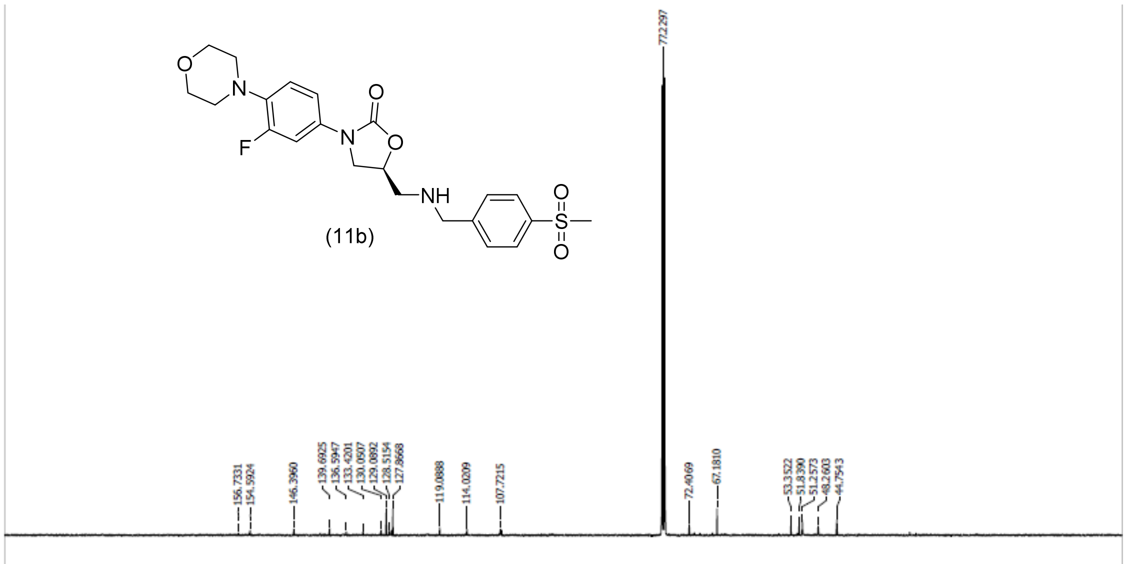
O
N
F
N
O
O
NH
S
O
O
(11b)
77.2297
156.7331
154.5924
146.3960
139.6925
136.5947
133.4201
130.0507
129.0892
128.5154
127.8668
119.0888
114.0209
107.7215
72.4069
67.1810
53.3522
51.8390
51.2573
48.2603
44.7543
f1 (ppm)

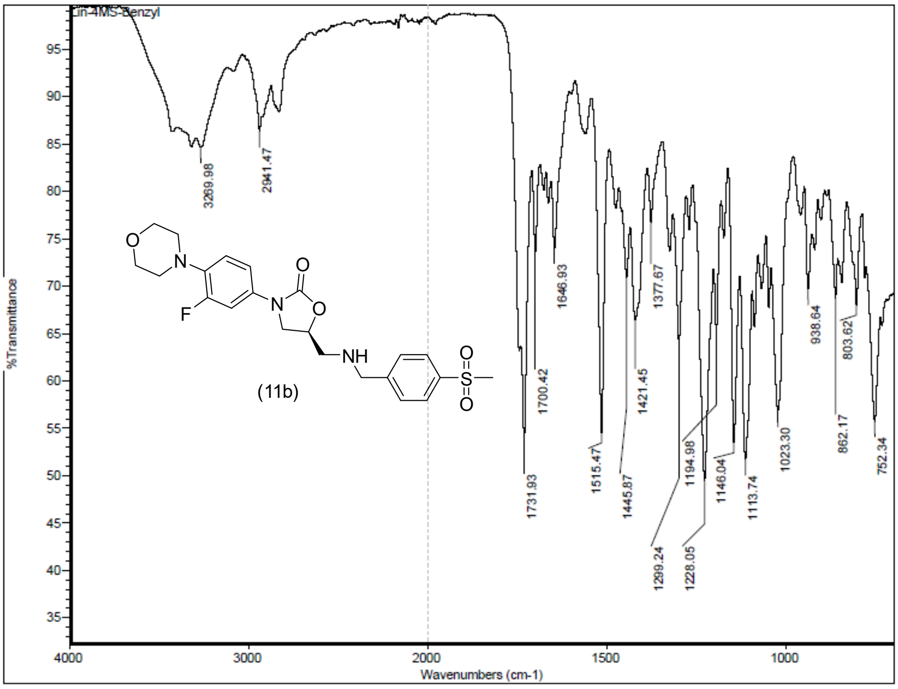
Lin-4MS-Benzyl
%Transmittance
Wavenumbers (cm-1)
3269.98
2941.47
1731.93
1700.42
1646.93
1515.47
1445.87
1421.45
1377.67
1299.24
1228.05
1194.98
1146.04
1113.74
1023.30
938.64
862.17
803.62
752.34
F
O
N
NH
(11b)

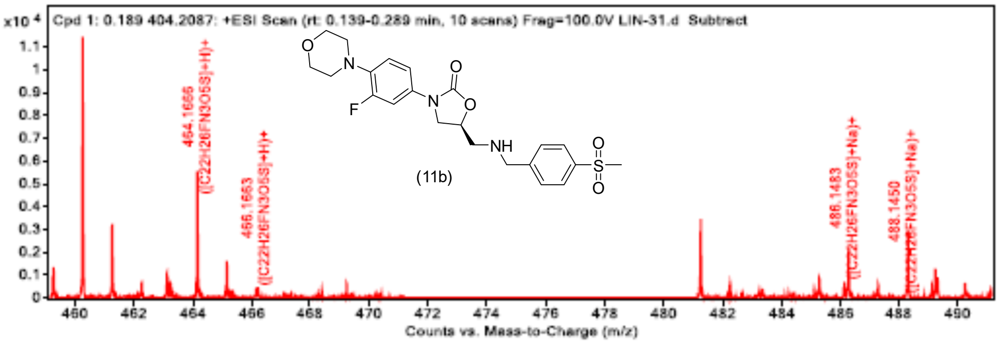
Cpd 1: 0.189 404.2087: +ESI Scan (rt: 0.139-0.289 min, 10 scans) Frag=100.0V LIN-31.d Subtract
464.1665 ([C22H26FN3O5S]+H)+
466.1663 ([C22H26FN3O5S]+H)+
486.1483 ([C22H26FN3O5S]+Na)+
488.1450 ([C22H26FN3O5S]+Na)+
Counts vs. Mass-to-Charge (m/z)
(11b)

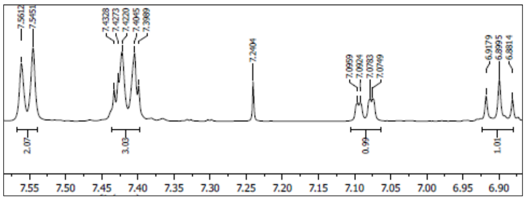

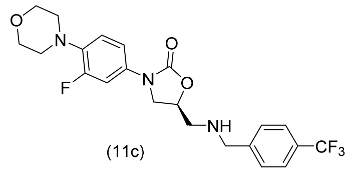

(11c)

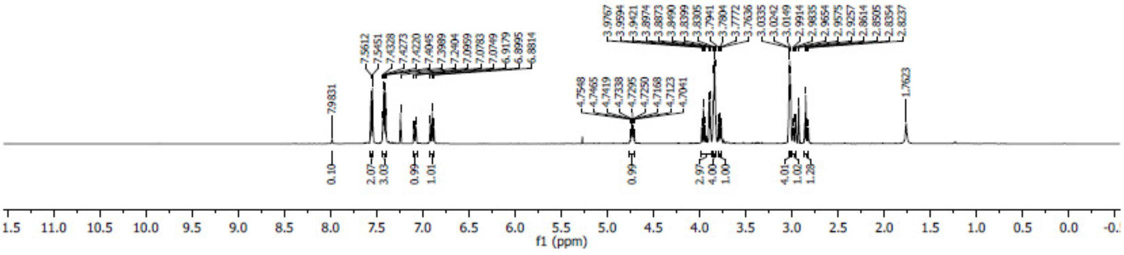

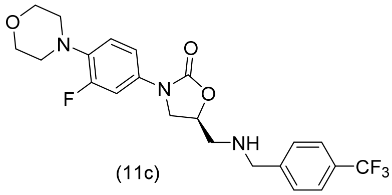

(11c)

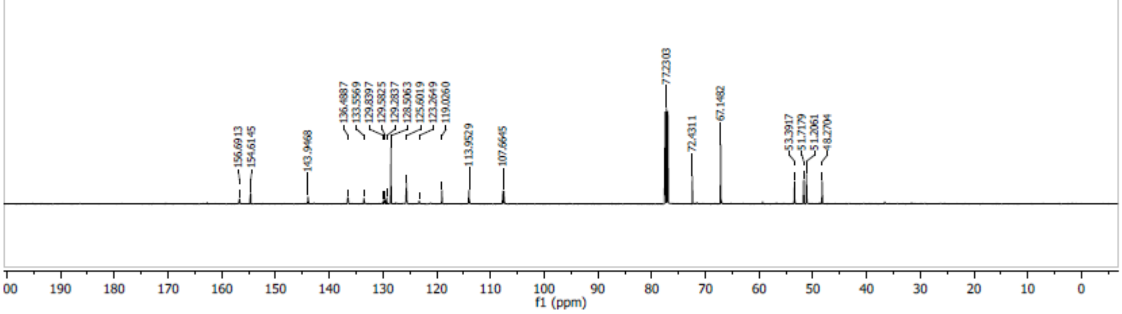

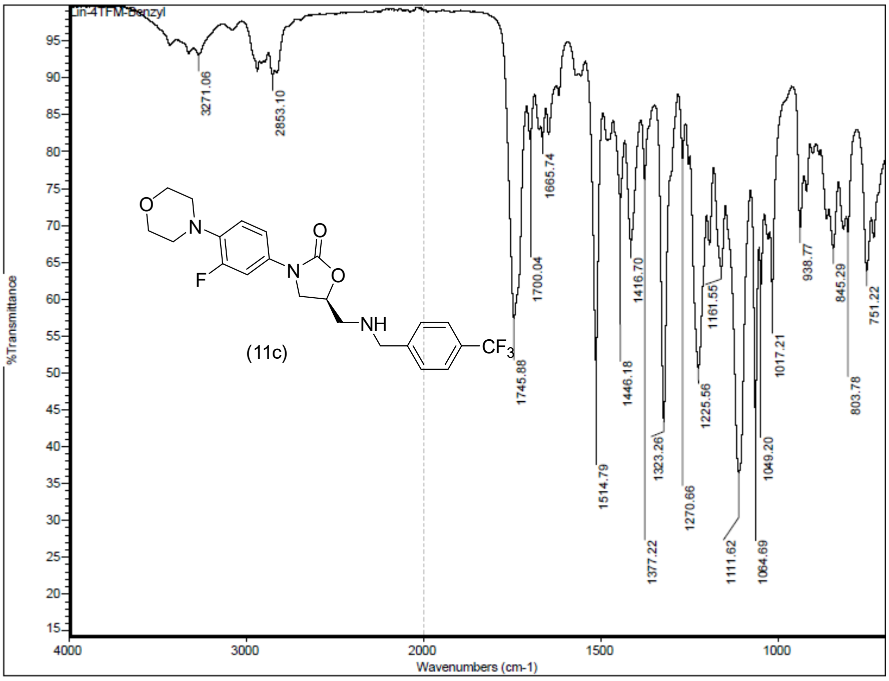
Lin-4TFM-Benzyl
%Transmittance
Wavenumbers (cm-1)
3271.06
2853.10
1745.88
1700.04
1665.74
1514.79
1446.18
1416.70
1377.22
1323.26
1270.66
1225.56
1161.55
1111.62
1064.69
1049.20
1017.21
938.77
845.29
803.78
751.22
(11c)

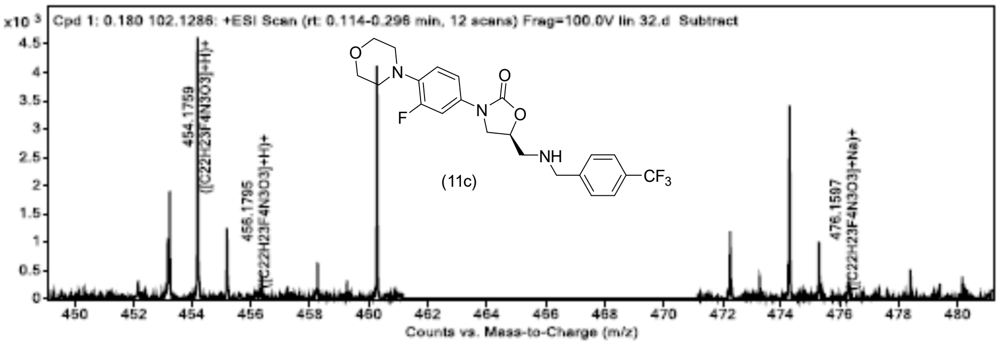
Cpd 1: 0.180 102.1286: +ESI Scan (rt: 0.114-0.296 min, 12 scans) Frag=100.0V lin 32.d Subtract
454.1759
([C22H23F4N3O3]+H)+
456.1795
([C22H23F4N3O3]+H)+
476.1597
([C22H23F4N3O3]+Na)+
(11c)
Counts vs. Mass-to-Charge (m/z)

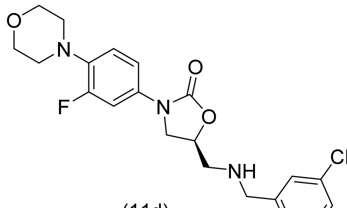

(11d)

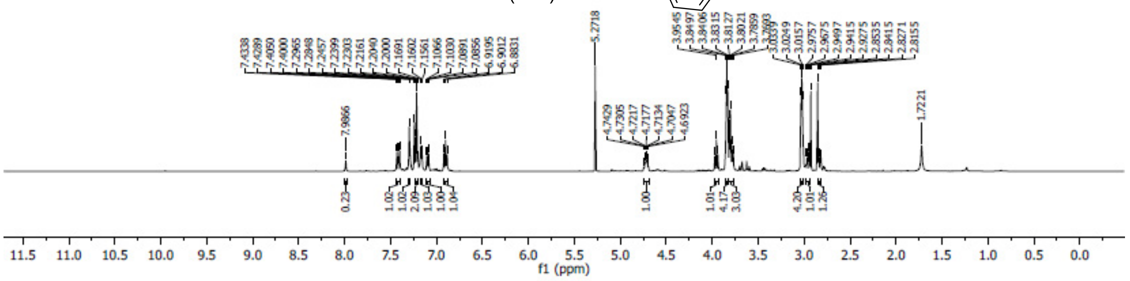

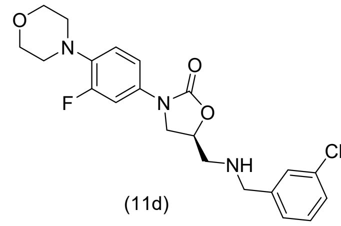

(11d)

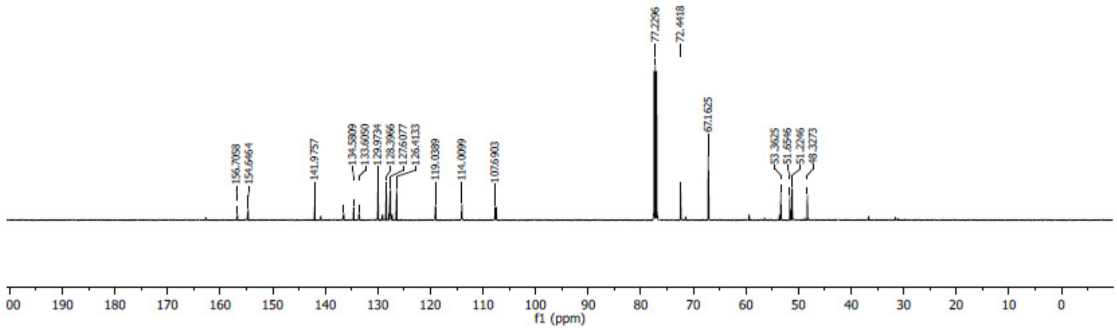

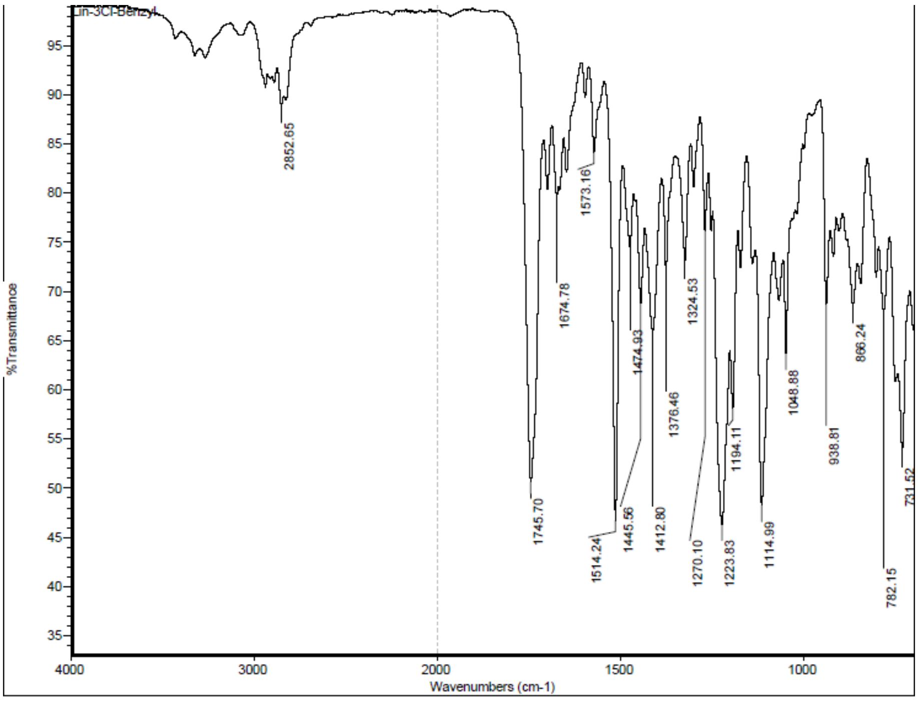
Lin-3Cl-Benzyl
%Transmittance
Wavenumbers (cm-1)
2852.65
1745.70
1674.78
1573.16
1514.24
1474.93
1445.56
1412.80
1376.46
1324.53
1270.10
1223.83
1194.11
1114.99
1048.88
938.81
866.24
782.15
731.52

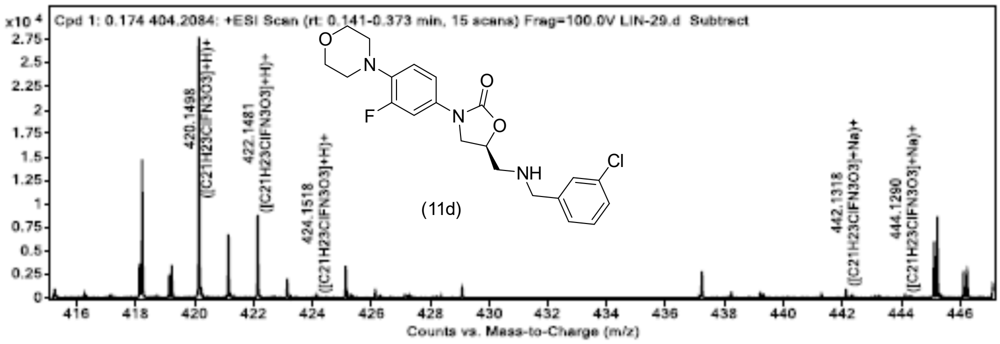
Cpd 1: 0.174 404.2084: +ESI Scan (rt: 0.141-0.373 min, 15 scans) Frag=100.0V LIN-29.d Subtract
Counts vs. Mass-to-Charge (m/z)
420.1498 ([C21H23ClFN3O3]+H)+
422.1481 ([C21H23ClFN3O3]+H)+
424.1518 ([C21H23ClFN3O3]+H)+
442.1318 ([C21H23ClFN3O3]+Na)+
444.1290 ([C21H23ClFN3O3]+Na)+
(11d)

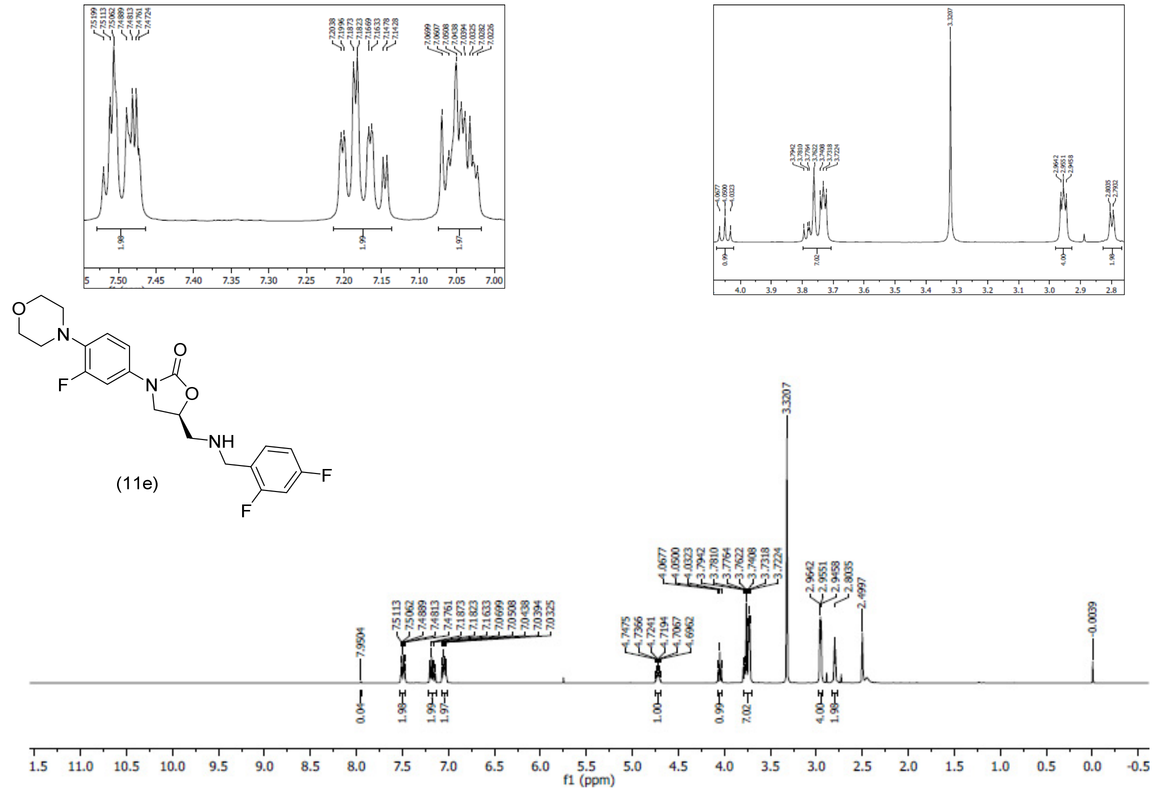

(11e)

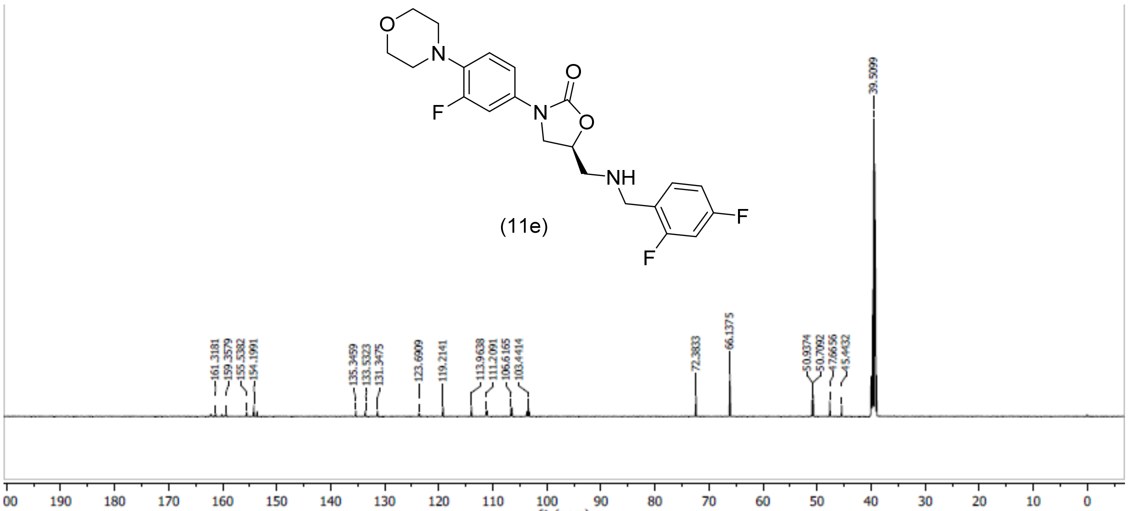

(11e)

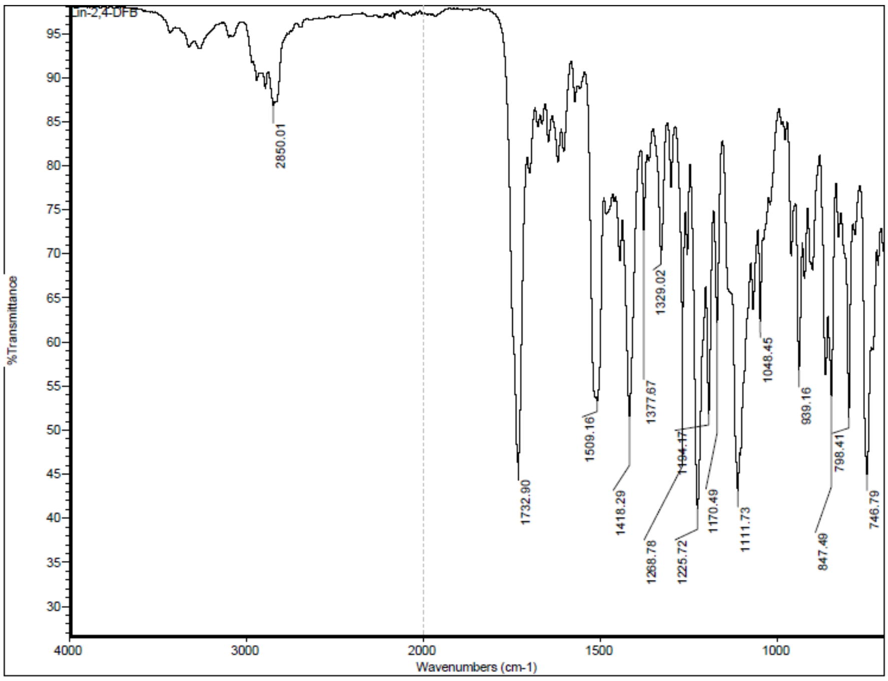
Lin-2,4-DFB
%Transmittance
Wavenumbers (cm-1)
2850.01
1732.90
1509.16
1418.29
1377.67
1329.02
1268.78
1194.17
1225.72
1170.49
1111.73
1048.45
939.16
847.49
798.41
746.79

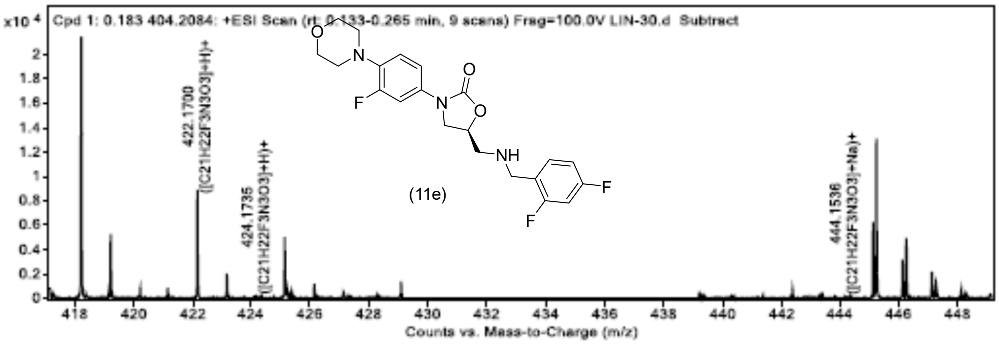
Cpd 1: 0.183 404.2084: +ESI Scan (rt: 0.133-0.265 min, 9 scans) Frag=100.0V LIN-30.d Subtract
x10 4
422.1700
([C21H22F3N3O3]+H)+
424.1735
([C21H22F3N3O3]+H)+
444.1536
([C21H22F3N3O3]+Na)+
(11e)
Counts vs. Mass-to-Charge (m/z)

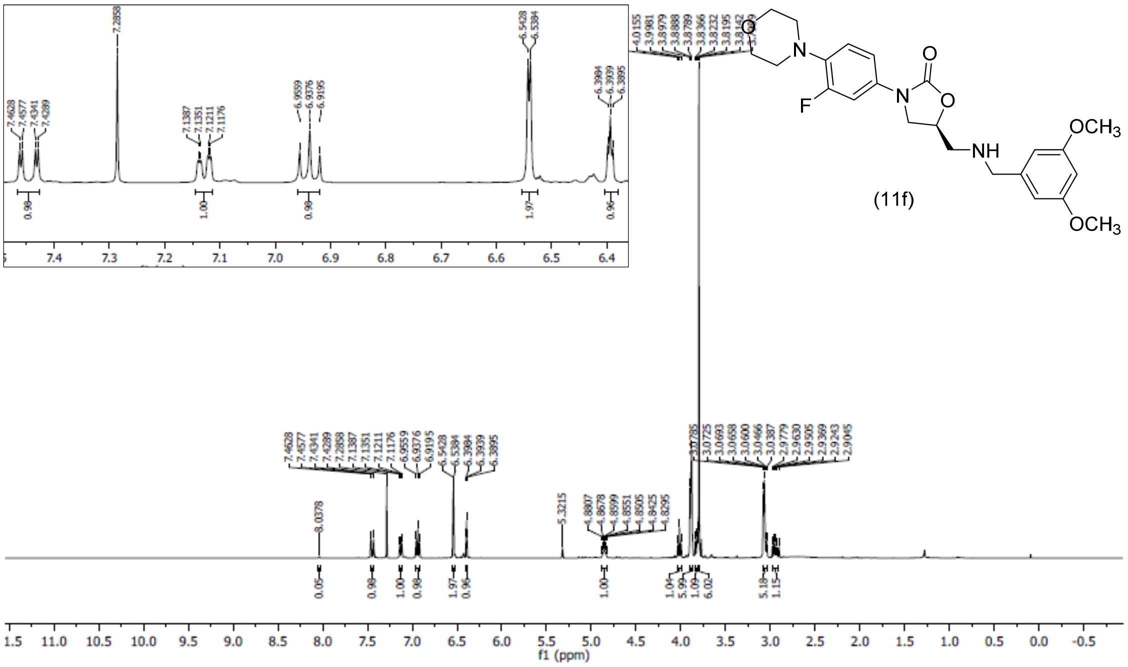
(11f)
f1 (ppm)

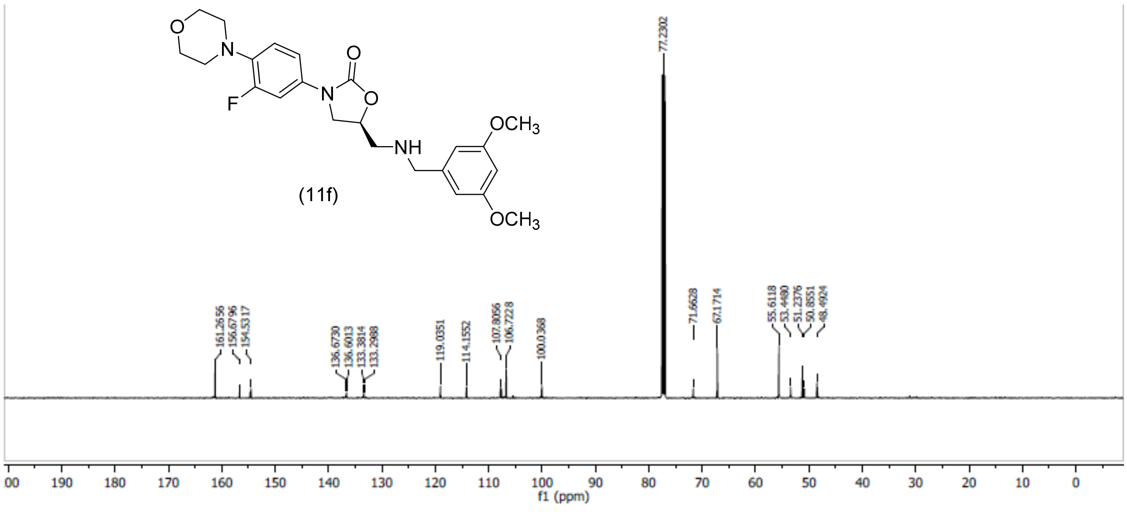
(11f)
f1 (ppm)

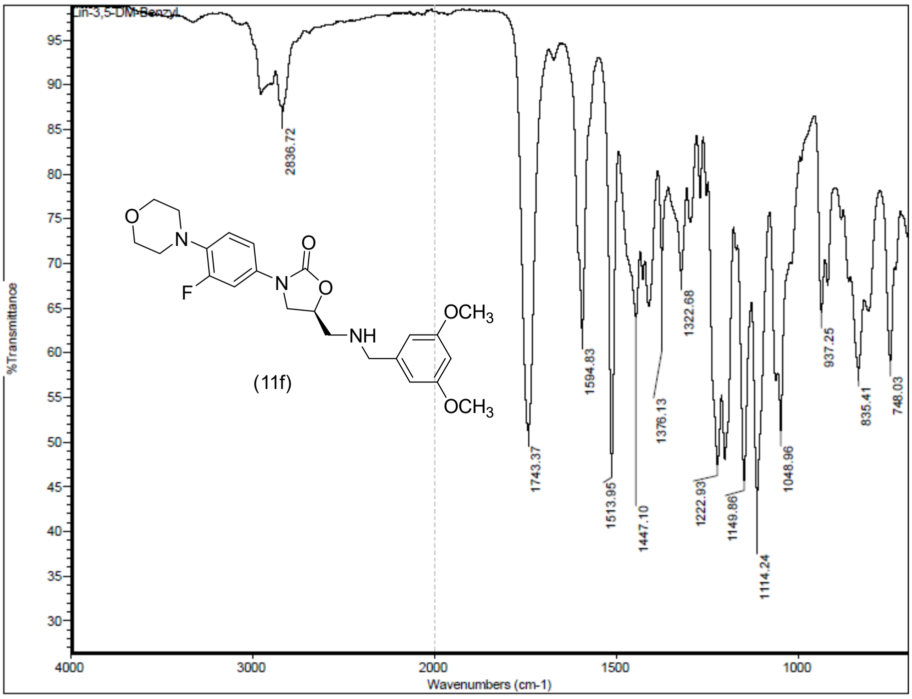

Lin-3,5-DM-Benzyl
%Transmittance
Wavenumbers (cm-1)
2836.72
1743.37
1594.83
1513.95
1447.10
1376.13
1322.68
1222.93
1149.86
1114.24
1048.96
937.25
835.41
748.03
(11f)

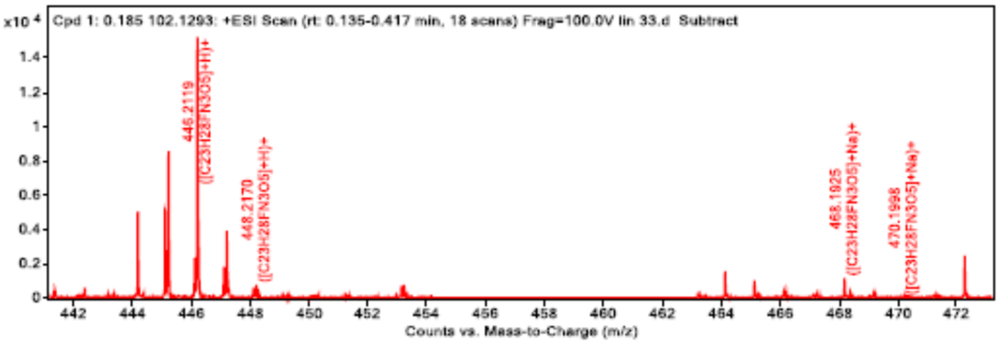

Cpd 1: 0.185 102.1293: +ESI Scan (rt: 0.135-0.417 min, 18 scans) Frag=100.0V lin 33.d Subtract
Counts vs. Mass-to-Charge (m/z)
446.2119 ([C23H28FN3O5]+H)+
448.2170 ([C23H28FN3O5]+H)+
468.1925 ([C23H28FN3O5]+Na)+
470.1998 ([C23H28FN3O5]+Na)+

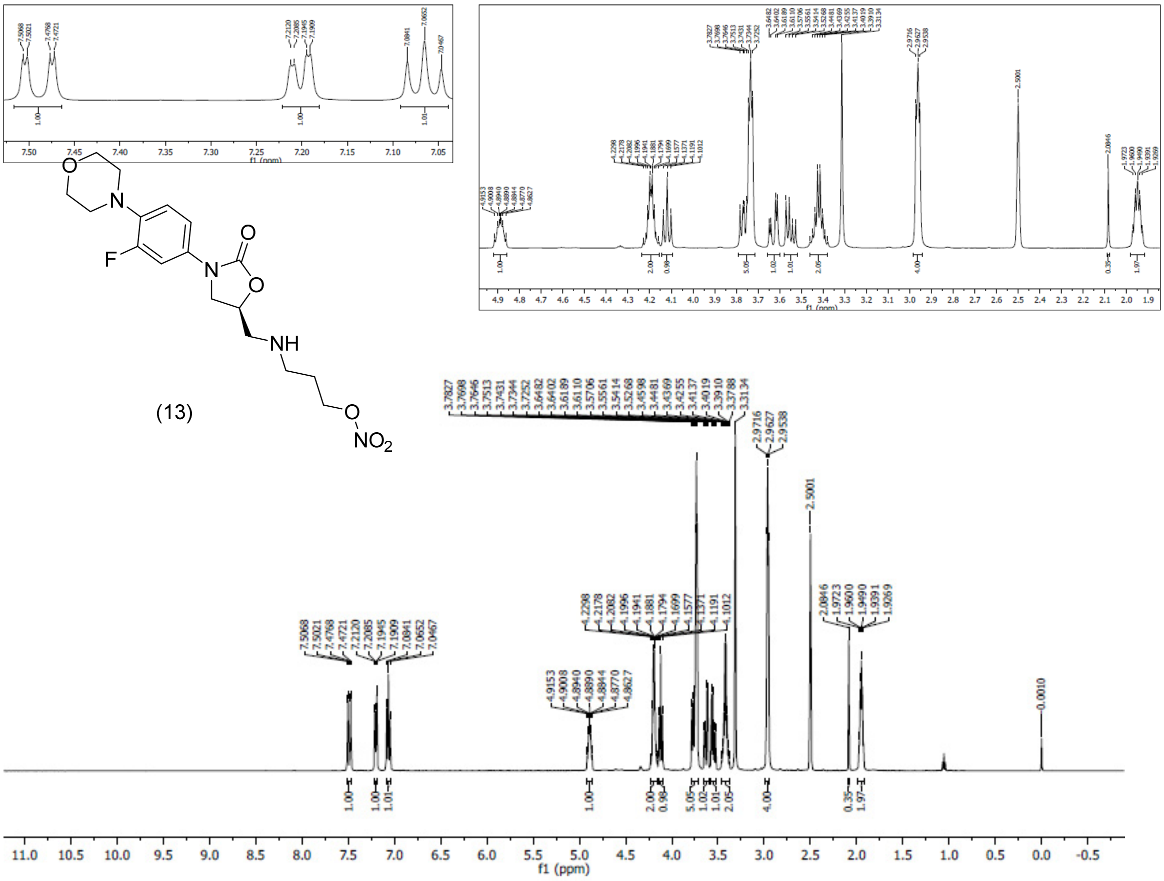

(13)

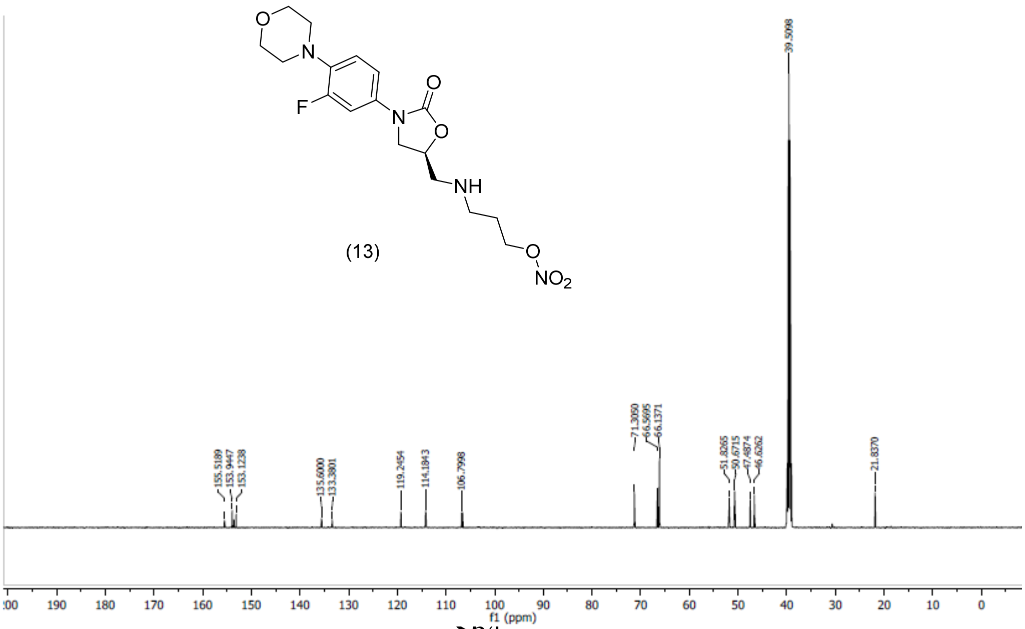

(13)

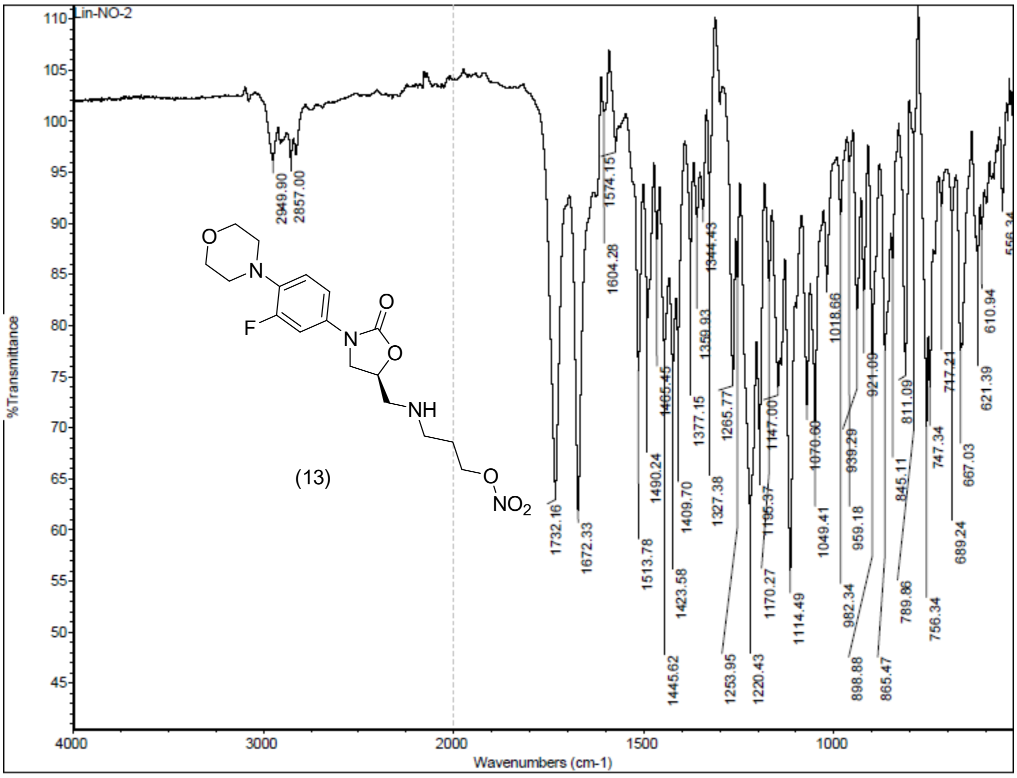

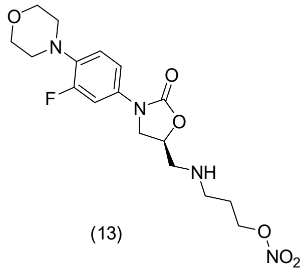

(13)

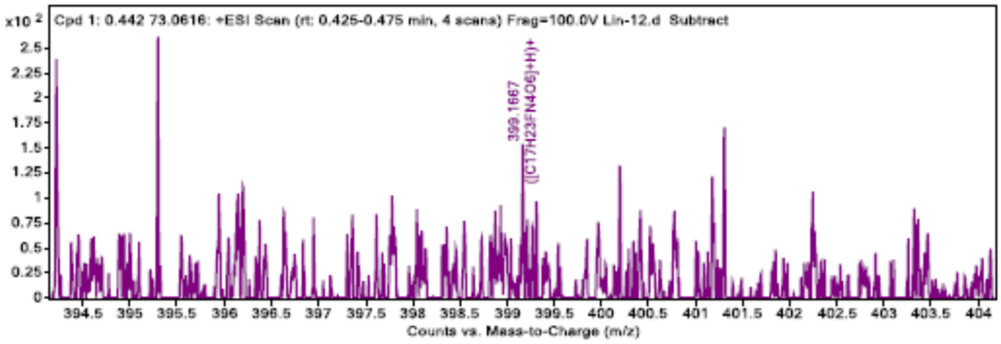

Cpd 1: 0.442 73.0616: +ESI Scan (rt: 0.425-0.475 min, 4 scans) Frag=100.0V Lin-12.d Subtract
399.1667
([C17H23FN4O6]+H)+
Counts vs. Mass-to-Charge (m/z)

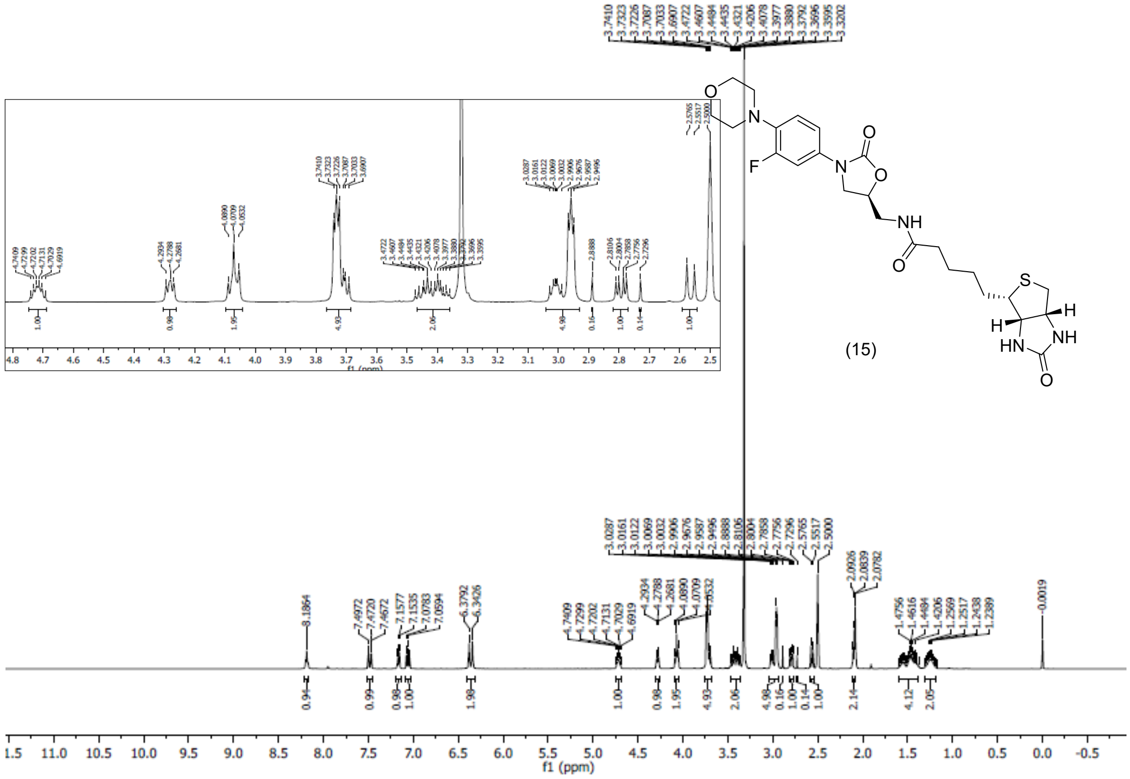
(15)
f1 (ppm)

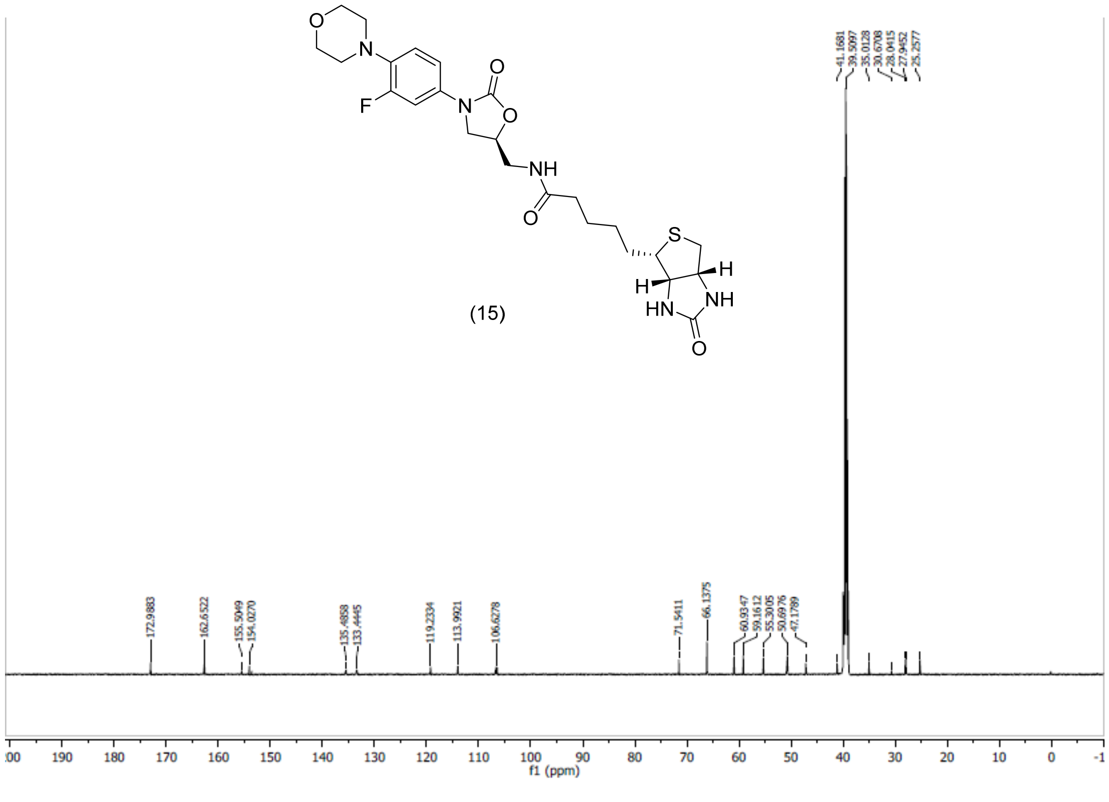
(15)
172.9883
162.6522
155.5049
154.0270
135.4858
133.4445
119.2334
113.9921
106.6278
71.5411
66.1375
60.9347
59.1612
55.3005
50.6976
47.1789
41.1681
39.5097
35.0128
30.6708
28.0415
27.9452
25.2577
f1 (ppm)

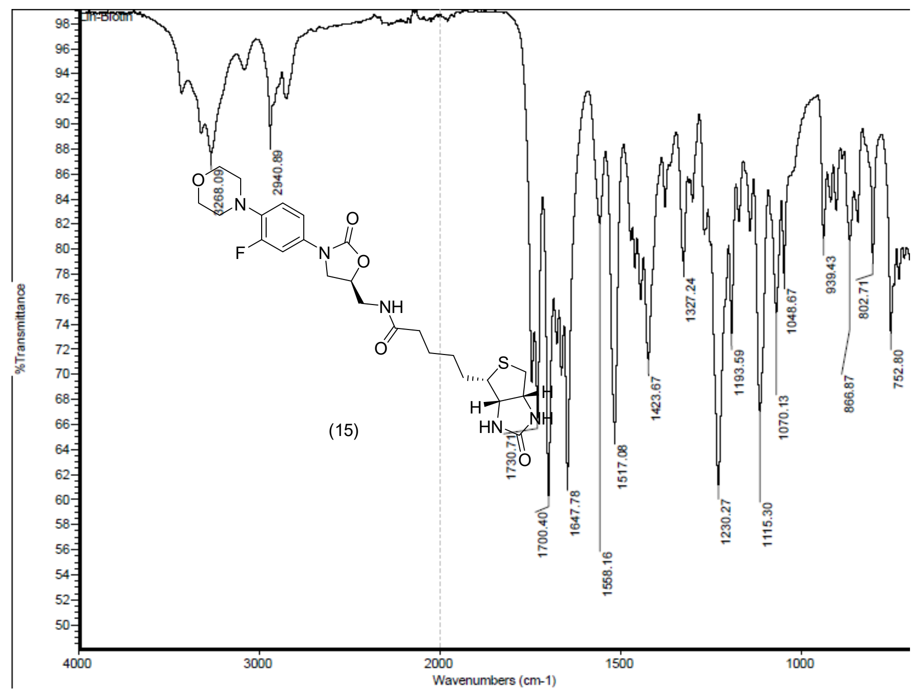
Lin-Biotin
%Transmittance
Wavenumbers (cm-1)
3268.09
2940.89
1730.71
1700.40
1647.78
1558.16
1517.08
1423.67
1327.24
1230.27
1193.59
1115.30
1070.13
1048.67
939.43
866.87
802.71
752.80
(15)

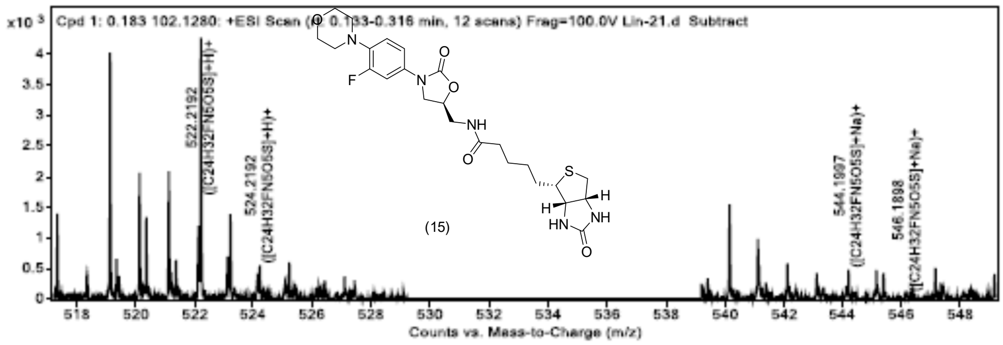
Cpd 1: 0.183 102.1280: +ESI Scan (0.133-0.316 min, 12 scans) Frag=100.0V Lin-21.d Subtract
x10 3
522.2192
([C24H32FN5O5S]+H)+
524.2192
([C24H32FN5O5S]+H)+
544.1997
([C24H32FN5O5S]+Na)+
546.1898
([C24H32FN5O5S]+Na)+
(15)
Counts vs. Mass-to-Charge (m/z)

Antiproliferative properties

The synthesized compounds were screened for their antiproliferative properties against RPE1 (normal human immortalized retinal pigment epithelial cell line) cell (in DMEM-F12 medium) to determine the toxicity towards normal cells relative to the cancer cell lines utilized by the standard mitochondrial dependent reduction of yellow MTT [3-(4,5-dimethylthiazol-2-yl)-2,5-diphenyl-tetrazolium bromide] to purple formazan technique [3]. Cells were suspended in the DMEM-F12 medium in addition to 1% antibiotic–antimycotic mixture (10000 μg ml^{-1} potassium penicillin, 10000 μg ml^{-1} streptomycin sulfate and 25 μg ml^{-1} amphotericin B), 10% fetal bovine serum and 1% L-glutamine at 37 °C, under 5% CO_2 and 95% humidity. Cells were seeded at concentration of 30000 cells per well in fresh complete growth medium in 96-well tissue culture microtiter plates for 24 h. Media was aspirated, fresh complete medium was added and cells were incubated with different concentrations of the tested compound to give a final concentration of [100, 50, 25 and 12.5 μM). 0.5% DMSO was used as negative control. Triplicate wells were prepared for each individual dose. After 72 h of incubation, medium was aspirated, 40 μl MTT salt (2.5 mg ml^{-1}) were added to each well and incubated for further 4 h at 37 °C. To stop the reaction and dissolve the formed crystals, 150 μl of 10% sodium dodecyl sulfate (SDS) in deionized water were added to each well and incubated overnight at 37 °C. The absorbance was then measured at 570 nm and a reference wavelength of 595 nm.

Data were collected as mean values for experiments performed in triplicates for each individual dose which had been measured by MTT assay. Control experiments did not exhibit significant change compared to the DMSO vehicle. The cell surviving fraction was calculated according to equ. (1).

$$\textit{Surviving fraction} = \frac{\textit{Optical density } (O.D.) \textit{of treated cells}}{O.D. \textit{of control cells}} \quad \ldots\ldots\ldots\ldots\ldots (1)$$

Graph-Pad PRISM version-5 software was used for the needed IC_{50} (concentration required to produce 50% inhibition of cell growth compared to the control experiment) . Statistical calculations for determination of the mean and standard error values were determined by SPSS 16 software. The observed anti-proliferative properties are presented in Supplementary Fig. S1.

QSAR studies

The geometry of the synthesized agents (**3a-k**, **5af, 7a-c**, **9a-c**, **11a-f**, **13** and **15**) revealing variable antimicrobial properties against the Gram-positive bacteria tested (*S. aureus* and *B. subtilis*) were optimized by the molecular mechanics force field (MM^+), followed by semi-empirical AM1 method implemented in the HyperChem 8.0 package (http://www.hyper.com). The structures were fully optimized without constraining any parameters, thus bringing all geometric variables to their equilibrium values. The energy minimization protocol employed the Polak–Ribiere conjugated gradient algorithm [1, 2]. Convergence to a local minimum was achieved when the energy gradient was ≤0.01 kcal mol^{-1}. The RHF (Restricted Hartree–Fock) method was used in the spin pairing for the semi-empirical tool.

2D-QSAR studies were undertaken to utilize the comprehensive descriptors for structural and statistical analysis (CODESSA-Pro) software [3, 4]. The optimized structures of the bio-active compounds were uploaded to CODESSA-Pro that includes MOPAC capability for the final geometry optimization. CODESSA-Pro calculated 794 molecular descriptors (constitutional, topological, geometrical, charge-related, semi-empirical, thermodynamical, molecular-type, atomic-type and bond-type descriptors) for the exported bio-active agents. Different mathematical transformations [including property (in MIC, μM), 1/property, log(property) and 1/log(property)] of the experimentally observed activity of the set compounds were utilized searching for the best QSAR model. The best multi-linear regression (BMLR) technique was utilized which is a stepwise search for the best *n* parameter regression equations (where *n* stands for the number of descriptors used), based on the highest R^2 (squared correlation coefficient), R^2cvOO (squared cross-validation “leave-one-out, LOO” coefficient), R^2cvMO (squared cross-validation “leave-many-out, LMO” coefficient), *F* (Fisher statistical significance criteria) values, and *s* (standard deviation). The QSAR models were generated (obeying the thumb rule, which determines a reasonable ratio between the data points and the number of QSAR descriptor) (Tables S1–S6, Figs. S2, S3).

Pharmacophoric modeling

The 3D-pharmocphoric modeling was undertaken for the synthesized agents (**3a-k**, **5af, 7a-c**, **9a-c**, **11a-f**, **13** and **15**) revealing variable antimicrobial properties against the Gram-positive bacteria tested (*S. aureus* and *B. subtilis*) by the Discovery Studio 2.5 software adopting the standard technique [5, 6].

References

1- Seliem, I. A.; Panda, S. S.; Girgis, A. S.; Moatasim, Y.; Kandeil, A.; Mostafa, A.; Ali, M. A.; Nossier, E. S.; Rasslan, F.; Srour, A. M.; Sakhuja, R.; Ibrahim, T. S.; Abdel-samii, Z. K. M.; Al-Mahmoudy, A. M. M. New quinoline-triazole conjugates: Synthesis, and antiviral properties against SARS-CoV-2, Bioorg. Chem. 2021, 114, 105117. doi.org/10.1016/j.bioorg.2021.105117

2- Srour, A. M.; Panda, S. S.; Mostafa, A.; Fayad, W.; El-Manawaty, M. A.; Soliman, A. A. F.; Moatasim, Y.; El Taweel, A.; Abdelhameed, M. F.; Bekheit, M. S.; Ali, M. A.; Girgis, A. S. Synthesis of aspirin-curcumin mimic conjugates of potential antitumor and anti-SARS-CoV-2 properties, Bioorg. Chem. 2021, 117, 105466. https://doi.org/10.1016/j.bioorg.2021.105466

3- Girgis, A. S.; Tala, S. R.; Oliferenko, P. V.; Oliferenko, A. A.; Katritzky, A. R. Computer-assisted rational design, synthesis, and bioassay of nonsteroidal anti-inflammatory agents, Eur. J. Med. Chem. 2012, 50, 1–8. doi:10.1016/j.ejmech.2011.11.034

4- Ghanim, A. M.; Girgis, A. S.; Kariuki, B. M.; Samir, N.; Said, M. F.; Abdelnaser, A.; Nasr, S.; Bekheit, M. S.; Abdelhameed, M. F.; Almalki, A. J.; Ibrahim, T. S.; Panda, S. S. Design and synthesis of ibuprofen-quinoline conjugates as potential anti-inflammatory and analgesic drug candidates, Bioorg. Chem. 2022, 119, 105557. doi.org/10.1016/j.bioorg.2021.105557

5- Youssef, M. A.; Panda, S. S.; El-Shiekh, R. A.; Shalaby, E. M.; Aboshouk, D. R.; Fayad, W.; Fawzy, N. G.; Girgis, A. S. Synthesis and molecular modeling studies of cholinesterase inhibitor dispiro[indoline-3,2'-pyrrolidine-3',3''-pyrrolidines], RSC Adv. 2020, 10, 21830–21838. DOI: 10.1039/d0ra03064c

6- Aziz, M. N.; Panda, S. S.; Shalaby, E. M.; Fawzy, N. G.; Girgis, A. S. Facile synthetic approach towards vasorelaxant active 4-hydroxyquinazoline-4-carboxamides, RSC Adv. 2019, 9, 28534–28540. DOI: 10.1039/c9ra04321g

Table S1. Descriptors of the QSAR model for the tested agents against *S. aureus*.

Entry	ID	Coefficient	*s*	*t*	Descriptor
1	0	74.0004	5.874	12.597	Intercept
2	D_1	3.29174	0.417	7.890	Partial surface area for atom H
3	D_2	0.00087934	0.0001	6.954	DPSA2 difference in CPSAs (PPSA2-PNSA2) (Zefirov PC)
4	D_3	-0.426925	0.083	-5.124	HOMO energy
5	D_4	-0.475568	0.036	-13.155	Max. e-e repulsion for bond C-N
$N = 31$, $n = 4$, $R^2 = 0.926$, R^2cvOO = 0.898, R^2cvMO = 0.903, $F = 81.973$, $s^2 = 0.010$ log(MIC, μM) = 74.0004 + (3.29174 x D_1) + (0.00087934 x D_2) – (0.426925 x D_3) – (0.475568 x D_4)					

Table S2. Observed and estimated antimicrobial properties for the tested compounds agents against *S. aureus* according to the BMLR-QSAR model.

Entry	Compd.	log(observed MIC, μM)	Observed MIC, μM	log(estimated MIC, μM)	Estimated MIC, μM	Error*
1	**3a**	1.51704	32.888	1.54496	35.072	-2.184
2	**3b**	1.80572	63.932	1.66028	45.738	18.194
3	**3c**	1.78204	60.54	1.91449	82.128	-21.588
4	**3d**	1.75647	57.078	1.92461	84.064	-26.986
5	**3e**	1.74425	55.495	1.69024	49.005	6.490
6	**3f**	1.84955	70.721	1.75121	56.391	14.330
7	**3g**	1.83629	68.594	1.77841	60.036	8.558
8	**3h**	1.81093	64.704	1.73129	53.863	10.841
9	**3i**	1.78365	60.765	1.78526	60.990	-0.225
10	**3j**	2.07169	117.948	2.03436	108.233	9.715
11	**3k**	1.79878	62.919	1.80741	64.182	-1.263
12	**5a**	1.00065	10.015	1.16152	14.505	-4.490
13	**5b**	0.981501	9.583	1.01677	10.394	-0.811
14	**5c**	1.26574	18.439	1.30165	20.029	-1.590
15	**5d**	0.653213	4.5	0.79323	6.212	-1.712
16	**5e**	0.970161	9.336	0.977633	9.498	-0.162
17	**5f**	1.22287	16.706	1.12631	13.375	3.331
18	**7a**	1.3006	19.98	1.29432	19.693	0.287
19	**7b**	1.3006	19.98	1.32712	21.238	-1.258
20	**7c**	1.29951	19.93	1.16767	14.712	5.218
21	**9a**	1.79371	62.189	1.78847	61.443	0.746
22	**9b**	1.80572	63.932	1.96232	91.690	-27.758
23	**9c**	1.76591	58.332	1.75686	57.129	1.203
24	**11a**	1.89938	79.32	1.84876	70.593	8.727
25	**11b**	1.83908	69.037	1.8294	67.515	1.522
26	**11c**	1.84863	70.572	1.91082	81.437	-10.865

27	**11d**	1.88202	76.212	1.84101	69.344	6.868
28	**11e**	1.88044	75.934	1.7744	59.484	16.450
29	**11f**	1.85631	71.831	2.05724	114.088	-42.257
30	**13**	1.90484	80.323	1.77181	59.130	21.193
31	**15**	1.78781	61.349	1.68528	48.448	12.901

*Error is the difference between the observed and estimated property (MIC, μM).

Table S3. Molecular descriptor values of the QSAR model for the tested compounds against *S. aureus*.

Entry	Compd	Descriptors*			
		D_1	D_2	D_3	D_4
1	**3a**	0.56246	1062.344	-8.611	165.9433
2	**3b**	0.61536	1124.793	-8.81	166.3611
3	**3c**	0.65382	1235.027	-8.942	166.4151
4	**3d**	0.65641	1128.694	-8.653	165.9557
5	**3e**	0.58863	1308.602	-8.31	166.0041
6	**3f**	0.67343	1075.835	-8.727	166.4068
7	**3g**	0.67728	1079.986	-8.544	166.2197
8	**3h**	0.68871	1188.451	-8.41	166.4781
9	**3i**	0.62159	1280.543	-8.697	166.328
10	**3j**	0.6916	1250.482	-8.486	166.0438
11	**3k**	0.69999	1272.396	-8.528	166.6573
12	**5a**	0.56527	763.6985	-8.581	166.1899
13	**5b**	0.52992	803.3015	-8.769	166.4916
14	**5c**	0.55014	752.541	-9.074	166.2125
15	**5d**	0.46488	847.5723	-8.532	166.3806
16	**5e**	0.61032	860.5664	-8.421	166.9239
17	**5f**	0.48445	862.5449	-8.449	165.7688
18	**7a**	0.56002	750.3674	-9.02	166.2438
19	**7b**	0.58567	746.1119	-9.012	166.3373
20	**7c**	0.53307	794.0137	-8.598	166.0254
21	**9a**	0.61856	1264.514	-8.977	166.522
22	**9b**	0.61312	1116.492	-8.556	165.4671
23	**9c**	0.57685	1327.415	-8.926	166.3703
24	**11a**	0.59535	753.0491	-8.863	165.1865
25	**11b**	0.58403	898.7879	-8.604	165.1858
26	**11c**	0.54741	898.6125	-9.136	165.2384

27	**11d**	0.57174	760.4601	-8.531	164.755
28	**11e**	0.54952	852.5845	-8.45	164.8389
29	**11f**	0.64039	956.7063	-8.543	165.1492
30	**13**	0.56125	757.8262	-8.999	165.2432
31	**15**	0.62826	1147.559	-9.028	166.6356

*D_1 = Partial surface area for atom H, D_2 = DPSA2 difference in CPSAs (PPSA2-PNSA2) (Zefirov PC), D_3 = HOMO energy, D_3 = Max. e-e repulsion for bond C-N.

Table S4. Descriptors of the QSAR model for the tested agents against *B. subtilis*.

Entry	ID	Coefficient	*s*	*t*	Descriptor
1	0	94.2625	5.572	16.918	Intercept
2	D_1	-2.09499	0.542	-3.867	Min. total interaction for bond H-C
3	D_2	-2.13915	0.193	-11.112	Min. total interaction for bond C-C
4	D_3	-0.272268	0.021	-13.130	Max. e-e repulsion for atom N
$N = 31$, $n = 3$, $R^2 = 0.935$, R^2cvOO = 0.915, R^2cvMO = 0.916, $F = 129.818$, $s^2 = 0.024$<br>log(MIC, μM) = 94.2625 – (2.09499 x D_1) – (2.13915 x D_2) – (0.272268 x D_3)					

Table S5. Observed and estimated antimicrobial properties for the tested compounds agents against *B. subtilis* according to the BMLR-QSAR model.

Entry	Compd.	log(observed MIC, μM)	Observed MIC, μM	log(estimated MIC, μM)	Estimated MIC, μM	Error*
1	**3a**	0.914977	8.222	0.97142	9.363	-1.141
2	**3b**	1.80572	63.932	1.62406	42.078	21.854
3	**3c**	1.78204	60.54	1.86703	73.626	-13.086
4	**3d**	1.75647	57.078	1.99012	97.751	-40.673
5	**3e**	1.74425	55.495	1.81555	65.396	-9.901
6	**3f**	1.24748	17.68	0.960204	9.124	8.556
7	**3g**	1.83629	68.594	1.94528	88.162	-19.568
8	**3h**	1.81093	64.704	1.62283	41.959	22.745
9	**3i**	1.78365	60.765	1.60249	40.040	20.725
10	**3j**	1.77066	58.974	1.92266	83.687	-24.713
11	**3k**	1.79878	62.919	1.8159	65.449	-2.530
12	**5a**	0.398634	2.504	0.61845	4.154	-1.650
13	**5b**	0.379487	2.396	0.470247	2.953	-0.557
14	**5c**	0.663701	4.61	0.59618	3.946	0.664
15	**5d**	0.352183	2.25	0.525078	3.350	-1.100
16	**5e**	0.368101	2.334	0.482452	3.037	-0.703
17	**5f**	0.921842	8.353	0.758413	5.733	2.620
18	**7a**	0.698535	4.995	0.664147	4.615	0.380
19	**7b**	0.698535	4.995	0.699902	5.011	-0.016
20	**7c**	0.697491	4.983	0.755404	5.694	-0.711
21	**9a**	1.79371	62.189	1.53791	34.507	27.682
22	**9b**	1.80572	63.932	1.75933	57.455	6.477
23	**9c**	1.76591	58.332	1.74688	55.832	2.500
24	**11a**	1.89938	79.32	1.77818	60.004	19.316
25	**11b**	1.83908	69.037	1.79938	63.006	6.031
26	**11c**	1.5476	35.286	1.77752	59.913	-24.627

27	**11d**	1.88202	76.212	1.94775	88.665	-12.453
28	**11e**	1.88044	75.934	1.97908	95.297	-19.363
29	**11f**	1.85631	71.831	1.75066	56.320	15.511
30	**13**	1.90484	80.323	1.68596	48.524	31.799
31	**15**	1.78781	61.349	1.92212	83.583	-22.234

*Error is the difference between the observed and estimated property (MIC, μM).

Table S6. Molecular descriptor values of the QSAR model for the tested compounds against *B. subtilis*.

Entry	Compd.	Descriptors*		
		D_1	D_2	D_3
1	**3a**	11.9931	13.6296	143.2774
2	**3b**	11.9319	13.1962	144.7564
3	**3c**	11.9393	13.1742	143.9799
4	**3d**	11.8739	13.2499	143.4363
5	**3e**	11.982	13.2244	143.446
6	**3f**	12.037	13.5018	143.9849
7	**3g**	11.9303	13.1653	143.8317
8	**3h**	11.9719	13.2406	144.1043
9	**3i**	11.9889	13.242	144.0372
10	**3j**	11.9032	13.2262	143.6448
11	**3k**	11.904	13.1915	144.3034
12	**5a**	12.0614	13.6487	143.8982
13	**5b**	12.0794	13.6499	144.2946
14	**5c**	12.0608	13.6514	143.9634
15	**5d**	12.0765	13.6532	144.0896
16	**5e**	12.0354	13.635	144.7054
17	**5f**	12.0674	13.6468	143.3529
18	**7a**	12.0582	13.6432	143.7982
19	**7b**	12.0488	13.6271	143.8657
20	**7c**	12.0598	13.6459	143.4295
21	**9a**	11.9042	13.3264	144.263
22	**9b**	11.8112	13.4505	143.1903
23	**9c**	11.9185	13.252	143.97
24	**11a**	12.0122	13.6386	140.0966
25	**11b**	11.9892	13.6664	139.9773
26	**11c**	12.0073	13.651	140.0393

27	**11d**	11.9629	13.6425	139.8225
28	**11e**	11.9663	13.637	139.7245
29	**11f**	12.0011	13.6443	140.2383
30	**13**	12.0378	13.5998	140.5432
31	**15**	12.0412	12.9446	144.7974

*D_1 = Min. total interaction for bond H-C, D_2 = Min. total interaction for bond C-C, D_3 = Max. e-e repulsion for atom N.

Table S7. Observed and estimated activity values for the tested compounds against *S. aureus* according to the 3D-pharmacophore model.

Entry	Compd.	Observed log(MIC, μM)	Estimated log(MIC, μM)	Fit value
1	**3a**	1.51704	1.84098	5.6784
2	**3b**	1.80572	1.88449	5.6682
3	**3c**	1.78204	1.37375	5.8055
4	**3d**	1.75647	2.01402	5.6393
5	**3e**	1.74425	1.7901	5.6905
6	**3f**	1.84955	1.74855	5.7007
7	**3g**	1.83629	1.78017	5.6929
8	**3h**	1.81093	1.57704	5.7456
9	**3i**	1.78365	1.58182	5.7442
10	**3j**	2.07169	2.19801	5.6014
11	**3k**	1.79878	1.53599	5.7570
12	**5a**	1.00065	1.23937	5.8502
13	**5b**	0.98150	1.37555	5.8049
14	**5c**	1.26574	1.50804	5.7650
15	**5d**	0.65321	1.18984	5.8679
16	**5e**	0.97016	1.30382	5.8282
17	**5f**	1.22287	1.61247	5.7359
18	**7a**	1.30060	1.30122	5.8291
19	**7b**	1.30060	1.28669	5.8339
20	**7c**	1.29951	1.20052	5.8640
21	**9a**	1.79371	2.14546	5.6119
22	**9b**	1.80572	2.09218	5.6228
23	**9c**	1.76591	1.6994	5.7131
24	**11a**	1.89938	1.29709	5.8304
25	**11b**	1.83908	1.60747	5.7373
26	**11c**	1.84863	1.35055	5.8129

27	**11d**	1.88202	1.31817	5.8234
28	**11e**	1.88044	1.64403	5.7275
29	**11f**	1.85631	1.4571	5.7799
30	**13**	1.90484	1.64206	5.7280
31	**15**	1.78781	1.56436	5.7491

Table S8. Observed and estimated activity values for the tested compounds against *B. subtilis* according to the 3D-pharmacophore model.

Entry	Compd.	Observed log(MIC, μM)	Estimated log(MIC, μM)	Fit value
1	**3a**	0.91498	0.75804	6.5619
2	**3b**	1.80572	0.81487	6.5305
3	**3c**	1.78204	1.33123	6.3174
4	**3d**	1.75647	1.09856	6.4008
5	**3e**	1.74425	2.36054	6.0686
6	**3f**	1.24748	1.50589	6.2638
7	**3g**	1.83629	2.10012	6.1194
8	**3h**	1.81093	1.36447	6.3066
9	**3i**	1.78365	1.14237	6.3838
10	**3j**	1.77066	1.71941	6.2062
11	**3k**	1.79878	1.88041	6.1674
12	**5a**	0.39863	0.65381	6.6262
13	**5b**	0.37949	0.72662	6.5803
14	**5c**	0.66370	1.15583	6.3787
15	**5d**	0.35218	0.57343	6.6831
16	**5e**	0.36810	0.32045	6.9359
17	**5f**	0.92184	0.80520	6.5357
18	**7a**	0.69854	1.37928	6.3020
19	**7b**	0.69854	0.67930	6.6095
20	**7c**	0.69749	1.02594	6.4305
21	**9a**	1.79371	1.27904	6.3347
22	**9b**	1.80572	2.08334	6.1228
23	**9c**	1.76591	1.16705	6.3745
24	**11a**	1.89938	1.83034	6.1791
25	**11b**	1.83908	2.08298	6.1229
26	**11c**	1.54760	2.25552	6.0884

27	**11d**	1.88202	2.85518	5.9860
28	**11e**	1.88044	1.03876	6.4251
29	**11f**	1.85631	1.24322	6.3471
30	**13**	1.90484	1.49658	6.2665
31	**15**	1.78781	1.45831	6.2778

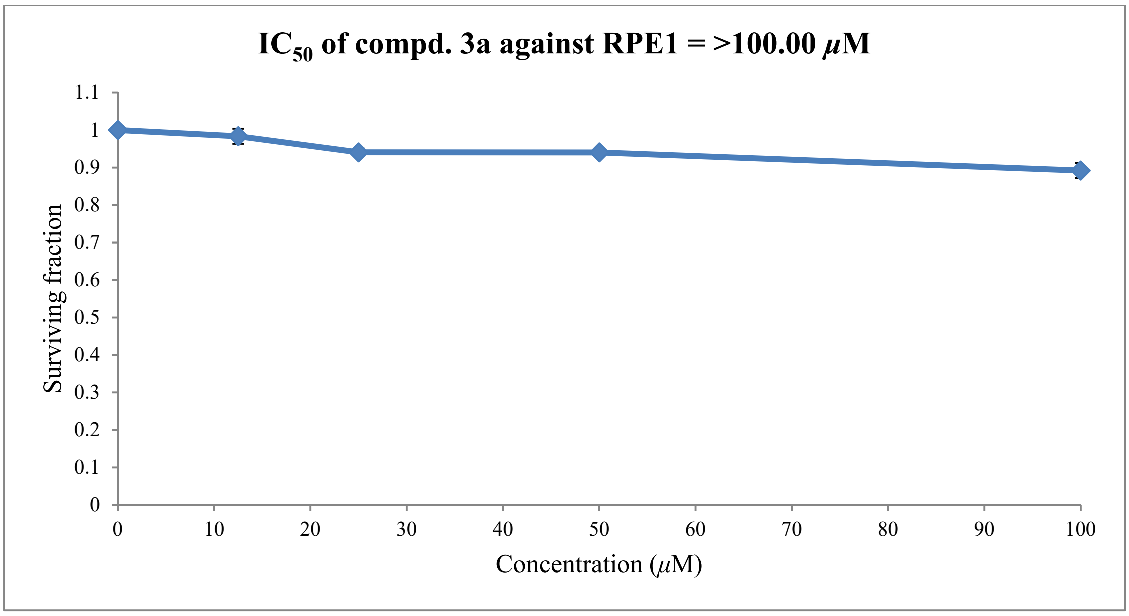
IC50 of compd. 3a against RPE1 = >100.00 μM
Surviving fraction
Concentration (μM)
1.1
1
0.9
0.8
0.7
0.6
0.5
0.4
0.3
0.2
0.1
0
0
10
20
30
40
50
60
70
80
90
100

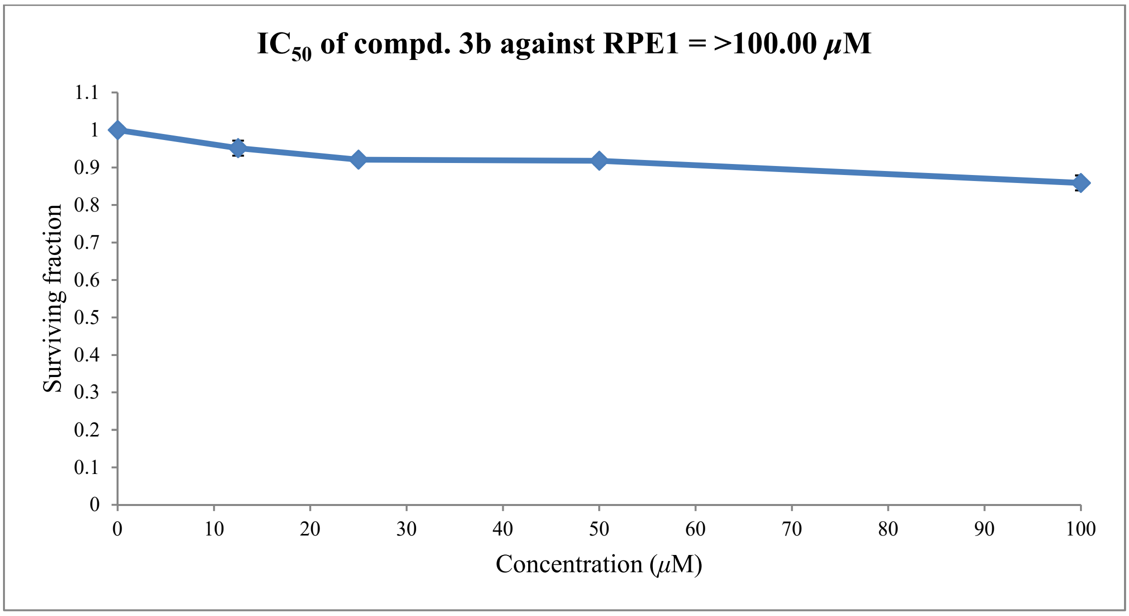
IC50 of compd. 3b against RPE1 = >100.00 μM
Surviving fraction
Concentration (μM)
1.1
1
0.9
0.8
0.7
0.6
0.5
0.4
0.3
0.2
0.1
0
0
10
20
30
40
50
60
70
80
90
100

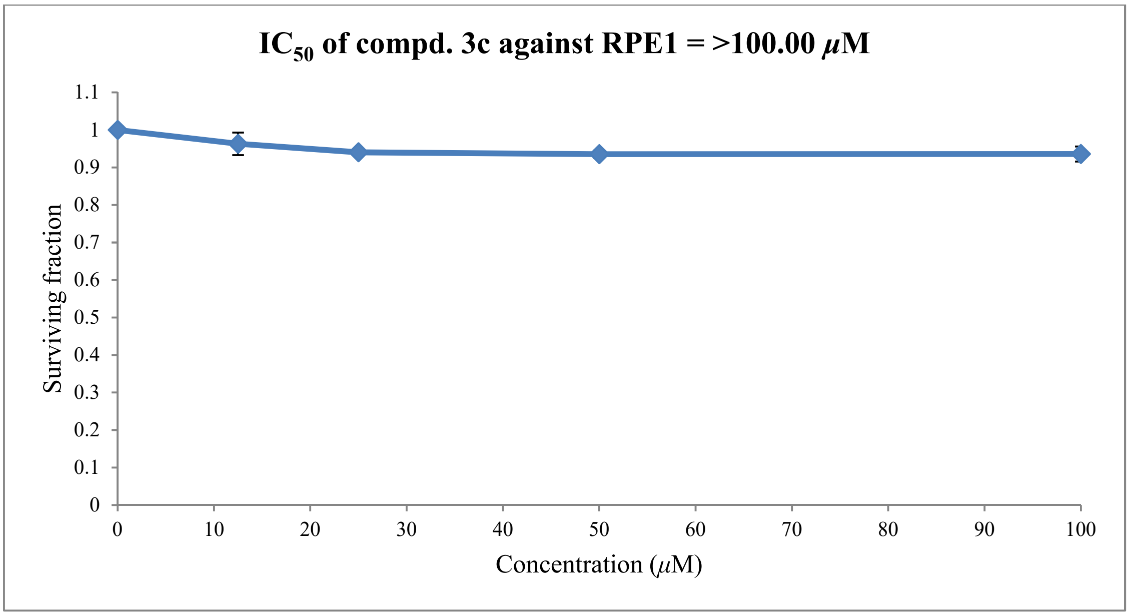
IC50 of compd. 3c against RPE1 = >100.00 μM
Surviving fraction
1.1
1
0.9
0.8
0.7
0.6
0.5
0.4
0.3
0.2
0.1
0
0
10
20
30
40
50
60
70
80
90
100
Concentration (μM)

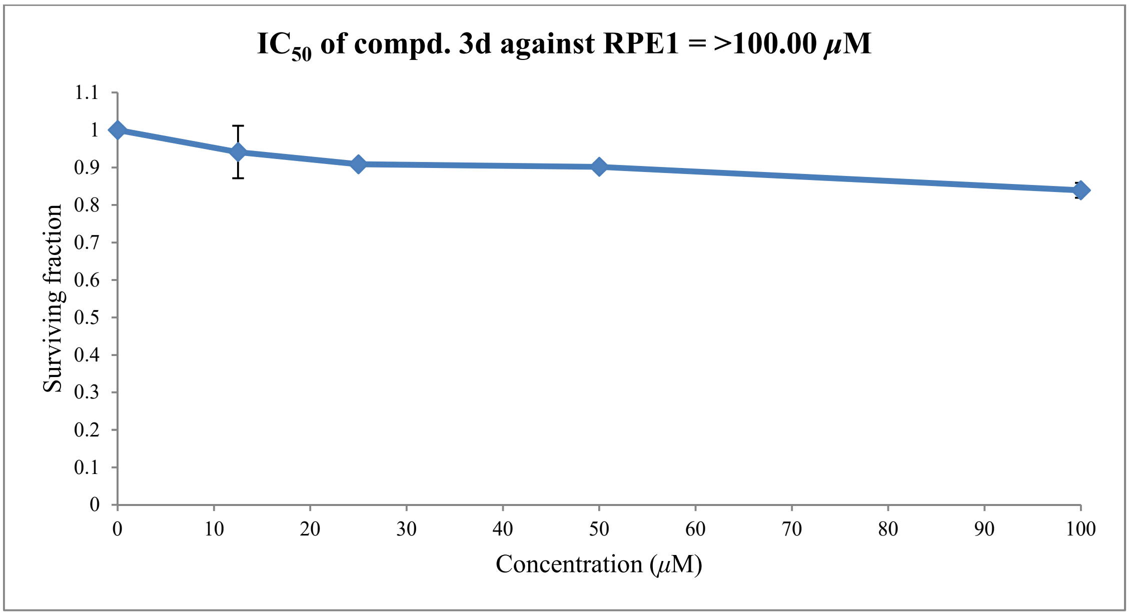
IC50 of compd. 3d against RPE1 = >100.00 μM
Surviving fraction
1.1
1
0.9
0.8
0.7
0.6
0.5
0.4
0.3
0.2
0.1
0
0
10
20
30
40
50
60
70
80
90
100
Concentration (μM)

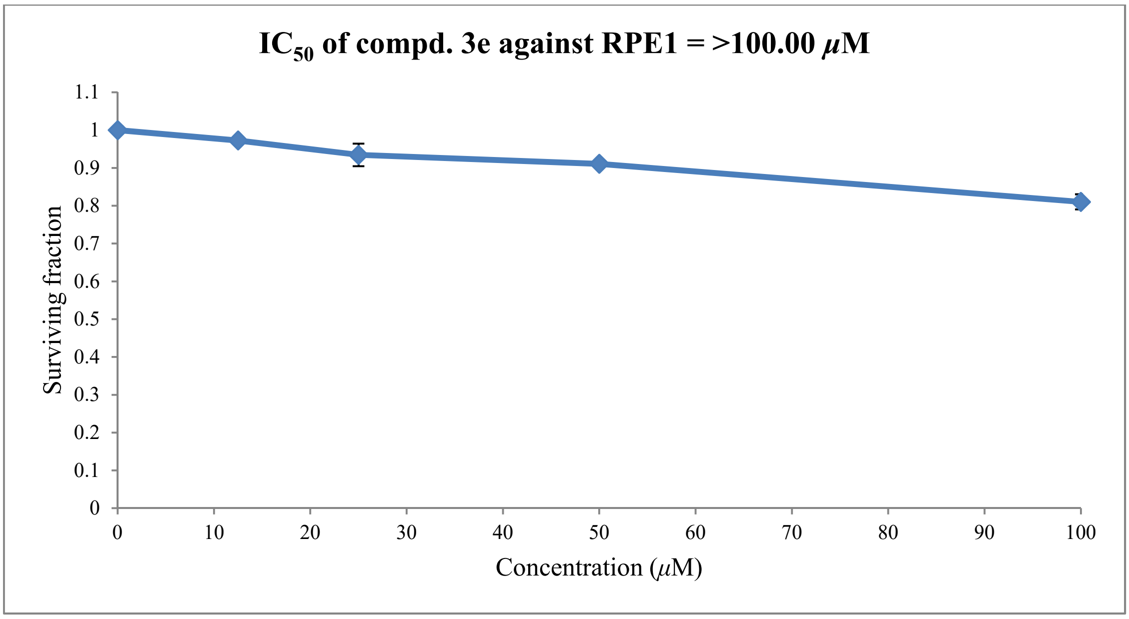

IC50 of compd. 3e against RPE1 = >100.00 μM
Surviving fraction
Concentration (μM)

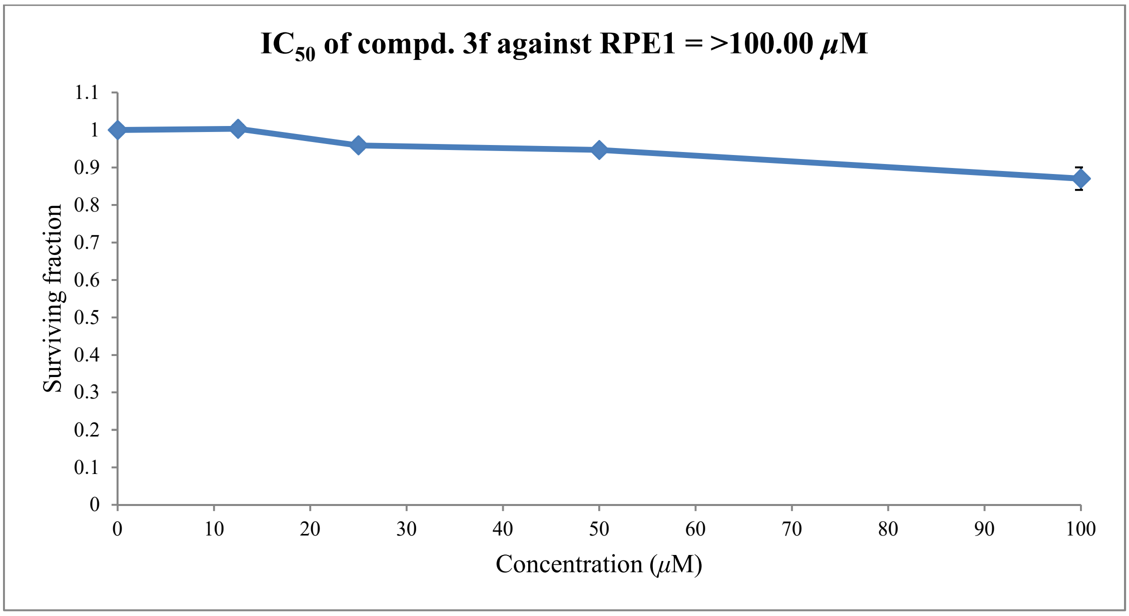

IC50 of compd. 3f against RPE1 = >100.00 μM
Surviving fraction
Concentration (μM)

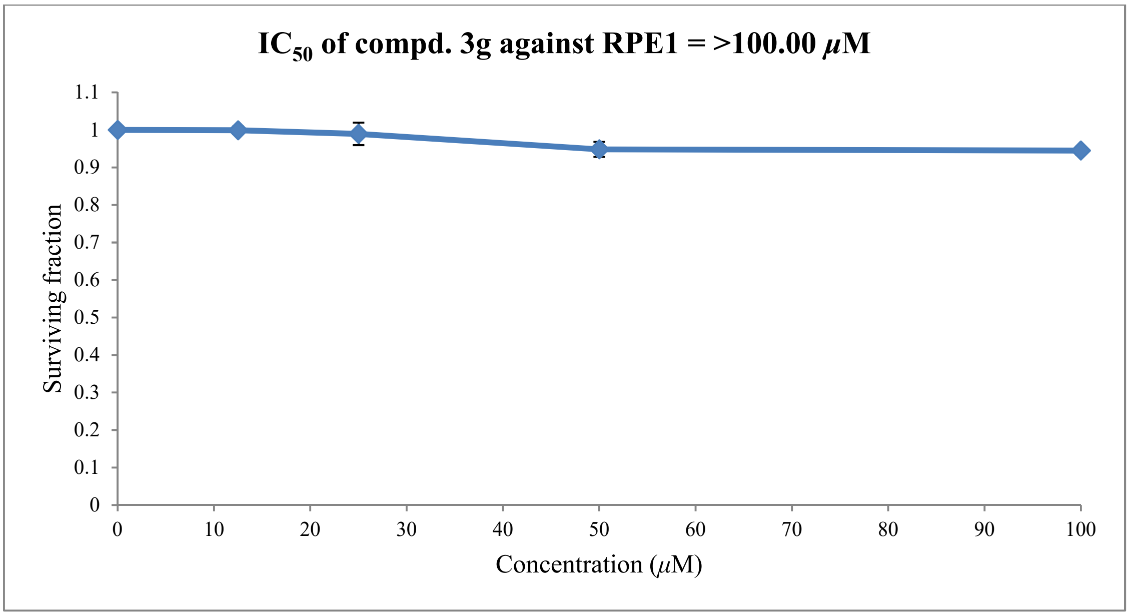

IC50 of compd. 3g against RPE1 = >100.00 μM
Surviving fraction
Concentration (μM)
0
0.1
0.2
0.3
0.4
0.5
0.6
0.7
0.8
0.9
1
1.1
0
10
20
30
40
50
60
70
80
90
100

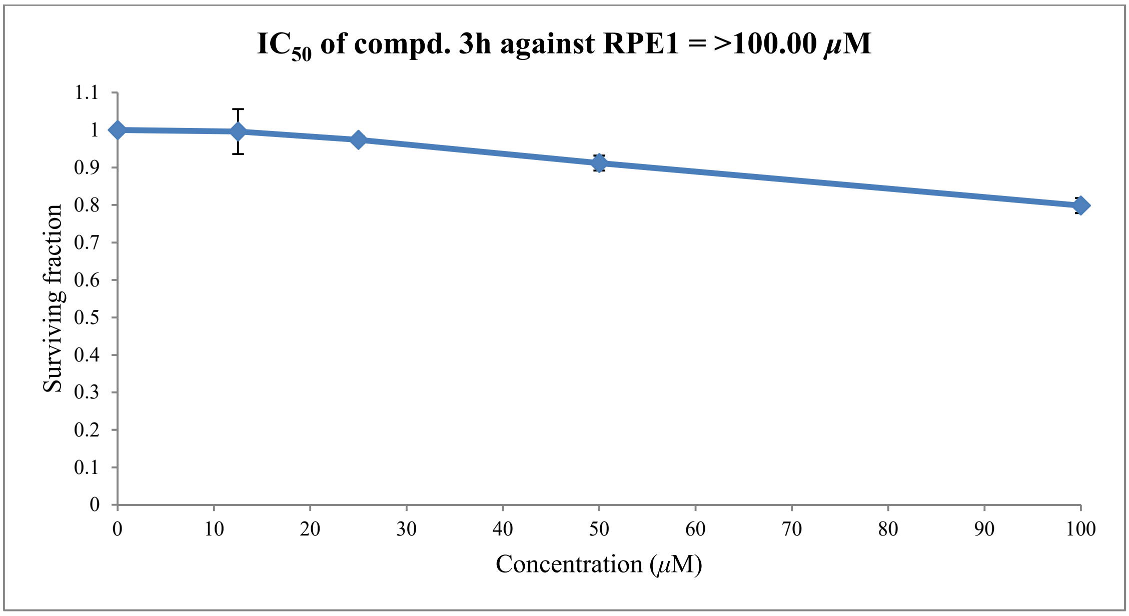

IC50 of compd. 3h against RPE1 = >100.00 μM
Surviving fraction
Concentration (μM)
0
0.1
0.2
0.3
0.4
0.5
0.6
0.7
0.8
0.9
1
1.1
0
10
20
30
40
50
60
70
80
90
100

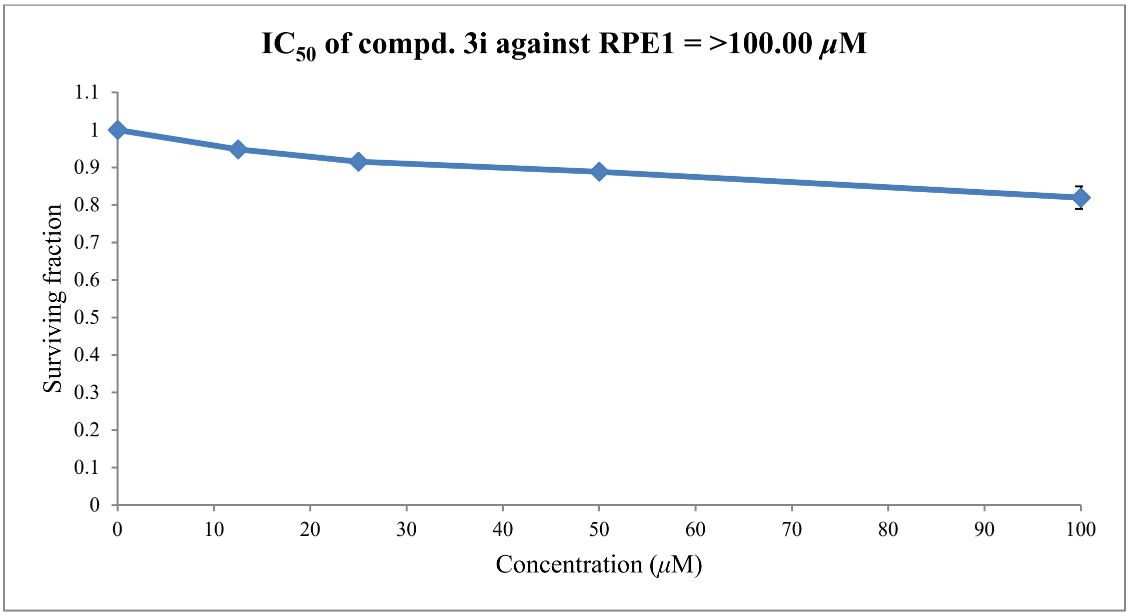
IC50 of compd. 3i against RPE1 = >100.00 μM
Surviving fraction
1.1
1
0.9
0.8
0.7
0.6
0.5
0.4
0.3
0.2
0.1
0
0
10
20
30
40
50
60
70
80
90
100
Concentration (μM)

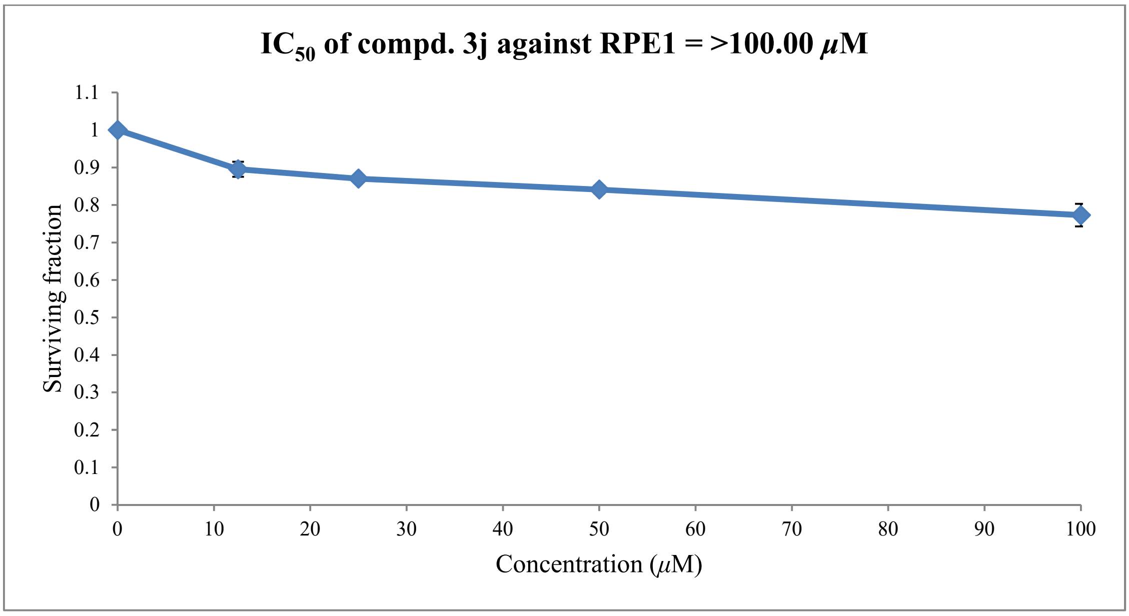
IC50 of compd. 3j against RPE1 = >100.00 μM
Surviving fraction
1.1
1
0.9
0.8
0.7
0.6
0.5
0.4
0.3
0.2
0.1
0
0
10
20
30
40
50
60
70
80
90
100
Concentration (μM)

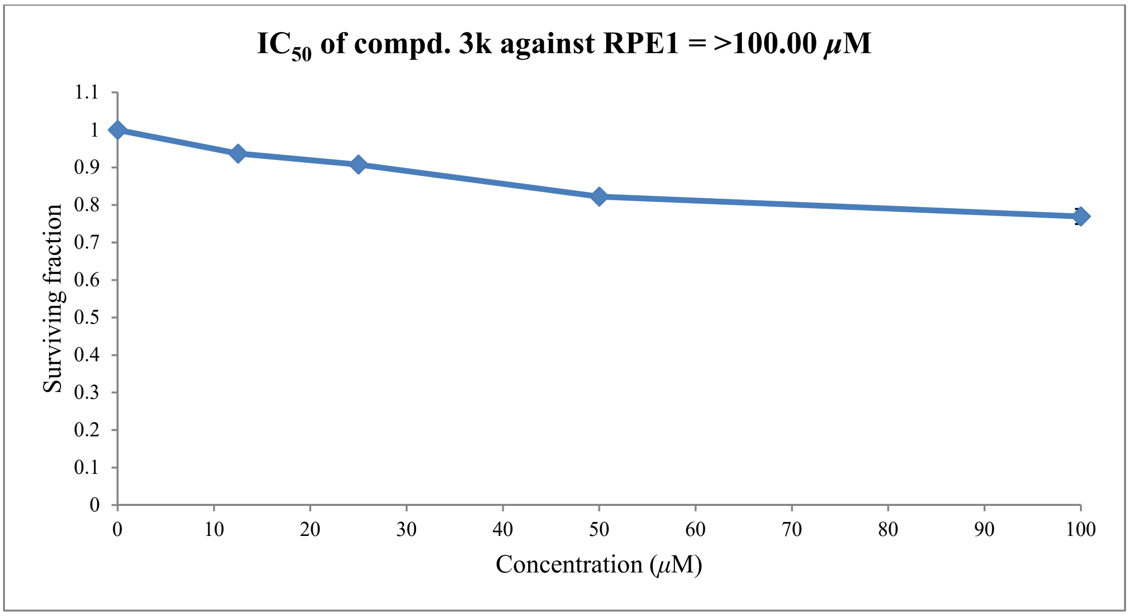

IC$_{50}$ of compd. 3k against RPE1 = >100.00 μM
Surviving fraction
Concentration (μM)

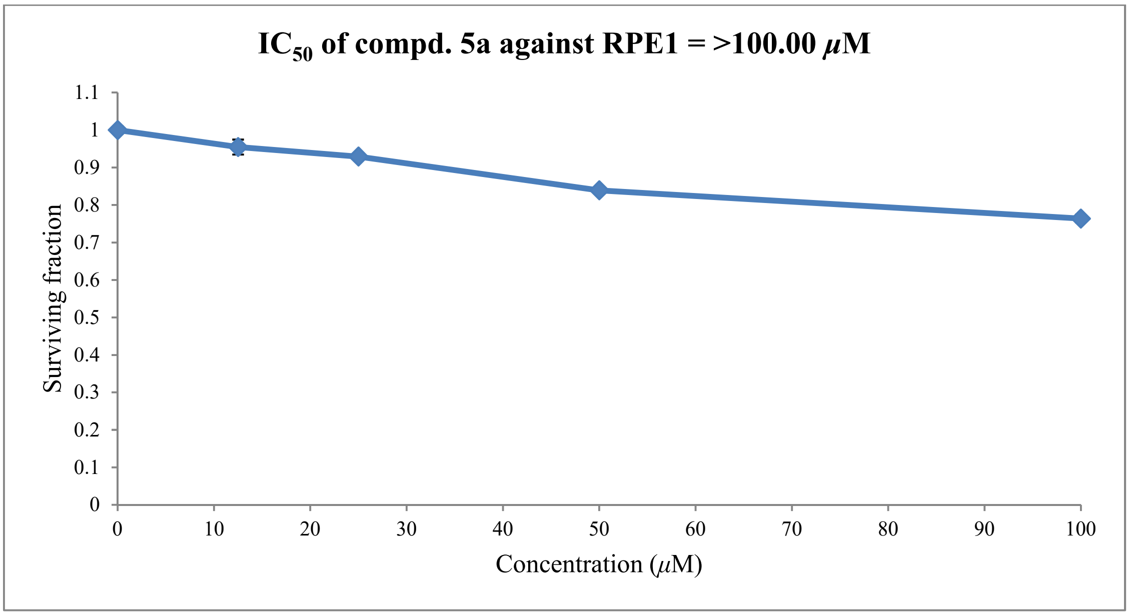

IC$_{50}$ of compd. 5a against RPE1 = >100.00 μM
Surviving fraction
Concentration (μM)

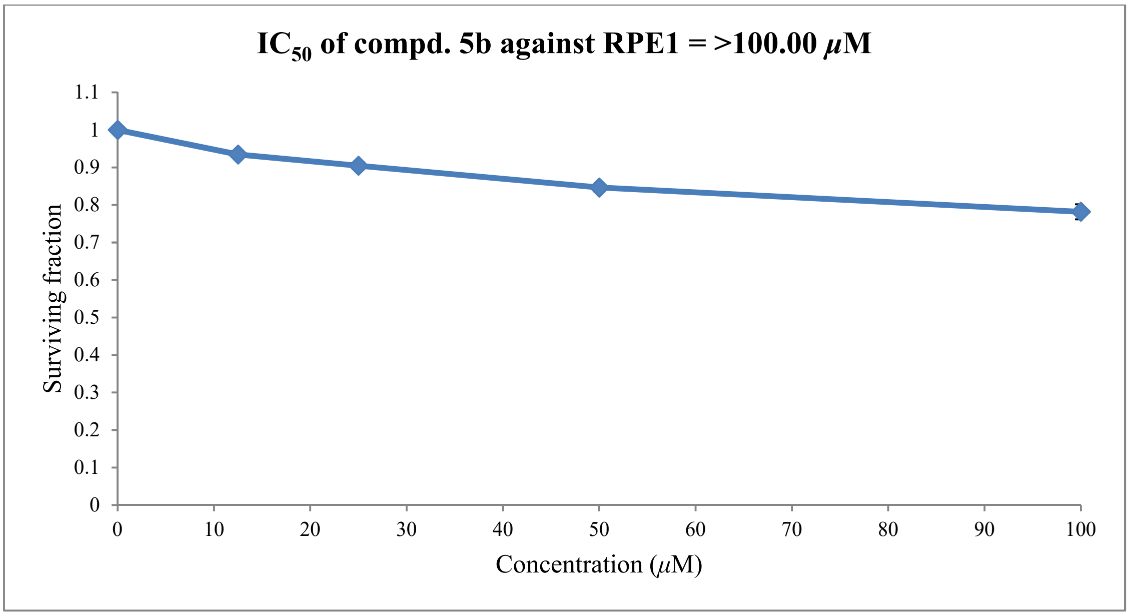
IC50 of compd. 5b against RPE1 = >100.00 μM
Surviving fraction
Concentration (μM)
0
0.1
0.2
0.3
0.4
0.5
0.6
0.7
0.8
0.9
1
1.1
0
10
20
30
40
50
60
70
80
90
100

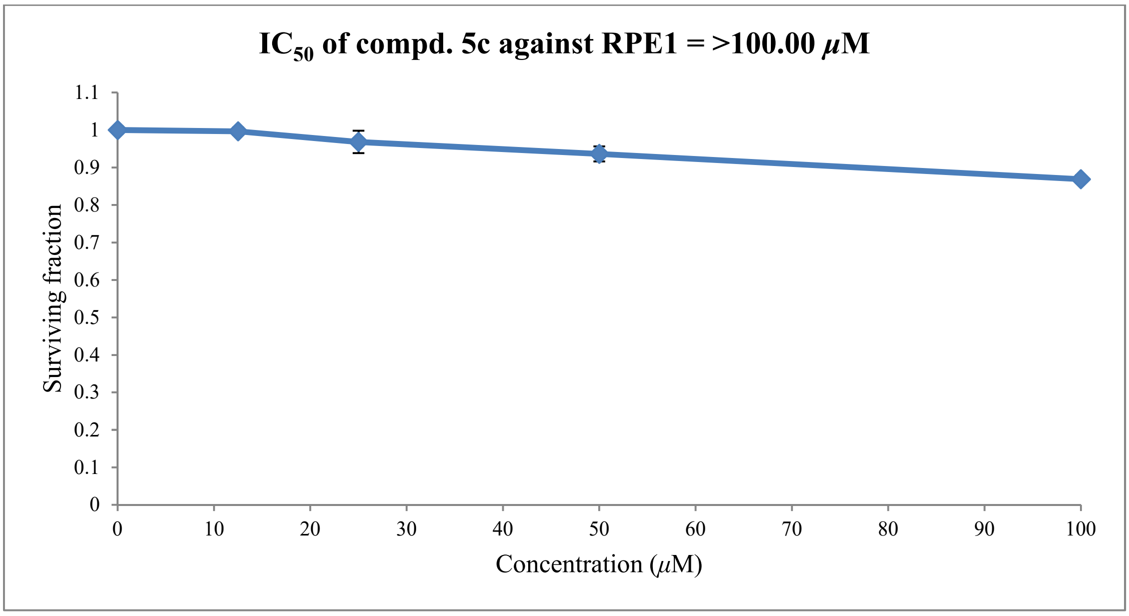
IC50 of compd. 5c against RPE1 = >100.00 μM
Surviving fraction
Concentration (μM)
0
0.1
0.2
0.3
0.4
0.5
0.6
0.7
0.8
0.9
1
1.1
0
10
20
30
40
50
60
70
80
90
100

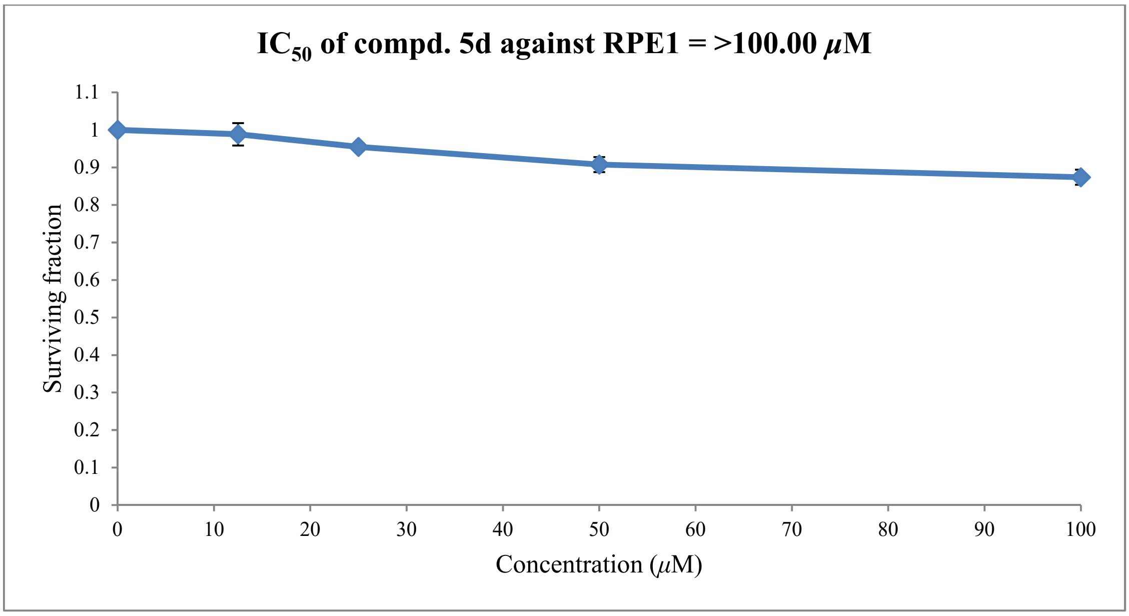
IC50 of compd. 5d against RPE1 = >100.00 μM
1.1
1
0.9
0.8
0.7
0.6
0.5
0.4
0.3
0.2
0.1
0
Surviving fraction
0
10
20
30
40
50
60
70
80
90
100
Concentration (μM)

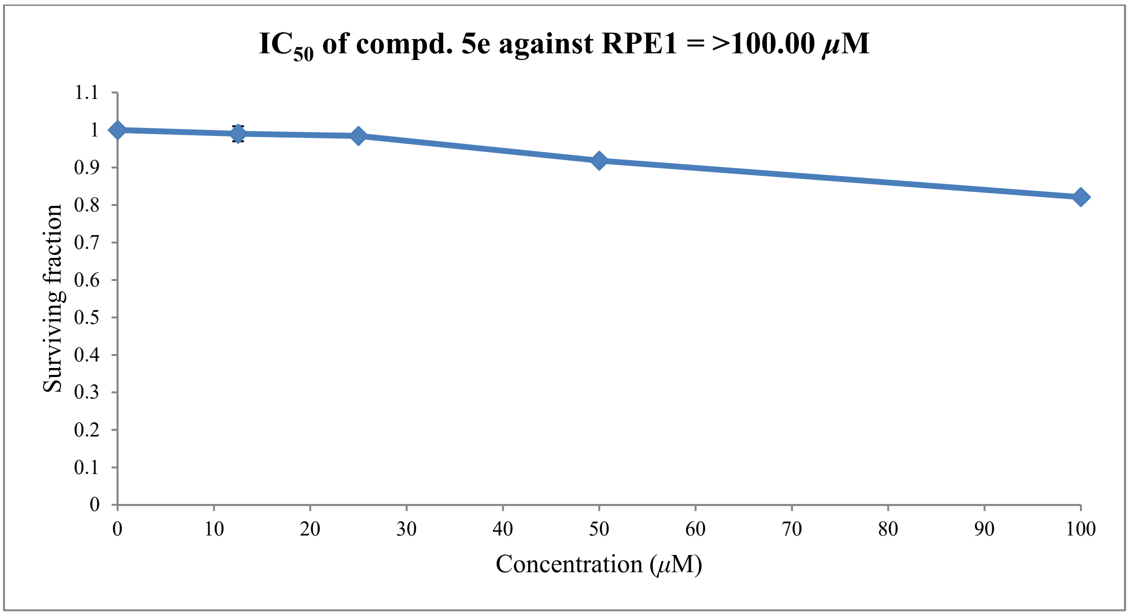
IC50 of compd. 5e against RPE1 = >100.00 μM
1.1
1
0.9
0.8
0.7
0.6
0.5
0.4
0.3
0.2
0.1
0
Surviving fraction
0
10
20
30
40
50
60
70
80
90
100
Concentration (μM)

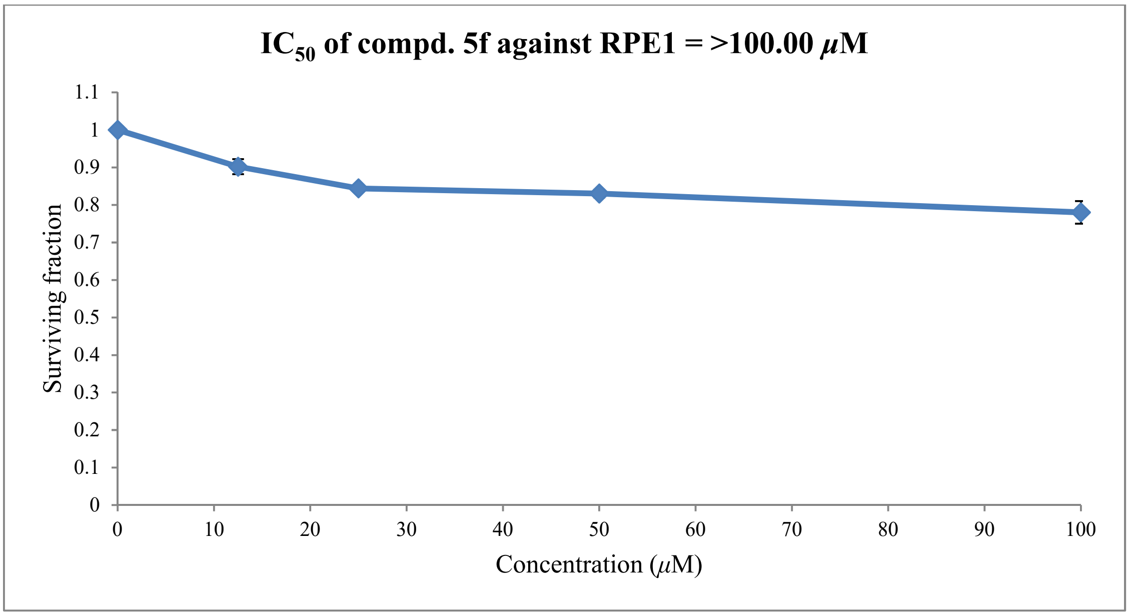

IC50 of compd. 5f against RPE1 = >100.00 μM
Surviving fraction
Concentration (μM)
1.1
1
0.9
0.8
0.7
0.6
0.5
0.4
0.3
0.2
0.1
0
0
10
20
30
40
50
60
70
80
90
100

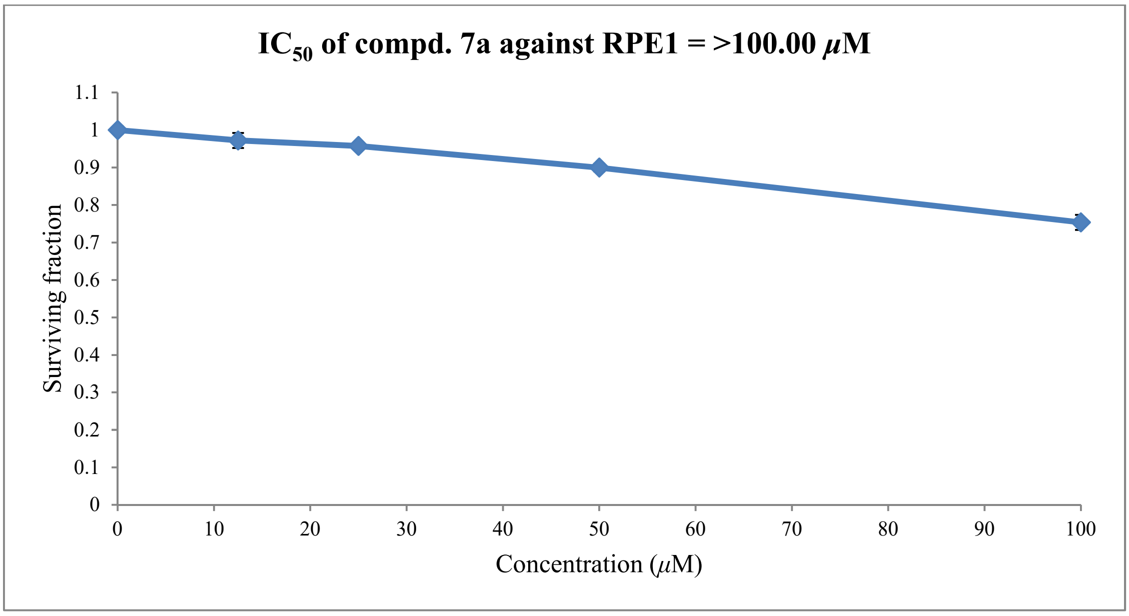

IC50 of compd. 7a against RPE1 = >100.00 μM
Surviving fraction
Concentration (μM)
1.1
1
0.9
0.8
0.7
0.6
0.5
0.4
0.3
0.2
0.1
0
0
10
20
30
40
50
60
70
80
90
100

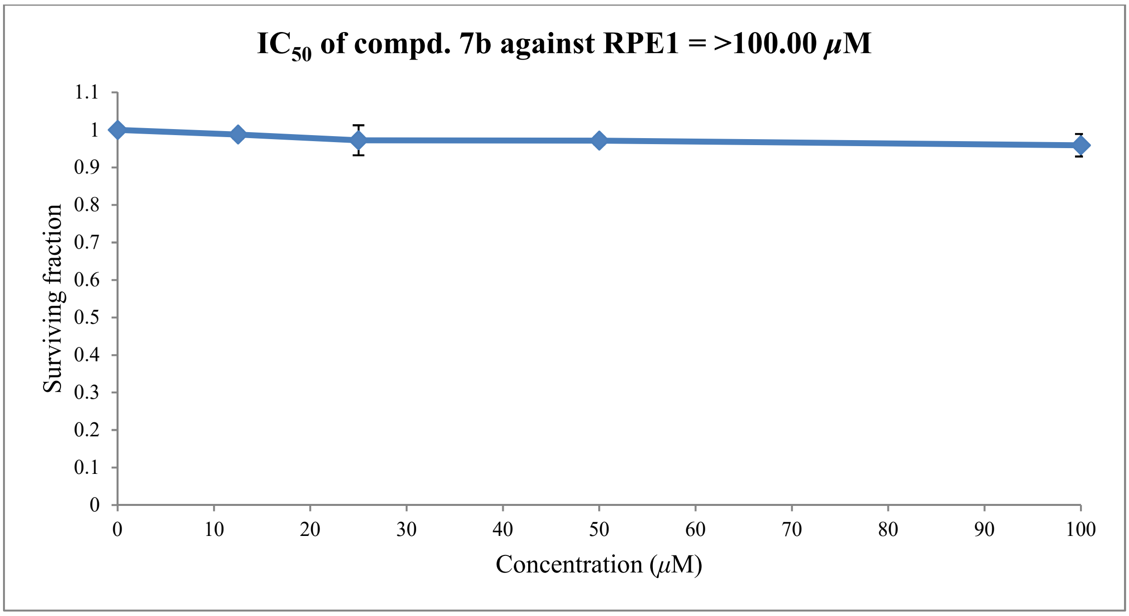
IC50 of compd. 7b against RPE1 = >100.00 μM
Surviving fraction
Concentration (μM)
1.1
1
0.9
0.8
0.7
0.6
0.5
0.4
0.3
0.2
0.1
0
10
20
30
40
50
60
70
80
90
100

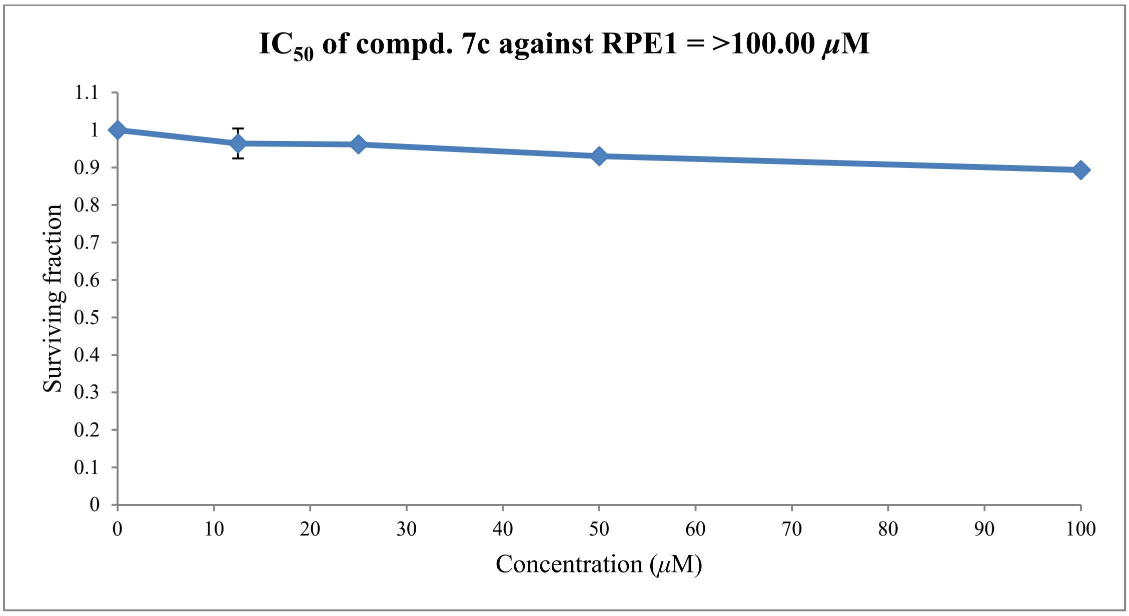
IC50 of compd. 7c against RPE1 = >100.00 μM
Surviving fraction
Concentration (μM)
1.1
1
0.9
0.8
0.7
0.6
0.5
0.4
0.3
0.2
0.1
0
10
20
30
40
50
60
70
80
90
100

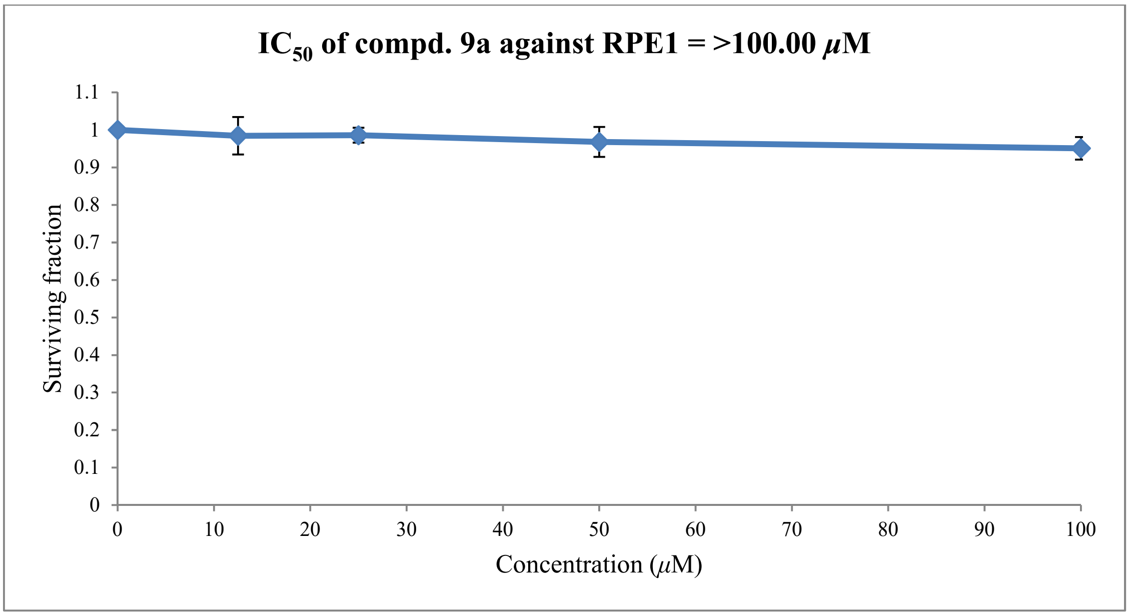
IC$_{50}$ of compd. 9a against RPE1 = >100.00 μM
Surviving fraction
1.1
1
0.9
0.8
0.7
0.6
0.5
0.4
0.3
0.2
0.1
0
0
10
20
30
40
50
60
70
80
90
100
Concentration (μM)

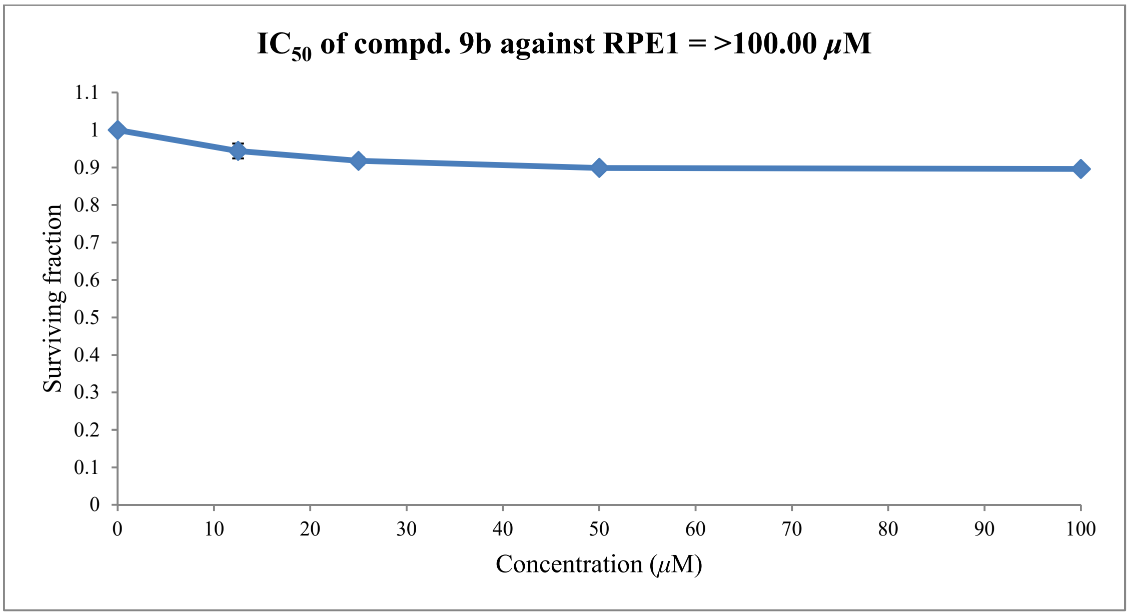
IC$_{50}$ of compd. 9b against RPE1 = >100.00 μM
Surviving fraction
1.1
1
0.9
0.8
0.7
0.6
0.5
0.4
0.3
0.2
0.1
0
0
10
20
30
40
50
60
70
80
90
100
Concentration (μM)

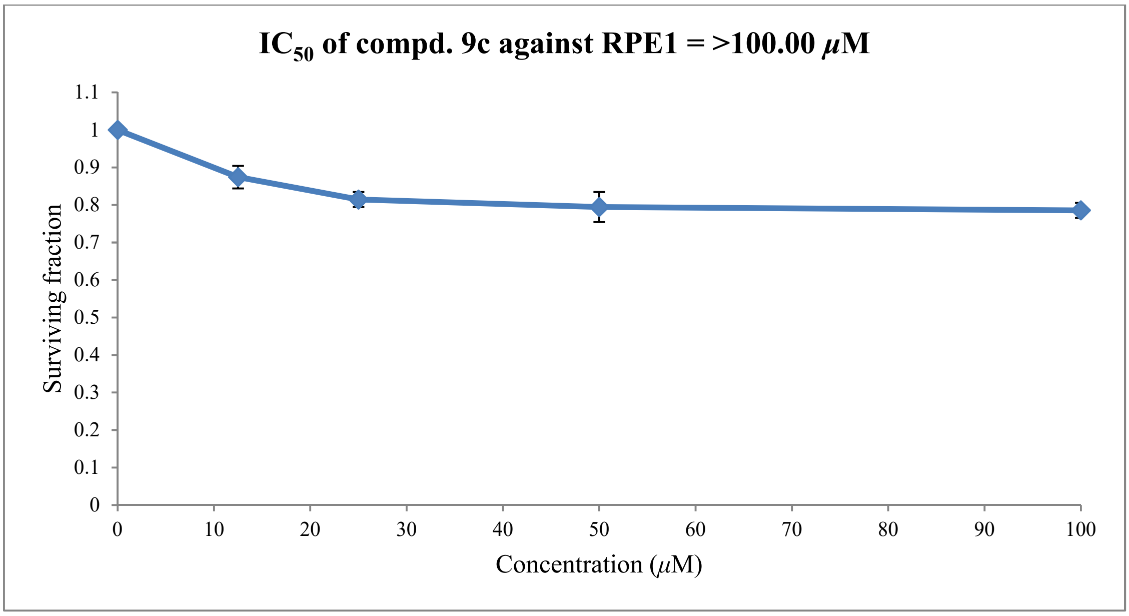
IC50 of compd. 9c against RPE1 = >100.00 μM
Surviving fraction
Concentration (μM)
1.1
1
0.9
0.8
0.7
0.6
0.5
0.4
0.3
0.2
0.1
0
0
10
20
30
40
50
60
70
80
90
100

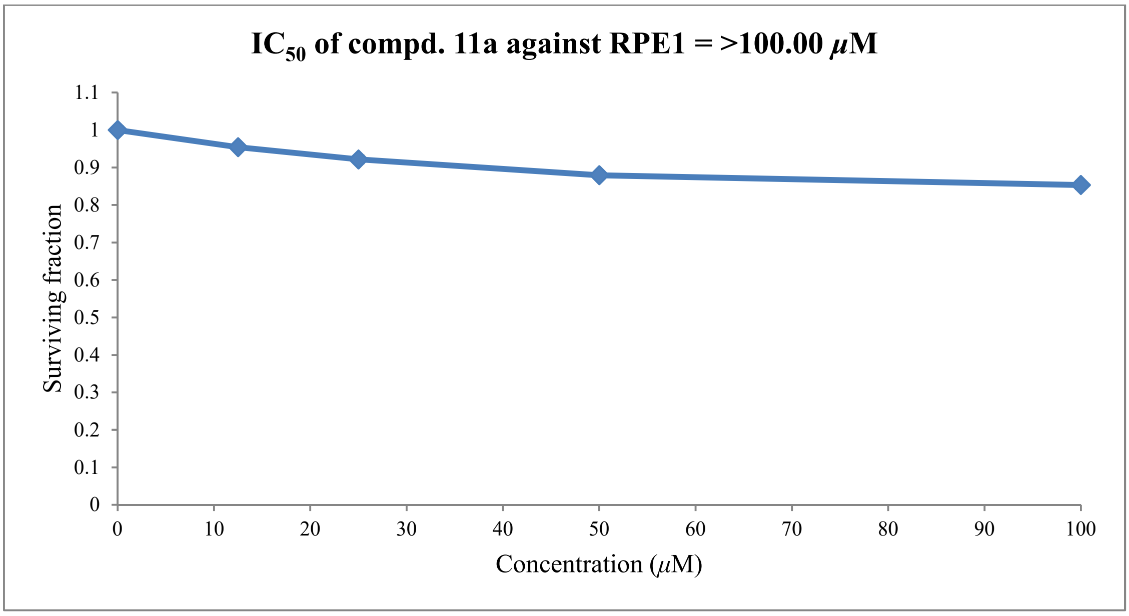
IC50 of compd. 11a against RPE1 = >100.00 μM
Surviving fraction
Concentration (μM)
1.1
1
0.9
0.8
0.7
0.6
0.5
0.4
0.3
0.2
0.1
0
0
10
20
30
40
50
60
70
80
90
100

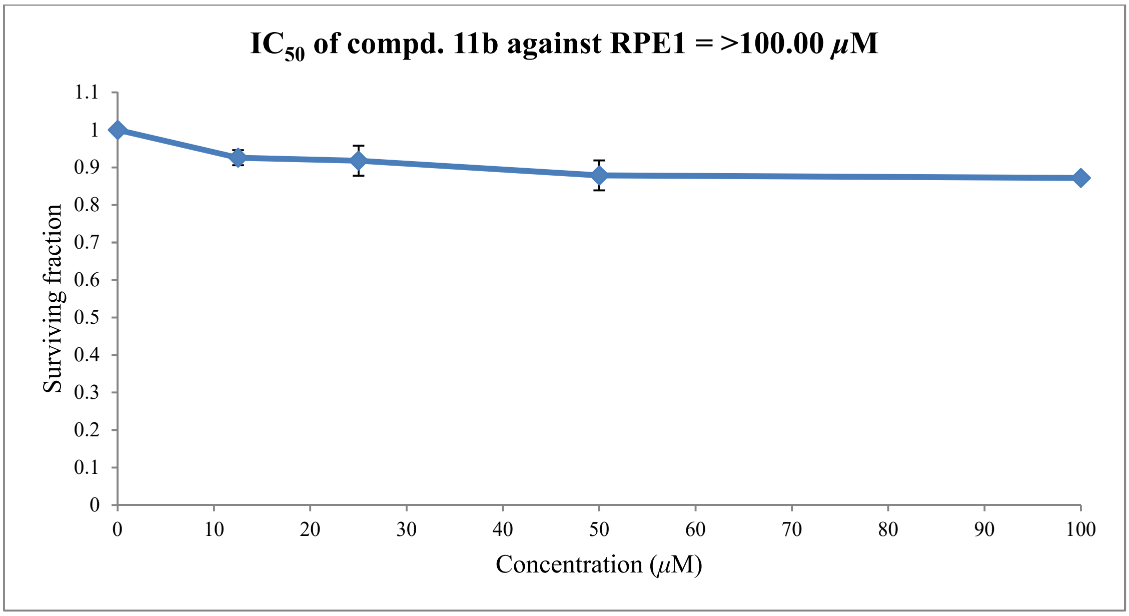
IC$_{50}$ of compd. 11b against RPE1 = >100.00 μM
Surviving fraction
Concentration (μM)
1.1
1
0.9
0.8
0.7
0.6
0.5
0.4
0.3
0.2
0.1
0
0
10
20
30
40
50
60
70
80
90
100

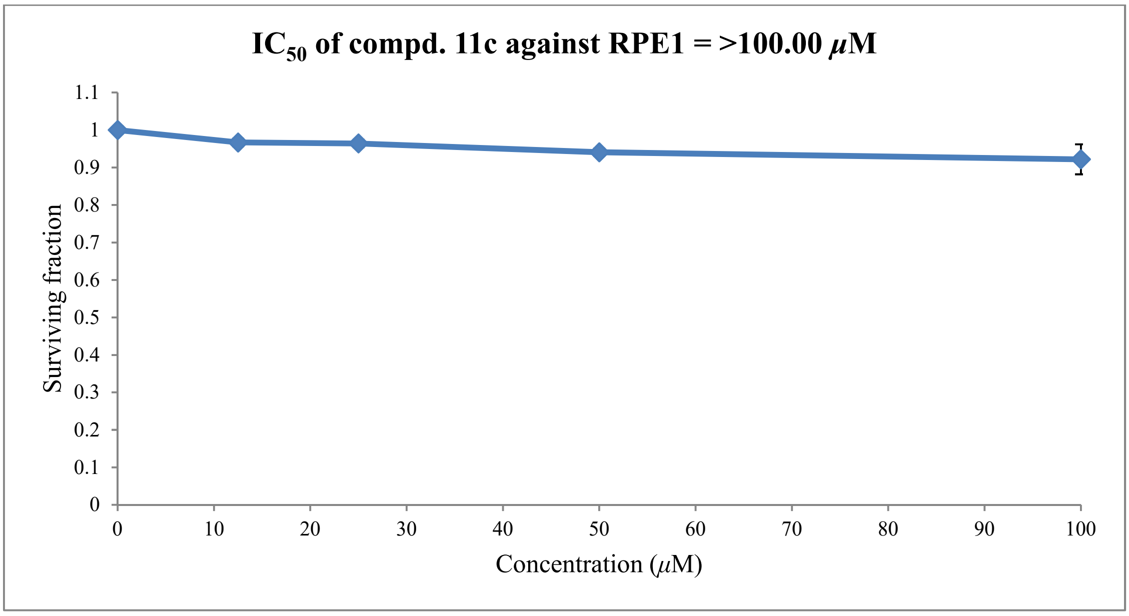
IC$_{50}$ of compd. 11c against RPE1 = >100.00 μM
Surviving fraction
Concentration (μM)
1.1
1
0.9
0.8
0.7
0.6
0.5
0.4
0.3
0.2
0.1
0
0
10
20
30
40
50
60
70
80
90
100

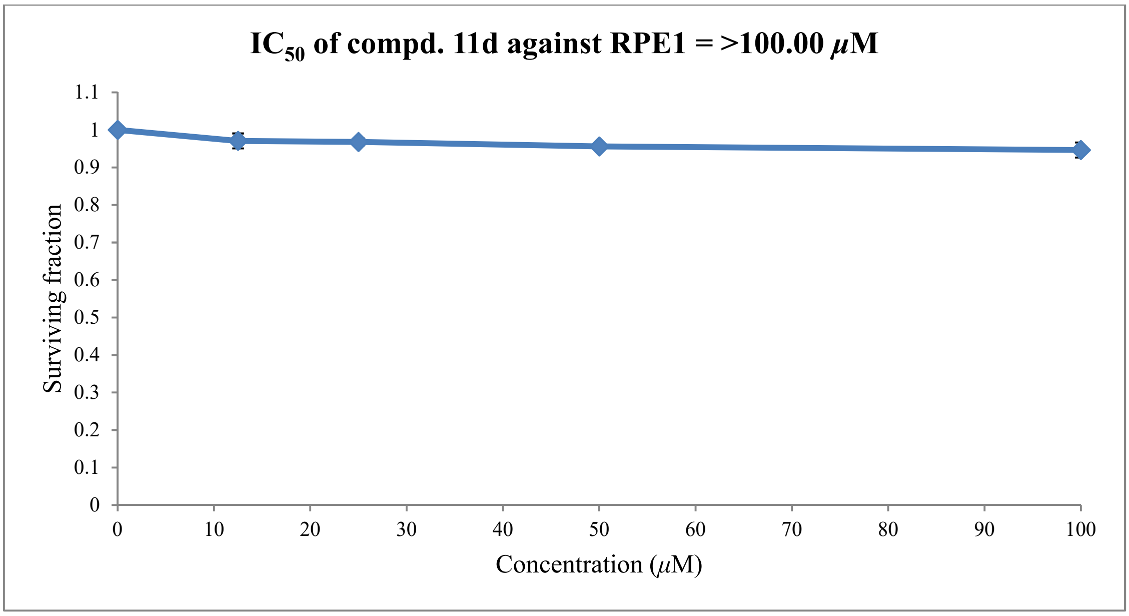

IC50 of compd. 11d against RPE1 = >100.00 μM
1.1
1
0.9
0.8
0.7
0.6
0.5
0.4
0.3
0.2
0.1
0
0
10
20
30
40
50
60
70
80
90
100
Surviving fraction
Concentration (μM)

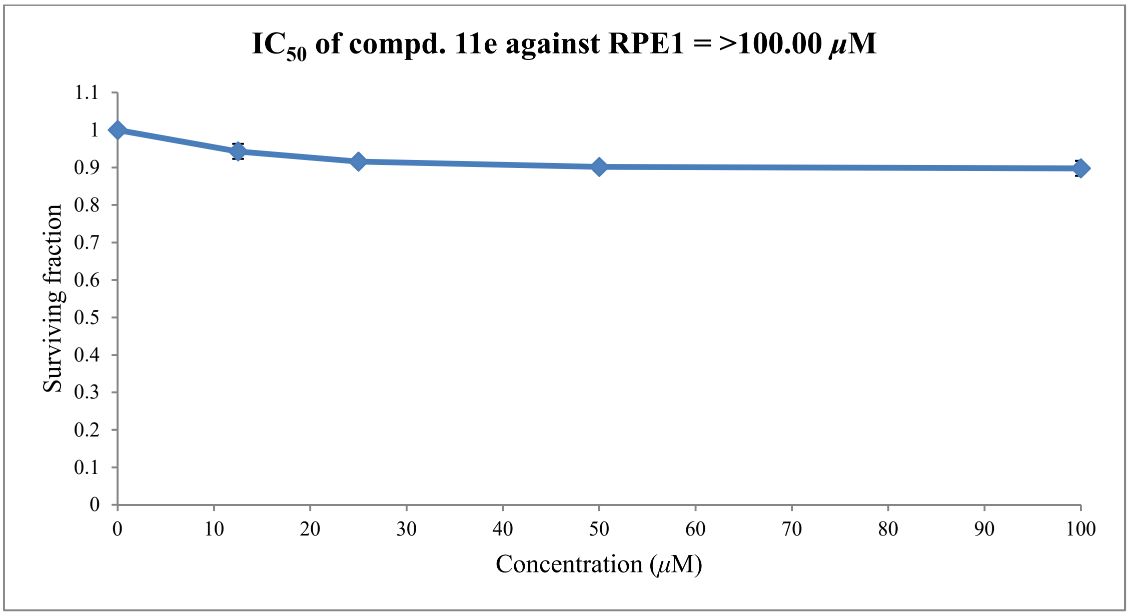

IC50 of compd. 11e against RPE1 = >100.00 μM
1.1
1
0.9
0.8
0.7
0.6
0.5
0.4
0.3
0.2
0.1
0
0
10
20
30
40
50
60
70
80
90
100
Surviving fraction
Concentration (μM)

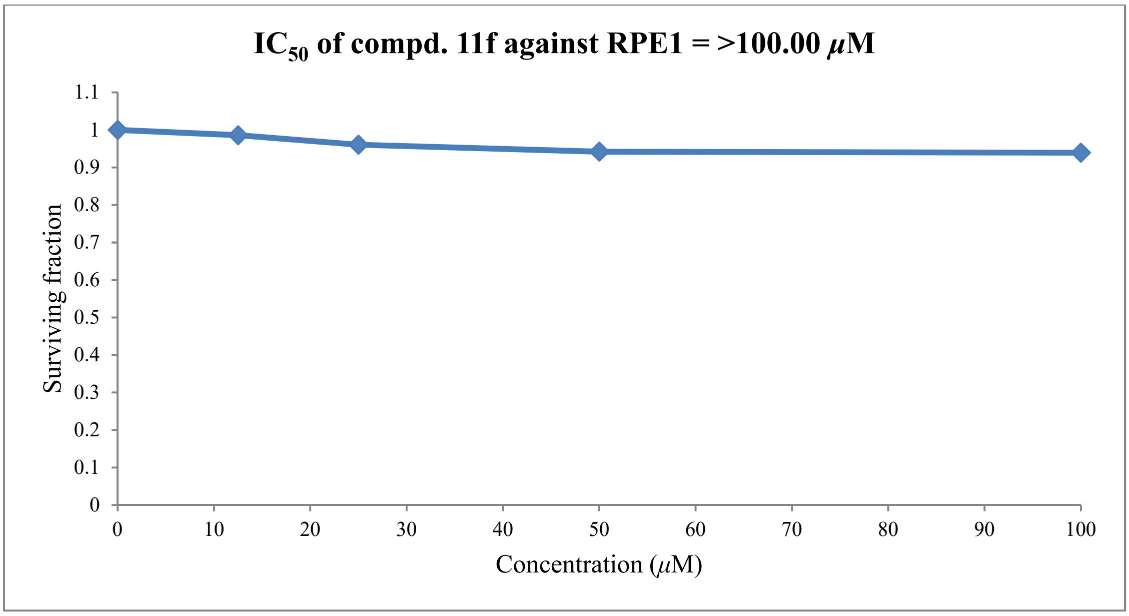
IC50 of compd. 11f against RPE1 = >100.00 μM
Surviving fraction
Concentration (μM)
0
0.1
0.2
0.3
0.4
0.5
0.6
0.7
0.8
0.9
1
1.1
0
10
20
30
40
50
60
70
80
90
100

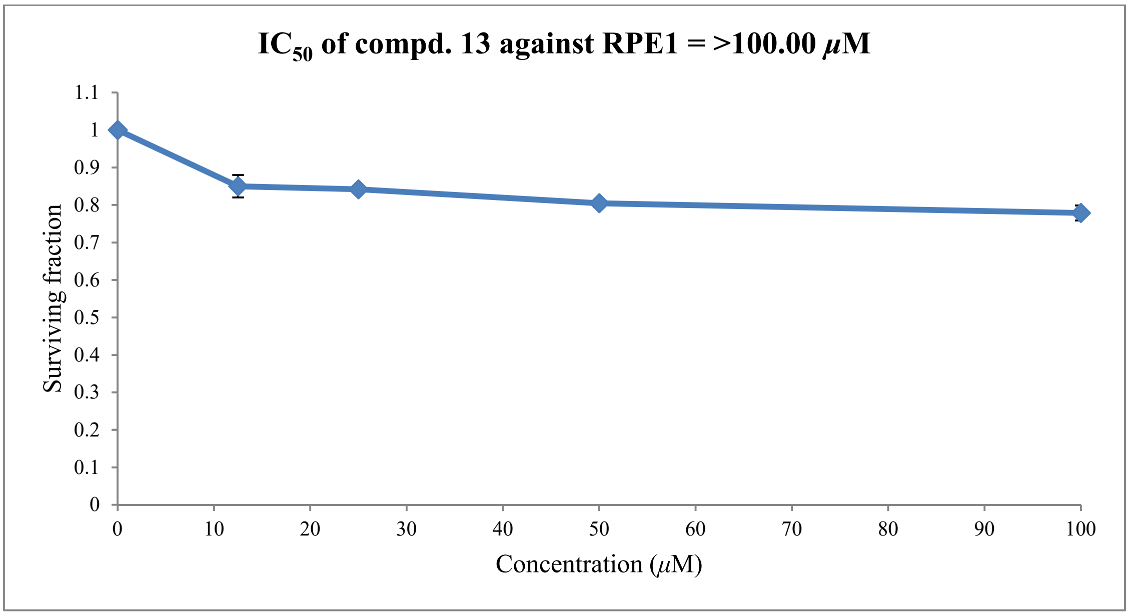
IC50 of compd. 13 against RPE1 = >100.00 μM
Surviving fraction
Concentration (μM)
0
0.1
0.2
0.3
0.4
0.5
0.6
0.7
0.8
0.9
1
1.1
0
10
20
30
40
50
60
70
80
90
100

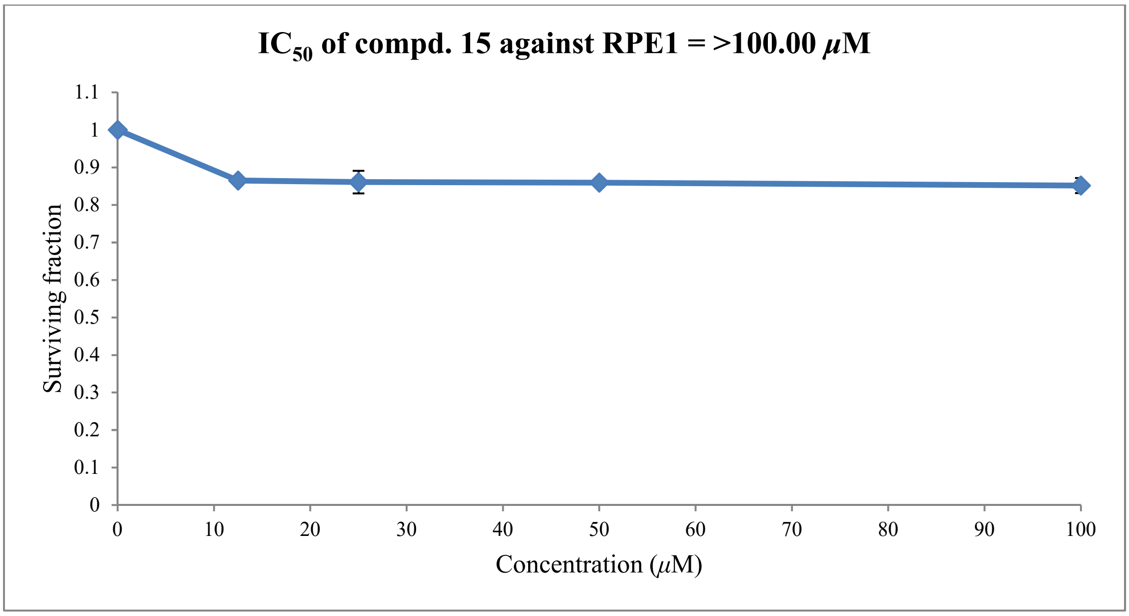

Fig. S1. Dose-response curve for the tested compounds against RPE1 (retinal pigment epithelium) cell line.

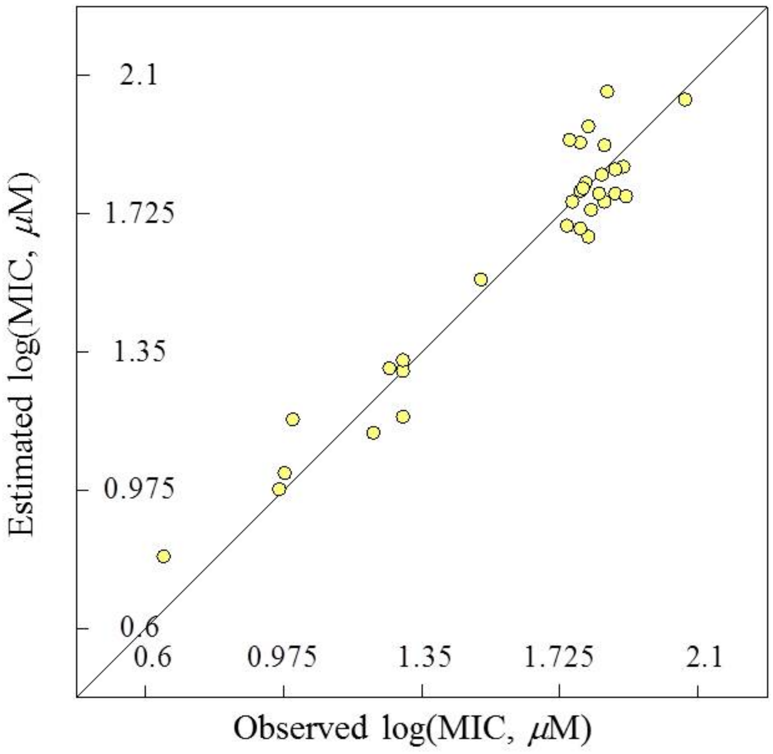

Fig. S2. QSAR plot representing the observed versus predicted log(MIC, μM) for the tested compounds against *S. aureus*.

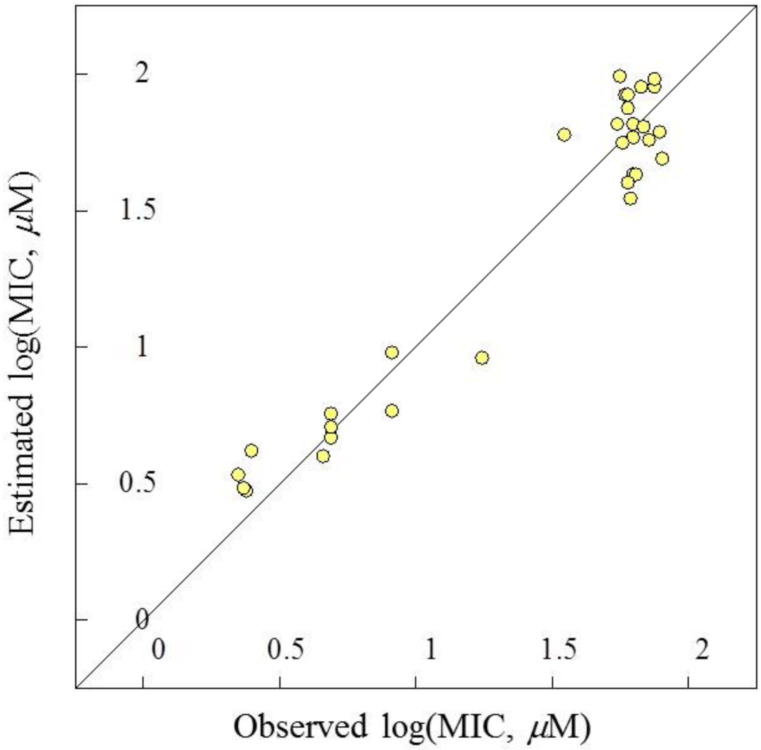

Fig. S3. QSAR plot representing the observed versus predicted log(MIC, μM) for the tested compounds against *B. subtilis*.

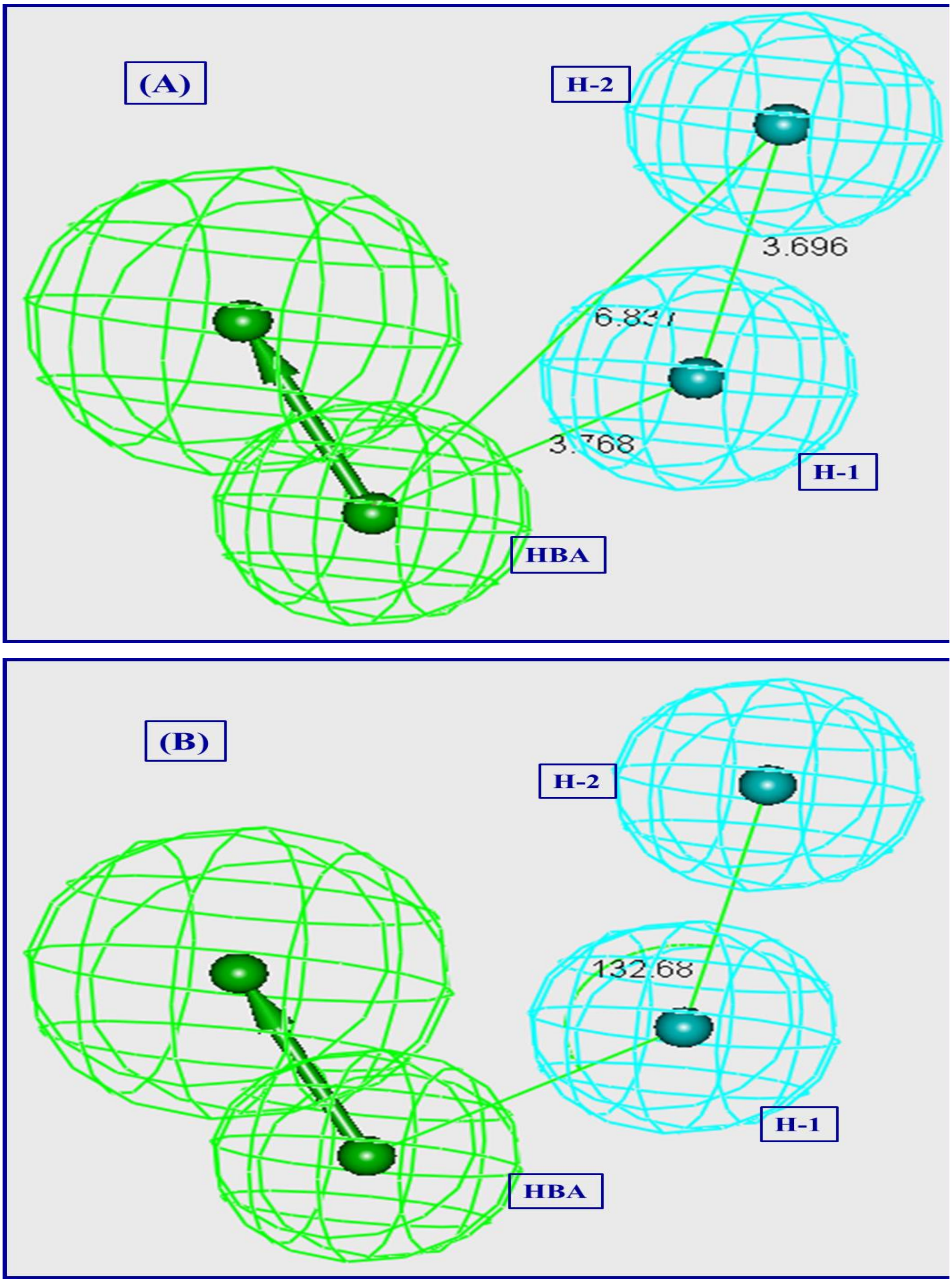

Fig. S4. (A) Constraint distances "H-1 – H-2 = 3.696, H-1 – HBA = 3.768, H-2 – HBA = 6.837 Å", **(B)** Constraint angle "H-2 – H-1 – HBA = 132.68 °" of the generated 3D-pharmacophore for the tested compounds against *S. aureus*.

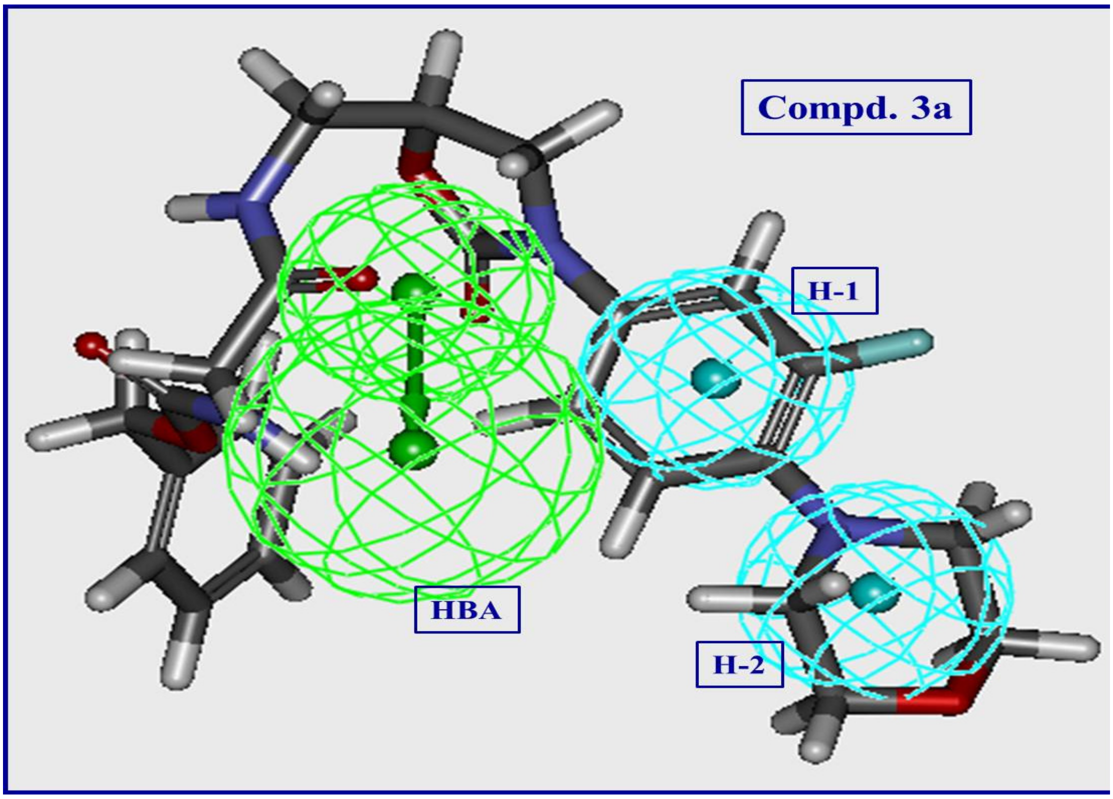

Compd. 3a
H-1
HBA
H-2

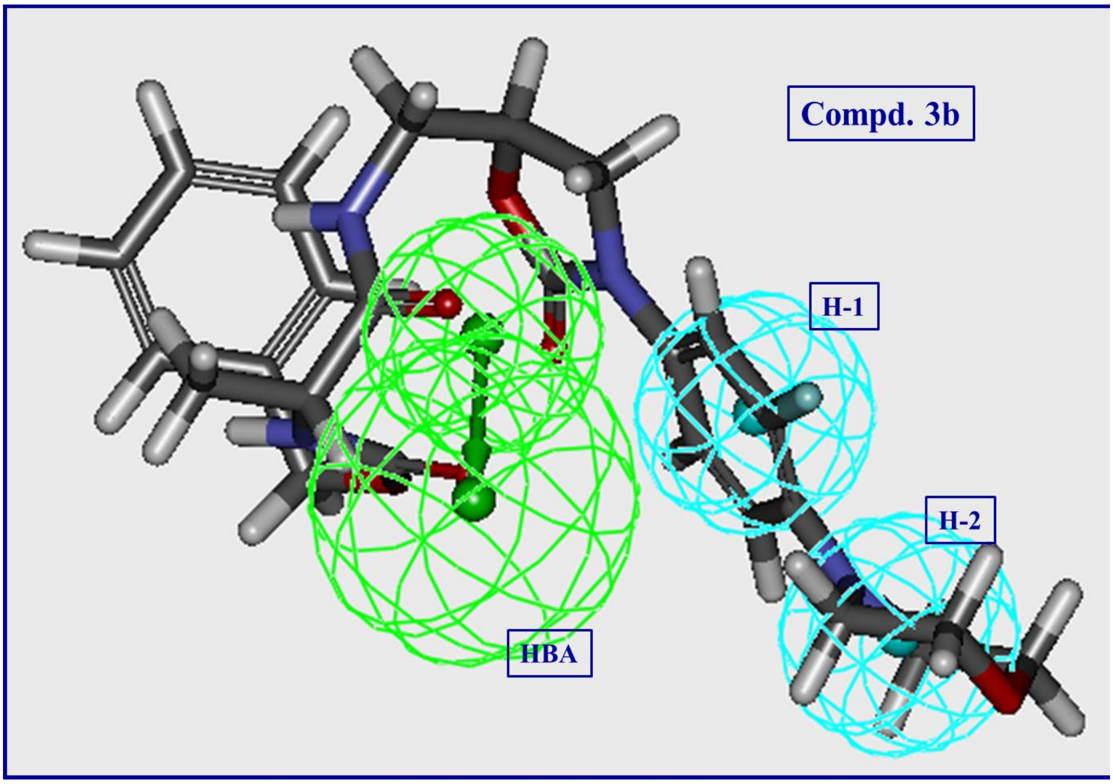

Compd. 3b
H-1
H-2
HBA

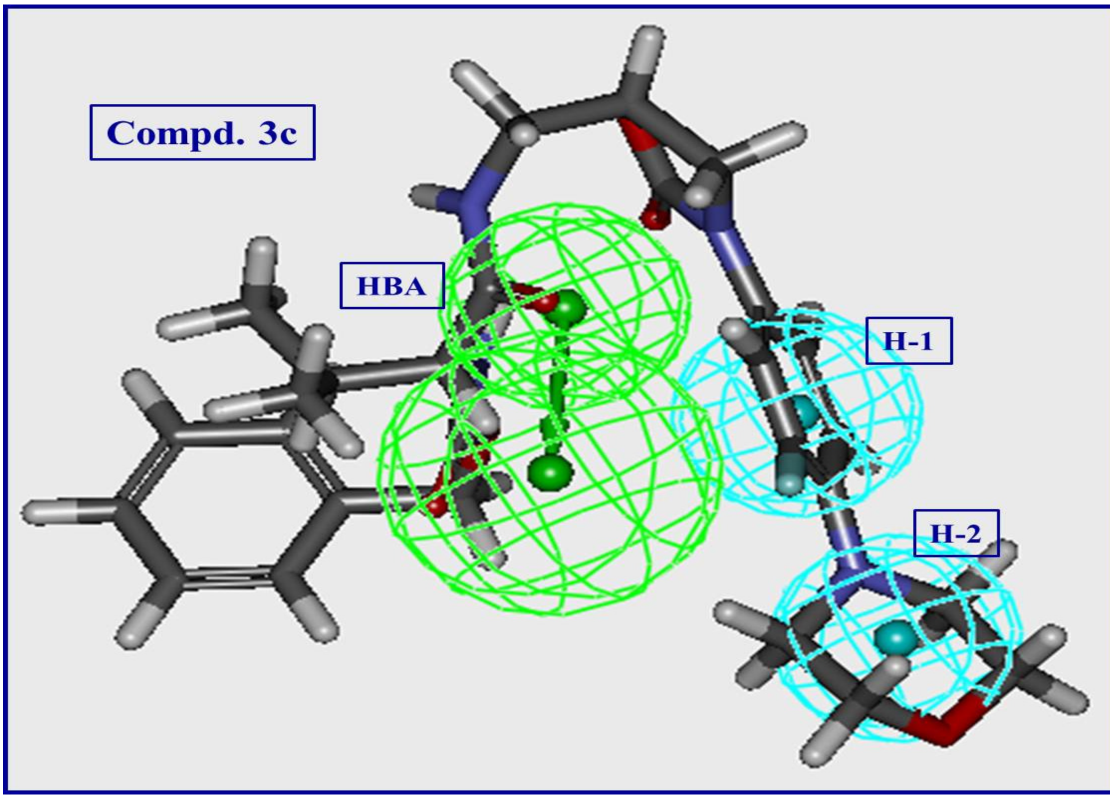
Compd. 3c
HBA
H-1
H-2

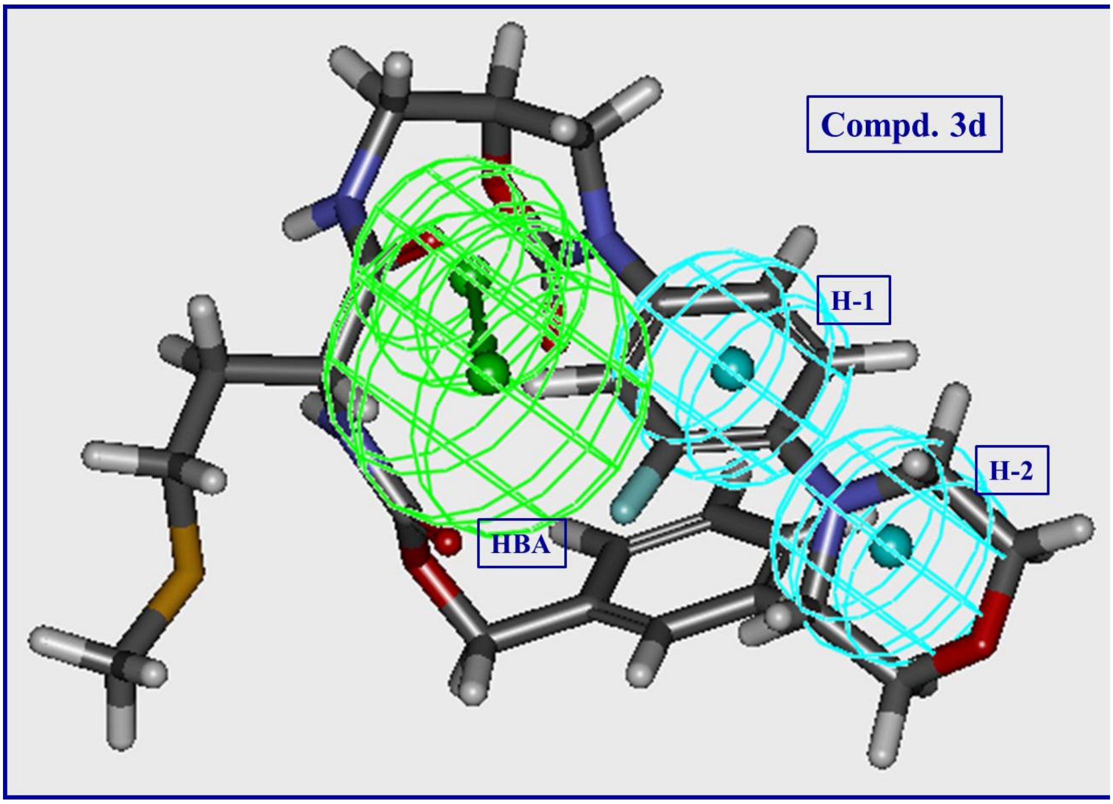
Compd. 3d
H-1
H-2
HBA

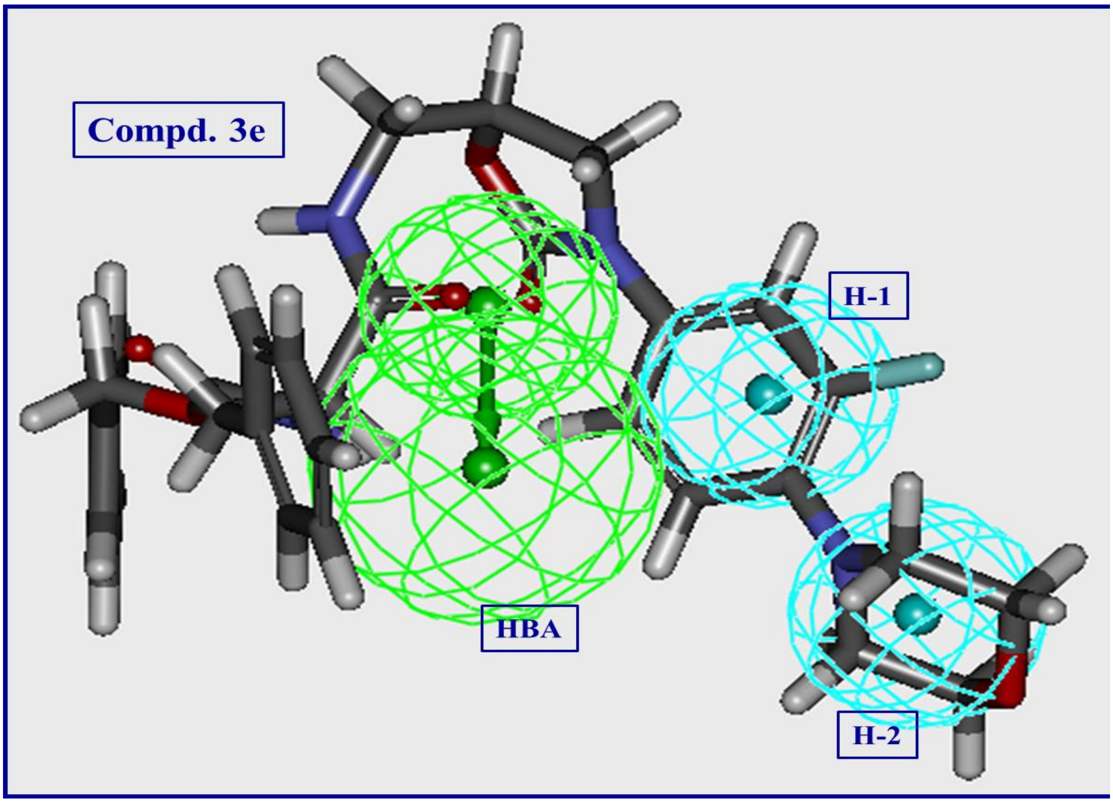
Compd. 3e
H-1
HBA
H-2

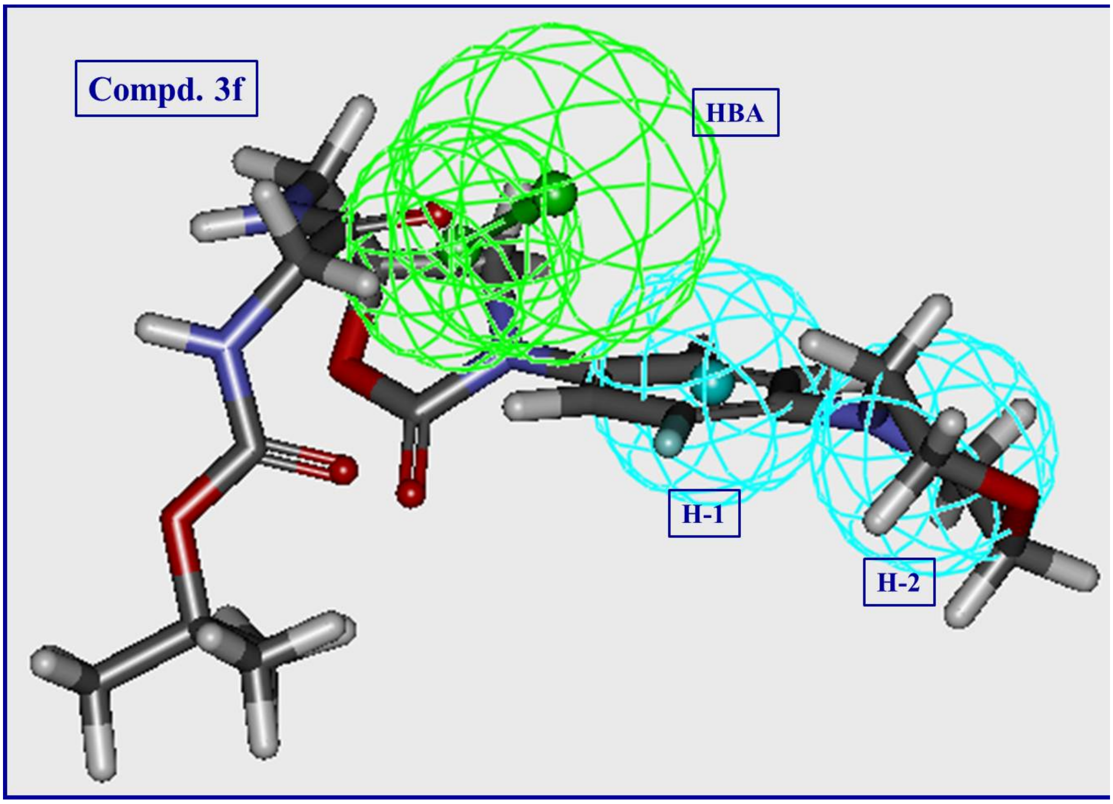
Compd. 3f
HBA
H-1
H-2

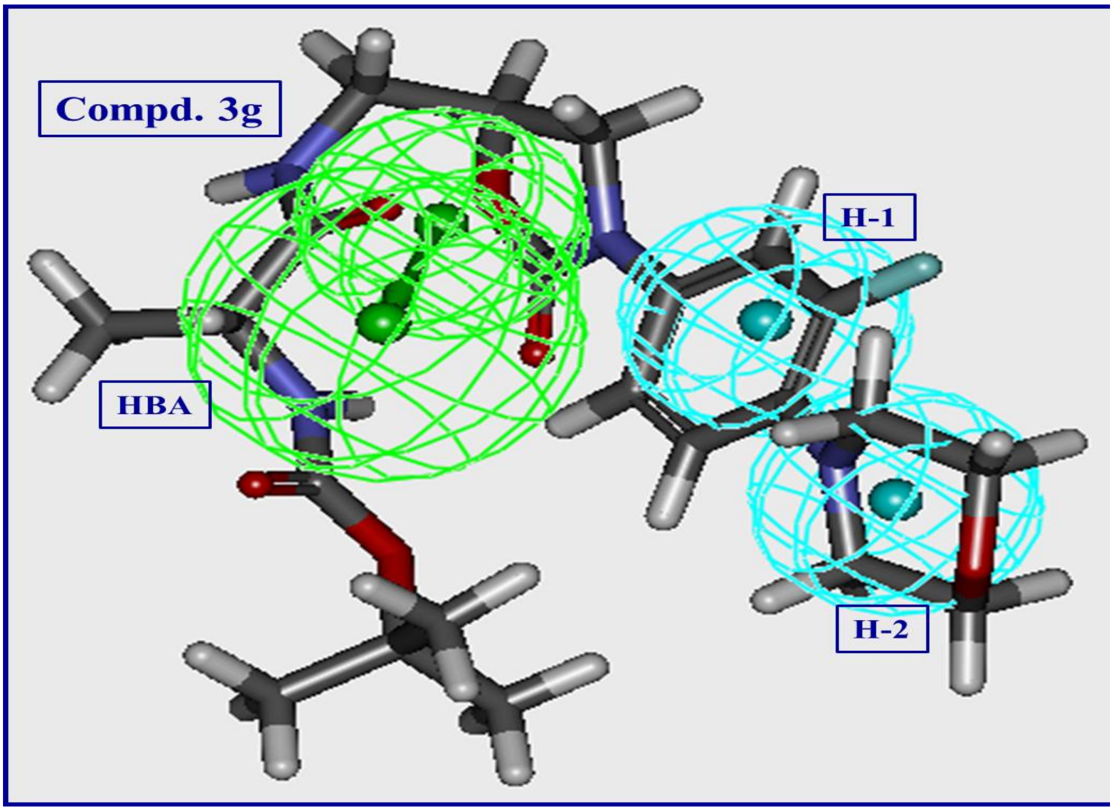
Compd. 3g
H-1
HBA
H-2

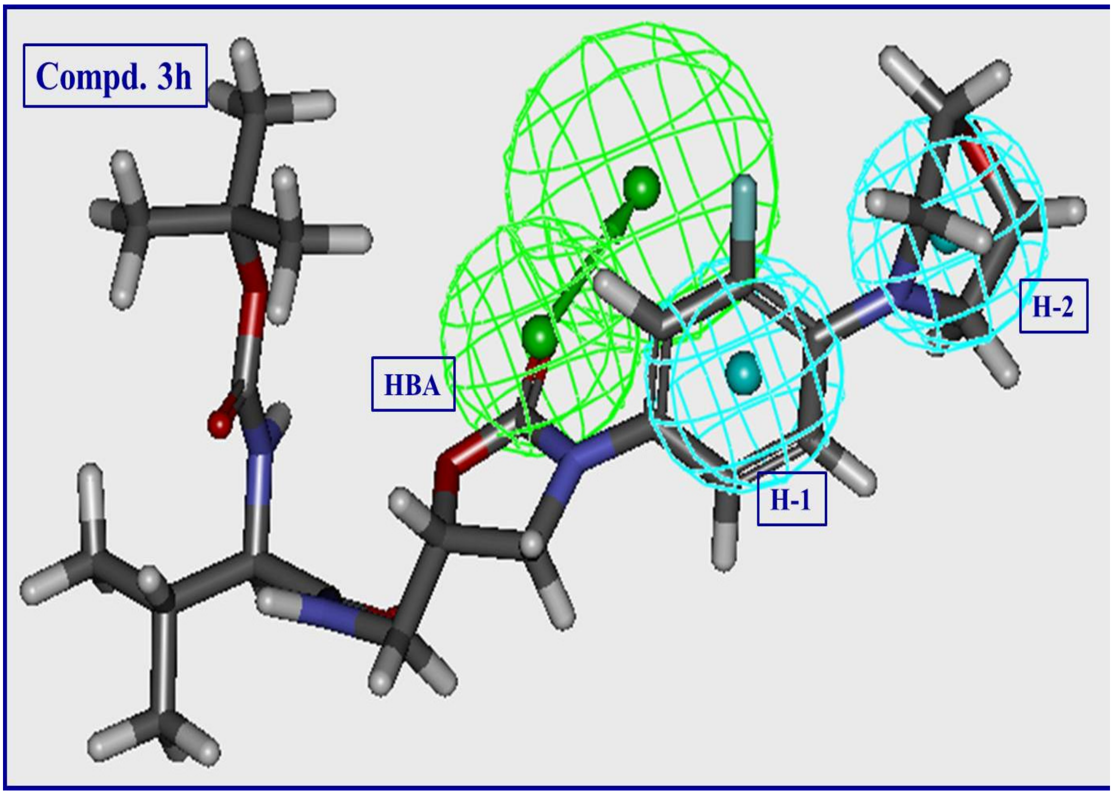
Compd. 3h
HBA
H-2
H-1

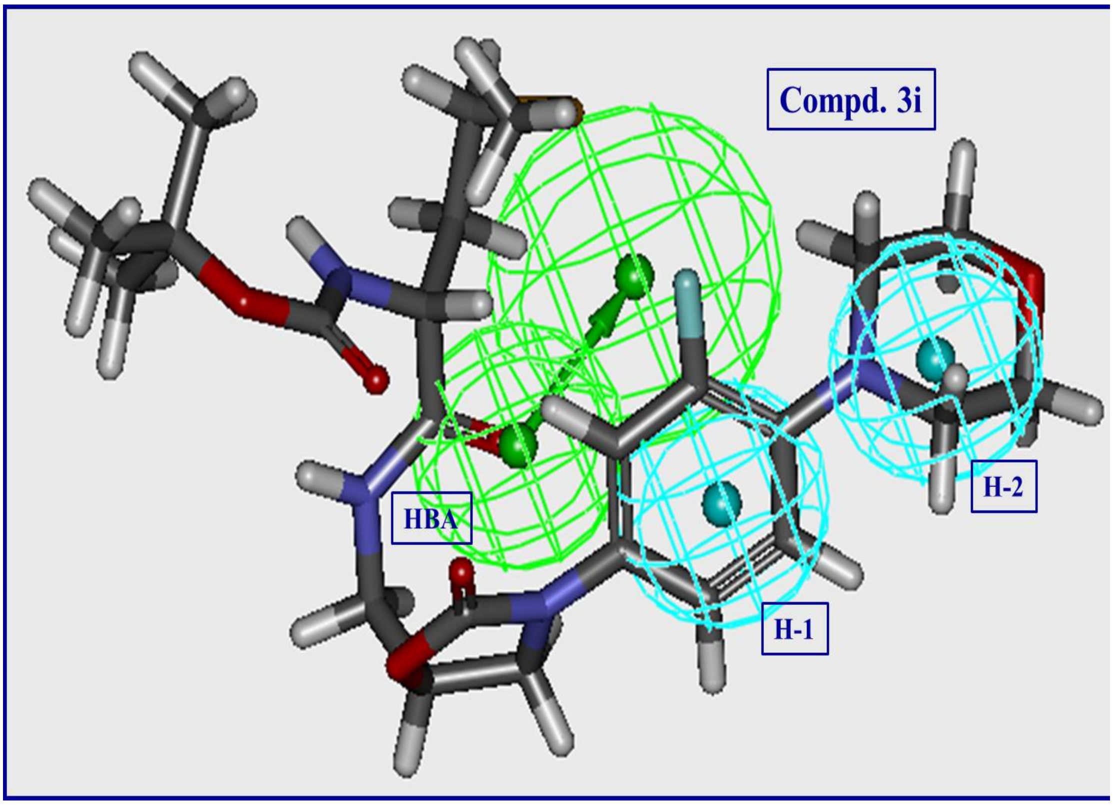
Compd. 3i
HBA
H-1
H-2

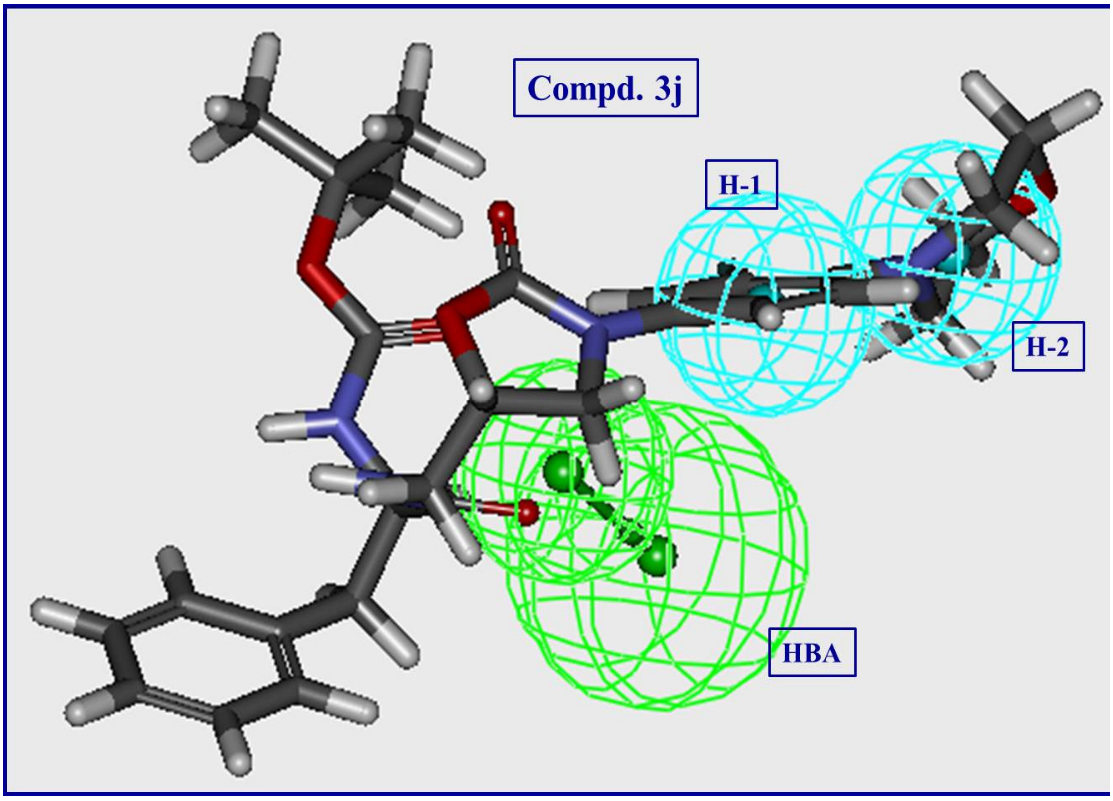
Compd. 3j
H-1
H-2
HBA

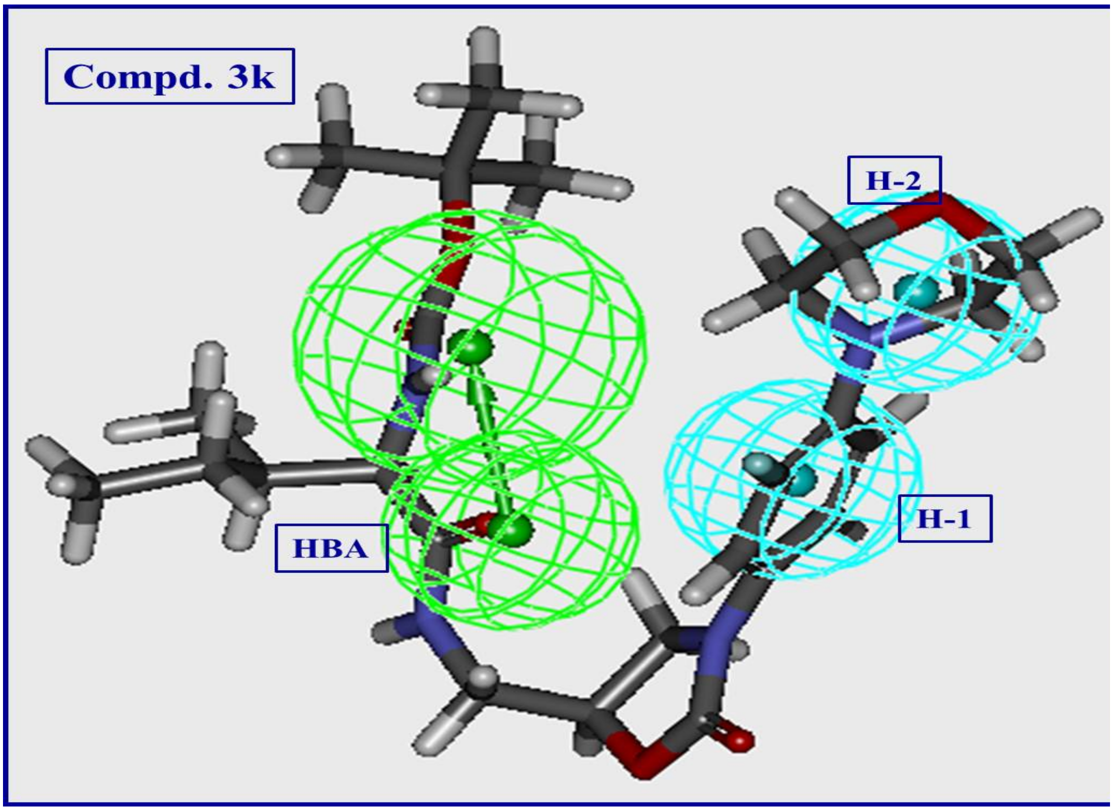
Compd. 3k
H-2
H-1
HBA

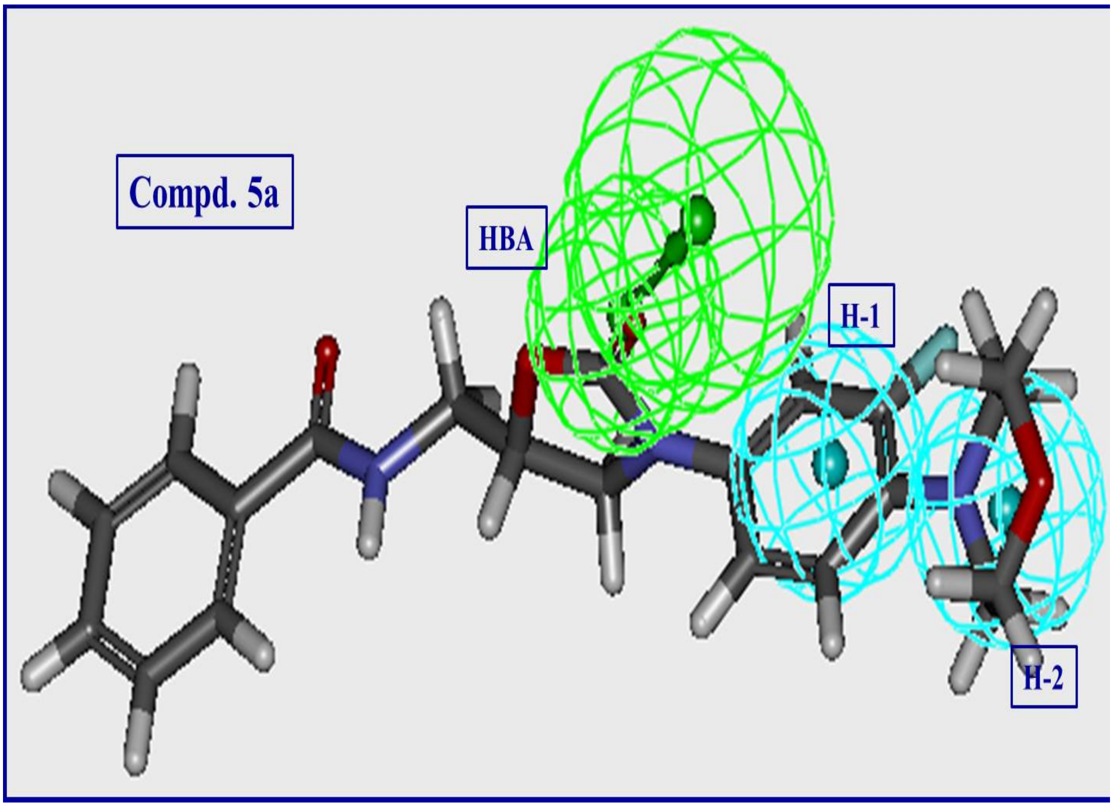
Compd. 5a
HBA
H-1
H-2

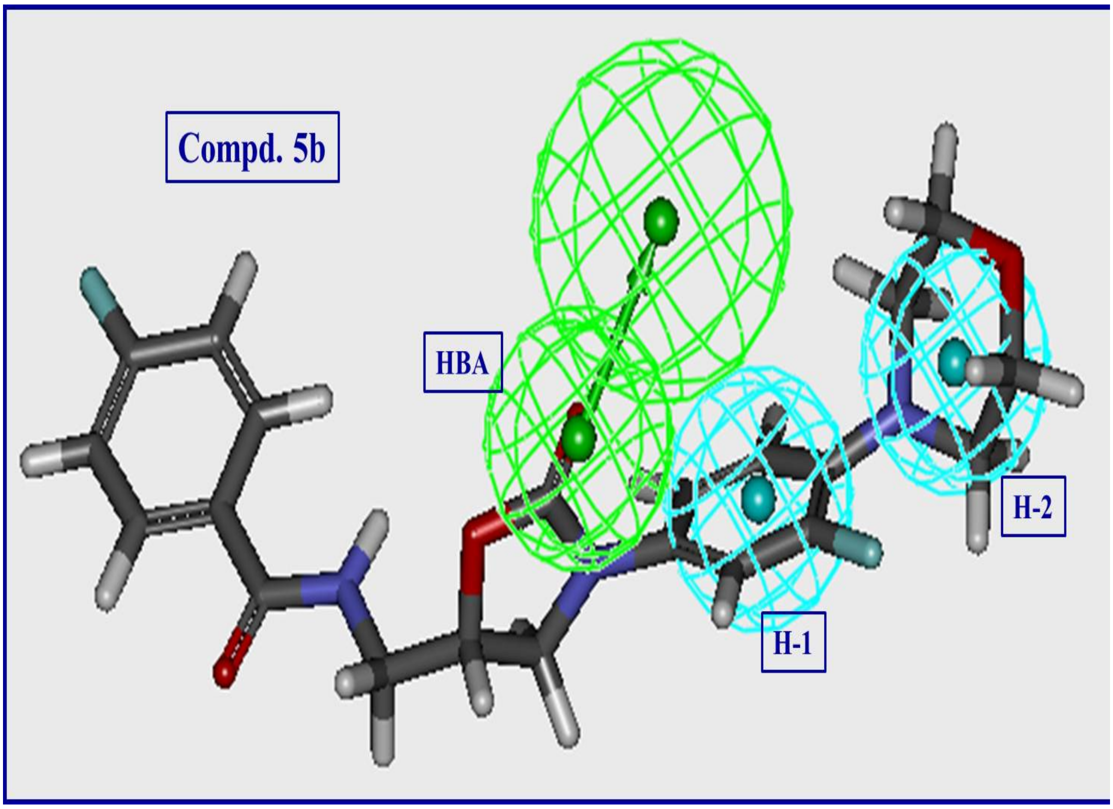
Compd. 5b
HBA
H-2
H-1

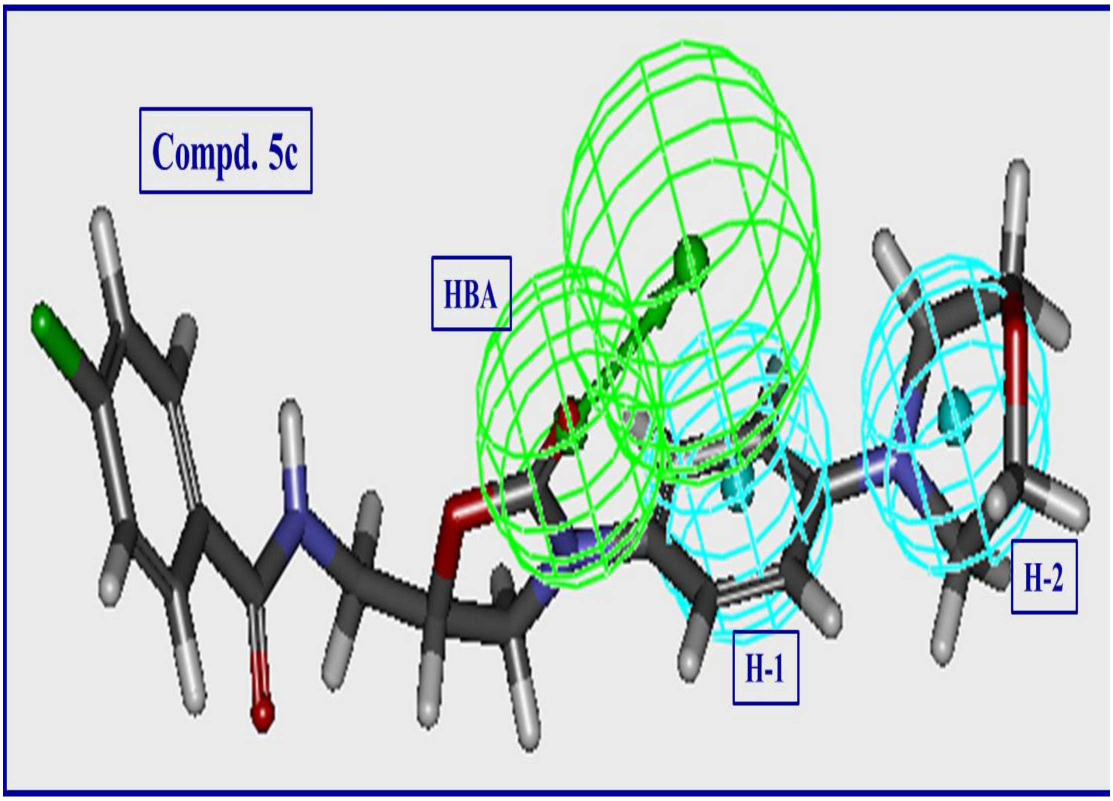
Compd. 5c
HBA
H-2
H-1

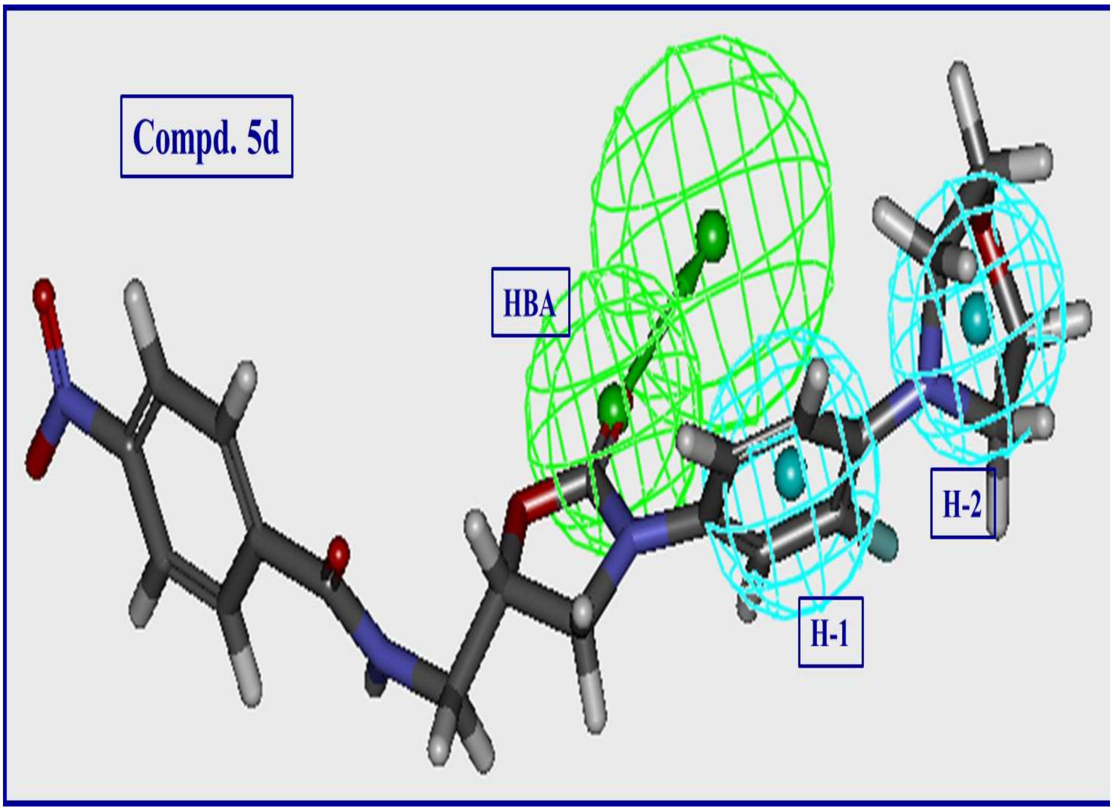
Compd. 5d
HBA
H-2
H-1

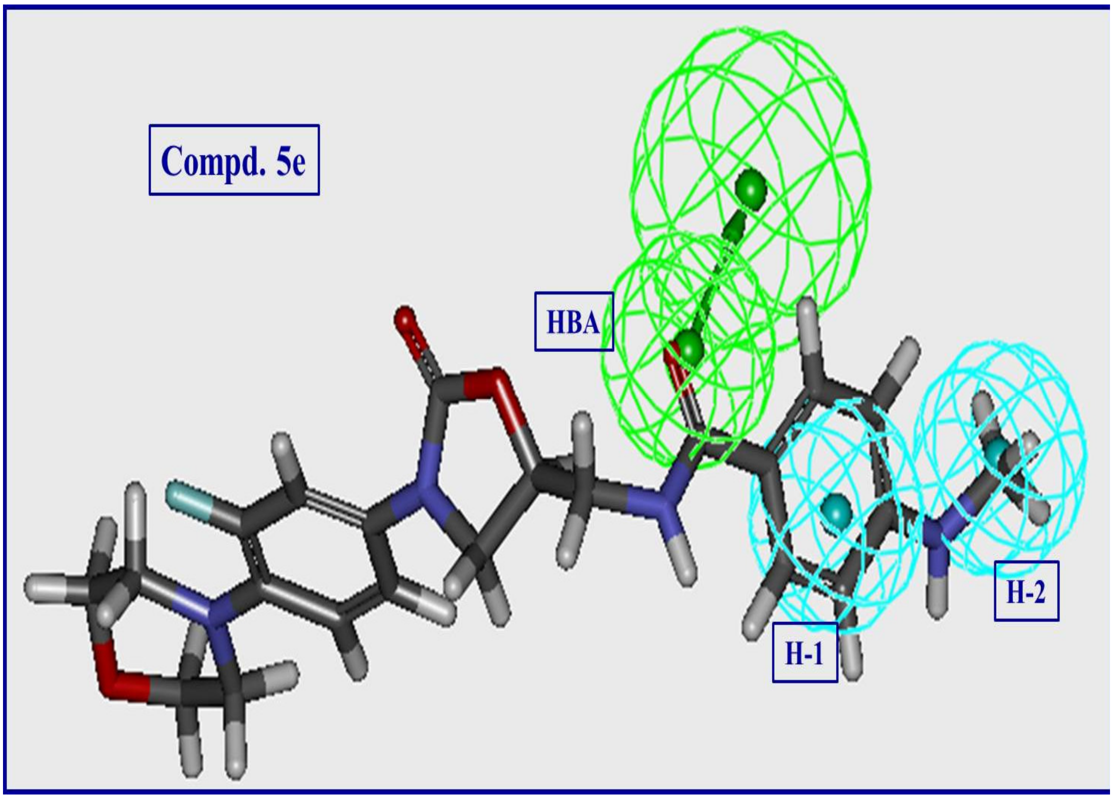
Compd. 5e
HBA
H-1
H-2

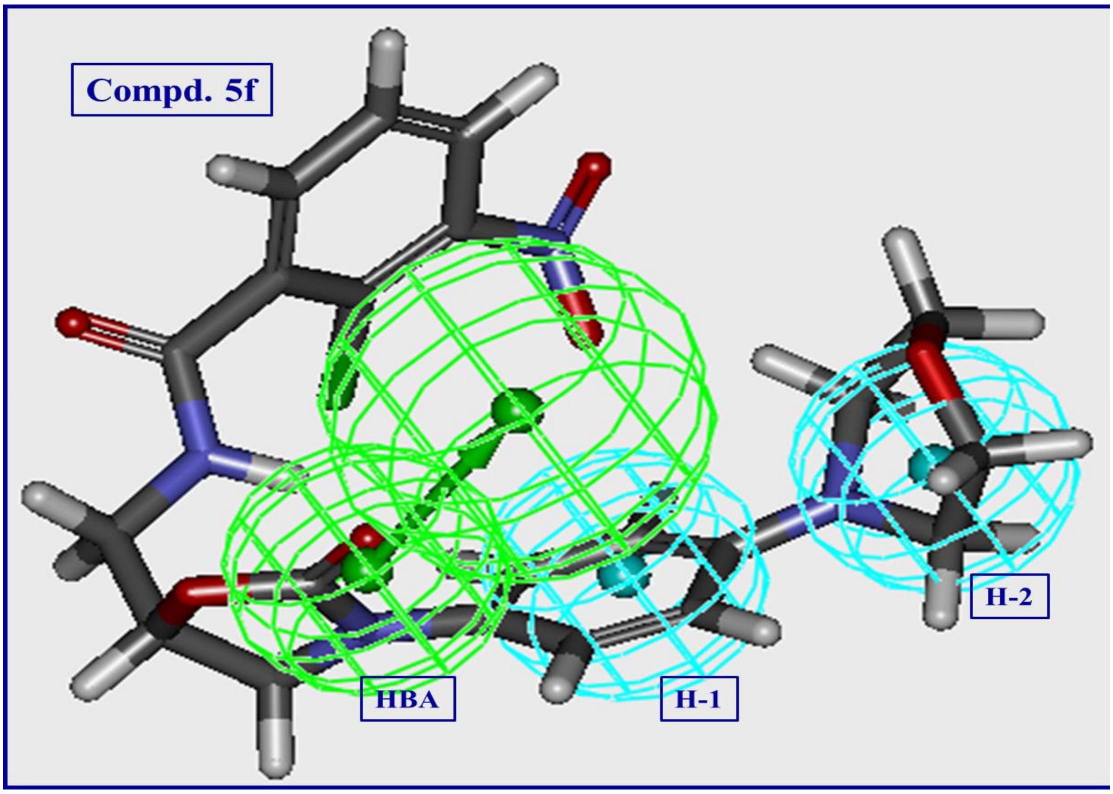
Compd. 5f
HBA
H-1
H-2

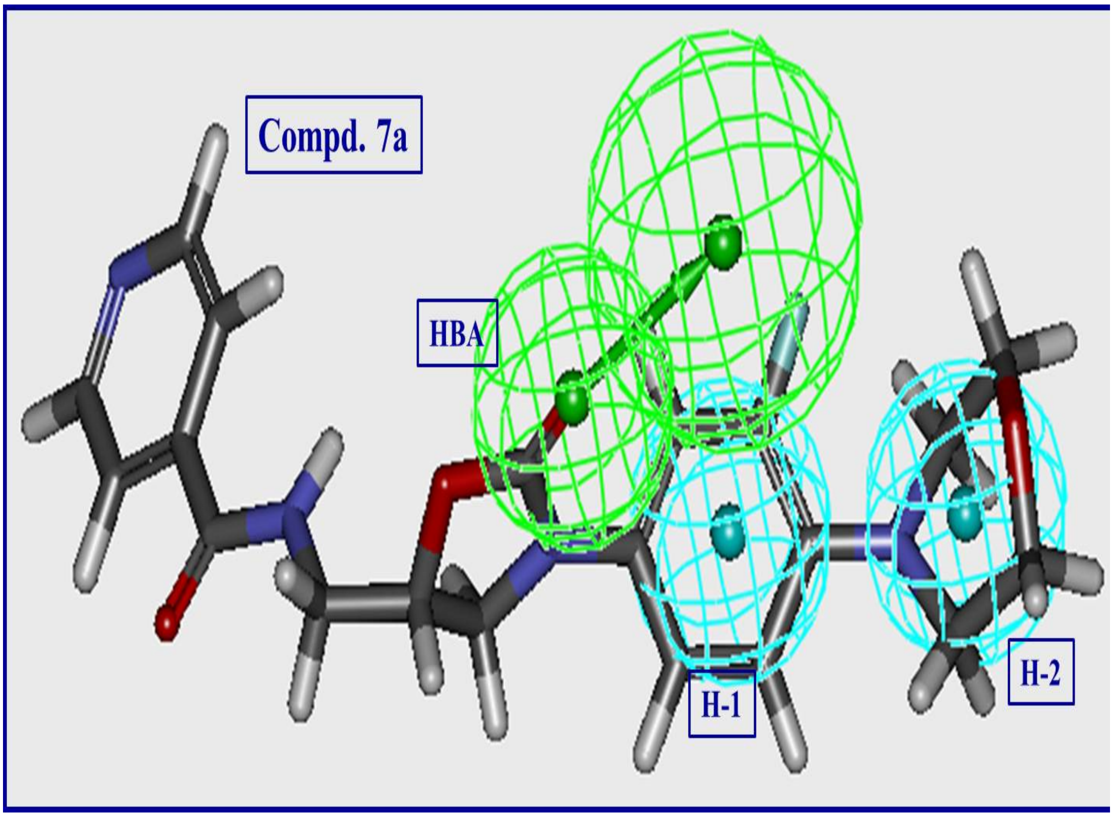
Compd. 7a
HBA
H-1
H-2

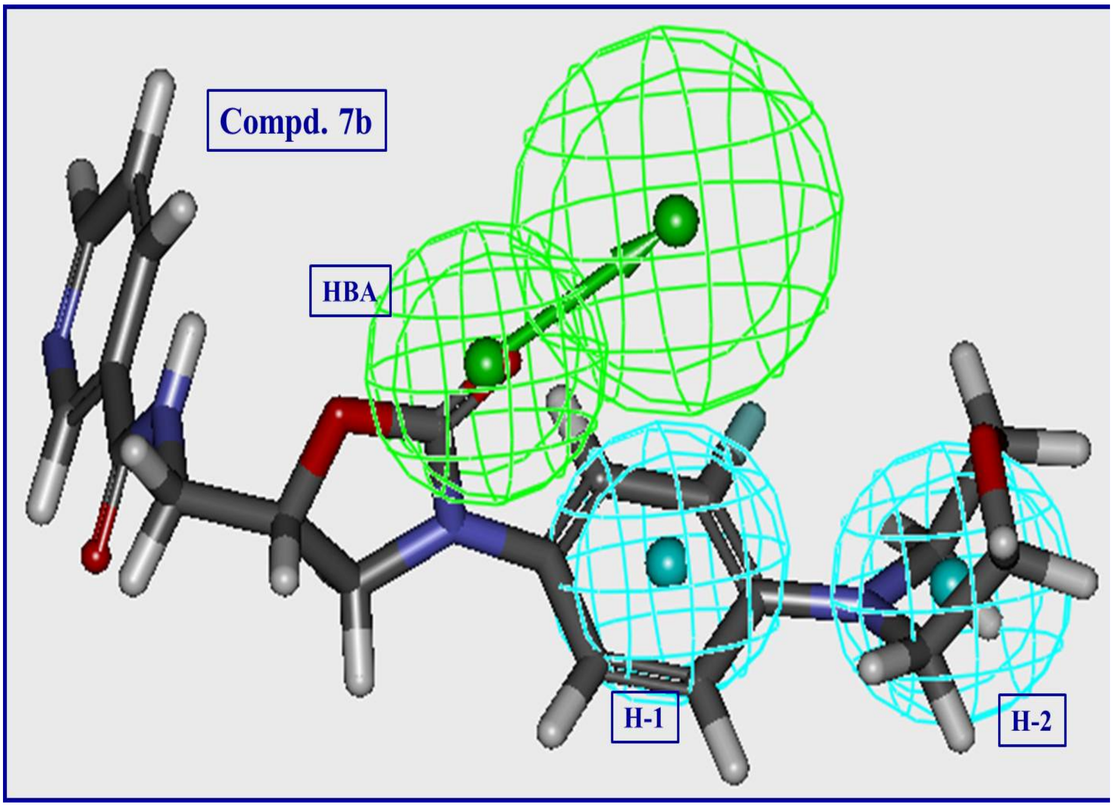
Compd. 7b
HBA
H-1
H-2

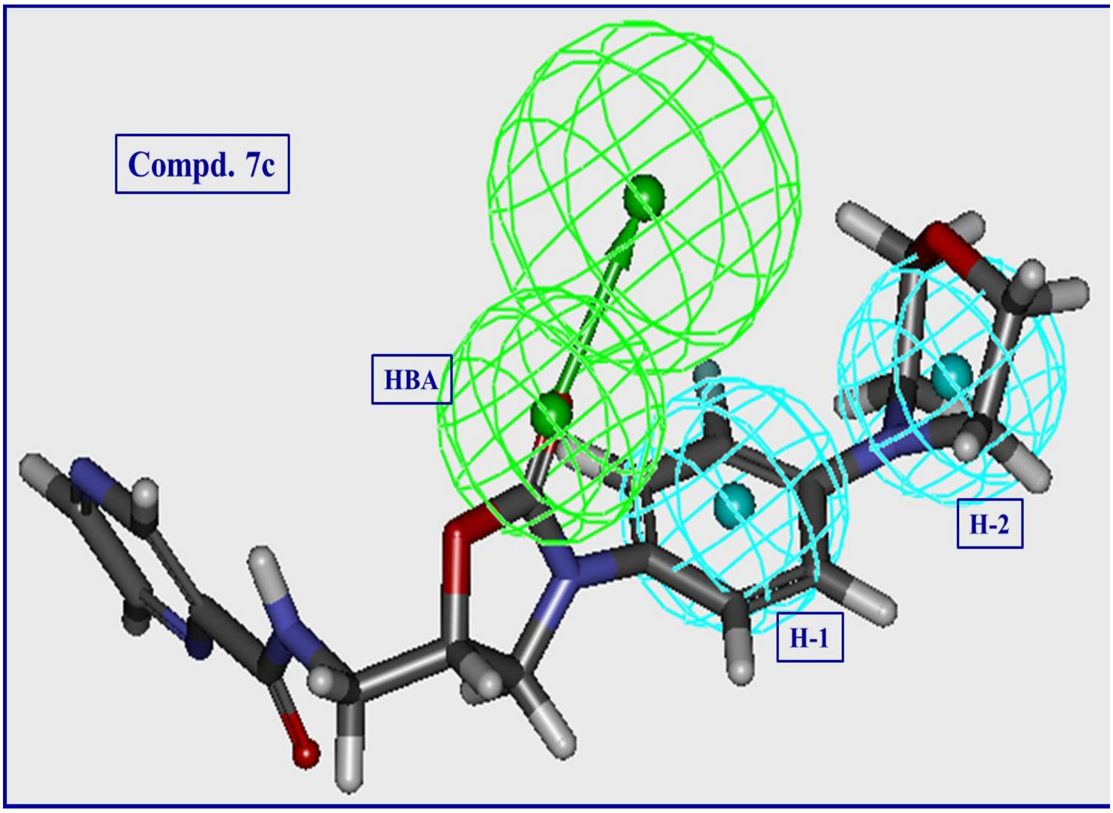
Compd. 7c
HBA
H-1
H-2

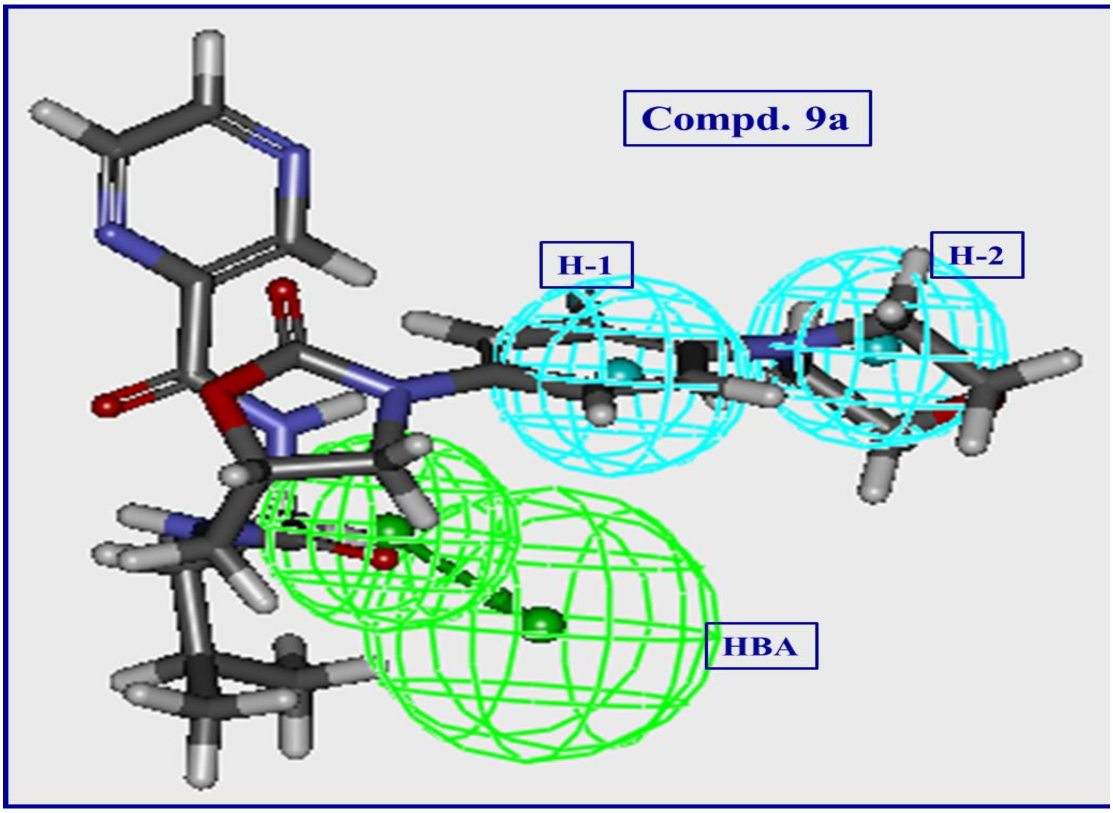
Compd. 9a
H-1
H-2
HBA

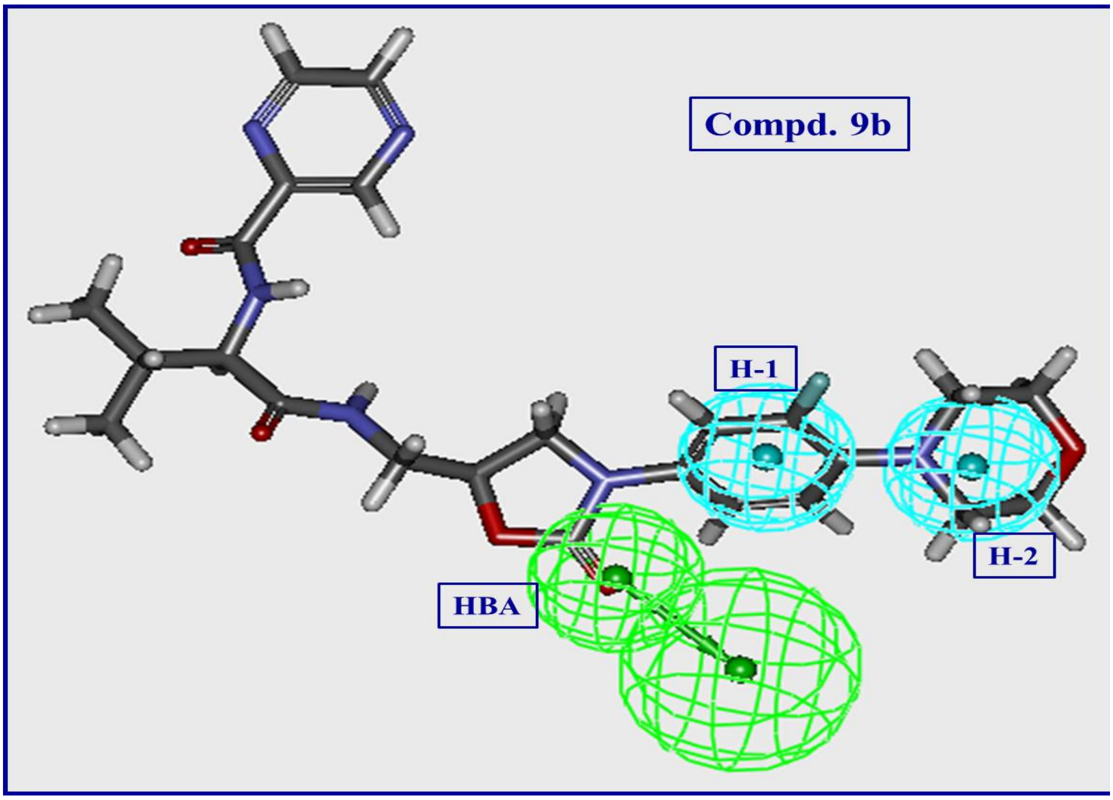
Compd. 9b
H-1
H-2
HBA

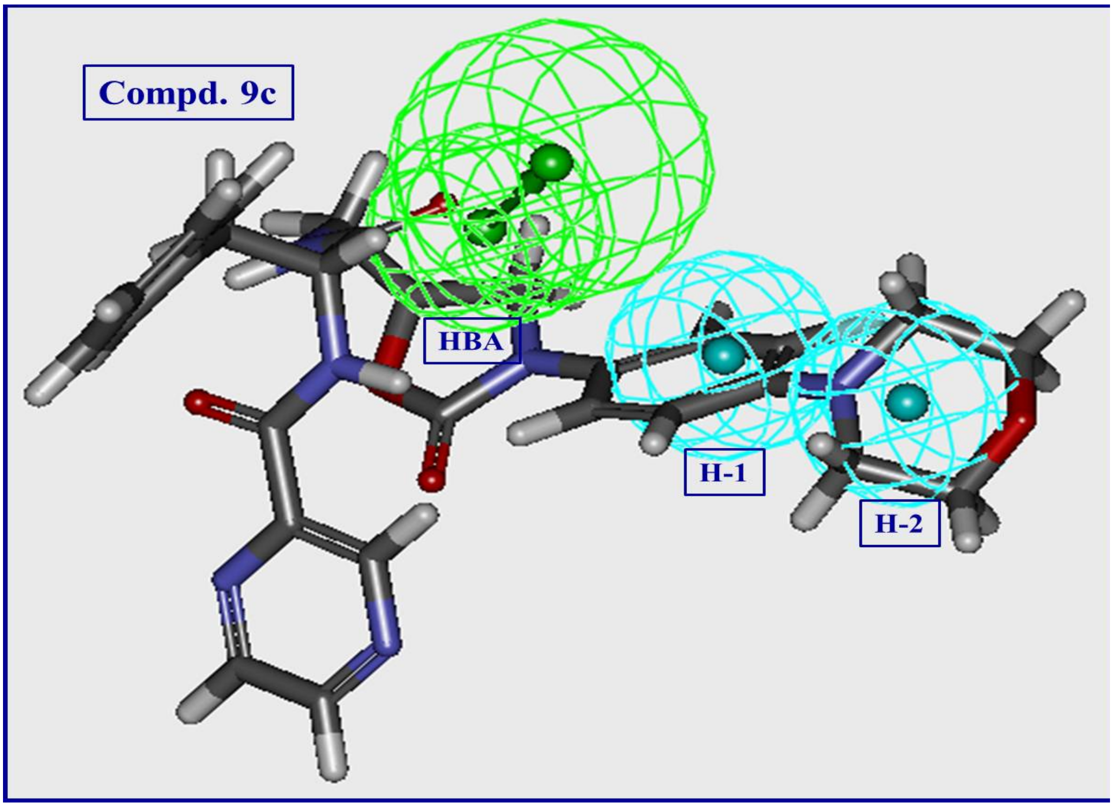
Compd. 9c
HBA
H-1
H-2

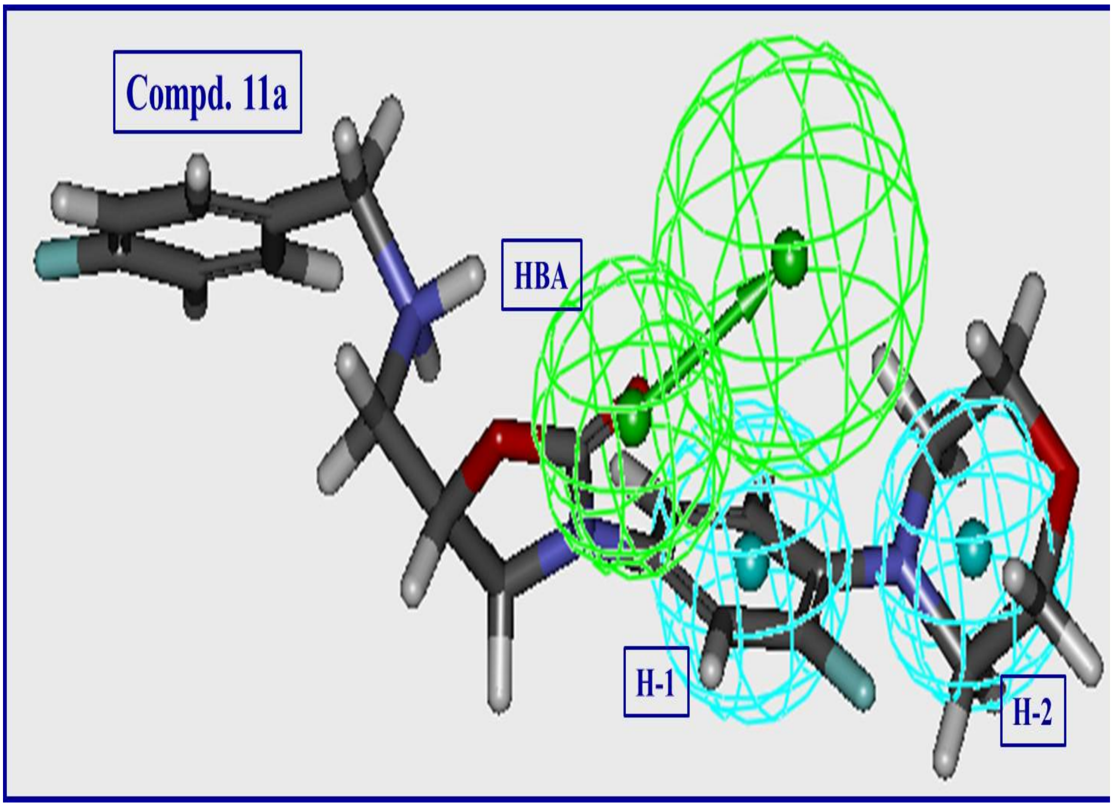
Compd. 11a
HBA
H-1
H-2

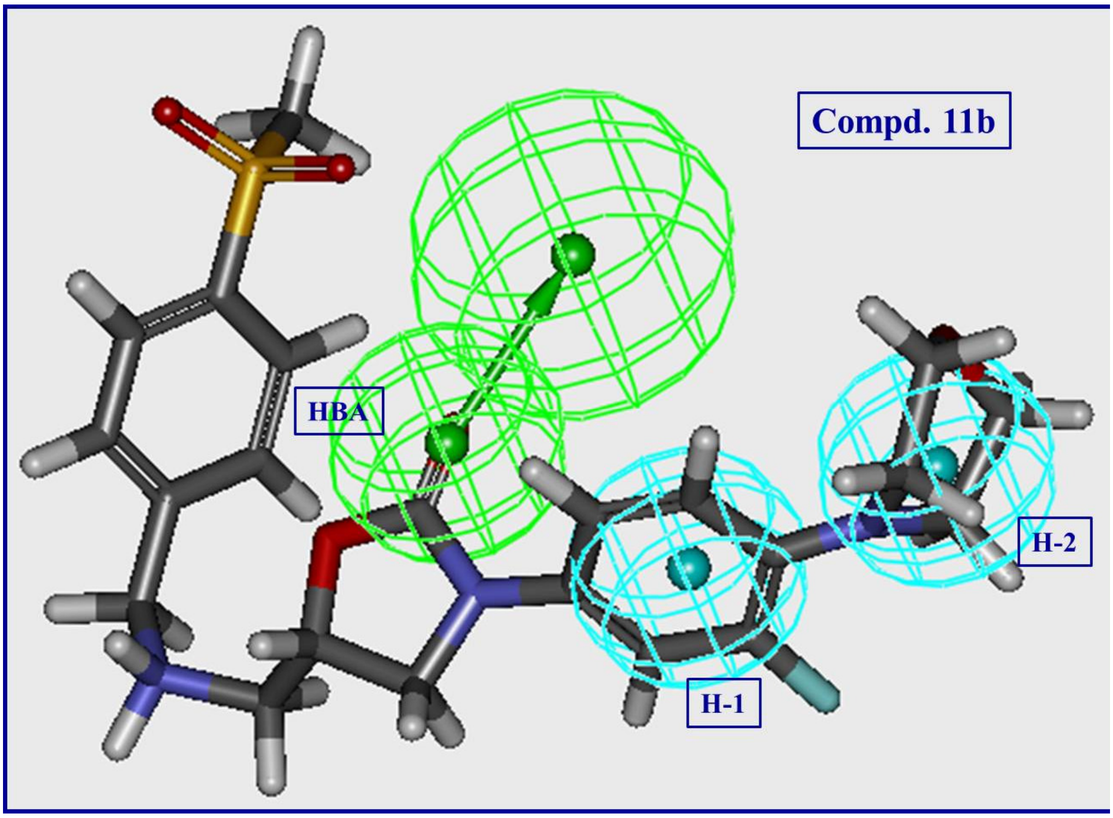
Compd. 11b
HBA
H-2
H-1

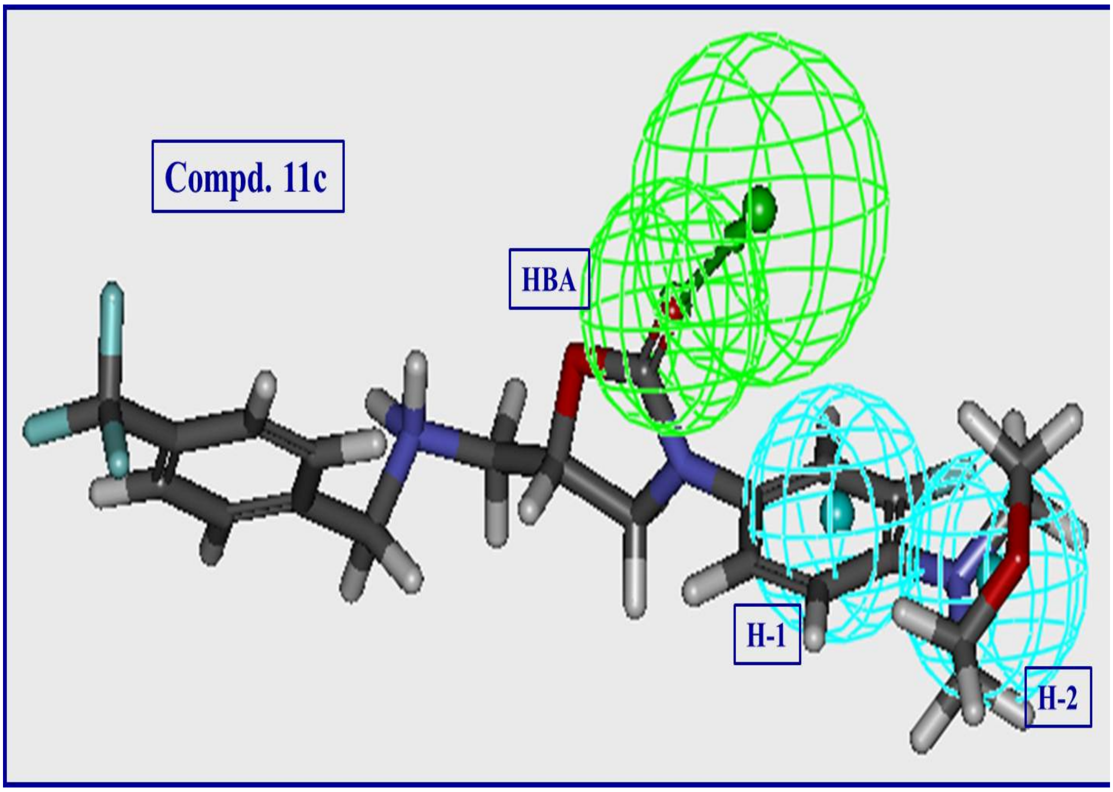
Compd. 11c
HBA
H-1
H-2

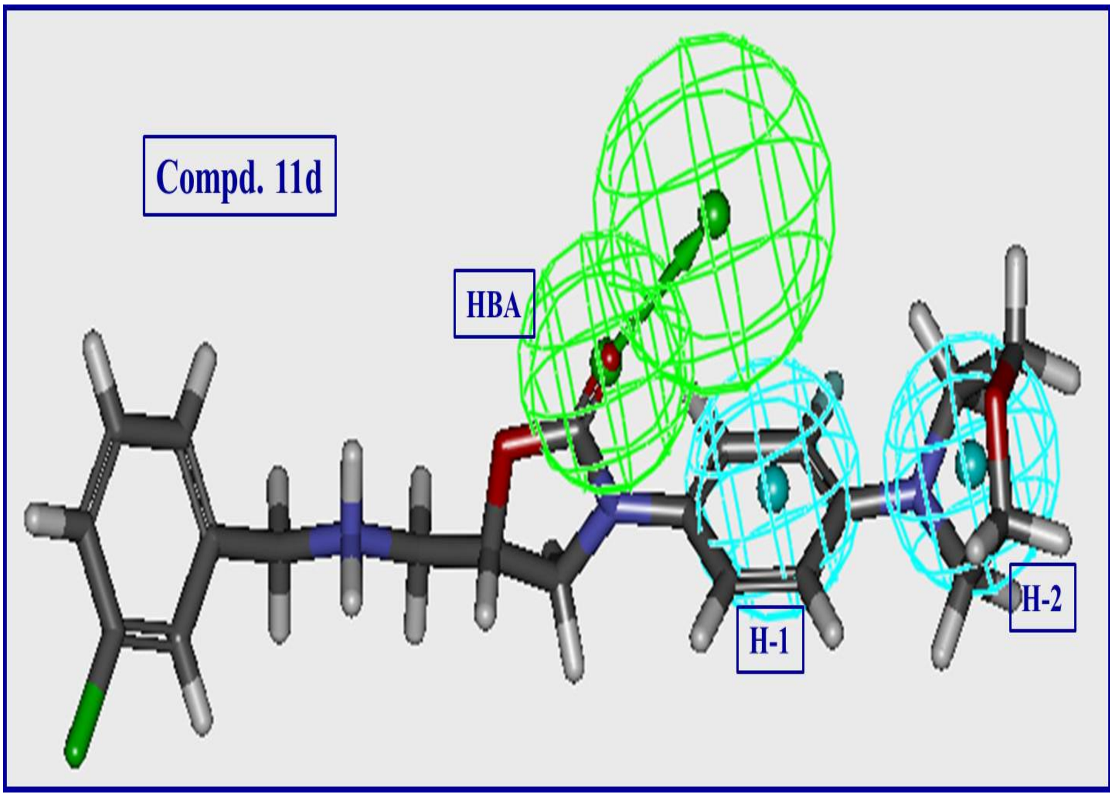
Compd. 11d
HBA
H-1
H-2

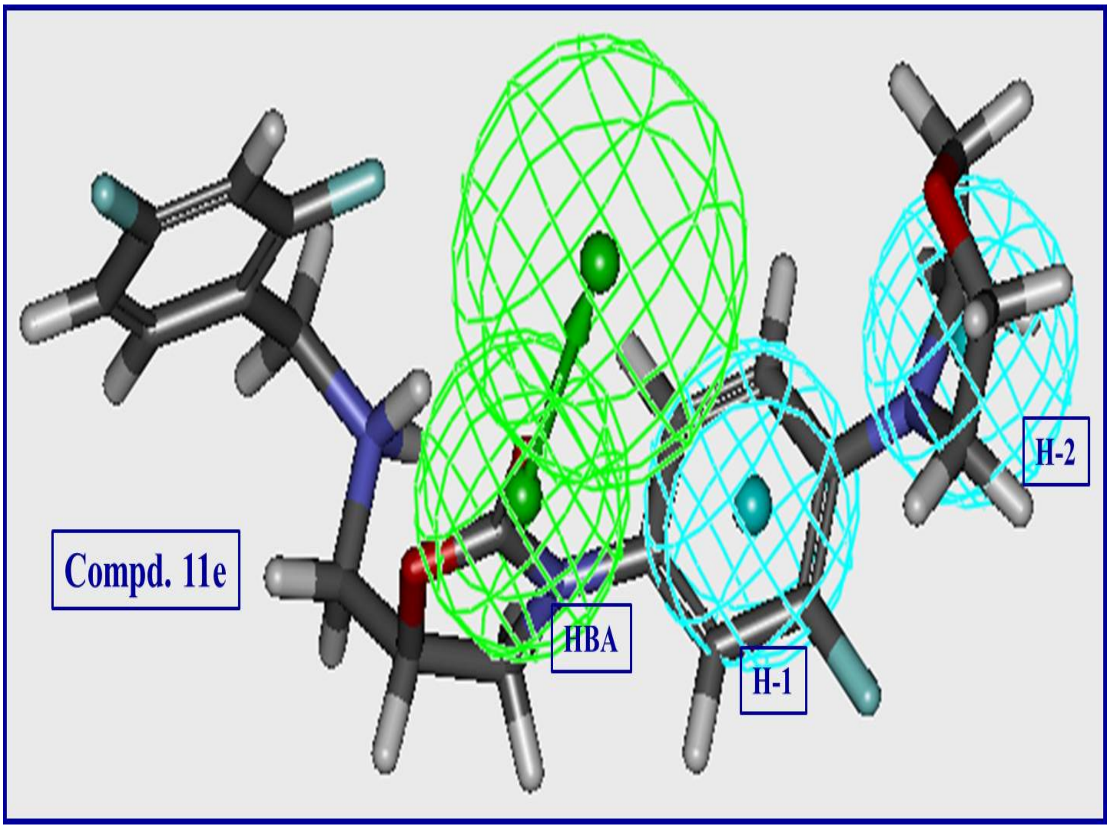
Compd. 11e
HBA
H-1
H-2

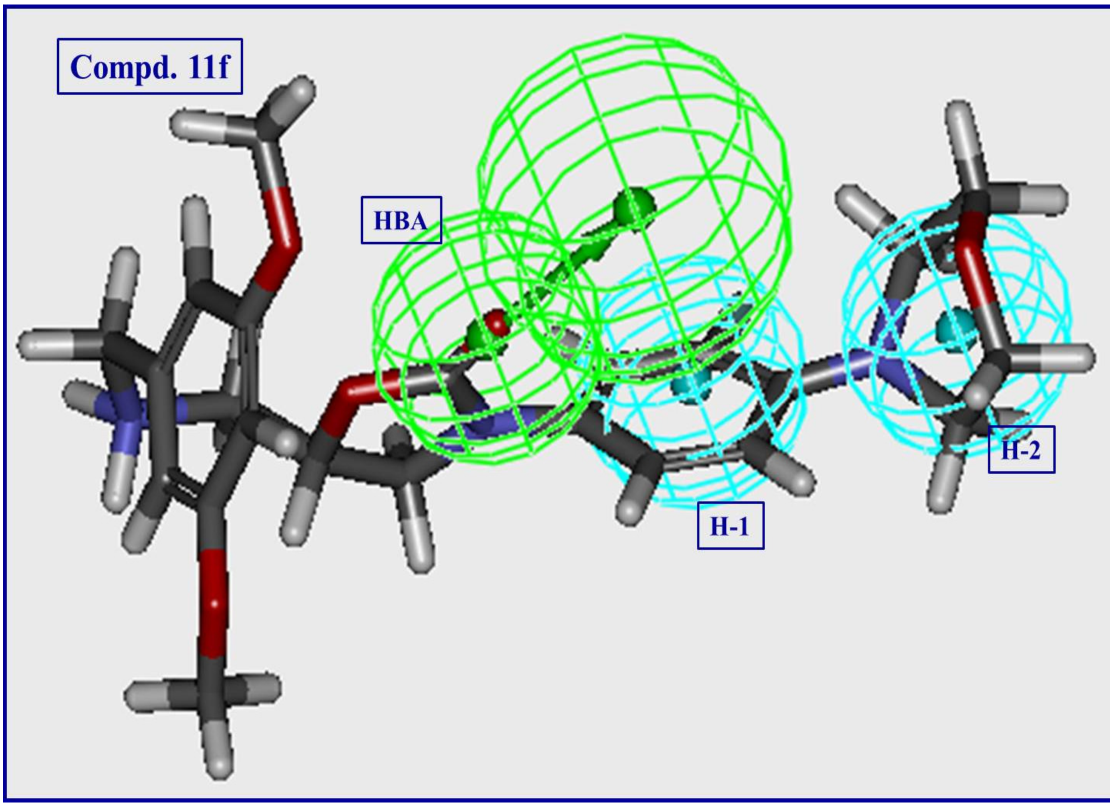
Compd. 11f
HBA
H-1
H-2

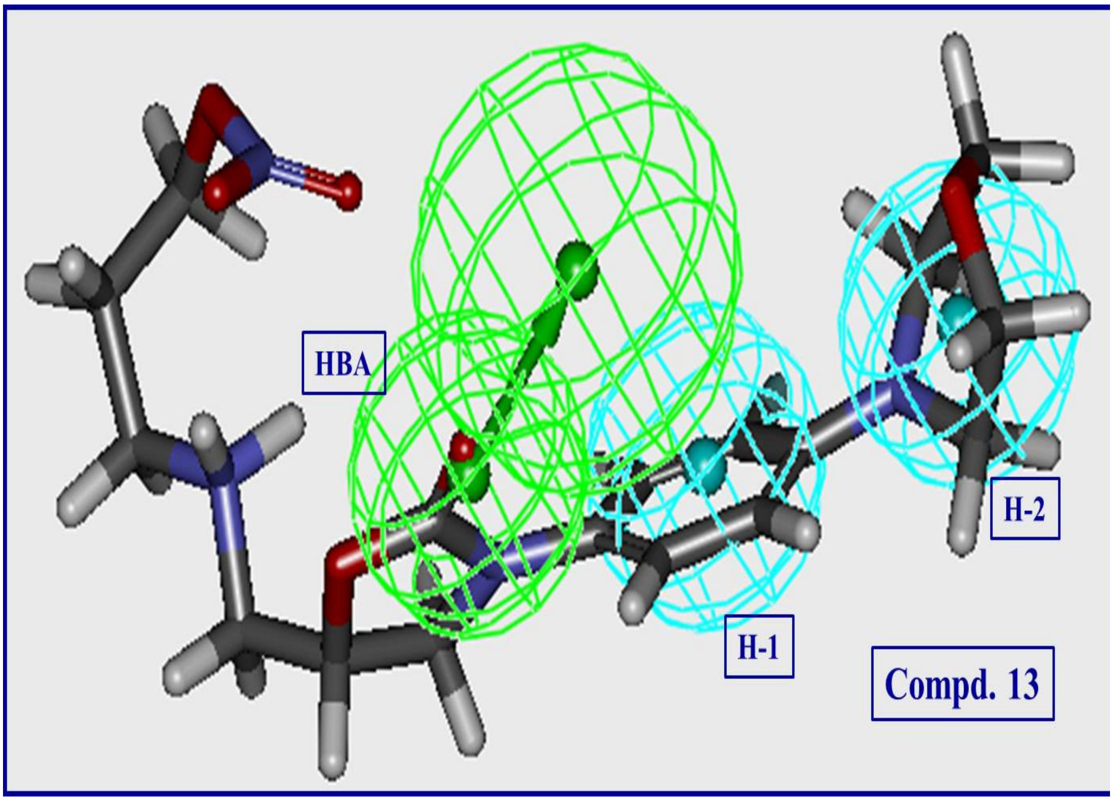
HBA
H-2
H-1
Compd. 13

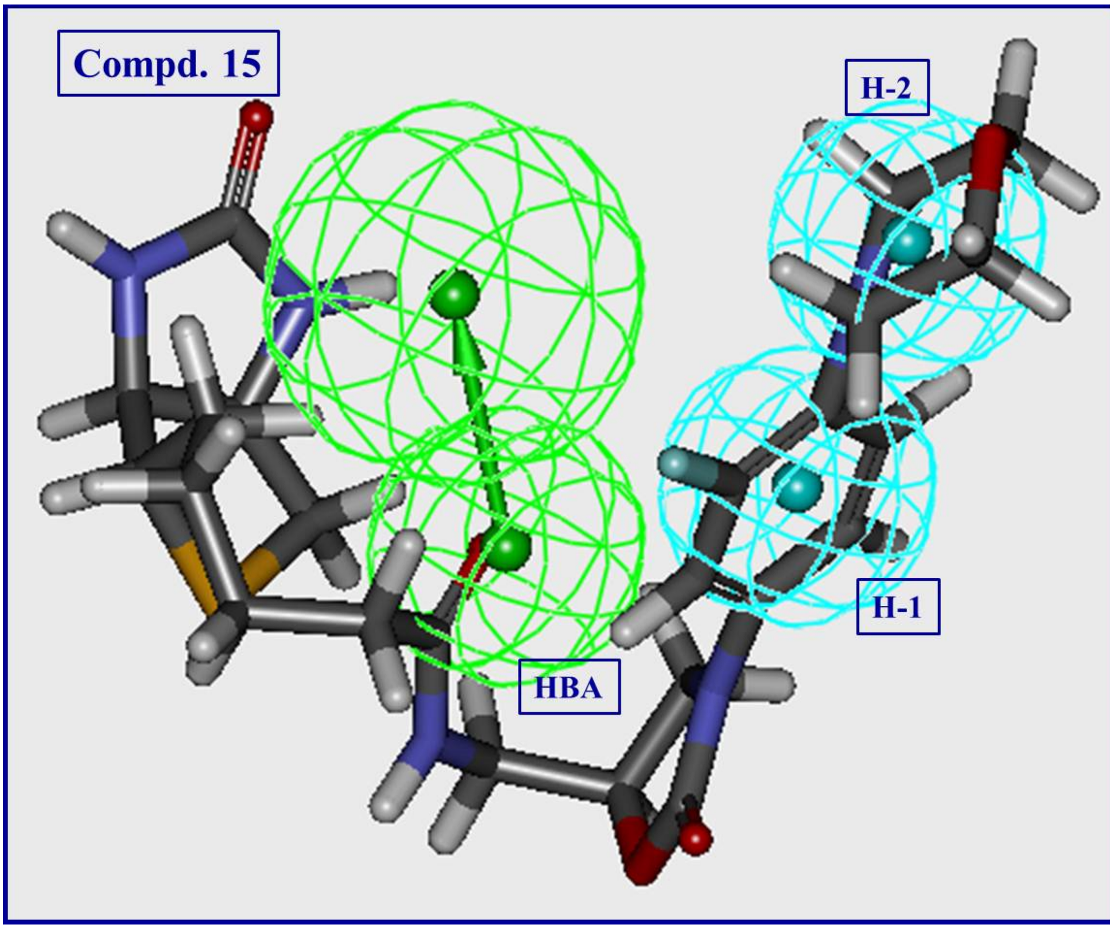

Fig. S5. 3D-pharmacophore model mapped on the tested compounds against *S. aureus*.

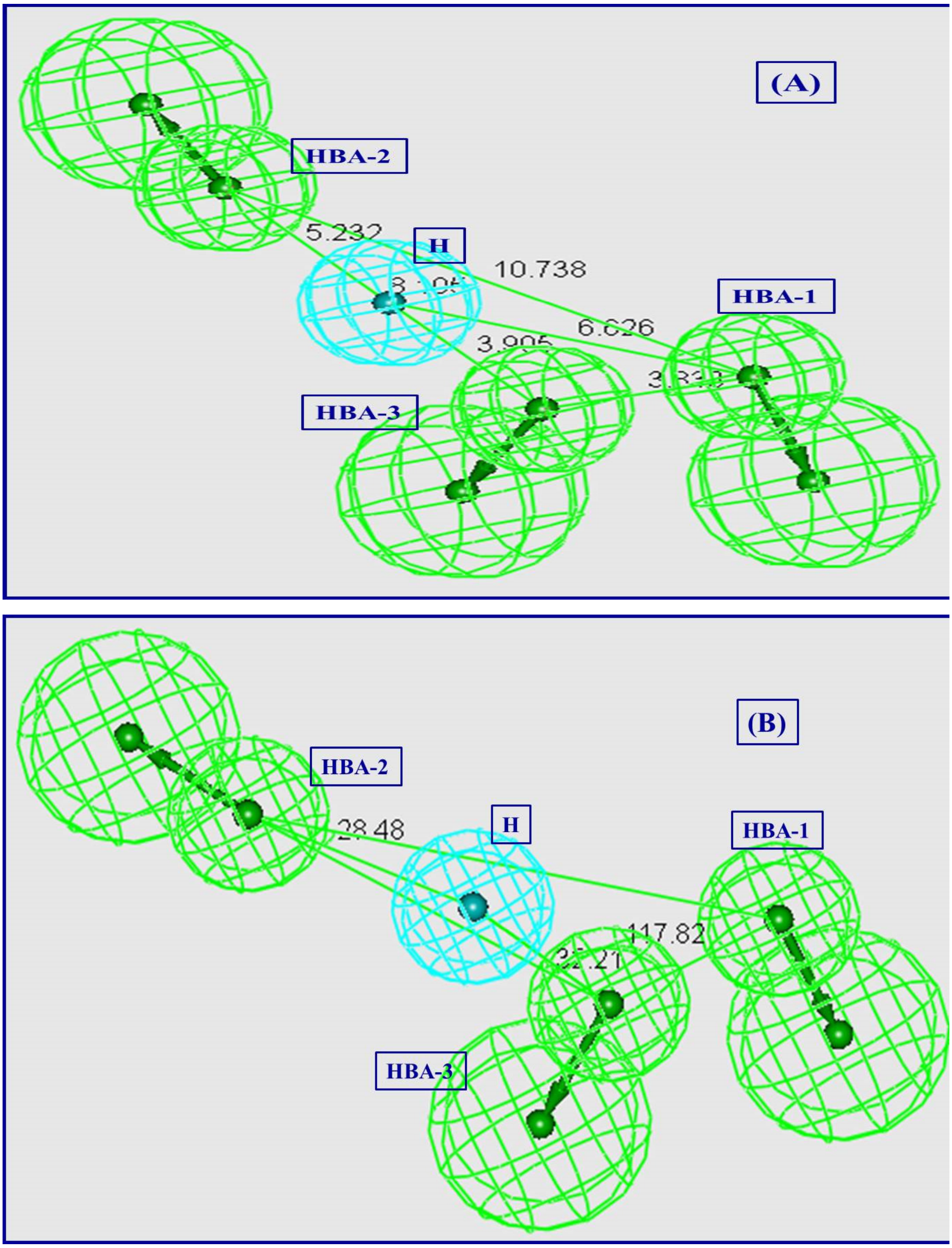

Fig. S6. (A) Constraint distances "HBA-1 – HBA-2 = 10.738, HBA-1 – HBA-3 = 3.833, HBA-2 – HBA-3 = 8.105, HBA-1 – H = 6.626, HBA-2 – H = 5.232, HBA-3 – H = 3.905 Å", **(B)** Constraint angle "HBA-1 – HBA-2 – HBA-3 = 17.17, HBA-1 – HBA-2 – H = 28.48, HBA-1 – HBA-3 – H = 117.82, HBA-2 – HBA-2 – H = 32.21 °" of the generated 3D-pharmacophore for the tested compounds against *B. subtilis*.

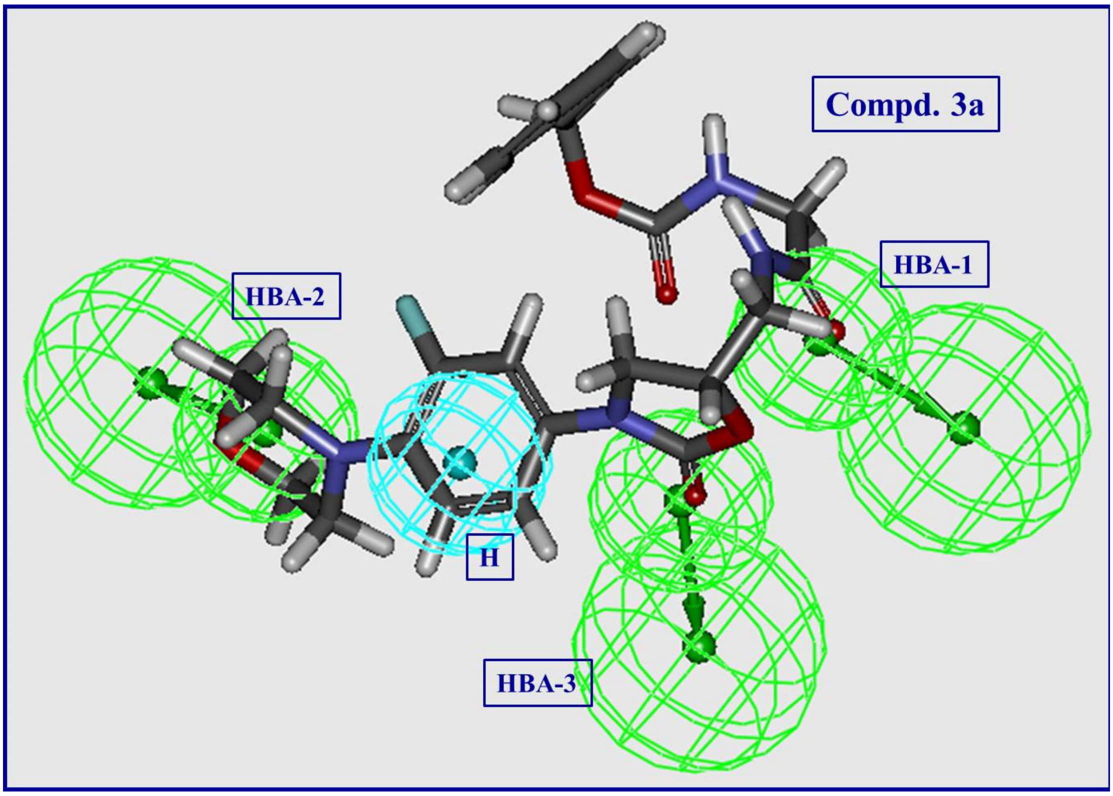
Compd. 3a
HBA-1
HBA-2
H
HBA-3

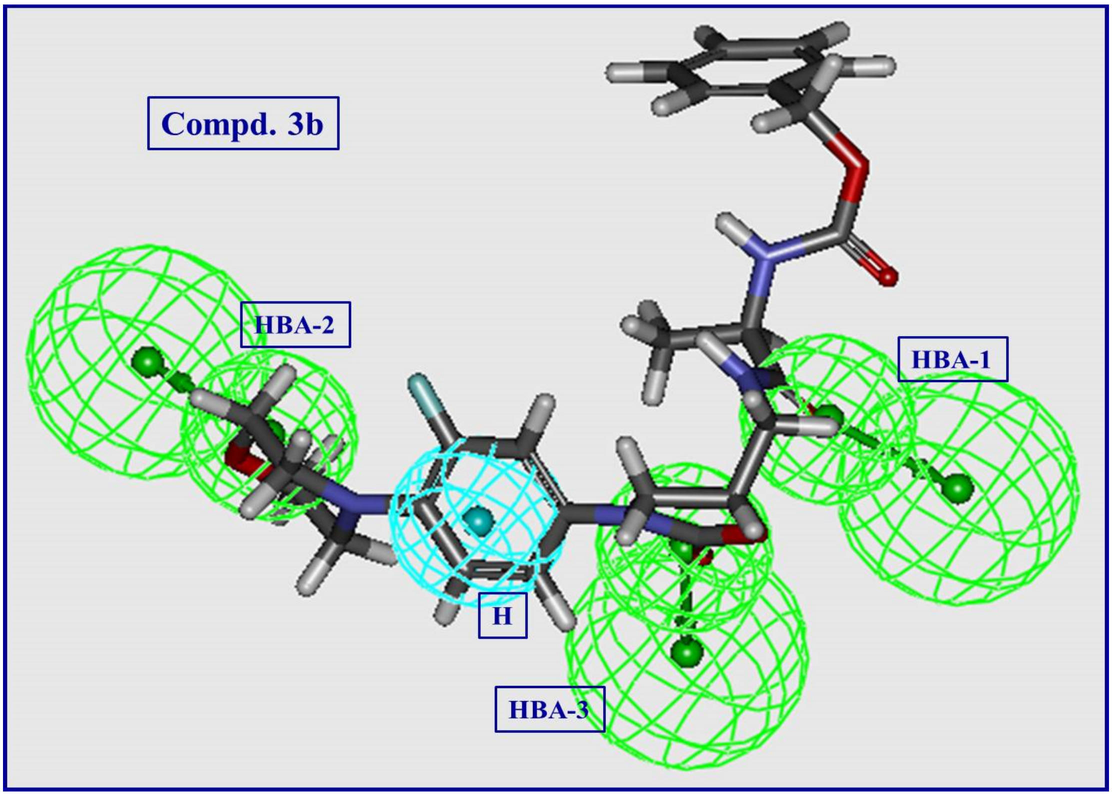
Compd. 3b
HBA-2
HBA-1
H
HBA-3

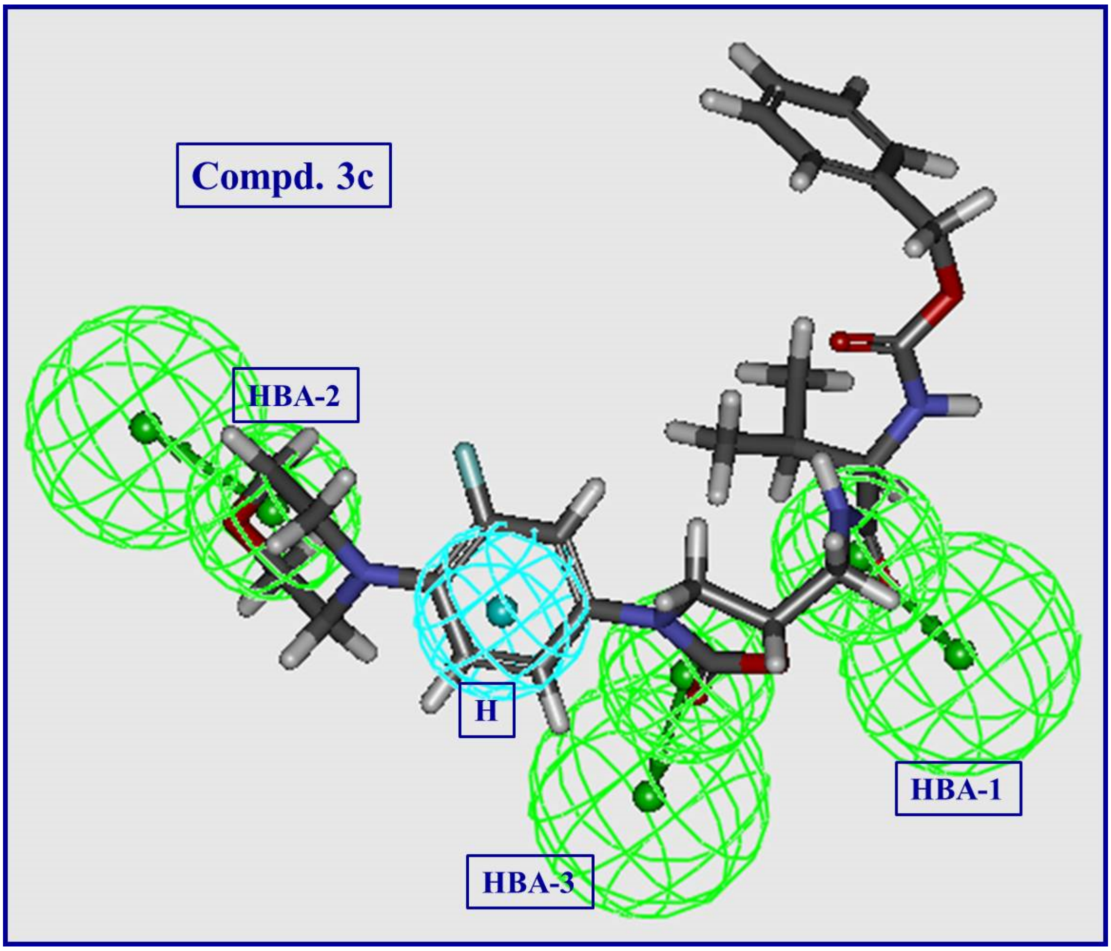

Compd. 3c
HBA-2
H
HBA-3
HBA-1

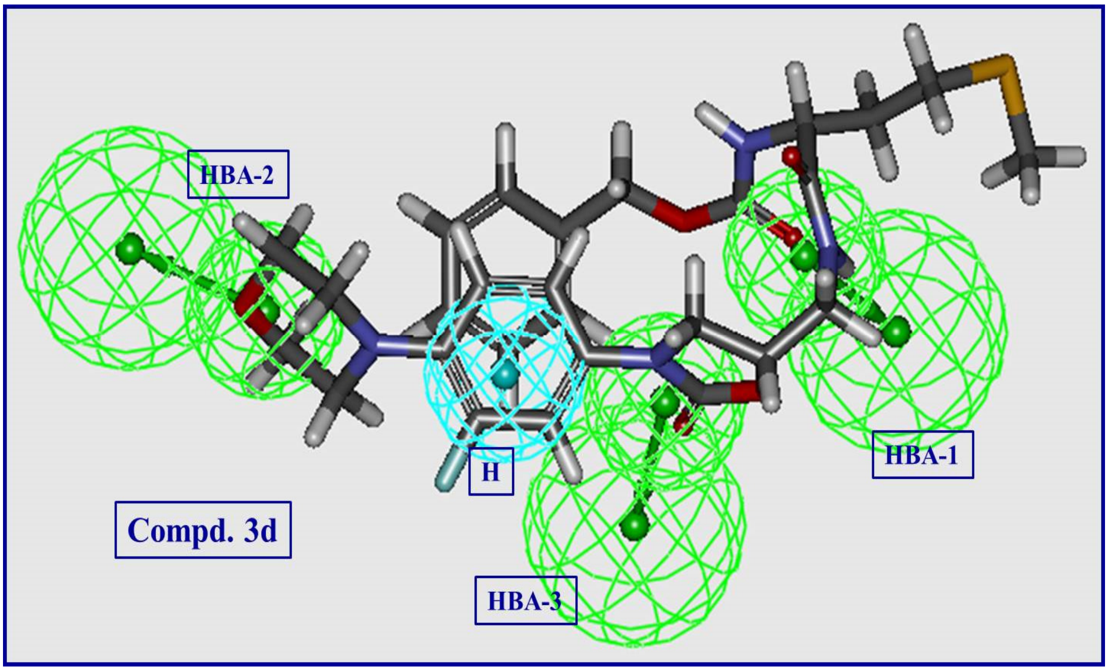

HBA-2
H
Compd. 3d
HBA-3
HBA-1

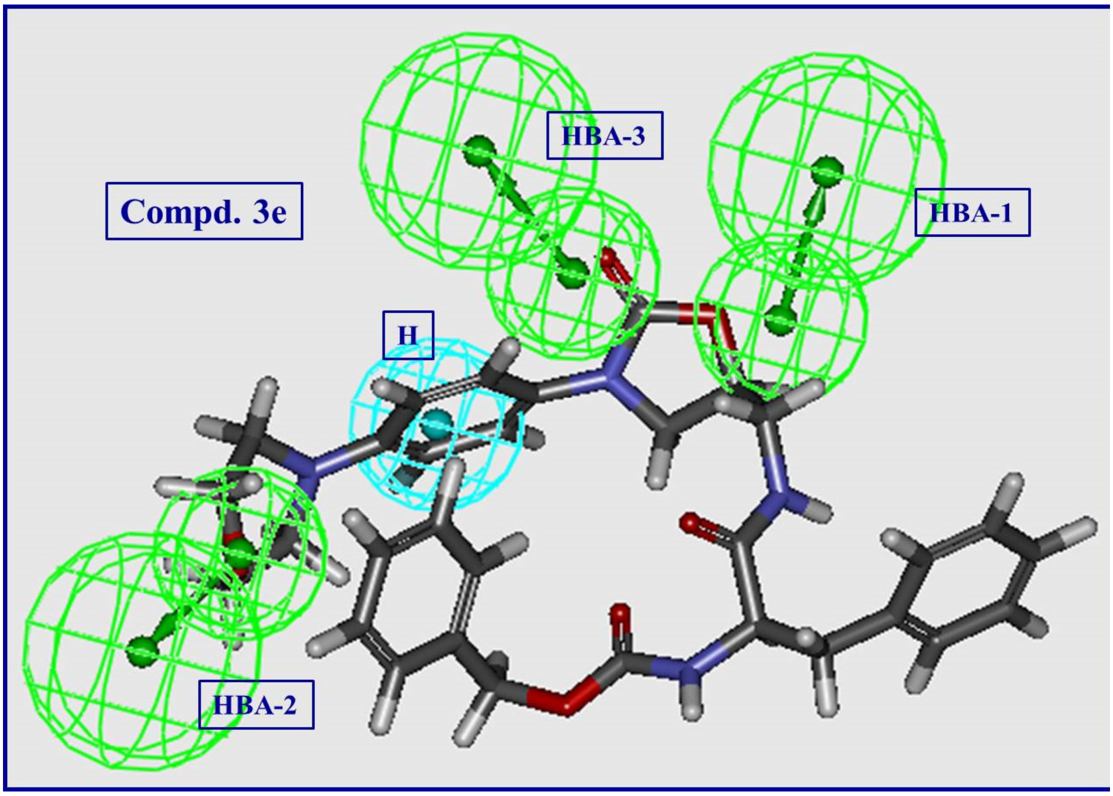
Compd. 3e
HBA-3
HBA-1
H
HBA-2

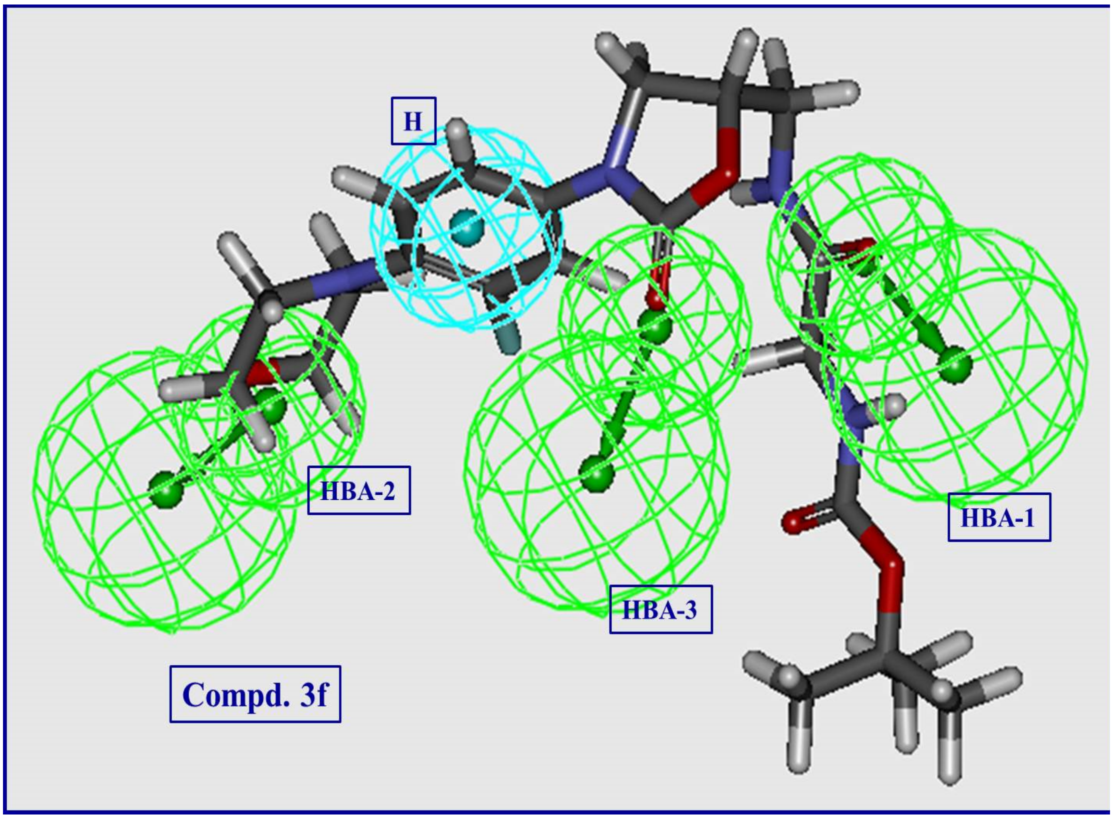
H
HBA-2
HBA-1
HBA-3
Compd. 3f

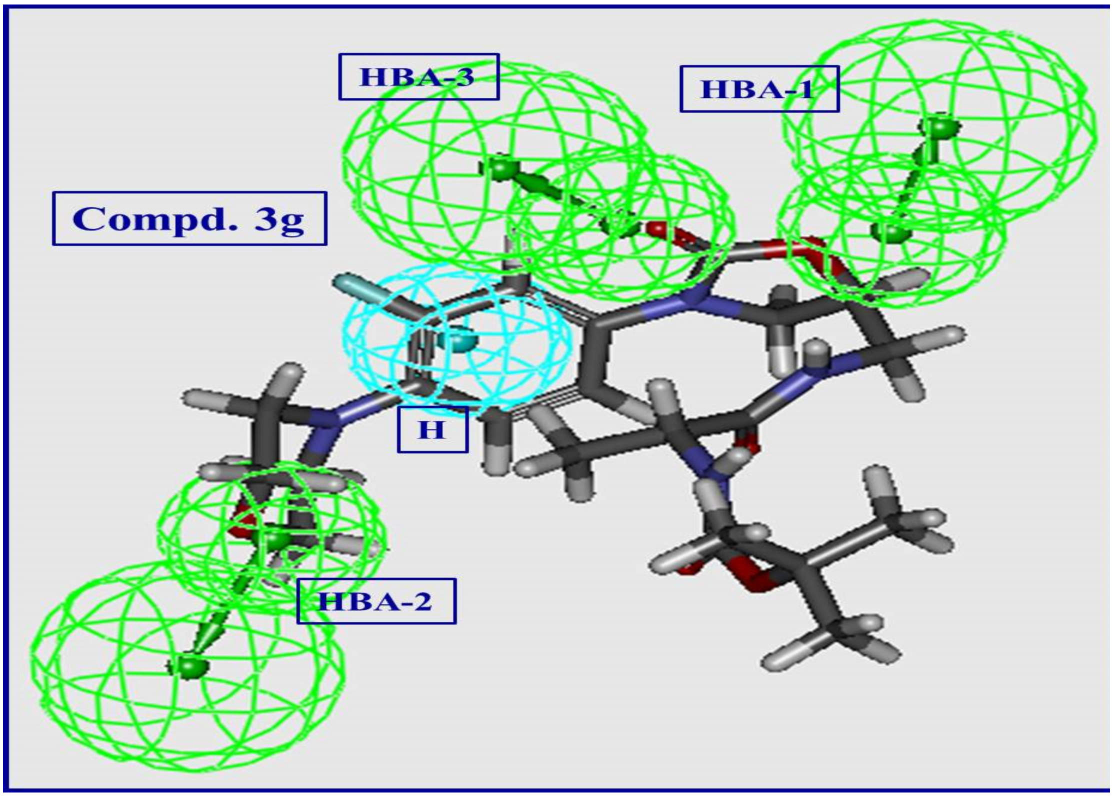
HBA-3
HBA-1
Compd. 3g
H
HBA-2

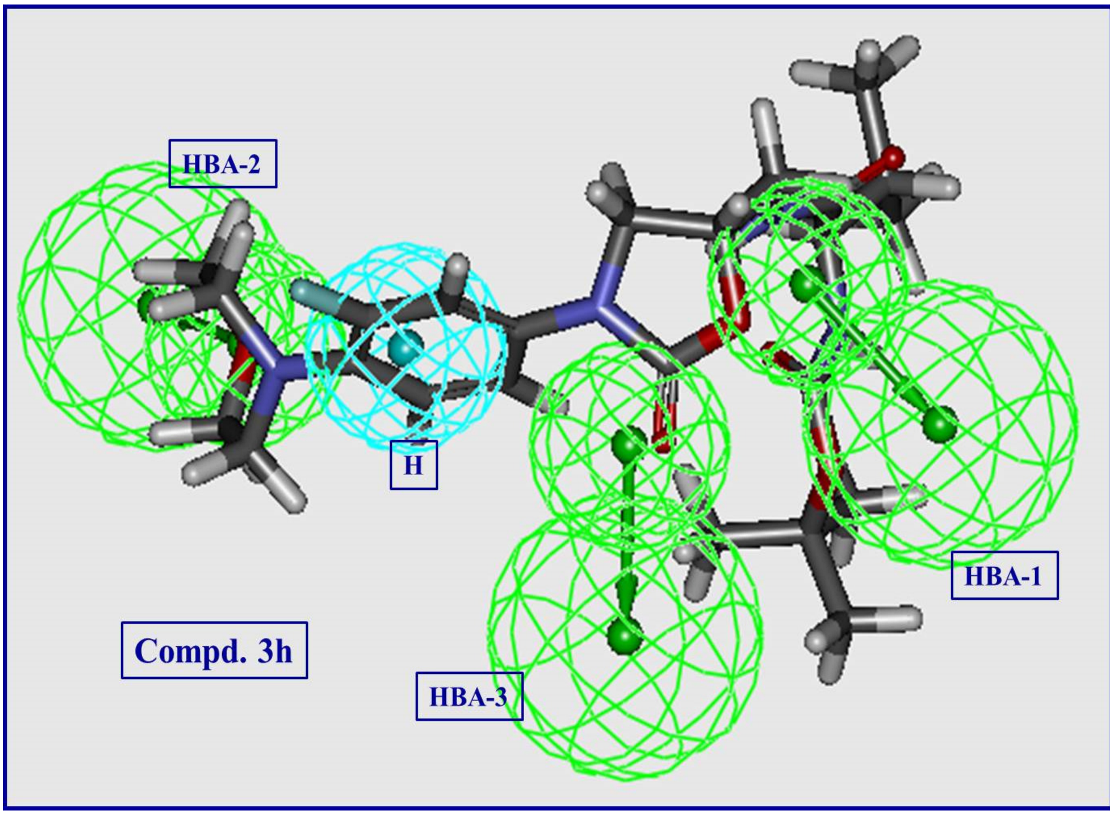
HBA-2
H
HBA-1
Compd. 3h
HBA-3

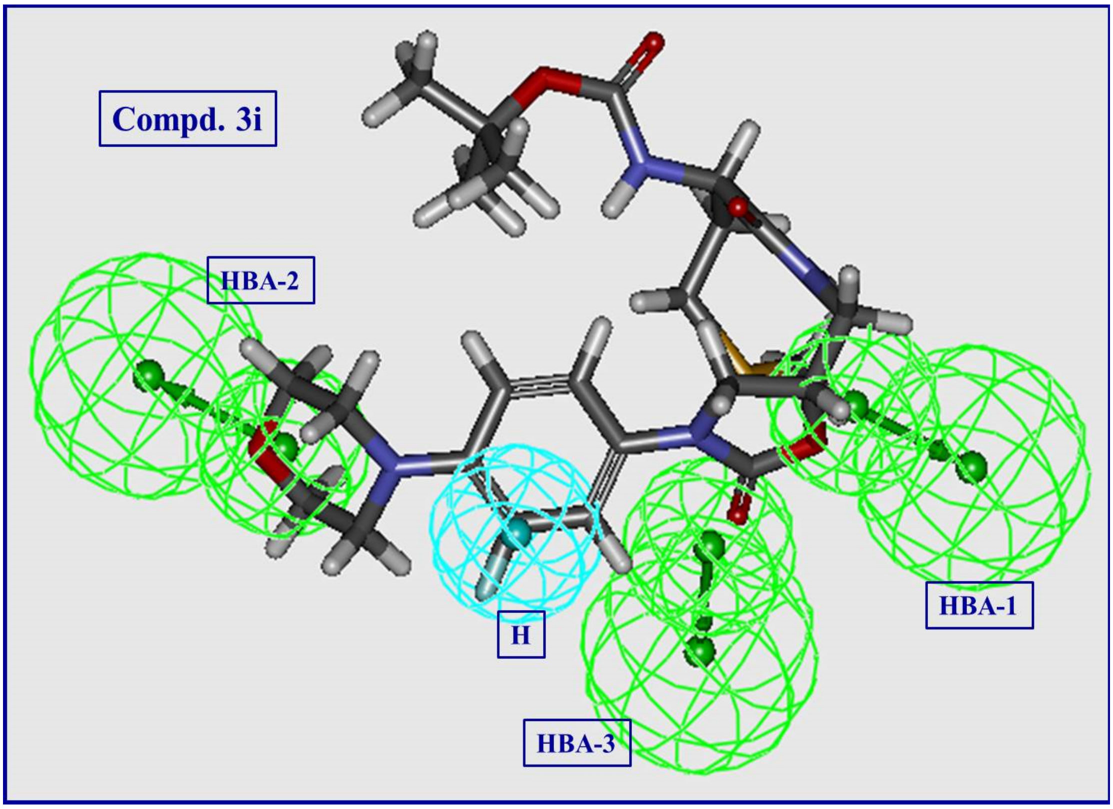
Compd. 3i
HBA-2
H
HBA-3
HBA-1

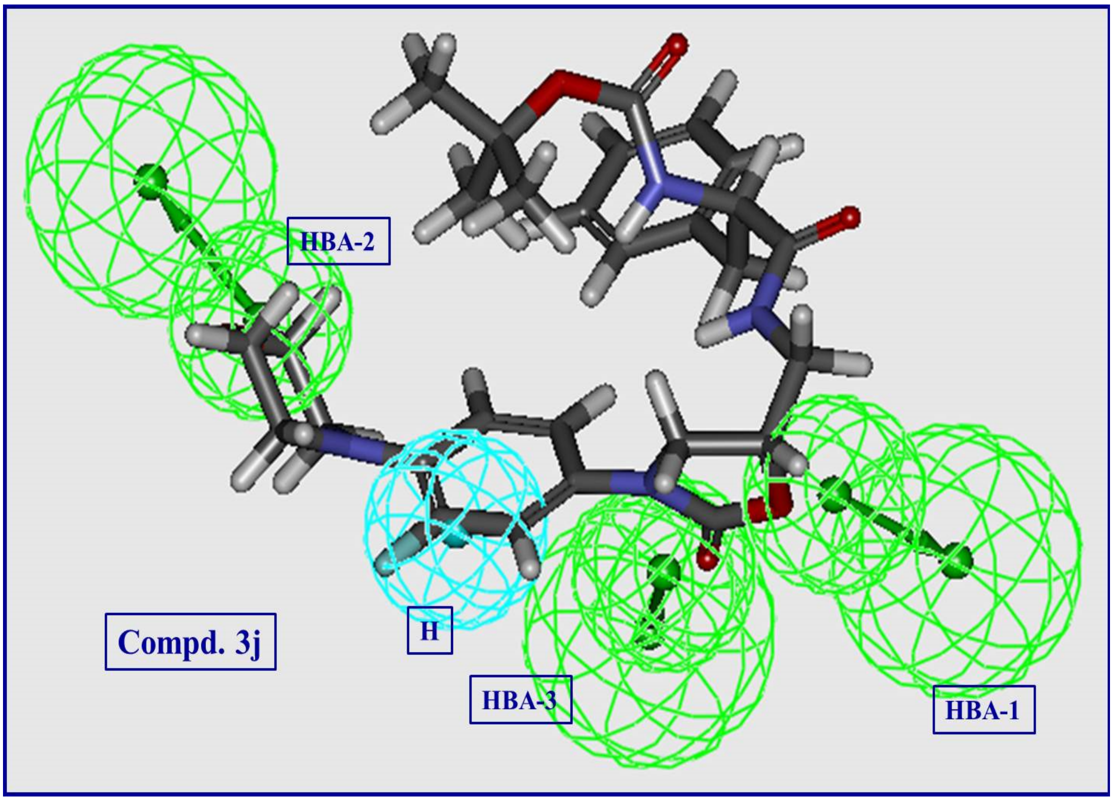
HBA-2
Compd. 3j
H
HBA-3
HBA-1

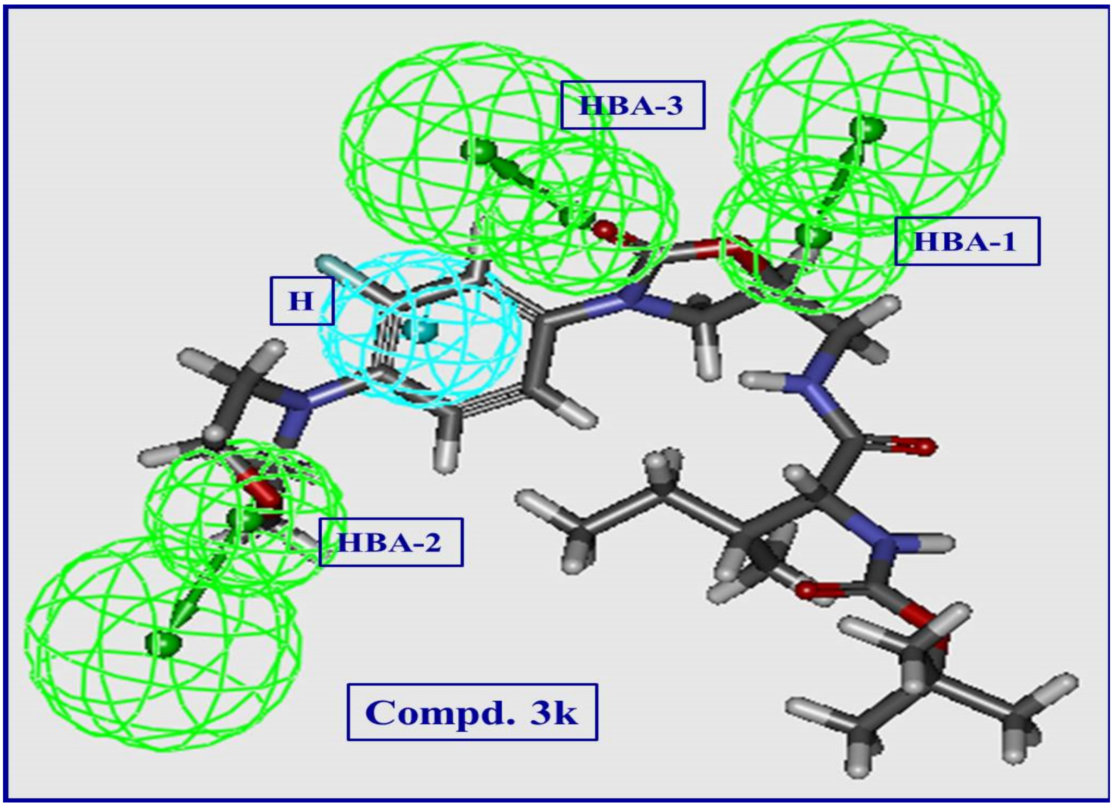
HBA-3
HBA-1
H
HBA-2
Compd. 3k

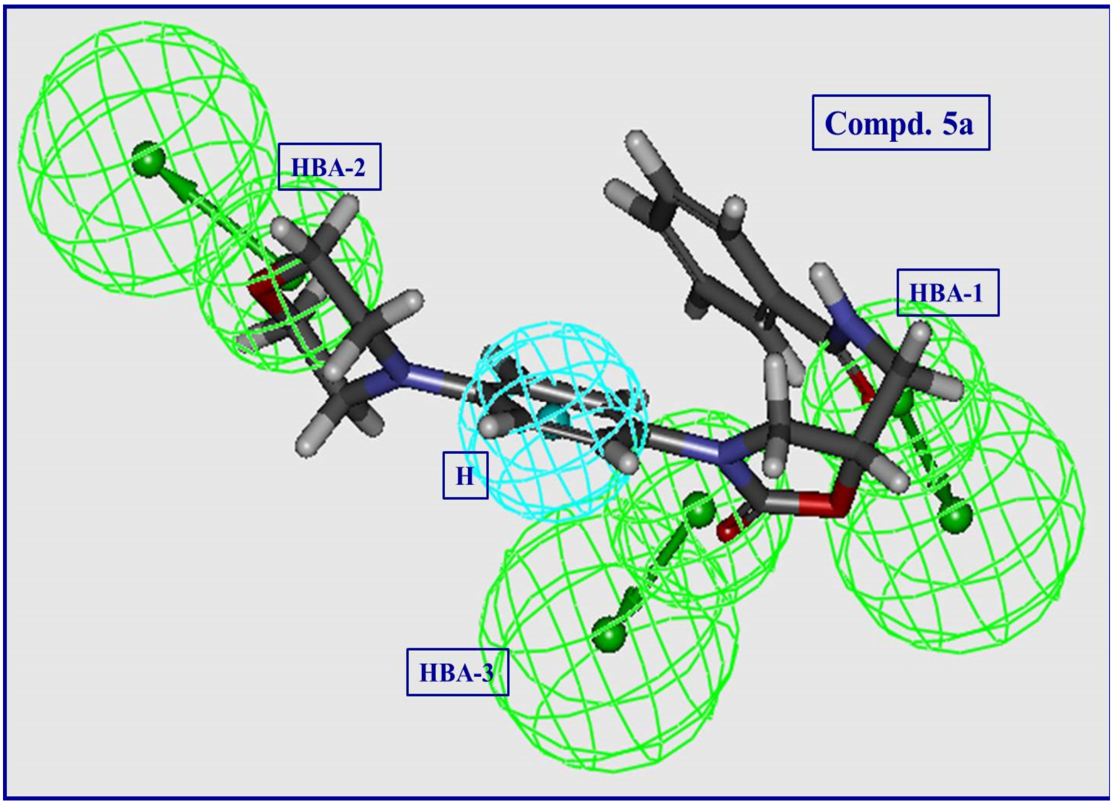
Compd. 5a
HBA-2
HBA-1
H
HBA-3

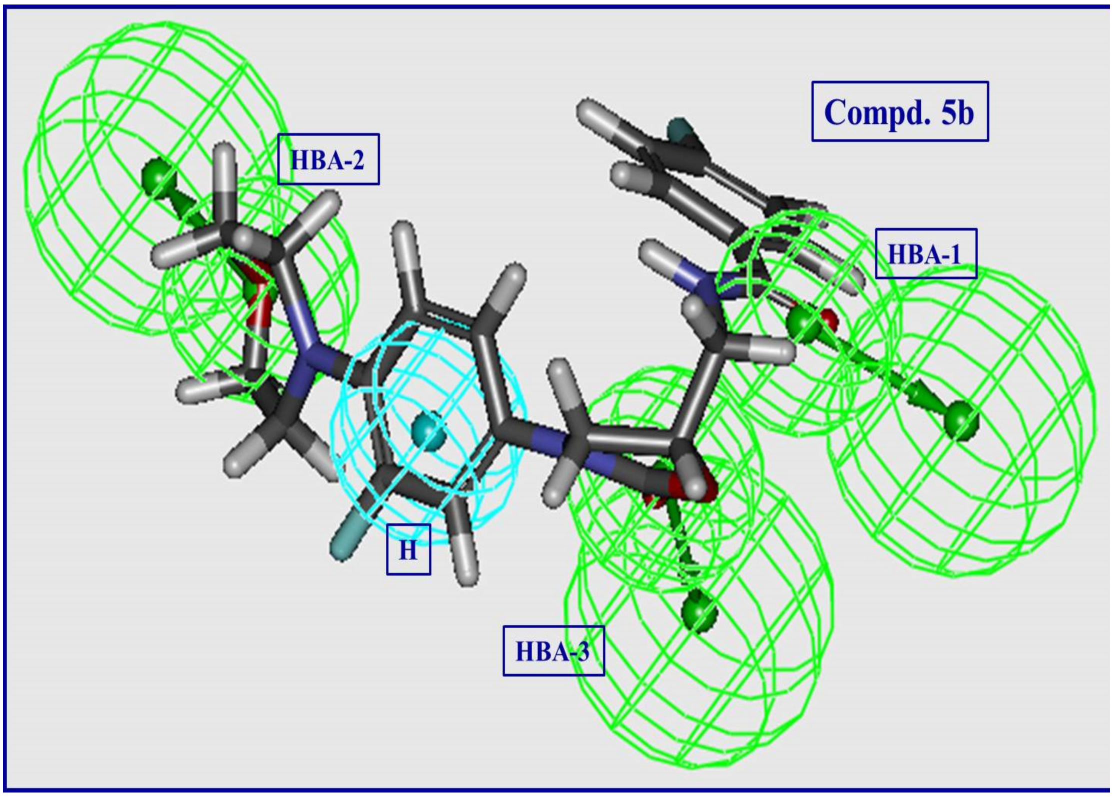
Compd. 5b
HBA-2
HBA-1
H
HBA-3

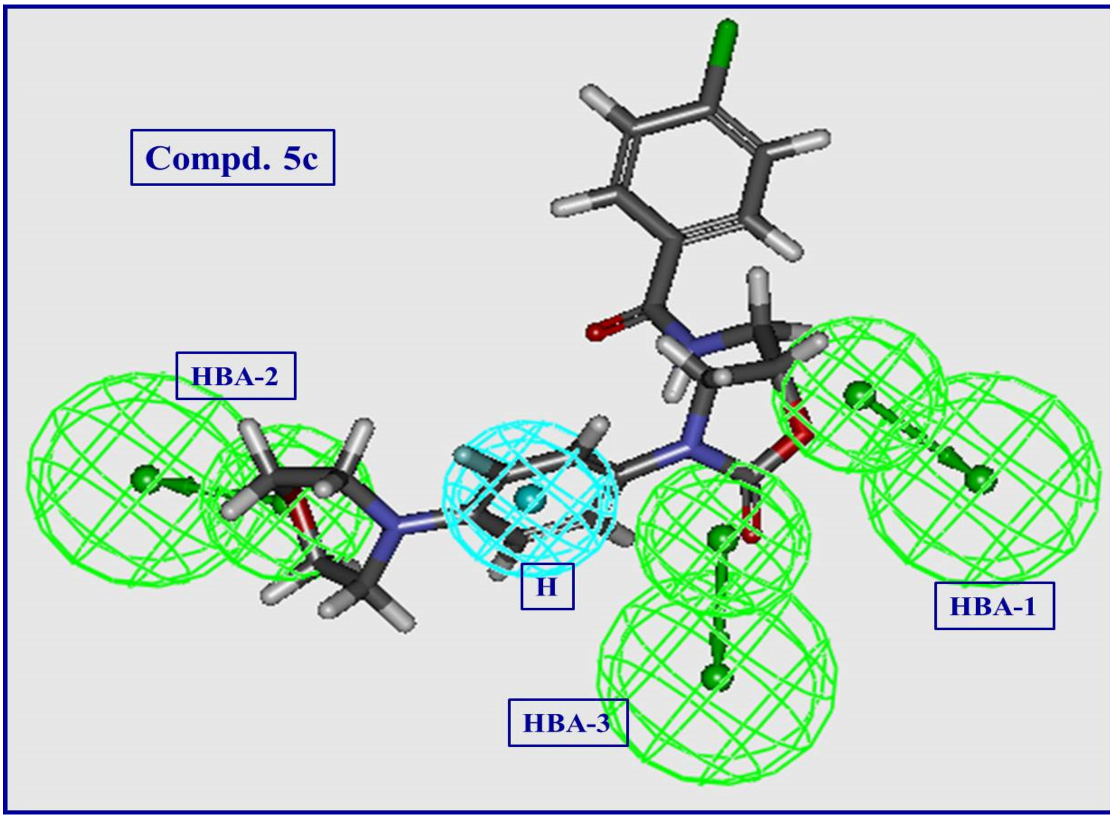
Compd. 5c
HBA-2
H
HBA-1
HBA-3

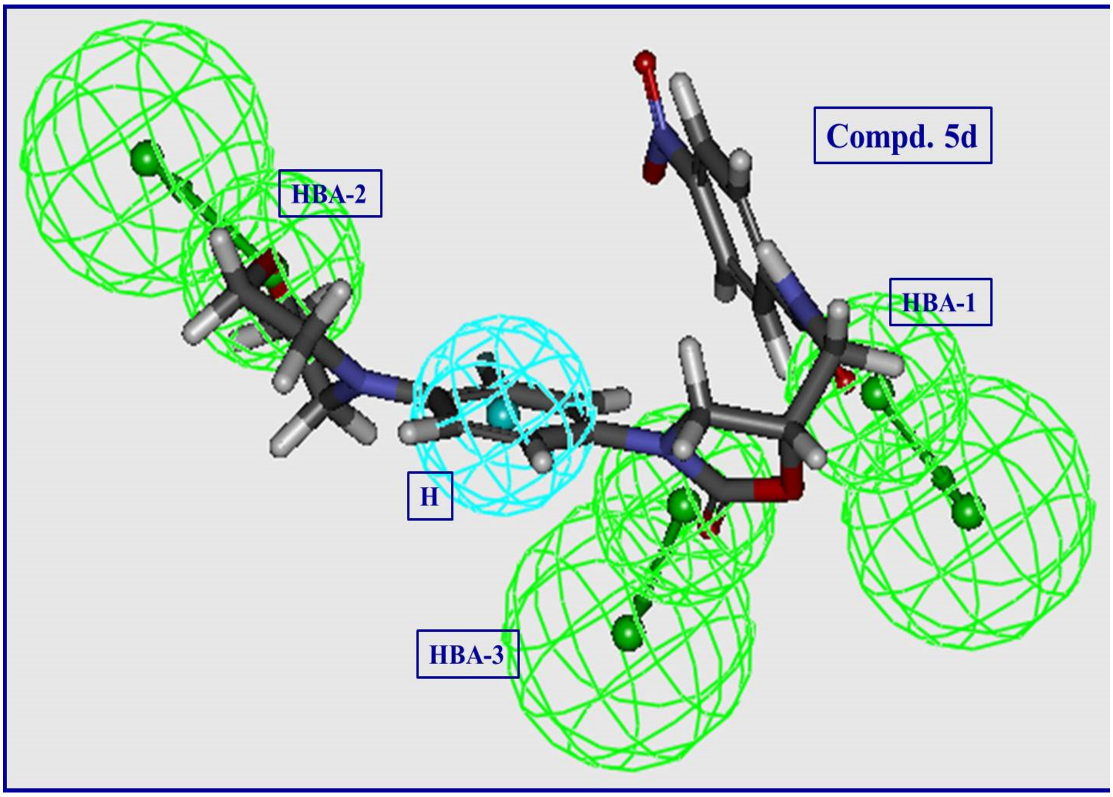
Compd. 5d
HBA-2
HBA-1
H
HBA-3

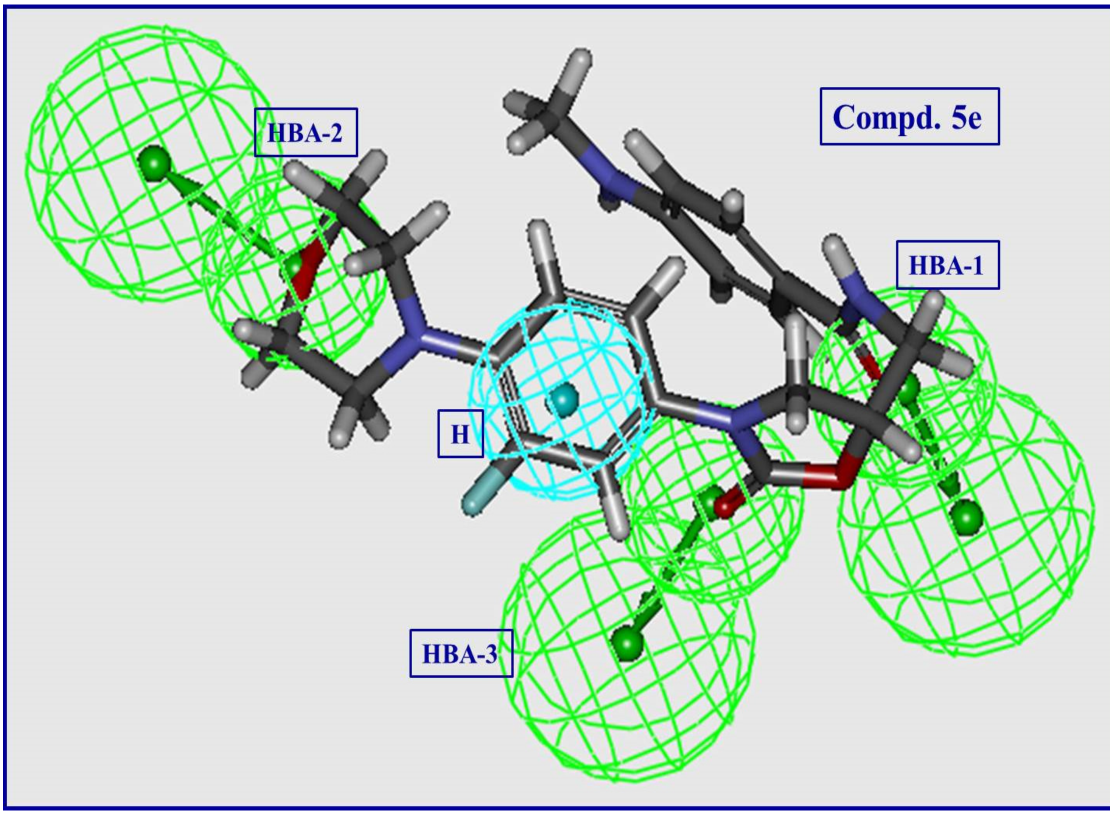
Compd. 5e
HBA-2
HBA-1
H
HBA-3

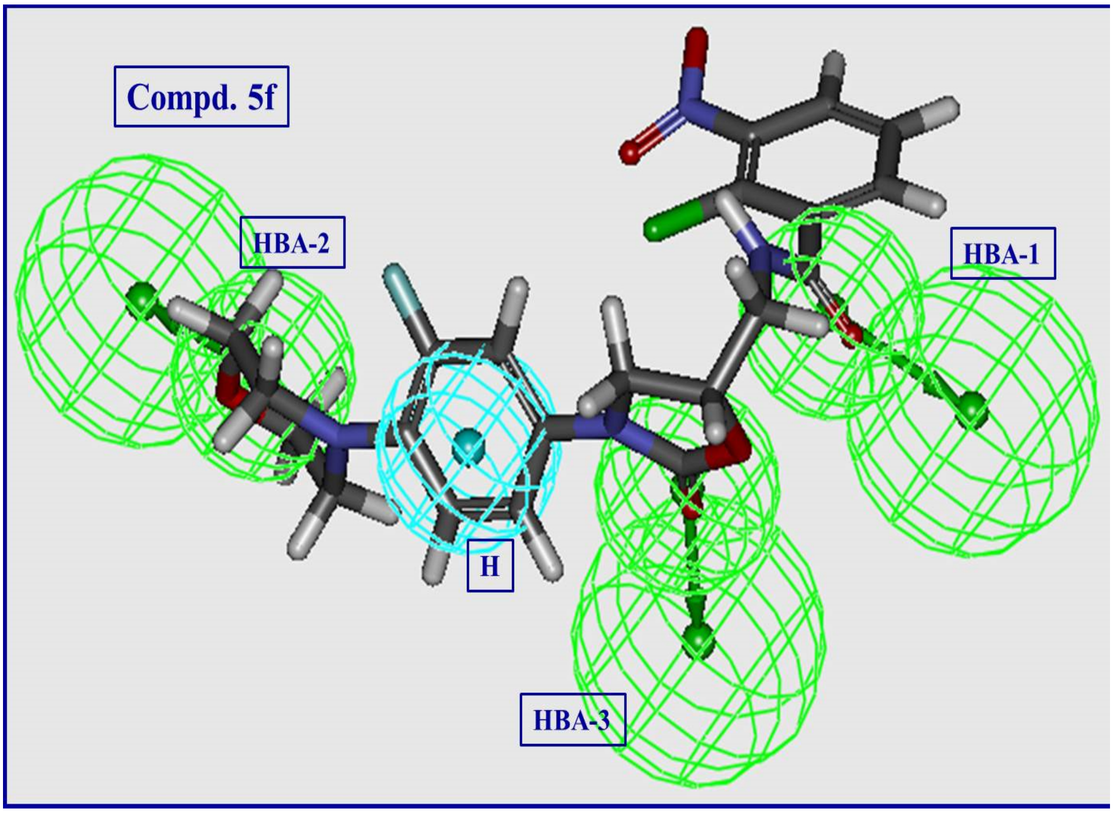

Compd. 5f
HBA-2
HBA-1
H
HBA-3

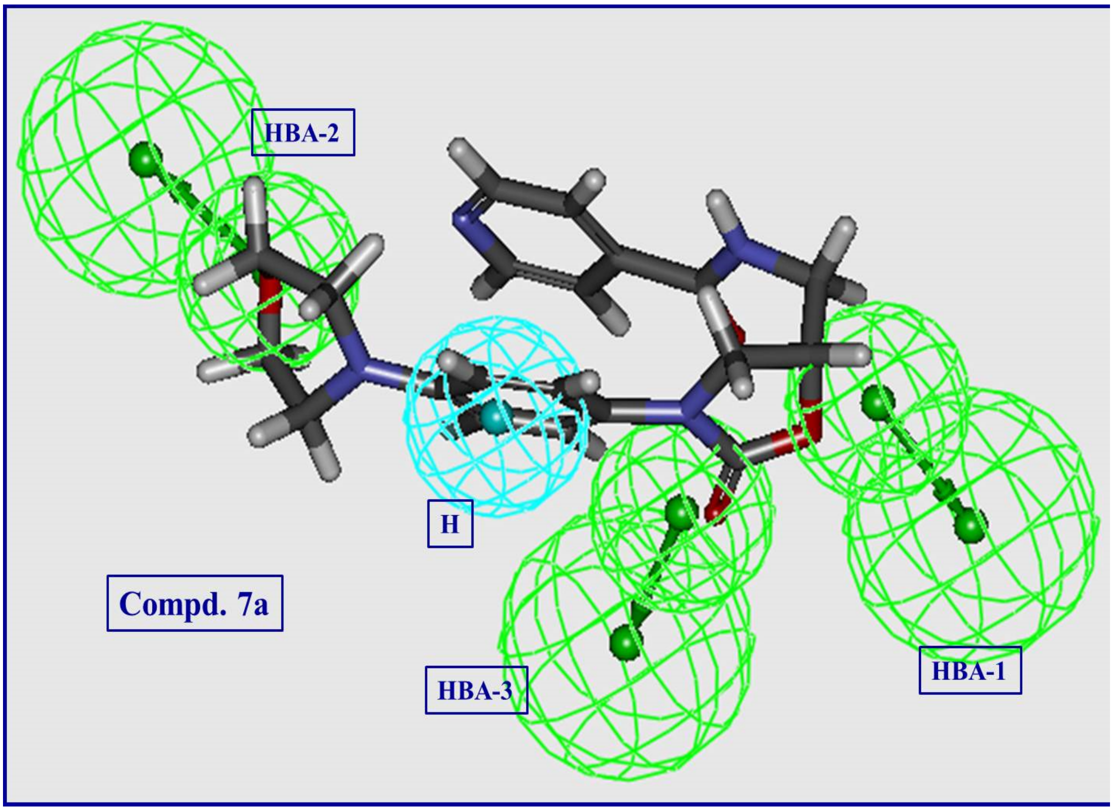

HBA-2
H
Compd. 7a
HBA-3
HBA-1

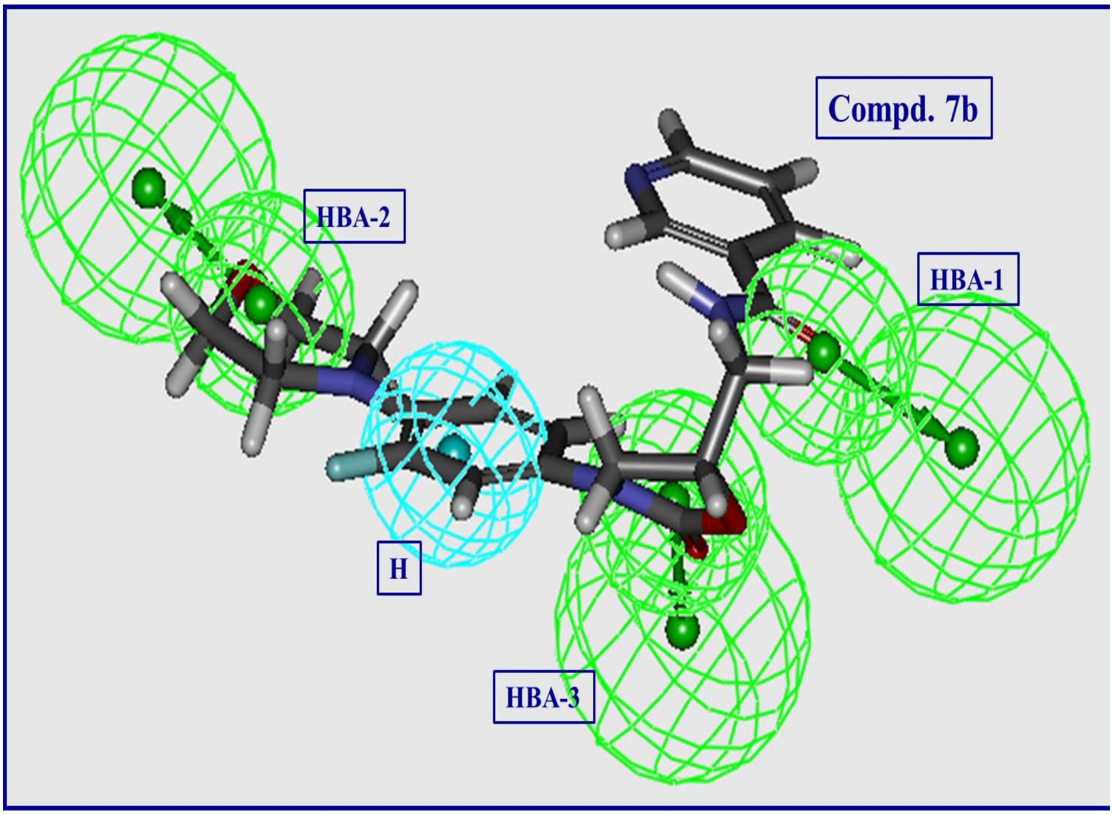
Compd. 7b
HBA-2
HBA-1
H
HBA-3

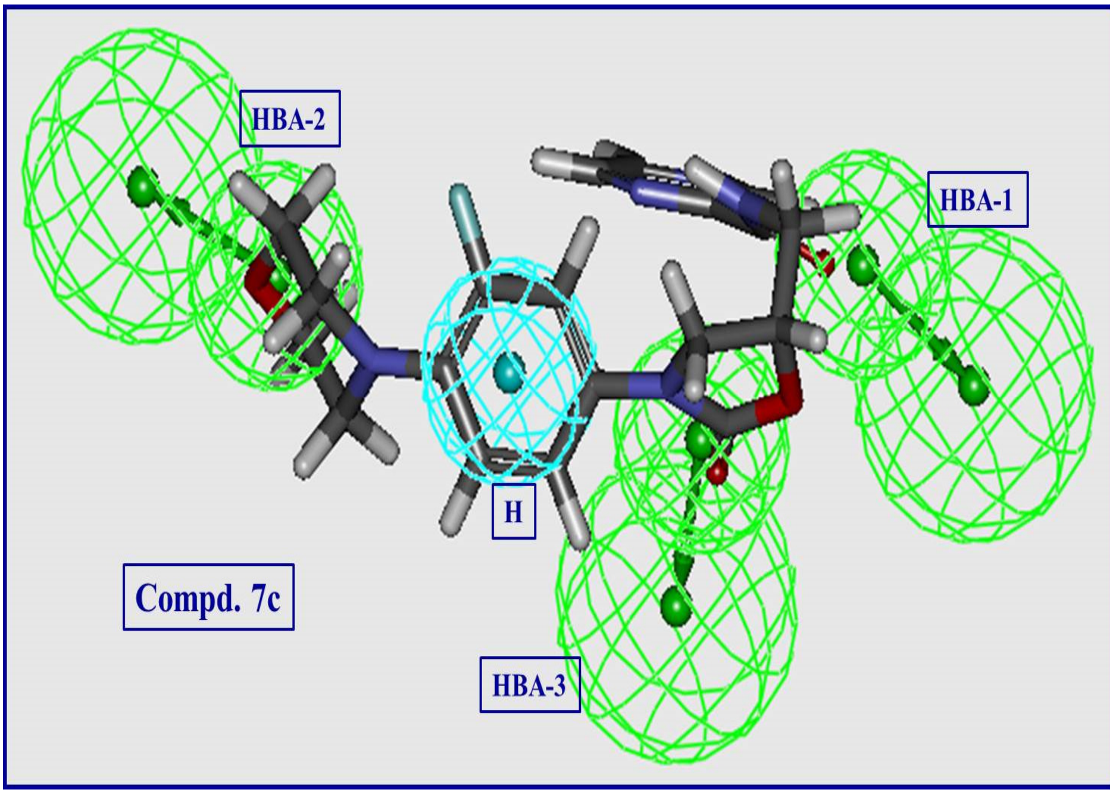
HBA-2
HBA-1
H
Compd. 7c
HBA-3

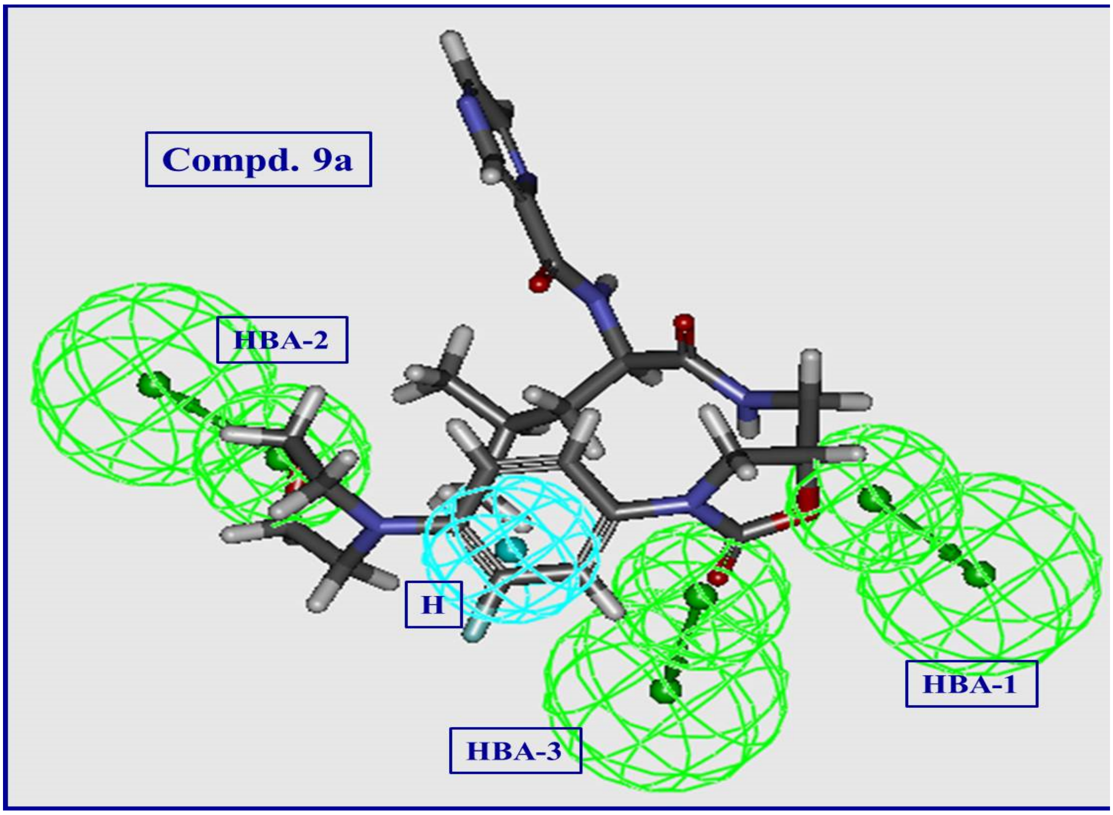
Compd. 9a
HBA-2
H
HBA-3
HBA-1

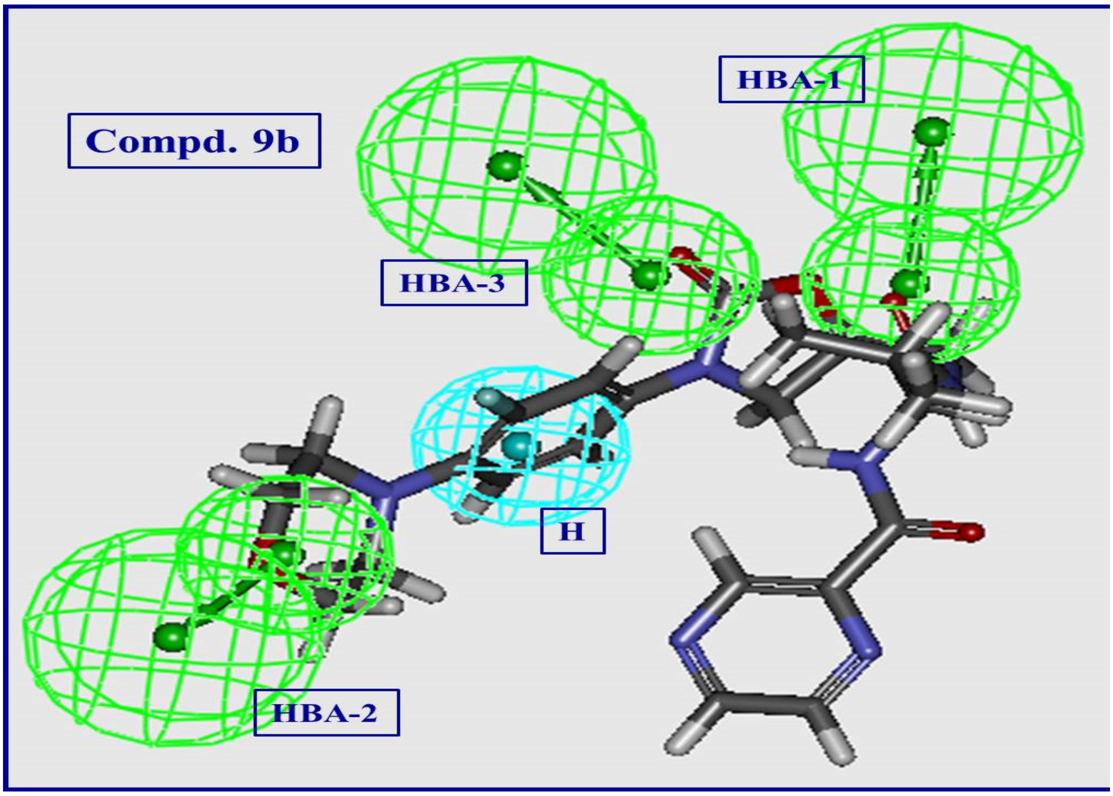
Compd. 9b
HBA-1
HBA-3
H
HBA-2

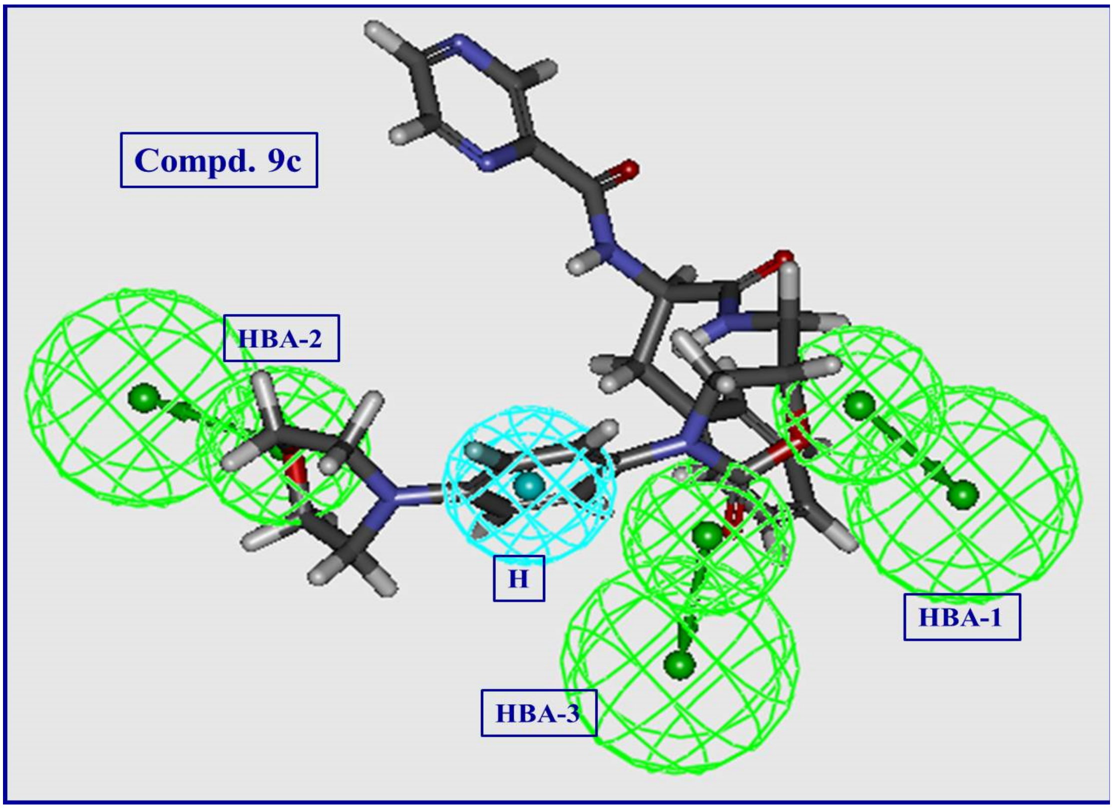
Compd. 9c
HBA-2
H
HBA-1
HBA-3

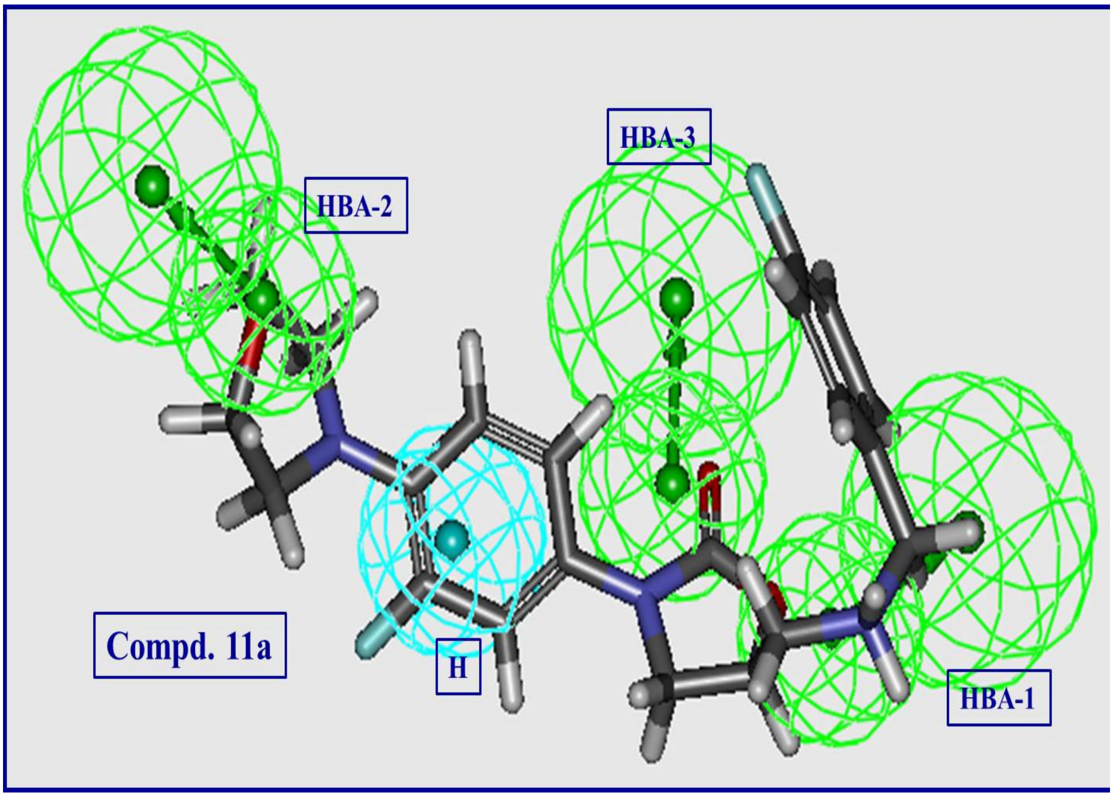
HBA-3
HBA-2
Compd. 11a
H
HBA-1

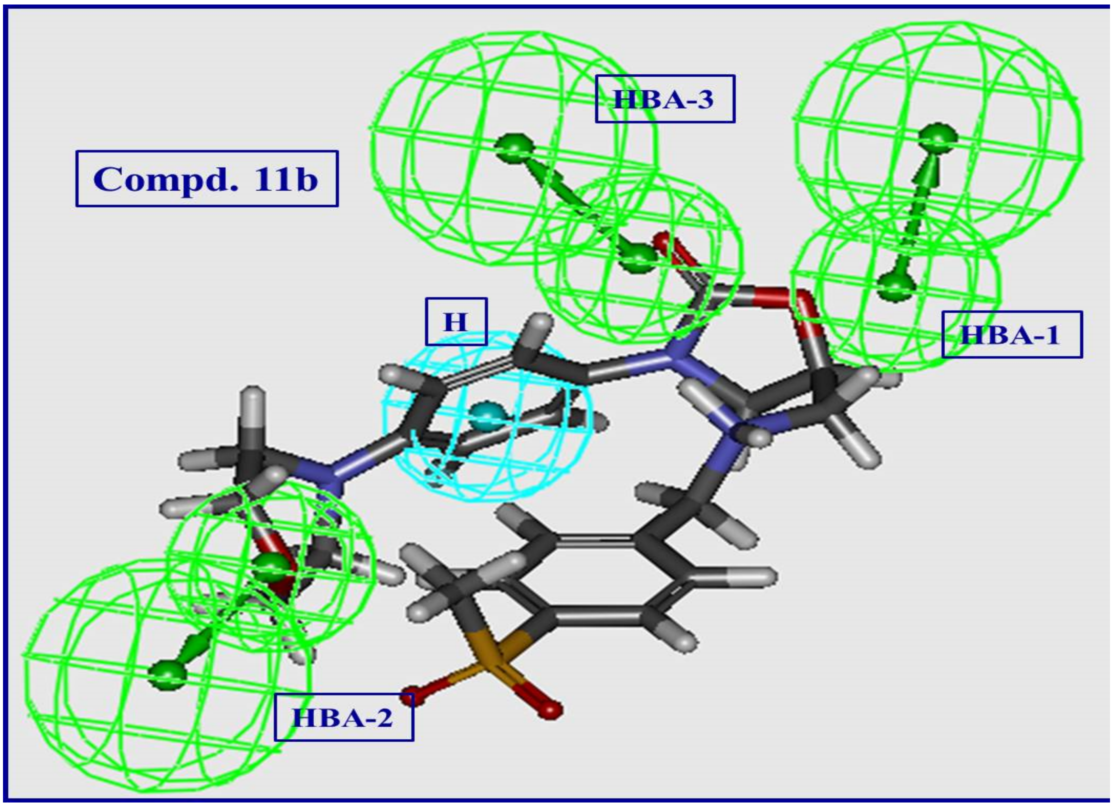

Compd. 11b
HBA-3
HBA-1
H
HBA-2

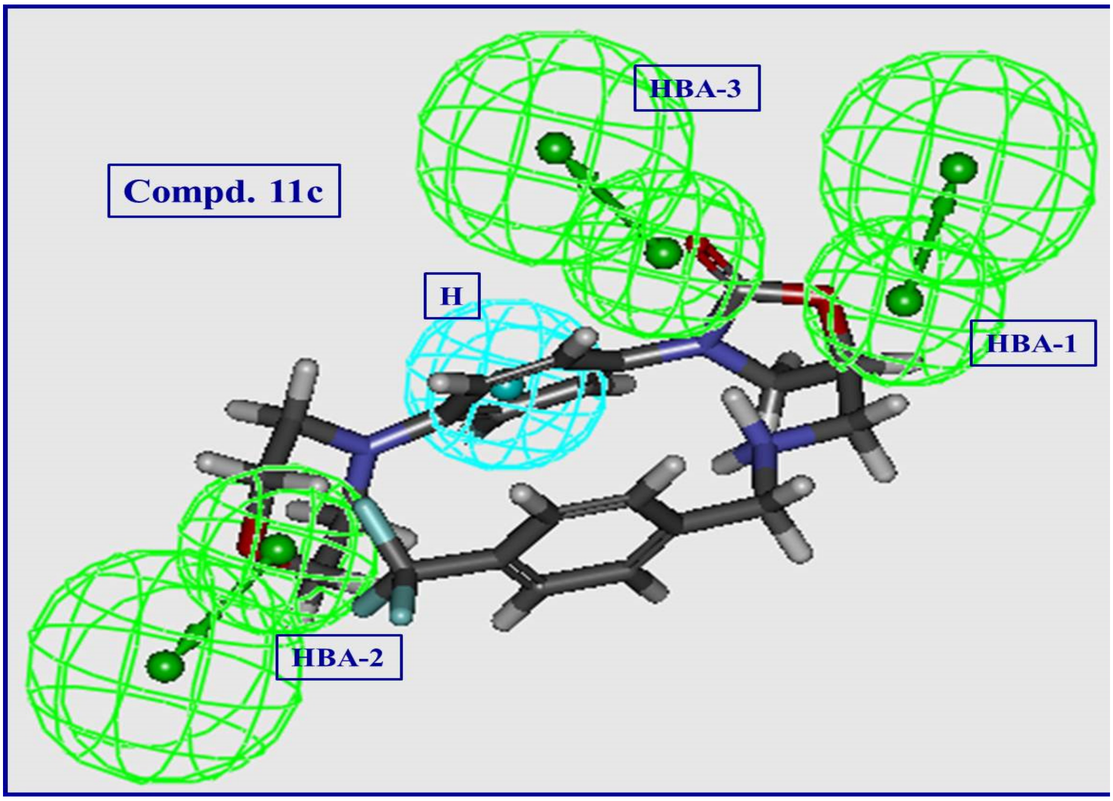

Compd. 11c
HBA-3
HBA-1
H
HBA-2

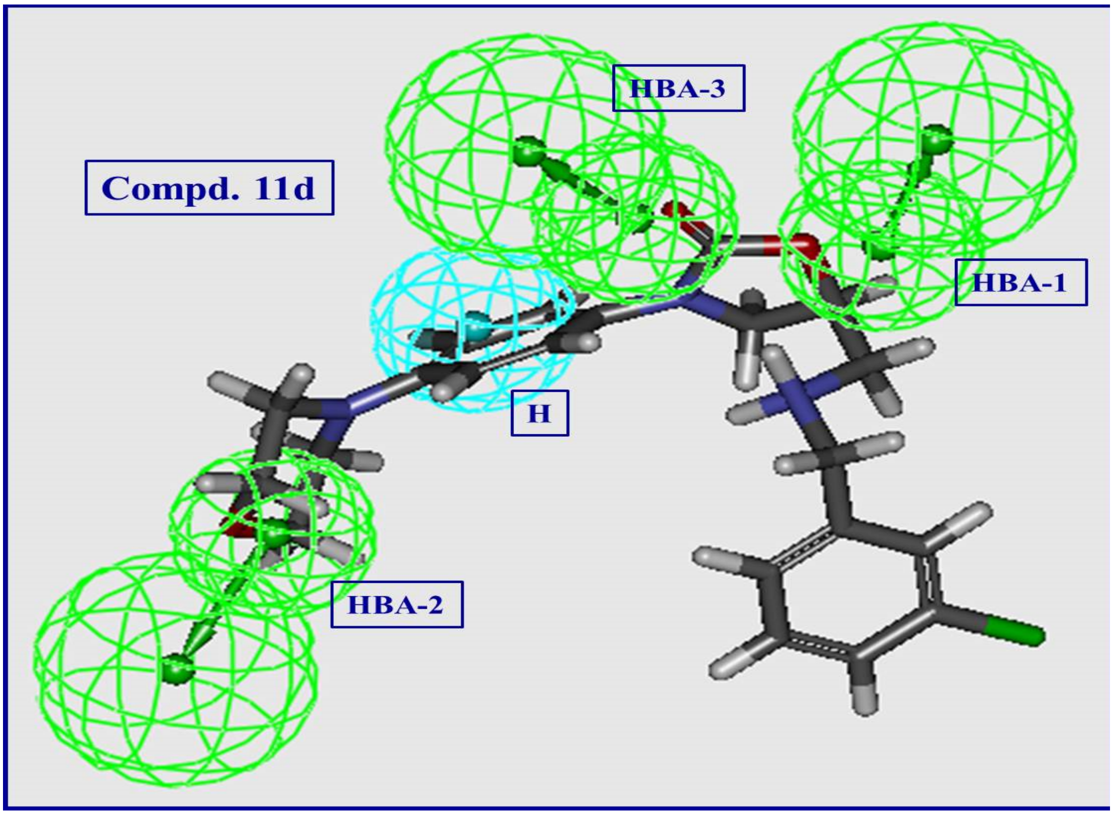
Compd. 11d
HBA-3
HBA-1
H
HBA-2

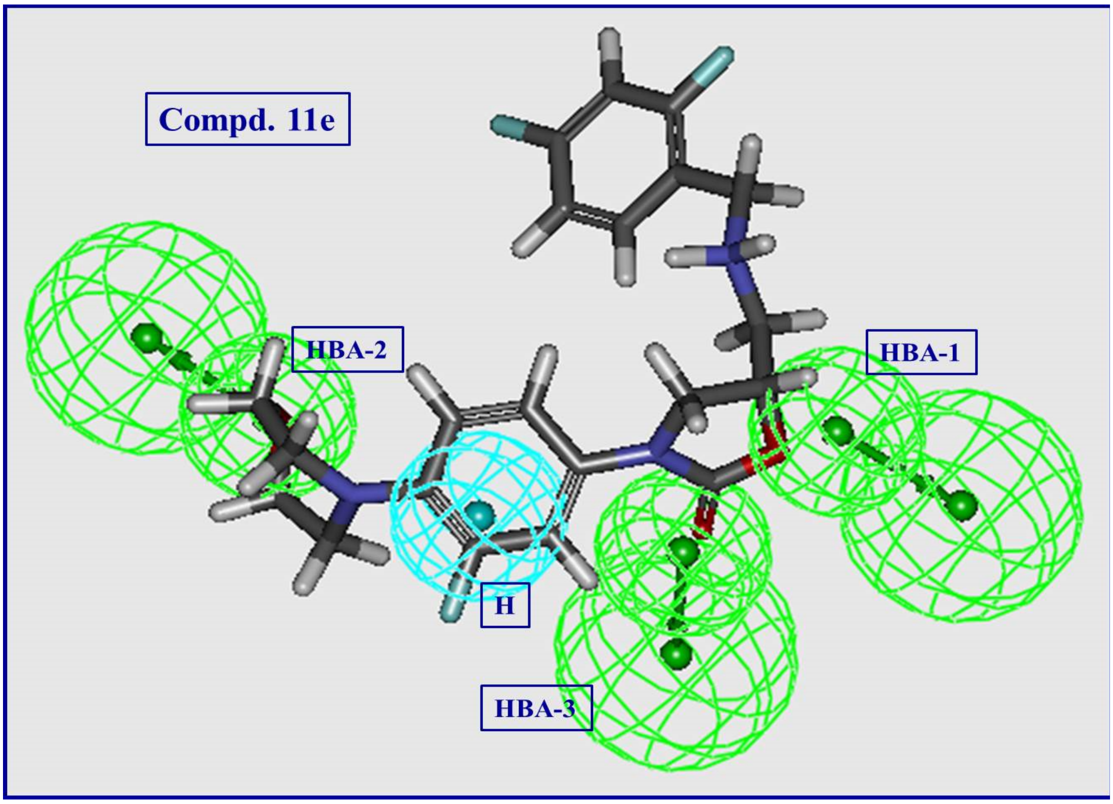
Compd. 11e
HBA-2
HBA-1
H
HBA-3

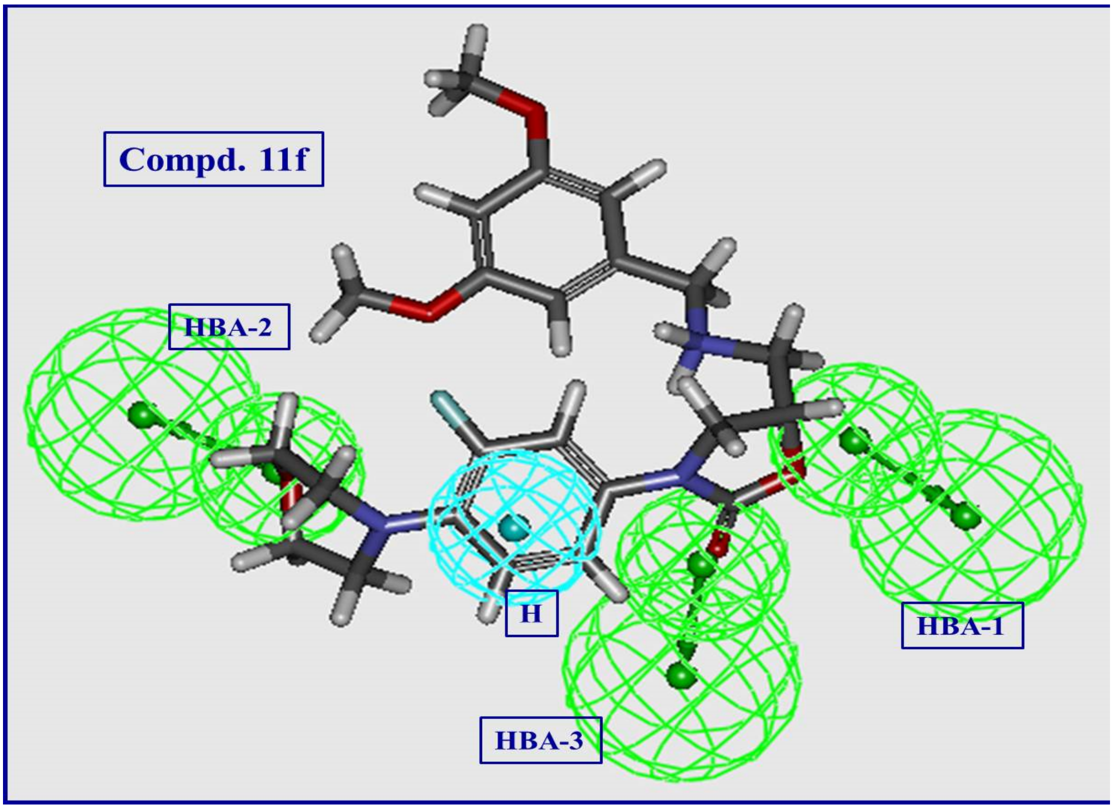
Compd. 11f
HBA-2
H
HBA-1
HBA-3

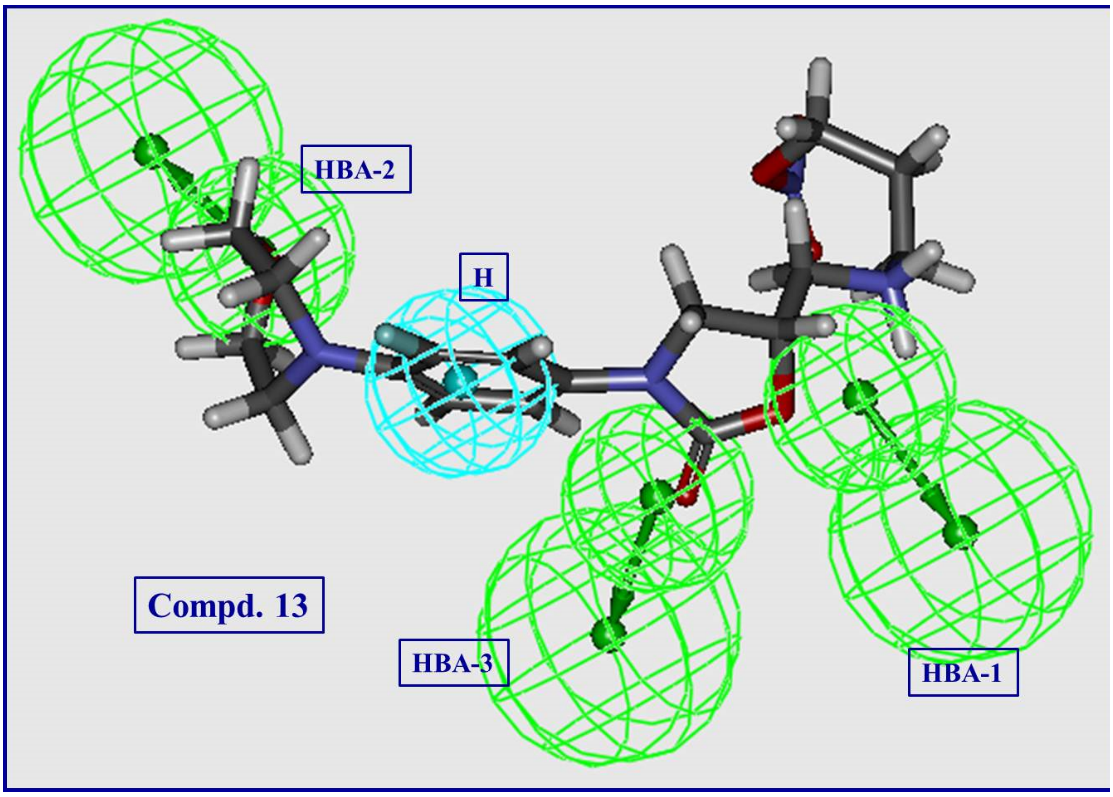
HBA-2
H
Compd. 13
HBA-3
HBA-1

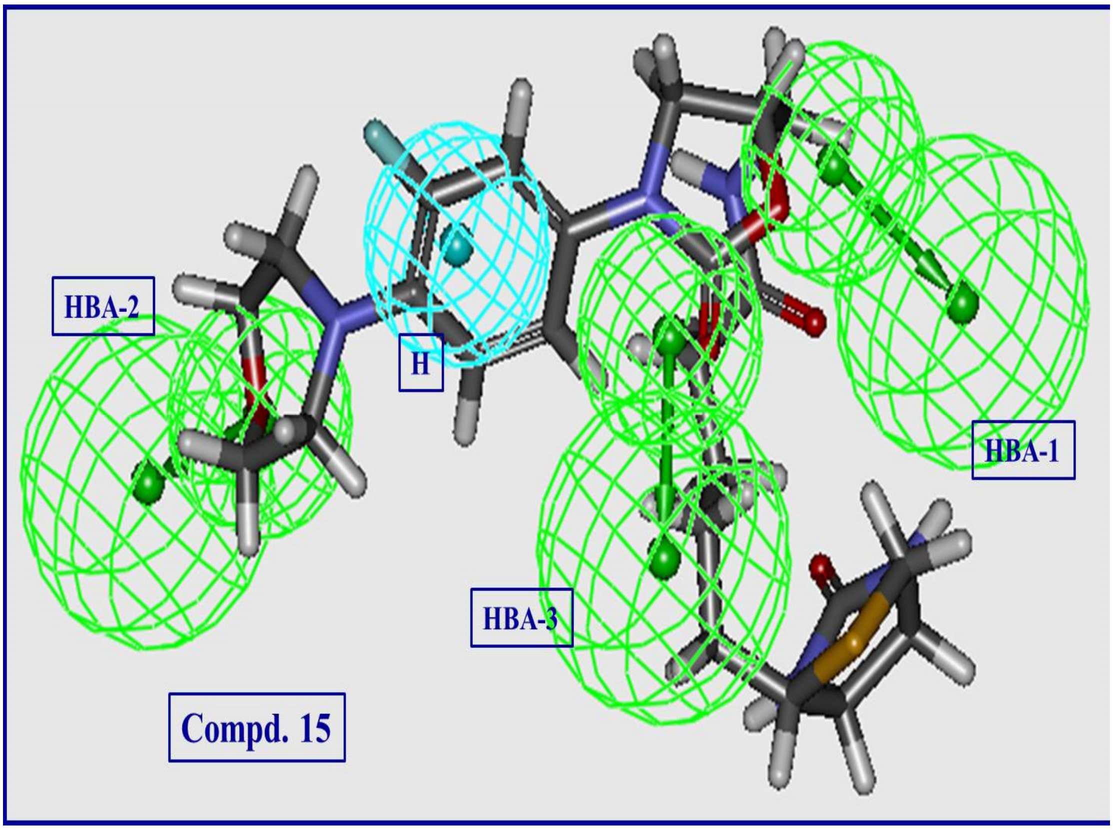

Fig. S7. 3D-pharmacophore model mapped on the tested compounds against *B. subtilis*.
